# Mining of RNA Methylation-Related Genes and Elucidation of Their Molecular Biology in Gallbladder Carcinoma

**DOI:** 10.3389/fonc.2021.621806

**Published:** 2021-02-25

**Authors:** Changhong Yang, Jialei Chen, Zhe Yu, Jing Luo, Xuemei Li, Baoyong Zhou, Ning Jiang

**Affiliations:** ^1^ Department of Bioinformatics, Chongqing Medical University, Chongqing, China; ^2^ Department of Otolaryngology, The First Affiliated Hospital of Chongqing Medical University, Chongqing, China; ^3^ School of Life Sciences, Peking University, Beijing, China; ^4^ Department of Pathology, Chongqing Medical University, Chongqing, China; ^5^ Department of Hepatobiliary Surgery, The First Affiliated Hospital of Chongqing Medical University, Chongqing, China

**Keywords:** gallbladder carcinoma, bioinformatics, biomarkers, differentially expressed genes, RNA methylation, bile metabolism

## Abstract

Gallbladder carcinoma (GBC), which has high invasion and metastasis risks, remains the most common biliary tract malignancy. Surgical resection for GBC is the only effective treatment, but most patients miss the opportunity for curative surgery because of a lack of timely diagnosis. The aim of this study was to identify and verify early candidate diagnostic and prognostic RNA methylation related genes for GBC *via* integrated transcriptome bioinformatics analysis. Lists of GBC-related genes and methylation-related genes were collected from public databases to screen differentially expressed genes (DEGs) by using the limma package and the RobustRankAggreg (RRA) package. The core genes were collected with batch effects corrected by the RRA algorithm through protein interaction network analysis, signaling pathway enrichment analysis and gene ranking. Four modules obtained from four public microarray datasets were found to be related to GBC, and *FGA*, *F2*, *HAO1*, *CFH*, *PIPOX*, *ITIH4*, *GNMT*, *MAT1A*, *MTHFD1*, *HPX*, *CTH*, *EPHX2*, *HSD17B6*, *AKR1C4*, *CFHR3*, *ENNP1*, and *NAT2* were revealed to be potential hub genes involved in methylation-related pathways and bile metabolism-related pathways. Among these, *FGA*, *CFH*, *F2*, *HPX*, and *PIPOX* were predicted to be methylated genes in GBC, but *POPIX* had no modification sites for RNA methylation. Furthermore, survival analysis of TCGA (the Cancer Genome Atlas) database showed that six genes among the hub genes, *FGA*, *CFH*, *ENPP1*, *CFHR3*, *ITIH4*, and *NAT2*, were highly expressed and significantly correlated with worse prognosis. Gene correlation analysis revealed that the *FGA* was positively correlated with the *ENPP1*, *NAT2*, and *CFHR3*, while *CFH* was positively correlated with the *NAT2*, *CFHR3*, and *FGA*. In addition, the results of immunohistochemistry (IHC) showed that the expressions of FGA, F2, CFH, PIPOX, ITIH4, GNMT, MAT1A, MTHFD1, HPX, CFHR3, NAT2, and ENPP1 were higher in GBC tissues than that in control tissues. In conclusion, two genes, *FGA* and *CFH*, were identified as RNA methylation-related genes also involved in bile metabolism in GBC, which may be novel biomarkers to early diagnose and evaluate prognosis for GBC.

## Introduction

Gallbladder carcinoma (GBC), which originates from the epithelia of bile ducts and the gallbladder, is the most common malignancy of the biliary tract and has a high possibility of metastasis (1). At present, the 5-year survival rate of patients with unresectable GBC is less than 5%, and complete surgical resection is still an effective curative therapy (2). However, due to a lack of specific signs and symptoms, it is very difficult to diagnose GBC at the early stage; thus, many patients miss the opportunity for surgery (3). A more in-depth understanding of the molecular mechanisms underlying the progression of gallbladder carcinoma will be advantageous for the development of treatment options. Thus, further exploration of GBC pathological development and identification of effective early prognostic biomarkers are important for GBC treatment.

RNA methylation is the most common modification of mRNAs (4) and is dynamic and reversible in mammalian cells (5). The dynamic regulation of RNA methylation has been indicated to be closely associated with gene expression (6, 7). Growing evidence has demonstrated that RNA methylation is involved in regulating RNA transcription (8), processing events (9), RNA stability (10), and translation (11). In addition, the clinical value of RNA methylation in cancers has become increasingly obvious. RNA methylation modification is reported to be associated with proliferation (12), tumorigenesis (13), invasion (13), and metastasis (14) in various cancers. In addition, RNA methylation not only affects the cleavage, transport, stability, and degradation of non-coding RNAs such as miRNAs, lncRNAs, and circRNAs but also regulates the biological functions of cells by regulating the levels of these non-coding RNAs (15). More importantly, non-coding RNAs can influence RNA-RNA or RNA-protein interactions to regulate particular biological functions (16). As a promising biomarker, RNA methylation has been increasingly utilized to detect and predict the occurrence of cancer, and its prognostic significance has been determined (17). Increasing numbers of studies have illustrated that RNA methylation may have potential clinical value as a therapeutic target for cancer patients (18). However, research on RNA methylation in GBC is still very scarce.

Previous studies on GBC have seemed to be limited and have focused mostly on either a single gene or a single omics data type (19, 20). However, the occurrence and development of GBC is a multifactorial and multistep process involving molecular changes at the transcriptional, posttranscriptional, and translational levels. Thus, there is an urgent need to comprehensively illustrate the gene interactions and molecular modulation network of GBC. With the rapid development of high-throughput technologies, bioinformatics has been widely applied to analyze massive amounts of biological data. Transcriptomics techniques include mainly microarrays and RNA sequencing, and microarrays include expression profile chips, lncRNA chips, miRNA chips, and methylation chips. Ma et al. (19) identified differentially expressed lncRNAs and mRNAs between GBC tissues and control tissues with chips and found that the lncRNA GCASPC could bind to miRNA-17-3 to negatively regulate the proliferation of GBC cells. Liang et al. (20) used a microRNA chip with high-throughput screening to discover that miRNA-143-3p inhibited the proliferation of GBC cells by binding to its target gene *MYBL2*. Currently, the most-studied pathways involved in GBC are the hedgehog pathway (21) and the PI3K/Akt pathway (22). Studies on RNA methylation in gallbladder carcinoma have not been reported, and integrated bioinformatics analysis using multiple public databases in gallbladder cancer is quite scarce.

In this study, we employed integrated transcriptome bioinformatics analysis based on four RNA microarray datasets (GSE45001, GSE31370, GSE26566, and GSE76633) to identify differentially expressed genes and RNA methylation-related genes in GBC. In addition, the core genes were collected through protein-protein interaction network analysis, signaling pathway enrichment analysis, and gene ranking. Moreover, the core genes were verified with the TCGA database, and posttranscriptional modifications, survival, a coexpression network, and the tumor microenvironment were predicted. Then, immunohistochemistry was used to detect the differences in the expression of the hub genes between the clinical GBC samples and control samples. The final screened genes could be novel biomarkers to early diagnose and evaluate prognosis for GBC.

## Methods

**Figure 1:**
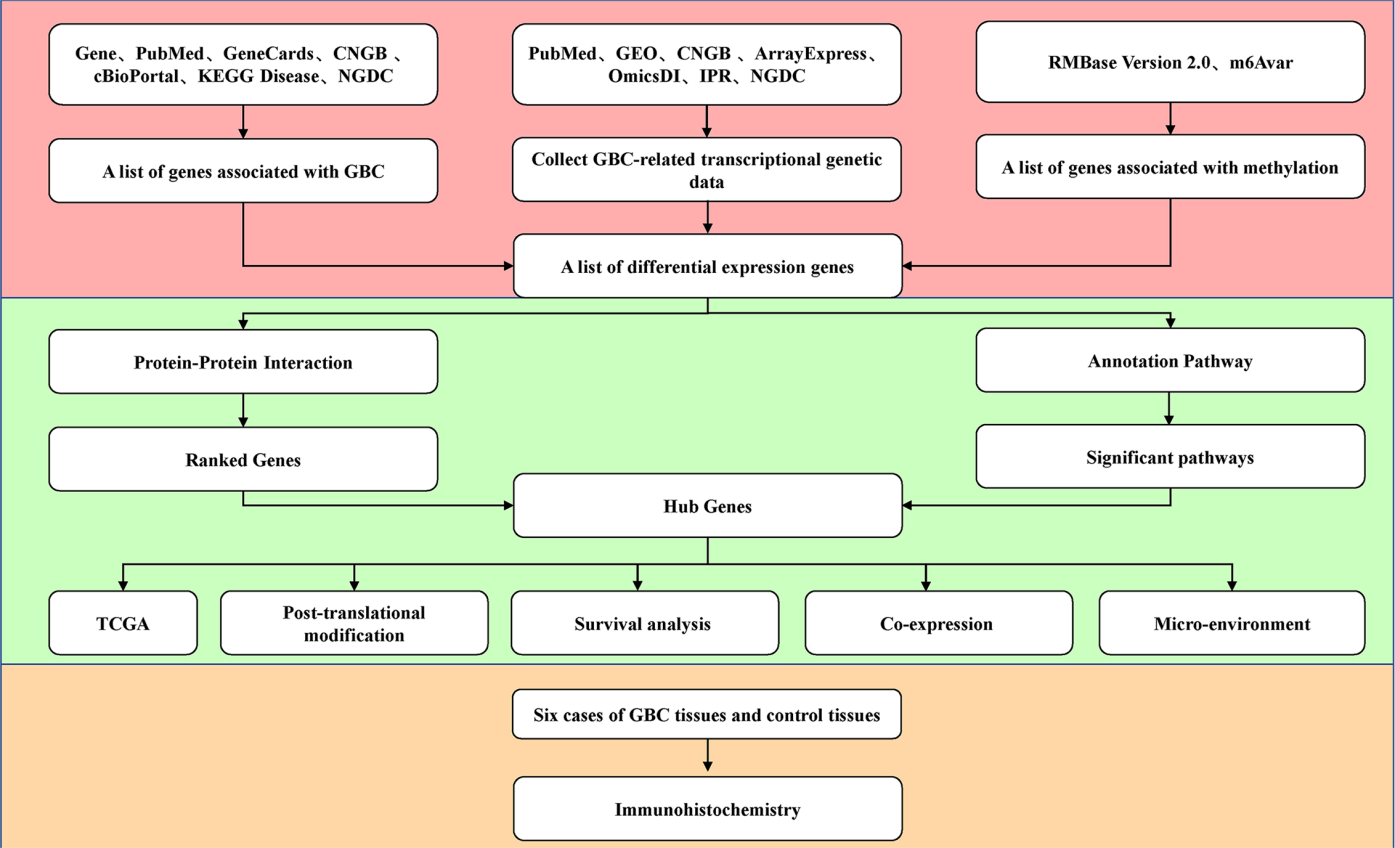
Workflow chart of mining of RNA methylation-related genes in gallbladder carcinoma.

GBC-related gene lists and related genetic data were collected from public databases. Methylation-related genes were also obtained to screen the differentially expressed genes. The core genes were collected through protein interaction network analysis, signaling pathway enrichment analysis, and gene ranking. They were verified with the TCGA database, and posttranscriptional modifications, survival, and a coexpression network were predicted. Then, immunohistochemistry was used to detect the expression of the core genes between the clinical GBC samples and control samples. The flow chart is shown in **Figure 1**.

### Data Sources

GBC-related genes were collected with the search terms “((gallbladder cancer) OR (gallbladder carcinoma) OR (gallbladder neoplasms) OR (cholecystic carcinoma) OR (biliary tract cancer)) AND (Homo sapiens[Organism])” from the NCBI Gene database, the GeneCards database, the KEGG DISEASE database, cBioPortal database and the National Gene Bank. In addition, GBC-related genes were obtained from the literature with the keywords “(gallbladder cancer[Title/Abstract]) OR (gallbladder carcinoma[Title/Abstract])) OR (gallbladder neoplasms[Title/Abstract]) OR (cholecystic carcinoma[Title/Abstract])) OR (biliary tract cancer[Title/Abstract])) AND (biomarker[Title/Abstract])” from NCBI PubMed. Next, GBC-related microarray data were collected with the search terms “((gallbladder cancer) OR (gallbladder carcinoma) OR (gallbladder neoplasms) OR (cholecystic carcinoma) OR (biliary tract cancer)) AND (Homo sapiens[Organism])” from the GEO (Gene Expression Omnibus) database, PubMed, ArrayExpress of the EBI, the National Gene Bank, the National Genomics Science Data Center, OmicsDI, and Integrated Proteome Resources. Finally, all differentially methylated genes in GBC were obtained from the RMBase v2.0 database and the m6AVar database.

### Methods for Identification of Differentially Expressed Genes

The Linear Models for Microarray Data (limma) package, which is a Bioconductor package of the R statistical language, was used to identify differentially expressed genes in six main steps. First, the expression matrix, grouping matrix and differential expression matrix were constructed. Second, the data were fitted to a linear model with the lmFit function. Third, the differences were calculated according to the contrast model with the contrasts.fit function. Fourth, the Bayesian test was conducted with the eBayes function. Fifth, test results were generated for all genes with the topTable function, and Benjamini and Hochberg test method was used to correct the *P* value. Finally, the results of differential analysis were screened according to a threshold of a corrected *P* value <0.050. RobustRankAggreg, another R package, is a tool that integrates differential expression analysis results from different platforms mainly with the RobustRank Aggregation (RRA) algorithm to obtain a comprehensive ranking list (23). To identify differentially expressed genes in this study, data were first downloaded from the GEO database. Limma software was then used to analyze the differentially expressed genes in each microarray dataset. The differentially expressed genes according to the fold change value were sorted. Finally, RobustRankAggreg software was used to integrate and analyze these results with the RRA algorithm.

### Protein-Protein Interaction Network and Module Analysis

To better mine the core regulatory genes, protein-protein interaction analysis was used in this study. First, STRING (version 11, https://string-db.org/) software was used to analyze the protein interactions of the differentially expressed genes. Next, the CytoHubba plugin of Cytoscape software (version 3.7.2, https://cytoscape.org/) was used to rank the genes in the network with the Degree algorithm, and the top 25 ranked genes were considered the hub gene set. Simultaneously, the MCODE plugin was used to extract the core module from the protein interaction results with the following parameters: degree cutoff >=2, K -score >=2.

### Functional Enrichment Analysis

ShinyGO V0.60 software was used to analyze the signaling pathways of the core module obtained from protein interaction analysis (24). ShinyGO software, a gene set enrichment analysis software program, is based on 55 kinds of databases, such as the Gene Ontology (GO), KEGG, Reactome, Panther, Biocarta, GeneSetDB EHMN, HumanCyc, NetPath, and MSigDB databases. ShinyGO software was used to functionally annotate genes or proteins with pathway databases, and the FDR (false discovery rate) values of the corresponding enriched pathways were obtained. The ggplot2 package was used to visualize the enrichment analysis results.

### Annotation of Core Genes

The core genes in the hub module of the protein-protein interaction network that overlapped with genes involved in significant biological processes were annotated. An RNA sequencing dataset for CHOL including data on 36 patients with GBC and 9 controls were downloaded from the TCGA (25), and the limma package of the R language was used to analyze differentially expressed genes in order to verify the hub genes. In addition, the RMBase v2.0 database (26), which is a database of epigenetic modifications at the RNA level, and the m6AVar database (27), which predicts the impact of SNPs on RNA methylation modification, were used to predict the sites of RNA methylation modification of the core genes.

### Survival Analysis of Hub Genes

Different endpoints that were stratified by mean and median, such as overall survival (OS), were used to analyze the prognosis of survival. Survival curves were assessed by the Kaplan-Meier method and Cox proportional hazards model. The hazard ratios (HRs) with 95% confidence intervals were determined.

### Coexpression Network and Microenvironment Prediction

The GeneMANIA plug-in of Cytoscape software (version 3.7.2) was applied to analyze the gene coexpression network. The strength of regulation among genes was represented in the coexpression network through a weight value ranging from 0 to 1. The Tumor Immune Single-cell Hub (TISCH) database (http://tisch.comp-genomics.org/home/) (28) was used to analyze the tumor microenvironment.

### Clinical Specimen Collection

Six samples of tumor tissues from patients with GBC and six samples of gallbladder tissues from patients with gallstones were obtained from the First Affiliated Hospital of Chongqing Medical University from 2019 to 2020, and all tissues were fixed with 4% paraformaldehyde. All patients agreed to provide informed consent, and the experimental protocols were approved by the local ethics committee.

### Immunohistochemistry and Staining Result Determination

All specimens were paraffin-embedded and sectioned at 4 μm. The sections were baked at 60°C for 1 h, dewaxed, rehydrated in a graded alcohol series, and washed. The sections were boiled for 10 min antigen repair solution for antigen repair, and the remaining steps were carried out in accordance with the instructions. Primary antibodies against the following proteins were purchased: FGA (1:50, Boster, China), F2 (1:50, Boster, China), CFH (1:50, Boster, China), PIPOX (1:50, Absin, China), ITIH4 (1:50, Proteintech, USA), GNMT (1:50, Proteintech, USA), MAT1A (1:50, Fine Biotech, China), MTHFD1 (1:50, Proteintech, USA), HPX (1:50, Boster, China), CTH (1:50, Boster, China), CFHR3 (1:50, Fine Biotech, China), ENNP1 (1:50, Abcam, USA), and NAT2 (1:50, Abclonal, China). After DAB staining, the tablets were redyed with hematoxylin and sealed. The proportion of positive tumor cells was scored as follows: 1, 1–25%; 2, 26–50%; 3, 51–75%; and 4, 76–100%. The intensity of methylated protein staining in GBC was scored as follows: 0, no staining; 1, weak staining; and 2, strong staining. These two kinks of scores were multiplied to obtain a final score, and the expression of protein was determined to be low for scores <4 or high for scores ≥4.

### Statistical Analysis

All statistical analyses were performed with R software (version 3.6.3). The threshold for screening of differentially expressed genes was an adjusted *P* value <0.05, an absolute fold change >1, and an RRA score <0.05. The methods of statistical analysis in this study included the hypergeometric test and Fisher’s exact test, and the false discovery rate method was used with Benjamini and Hochberg correction. Correlations were assessed using Pearson’s correlation coefficient.

## Results

### Construction of Lists of Related Genes

**Figure 2:**
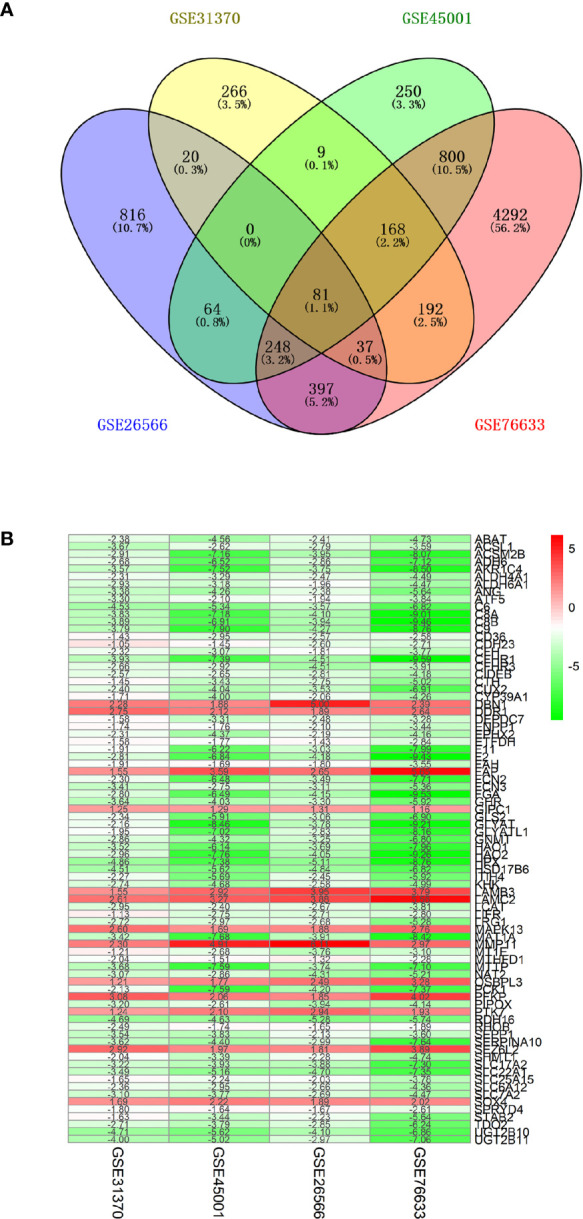
Collection of differentially expressed genes (DEGs) in GBC. **(A)** Venn diagram of four RNA microarray datasets (GSE45001, GSE31370, GSE26566, and GSE76633). **(B)** heat map of differentially expressed genes. Each column represents one dataset and each row represents one gene. Green represents a lower expression level, red represents higher expression levels, and white represents that there is no different expression amongst the genes. The number in each rectangle represents the normalized gene expression level.

GBC-related genes were collected from the following sources: 415 from the Gene database of NCBI, 415 from the GeneCards database, 4 from the KEGG DISEASE database, 1,637 from the cBioPortal gene database, 100 from the National Gene Bank, and 151 from related literature in the PubMed database. After merging these gene sets and removing the redundancies, we obtained 6,026 genes related to GBC. Next, 9,569 RNA methylation-related genes were collected from the RMBase v2.0 database, while 3,472 RNA methylation-related genes were collected from the m6AVar database. Together, 11,581 RNA methylation-related genes were collected. The overlap of the above GBC-related genes and the RNA methylation-related genes was examined, and 3,072 GBC-related genes involved in RNA methylation were ultimately obtained, as shown in **Figure 2**. RNA microarray datasets (GSE45001, GSE31370, GSE26566, and GSE76633) were downloaded from GEO database (29), as shown in **Table 1**. There were 159 samples with transcriptomic data, including 129 GBC samples and 30 control samples.

**Table 1:** Information of the four microarray datasets from GEO.

GEO ID	Sample_Case	Sample_Control	Sample Total
**GSE26566**	104	6	110
**GSE31370**	6	5	11
**GSE45001**	10	10	20
**GSE76633**	9	9	18

GEO, Gene Expression Omnibus.

### Collection of Differentially Expressed Genes (DEGs) in GBC

Four microarray datasets with group correction and normalization were used to analyze the differentially expressed genes. The specific numbers of upregulated and downregulated genes for each set are shown in **Supplementary Table 1**. The four datasets were intersected, and the genes were ranked according to the fold change value and integrated with the RRA algorithm of the RobustRankAggreg package to obtain 81 DEGs (score <0.050), including 13 upregulated genes and 68 downregulated genes (control *vs* GBC) (shown in **Figure 2B**).

### Establishment of the PPI Network

Based on the STRING database, a PPI network of the above 81 DEGs was constructed and ranked with topological analysis on nodes. The top 25 genes were clustered with MCODE to finally obtain four modules, as shown in **Table 2**.

**Table 2:** Top 25 hub genes identified in PPI network for DEGs.

Rank	Name	Score	MCODE_Cluster	MCODE_Score
1	FGA	15	Cluster 1	6.000
2	F2	14	Cluster 1	6.000
3	C8A	13	Cluster 1	6.000
4	C8B	11	Cluster 1	6.000
5	C9	10	Cluster 3	4.464
6	C6	9	Cluster 1	6.000
6	HAO1	9	Cluster 1	5.000
6	UGT2B10	9	Cluster 1	5.000
9	SERPINA10	8	Cluster 1	6.000
9	HAO2	8	Cluster 2	4.000
12	ANG	7	Cluster 1	5.000
12	CFH	7	Cluster 1	5.000
12	F11	7	Cluster 1	6.000
12	PIPOX	7	Cluster 2	4.000
12	ITIH4	7	Cluster 3	4.000
17	SLC17A2	5	Cluster 1	5.000
17	GNMT	5	Cluster 2	4.000
17	MAT1A	5	Cluster 2	4.000
17	MTHFD1	5	Cluster 2	4.000
17	HPX	5	Cluster 3	4.000
22	CTH	4	Cluster 2	4.000
22	EPHX2	4	Cluster 2	4.000
22	UGT2B11	4	Cluster 4	3.000
25	AKR1C4	3	Cluster 4	3.000
25	HSD17B6	3	Cluster 4	3.000

PPI, protein-protein interaction; DEG, differentially expressed genes.

### GO, KEGG, and Reactome Enrichment Analysis

**Figure 3:**
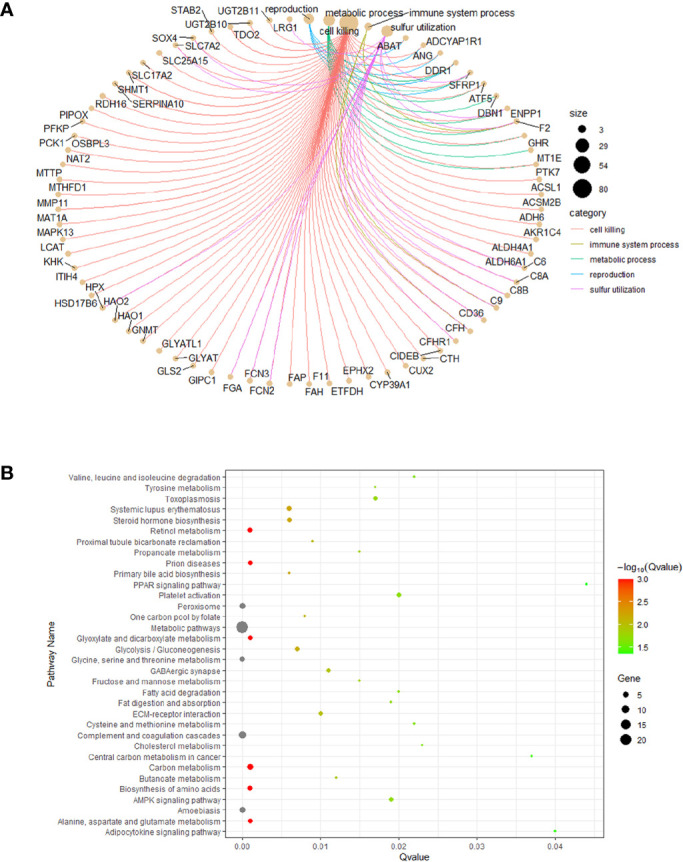
Functional enrichment analysis. **(A)** co-expression of the DEGs. **(B)** the DEGs enriched pathway analysis.

Almost 2.30~48.28% of the gene products were found to be associated with 33 biological process terms, such as the extracellular, cell surface, and mitochondria terms (enrichment FDR: 2.683E-10 ~ 3.348E-02). Approximately 2.30~35.63% of the genes were found to be associated with 87 molecular biology terms, including the REDOX enzyme activity, drug binding, and signal receptor binding terms (enrichment FDR: 6.936E-06 ~ 4.886E-02). Nearly 2.30~42.53% of the genes were associated with 400 biological process terms, such as the cholic acid biosynthesis, cholic acid metabolism, JAK-stat cascade, vascular development, inflammatory response, and cell adhesion terms (enrichment FDR: 1.022E-16 ~ 4.950E-02), as shown in **Figures 3A, B**. Overall, we obtained 23 genes involved in biological processes related to RNA methylation, and 12 of those genes were involved in biological processes related to bile metabolism.

### Annotation of Core Genes

**Figure 4:**
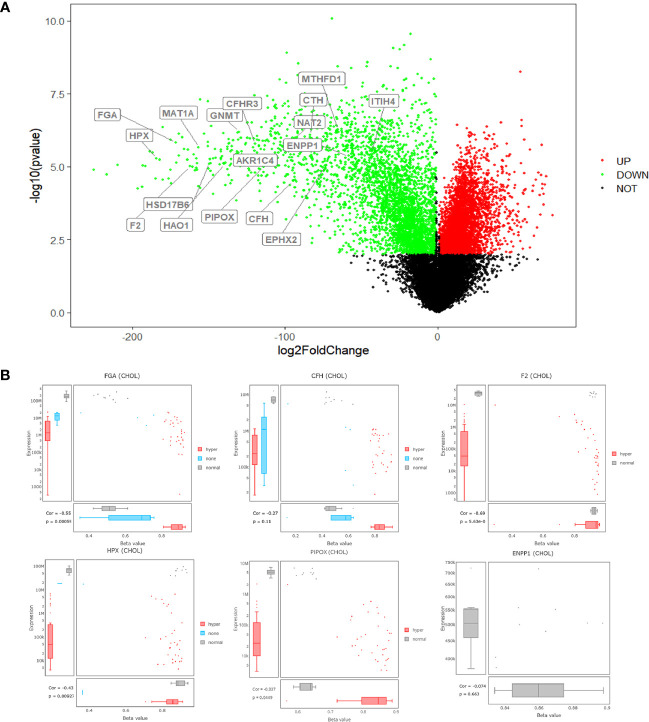
Annotation of core genes. **(A)** volcano plots for DECs in GBC based on the four microarray datasets from GEO. **(B)** the levels of methylation of hub genes between the control and GBC cases from TCGA database.

Seventeen core genes were obtained *via* intersection of the PPI network, methylation-related signaling pathways, and bile-related biological processes, including *FGA*, *F2*, *HAO1*, *CFH*, *PIPOX*, *ITIH4*, *GNMT*, *MAT1A*, *MTHFD1*, *HPX*, *CTH*, *EPHX2*, *HSD17B6*, *AKR1C4*, *CFHR3*, *ENNP1*, and *NAT2*. The 17 core genes were verified in TCGA’s CHOL RNA-seq dataset, and all were significantly expressed. The fold change range of these 17 genes in the TCGA dataset was −184.580~−43.240, and the corrected *P* value range was 1.40E-04~1.24E-03, illustrating that the 17 core genes were all downregulated genes in control *vs* GBC samples, as shown in **Figure 4A**. Then, Spearman correlation coefficients and Spearman *P* value were used to analyze the correlations of the levels of methylation of hub genes between the control and GBC cases. The results showed that the genes *CFH*, *F2*, *FGA*, *HPX*, and *PIPOX* were highly methylated in GBC cases compared with control cases (**Figure 4B**). Next, the RMBase database was used to analyze the RNA methylation sites of core genes (as shown in **Table 3**). The results showed that the genes *FGA*, *F2*, *HAO1*, *CFH*, *ITIH4*, *GNMT*, *MTHFD1*, *HPX*, *CTH*, *HSD17B6*, and *AKR1C4* had RNA methylation modification sites, but *EPHX2*, *MAT1A*, and *PIPOX* had no RNA methylation modification sites. Combining the above results, we came to the conclusion that the genes *CFH*, *F2*, *FGA*, and *HPX* are methylated genes in GBC.

**Table 3:** RNA methylation sites of hub genes form RMBase database.

Gene_Symbol	RNA methylation_Site_Number	Motif_Score	Location
**AKR1C4**	2	226.96~301.91	cds,5’-UTR
**CFH**	38	226.96~ 419.59	cds,5’-UTR,3’-UTR, intron
**CTH**	9	224.83~371.87	cds,5’-UTR,3’-UTR
**F2**	6	274.68~419.59	cds,5’-UTR,3’-UTR, intron
**FGA**	60	224.83~419.59	cds,5’-UTR,3’-UTR, intron
**GNMT**	2	344.64~369.74	3’-UTR
**HAO1**	3	226.96~349.63	cds,5’-UTR
**HPX**	4	274.68~371.87	cds,3’-UTR
**HSD17B6**	8	301.91~ 419.59	cds,5’-UTR, intron
**ITIH4**	45	224.83~ 419.59	cds,5’-UTR,3’-UTR, intron
**MTHFD1**	53	224.83~ 419.59	cds,5’-UTR,3’-UTR, intron

### Survival Analysis

**Figure 5:**
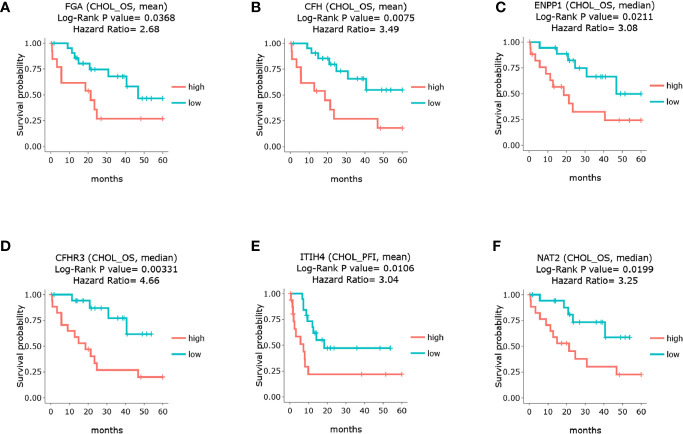
Survival analysis of the core genes in GBC patients through TCGA database. **(A)** survival analysis of FGA. **(B)** survival analysis of CFH. **(C)** survival analysis of ENPP1. **(D)** survival analysis of CFHR3. **(E)** survival analysis of ITIH4. **(F)** survival analysis of NAT2.

**Figure 6:**
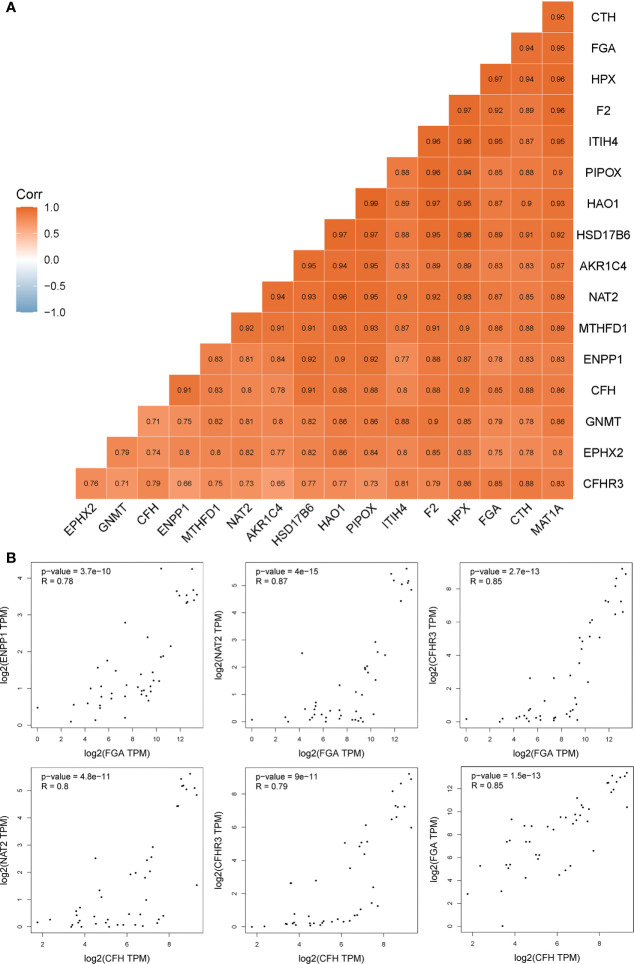
The correlation coefficients among 17 core genes from the TCGA CHOL dataset. **(A)** the details of correlation coefficients by Pearson statistical analysis among 17 core genes. **(B)** the genes positively correlated with *FGA* and *CFH*.

To illustrate whether any core genes affect the overall survival of GBC patients, survival analysis was used to assess prognostic markers in the TCGA database. The results demonstrated that highly expressed *FGA*, *CFH*, *ENPP1*, *CFHR3*, *ITIH4*, and *NAT2* were associated with poor prognosis. Specifically, *FGA* was highly expressed in 14 samples but expressed at low levels in 22 samples. The 5-year overall survival HR was 2.680, and the log-rank *P* value was 0.037 (**Figure 5A**). *CFH* was highly expressed in 14 samples but expressed at low levels in 22 samples, and the 5-year overall survival HR was 3.490, with a log-rank *P* value of 0.007 (**Figure 5B**). *ENPP1* was highly expressed in 18 samples but expressed at low levels in 18 samples, and the 5-year overall survival HR was 3.250, with a log-rank *P* value of 0.020 (**Figure 5C**). *CFHR3* was highly expressed in 18 samples but expressed at low levels in 18 samples, and the 5-year overall survival HR was 4.660, with a log-rank *P* value of 0.003 (**Figure 5D**). *ITIH4* was highly expressed in 18 samples but expressed at low levels in 18 samples; the 5-year overall survival HR was 3.520, and the log-rank *P* value was 0.004 (**Figure 5E**). *NAT2* was highly expressed in 18 samples but expressed at low levels in 18 samples; the 5-year overall survival HR was 3.250, and the log-rank *P* value was 0.020 (**Figure 5F**). Moreover, survival analysis of the other 12 hub genes showed no significant differences (shown in **Supplementary Figure 1**). In addition, *Pearson* statistical analysis was performed to calculate the correlation coefficients among 17 core genes from the TCGA CHOL dataset, and the range of correlation coefficients between hub genes was −0.067~0.950, with a *P* value range of 4.8E-11~0.900 (details shown in **Supplementary Table 2** and **Figure 6A**). The results showed that the FGA gene was positively correlated with *ENPP1*, *NAT2*, and *CFHR3*, while the CFH gene was positively correlated with *FGA*, *NAT2*, and *CFHR3* (as shown in **Figure 6B**). Our results provide evidence for further research on RNA methylation in GBC.

### Coexpression Network and Tumor Microenvironment Prediction

Coexpression network analysis of six genes (*FGA*, *CFH*, *CFHR3*, *NAT2*, *ENPP1*, and *ITIH4*) was carried out through GeneMANIA, and the results are shown in **Supplementary Figure 2A**. A weighted network was constructed with the following weight values: *FGA*, 0.003~0.108; *CFH*, 0.005~0.012; *CFHR3*, 0.003~0.027; *NAT2*, 0.006~0.020; *ENPP1*, 0.004~0.021; and *ITIH4*, 0.003~0.008. These results indicated that *FGA*, *CFH*, *CFHR3*, *NAT2*, *ENPP1*, and *ITIH4* had coexpression patterns. The TISCH database was used to predict the tumor microenvironment of these six genes, and the results revealed that the five genes other than *NAT2* were specifically expressed in cells, as shown in **Supplementary Figure 2A**. *FGA*, *CFH*, and *ITIH4* were expressed mainly in immune cells, malignant cells, and stromal cells, while *CFHR3* and *ENPP1* were expressed mainly in malignant cells and stromal cells. Our results further illustrated that these genes are closely associated and should be further studied.

### Clinical Specimen Verification by Immunohistochemistry

**Figure 7:**
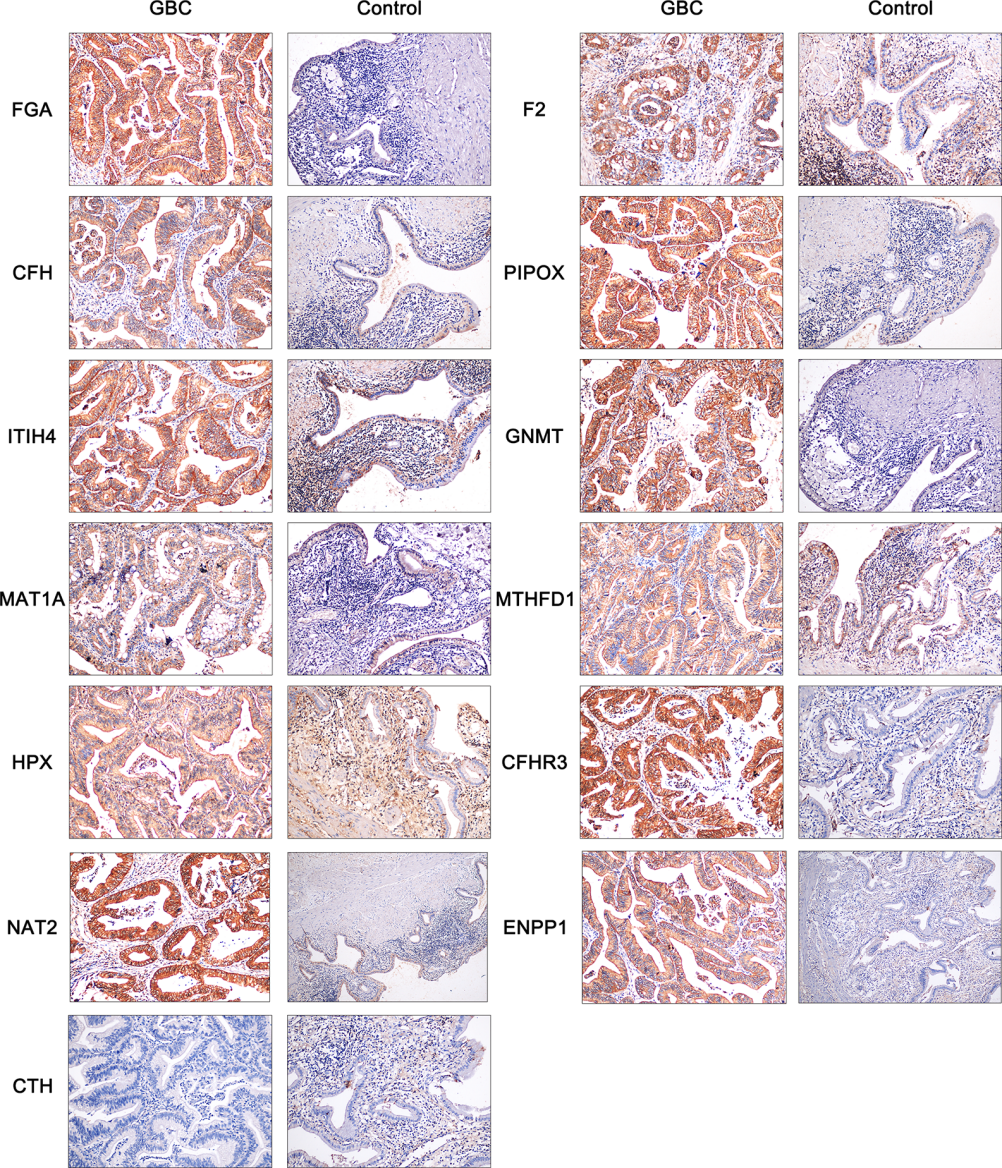
Immunohistochemistry of FGA, F2, CFH, PIPOX, ITIH4, GNMT, MAT1A, MTHFD1, HPX, CTH, CFHR3, ENNP1, and NAT2 in clinical GBC and control specimens.

To further verify the expression of hub genes in clinical samples, immunohistochemistry was used. According to the above findings, we screened 13 of the 17 hub genes for immunohistochemistry, including FGA, F2, CFH, PIPOX, ITIH4, GNMT, MAT1A, MTHFD1, HPX, CTH, CFHR3, ENNP1, and NAT2. As shown in **Figure 7**, the results showed that the expressions of FGA, CFH, PIPOX, GNMT, MAT1A, CFHR3, NAT2, and ENPP1 were higher in GBC tissues than in control tissues, and these proteins were mainly located in the cytoplasm of tumor cells. It is interesting to find that F2, ITIH4, and HPX proteins were mainly expressed in tumor cells of GBC tissues, but these proteins were located mainly in inflammatory cells of control tissues not normal gallbladder epithelium. Besides, MTHFD1 was expressed in both GBC tissues and control tissues, and there was no significant difference between tissue types. In addition, our results showed that CTH was not expressed in either GBC tissues or control tissues.

## Discussion

Although some advances in GBC research have been made, effective methods for the early diagnosis of GBC are still very scarce. In this study, 81 differentially expressed genes were identified from four array datasets from the GEO database through bioinformatics analysis and protein-protein interaction analysis. Among the ranked genes, 17 hub genes (*FGA*, *F2*, *HAO1*, *CFH*, *PIPOX*, *ITIH4*, *GNMT*, *MAT1A*, *MTHFD1*, *HPX*, *CTH*, *EPHX2*, *HSD17B6*, *AKR1C4*, *CFHR3*, *ENNP1*, and *NAT2*) involved in methylation signaling pathways and bile-related biological processes were confirmed. We performed pathway annotation, TCGA verification, posttranslational modification analysis, survival analysis, and IHC of these hub genes to finally obtain six candidates, including *FGA*, *CFH*, *ENPP1*, *ITIH4*, *CFHR3*, and *NAT2*, associated with GBC prognosis. Among these, *FGA* and *CFH* were identified as RNA methylation-related biomarkers in GBC, which may be novel biomarkers to early diagnose and evaluate prognosis for GBC.

The occurrence and progression of GBC is influenced by heredity and the environment, and epigenetic mechanisms, including histone modification (acetylation, methylation, and phosphorylation), have been reported to play important roles in the pathology of GBC (30). RNA methylation, which is one of the most common posttranscriptional modifications of RNAs, has recently been found to participate in tumorigenesis, invasion, metastasis, and drug resistance and could be a new diagnostic biomarker and therapeutic target (5). One study recently illustrated that NOP2/Sun domain family member 2 (*NSUN2*), a nuclear RNA methyltransferase catalyzing 5‐methylcytosine formation, closely interacts with RPL6 to participate in GBC (31). However, research on the role of RNA methylation in gallbladder cancer is still very rare. In our present study, we finally identified 17 hub genes, among which *FGA*, *CFH*, *F2*, *HPX*, and *PIPOX* were highly methylated in GBC tissues. Surprisingly, our bioinformatics results showed that *PIPOX* has no methylation modification site. Next, we used clinical samples for verification and discovered that *FGA*, *CFH*, *F2*, *HPX*, and *PIPOX* were more highly expressed in GBC tissues than in control tissues but that expression was not significantly different. *FGA* encodes the alpha subunit of the coagulation factor fibrinogen, and downregulation of *FGA* seems to be associated with poor prognosis in human lung cancer (32). However, in our study, we found that *FGA* was highly expressed in GBC tissues from patients with a poor prognosis. Thus, it will be interesting to further explore the effect and underlying mechanism of *FGA* in GBC and whether *FGA* could be an indicator of the specific diagnosis of GBC. Complement factor H (*CFH*) was recently found to regulate complement activation in the liver, which is associated with hepatocellular injury (33), but our results demonstrated that there was no significant difference in *CFH* expression between GBC and control tissues. *F2*, known as coagulation factor II, encoding the prothrombin protein, was recently reported to be aberrantly methylated in old Chinese rhesus macaques, which is similar to our findings (34). *HPX* (hemagglutinin) is a plasma acute-phase glycoprotein produced by the liver that binds with high affinity to equimolar heme, and it can counteract cardiac heme toxicity such as that caused by oxidative stress, disruption of cardiac Ca^2+^ homeostasis and contractile dysfunction (35). *PIPOX*, which is a sarcosine-metabolizing enzyme, is highly expressed in HER-2 type cancer (36) but expressed at low levels in prostate cancer (33). We found that PIPOX was more highly expressed in GBC tissues than in control gallbladder tissues but that it has no methylation site. Differences in PIPOX expression in different tumors may be related to sarcosine metabolism, which also needs further study.

Aside from the above five methylation-related genes, other genes involved in bile-related biological processes, including *CTH*, *GNMT*, *HAO1*, *TIH4*, *MAT1A*, and *MTHFD1*, were identified. Abnormalities in bile metabolism, including increased biliary secretion (37) and hyposecretion of biliary bile acids (38), are closely related to gallstones, which are known risk factors for GBC (39). Therefore, we believe that these hub genes may have important clinical significance in the pathogenesis, diagnosis, and treatment of GBC. Increased *CTH* (Cystathionine Gamma-Lyase) expression in patients with advanced prostate cancer is associated with poor survival, and H2S produced by *CTH* promotes the progression and metastasis of prostate cancer through the IL-1β/NF-κB signaling pathway (40). *GNMT* (Glycine N-Methyltransferase) catalyzes the methylation of glycine to form sarcosine, but in this study, we found that it mainly participates in bile metabolism and has little to do with the methylation of hub genes in the development of gallbladder cancer (41). HAO1, encoding the enzyme hydroxyacid oxidase 1, is expressed primarily in the liver and is related to primary hyperoxaluria type 1 (42). *ITIH4*, inter-alpha-trypsin inhibitor heavy chain 4, is an acute response protein that is secreted primarily by the liver and associated with hepatocellular carcinoma (43). It has been found that crosstalk between FOXM1/NF‐κB and MAT1A (methionine adenosyl transferase 1A) may affect tumorigenesis in liver cancer, but little is known about *MAT1A* in GBC (44). *MTHFD1*, methylenetetrahydrofolate dehydrogenase, cyclohydrolase, and formyltetrahydrofolate synthetase 1, has also been reported to be overexpressed in hepatocellular carcinoma and to predict poor survival and recurrence (45).

On account of the results above, *FGA*, *CFH*, *ENPP1*, *ITIH4*, *CFHR3*, and *NAT2* were highly expressed in GBC tissues than control tissues, and associated with GBC prognosis. In addition, *FGA* and *CFH* were identified as RNA methylation-related biomarkers in GBC, which may be novel biomarkers to early diagnose and evaluate prognosis for GBC.

## Conclusion

In conclusion, by utilizing a comprehensive strategy of big data mining and computational biology, we constructed a protein-protein interaction network and ranked genes, ultimately finding that the core genes *FGA*, *F2*, *HAO1*, *CFH*, *PIPOX*, *ITIH4*, *GNMT*, *MAT1A*, *MTHFD1*, *HPX*, *CTH*, *EPHX2*, *HSD17B6*, *AKR1C4*, *CFHR3*, *ENNP1*, and *NAT2* are involved in methylation signaling pathways and bile-related biological processes in GBC. We performed pathway annotation, TCGA verification, posttranslational modification analysis, survival analysis, and IHC on these core genes to finally obtain six candidates, including *FGA*, *CFH*, *ENPP1*, *ITIH4*, *CFHR3* and *NAT2*, associated with prognosis in GBC, and *FGA* and *CFH* were identified as RNA methylation-related biomarkers in GBC. Focusing on development and epigenetic changes such as RNA methylation may be helpful for early diagnosis of GBC, and we have reason to believe that detection of the RNA methylation levels of *FGA* and *CFH* could be used as a potential diagnostic and prognostic evaluation strategy. Moreover, whether the detection of circulating cell-free RNA of *FGA* and *CFH* is effective to diagnose the early stage of GBC will be further studied in our subsequent experiments.

## Data Availability Statement

The original contributions presented in the study are included in the article/**Supplementary Material**. Further inquiries can be directed to the corresponding authors.

## Ethics Statement

The studies involving human participants were reviewed and approved by the ethics committee of Chongqing Medical University. The patients/participants provided their written informed consent to participate in this study. Written informed consent was obtained from the individual(s) for the publication of any potentially identifiable images or data included in this article.

## Author Contributions

CY and NJ conceived and designed this research. ZY, JL, and XL collected and downloaded the relative data. CY and JC analyzed the data and processed them. BZ collected clinical samples and performed histochemistry. NJ wrote the paper. All authors contributed to the article and approved the submitted version.

## Funding

This work was supported by The National Natural Science Foundation of Chongqing of China (No. cstc2020jcyj-bshX0069).

## Conflict of Interest

The authors declare that the research was conducted in the absence of any commercial or financial relationships that could be construed as a potential conflict of interest.

## Abbreviation

GBC, gallbladder carcinoma; DEGs, differentially expressed genes; RRA, RobustRankAggreg; PPI, protein-protein interaction; IHC, immunohistochemistry; TCGA, the Cancer Genome Atlas; GEO, gene expression omnibus; NCBI, national coalition building institute; KEGG, Kyoto encyclopedia of genes and genomes; EBI, European bioinformatics institute; limma, the linear models for microarray data; GO, gene ontology; FDR, false discovery rate; CHOL, cholangio carcinoma; SNP, single nucleotide polymorphisms; OS, overall survival; HR, hazard ratio; TISCH, the Tumor Immune Single-cell Hub database; FGA, fibrinogen alpha chain; F2, coagulation factor II; HAO1, the enzyme hydroxyacid oxidase 1; CFH, complement factor H; PIPOX, pipecolic acid and sarcosine oxidase; ITIH4, inter-alpha-trypsin inhibitor heavy chain 4; GNMT, glycine N-methyltransferase; MAT1A, methionine adenosyl transferase 1A; MTHFD1, methylenetetrahydrofolate dehydrogenase, cyclohydrolase, and formyltetrahydrofolate synthetase 1; HPX, hemagglutinin; CTH, cystathionine gamma-Lyase; EPHX2, epoxide hydrolase 2; HSD17B6, hydroxysteroid 17-Beta Dehydrogenase 6; AKR1C4, Aldo-Keto reductase family 1 member C4; CFHR3, complement factor H related 3; ENNP1, ectonucleotide pyrophosphatase/phosphodiesterase 1; NAT2, N-acetyltransferase 2; NSUN2, NOP2/Sun domain family member 2.
