# Supplementary figures and images for: Mining of RNA Methylation-Related Genes and Elucidation of Their Molecular Biology in Gallbladder Carcinoma
Source: Front Oncol. 2021 Feb 25;11:621806. doi: 10.3389/fonc.2021.621806 (PMC7947712; doi:10.3389/fonc.2021.621806)

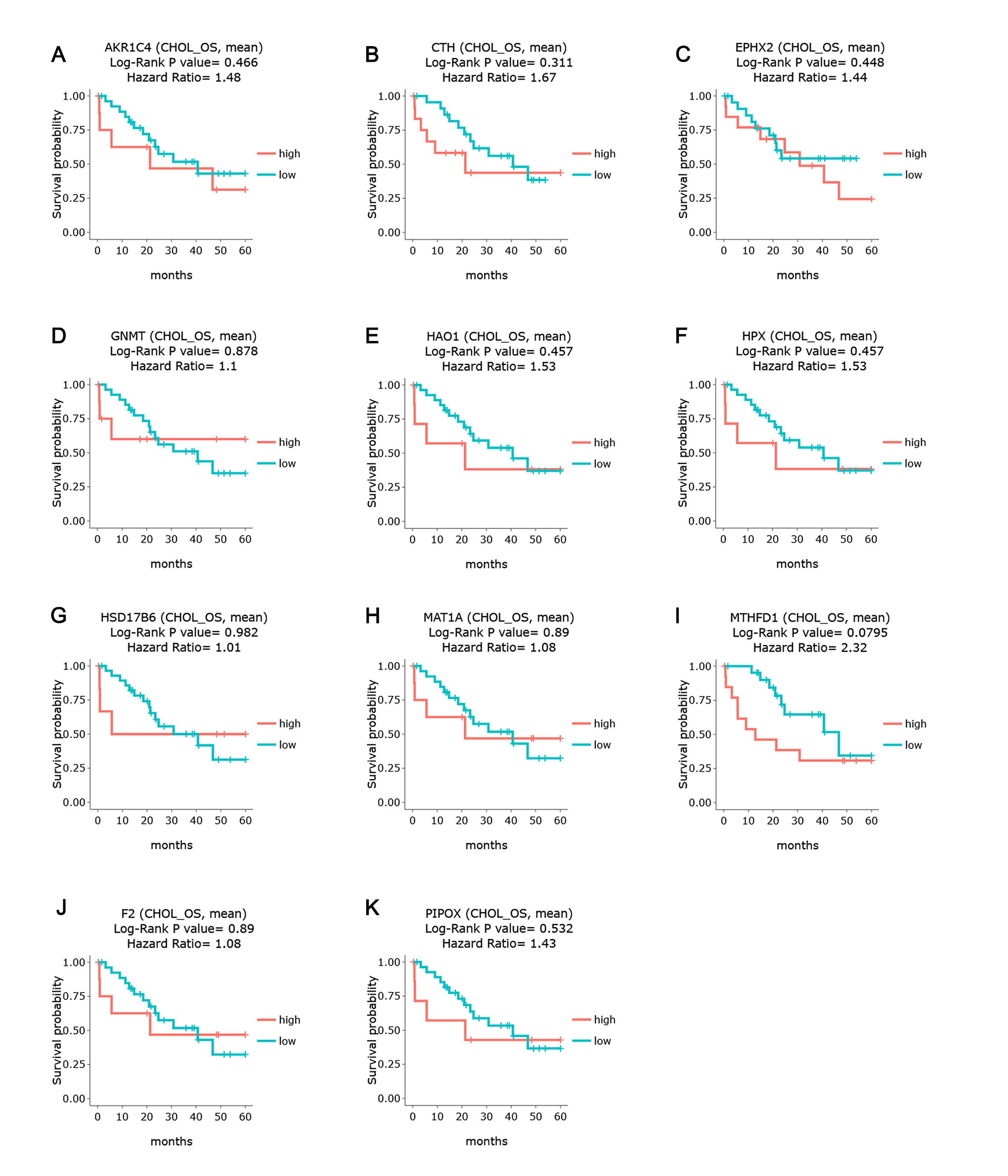

Supplement: Supplementary file 1 [file Image_1.tif]

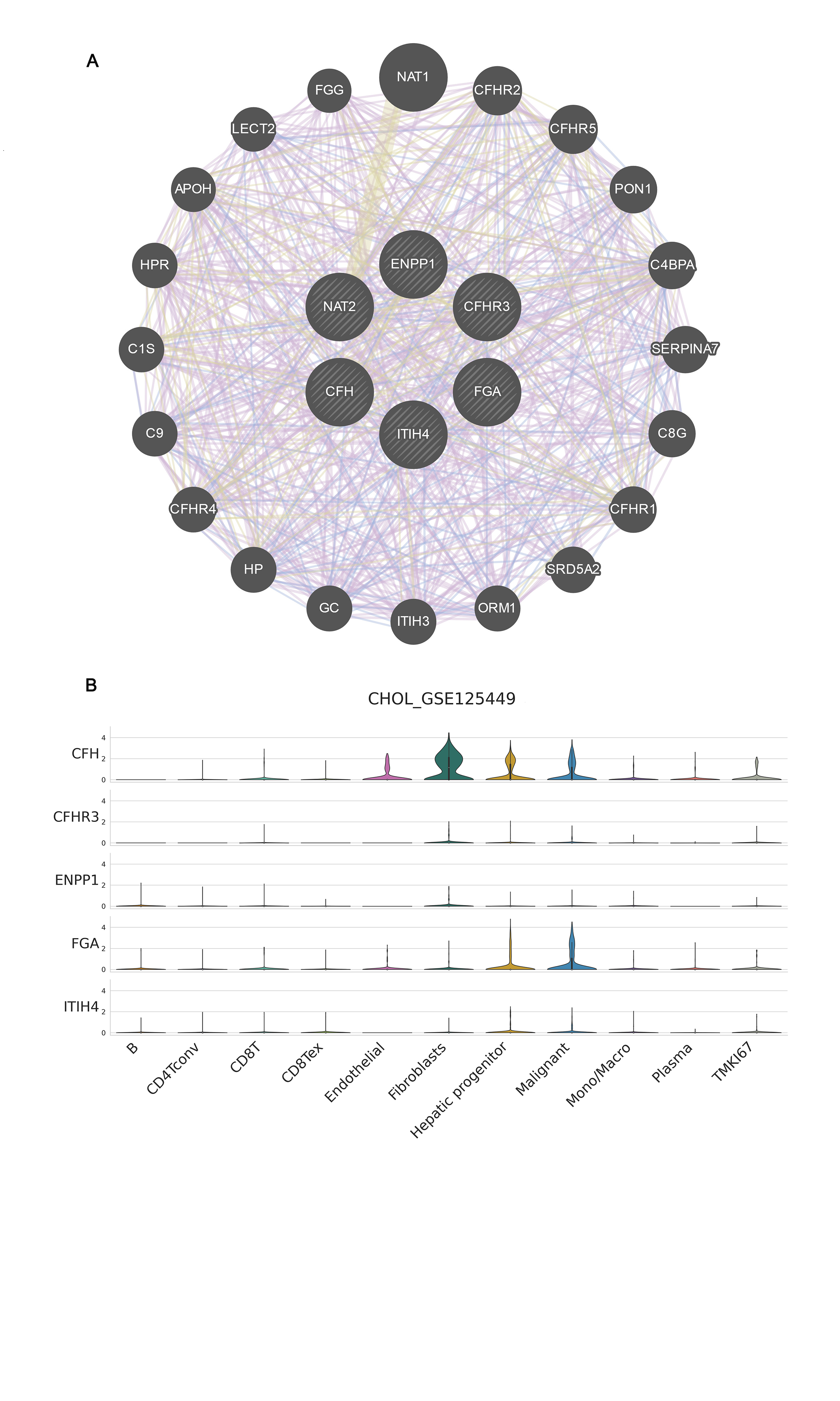

Supplement: Supplementary file 2 [file Image_2.tif]
