# Supplementary material for: Mining of RNA Methylation-Related Genes and Elucidation of Their Molecular Biology in Gallbladder Carcinoma
Source: Front Oncol. 2021 Feb 25;11:621806. doi: 10.3389/fonc.2021.621806 (PMC7947712; doi:10.3389/fonc.2021.621806)
Supplement: Supplementary file 3 [file Table_1.doc]

| **Supplementary Table 1. Identifying differentially expressed genes** | | | | |
| --- | --- | --- | --- | --- |
|  | **GSE31370** | **GSE45001** | **GSE26566** | **GSE76633** |
| A1CF | -2.22 | -4.5 | 0 | -6.23 |
| A2M | -3.75 | -2.51 | 0 | -2.02 |
| AADAC | -3.11 | -4.58 | 0 | -6.21 |
| AAK1 | 1.94 | 0 | 0 | 1.05 |
| ABAT | -2.38 | -4.56 | -2.41 | -4.73 |
| ABLIM1 | 1.48 | 0 | 0 | 0 |
| ABLIM3 | -1.78 | -1.88 | 0 | -2.35 |
| ACAD11 | -1.69 | 0 | -1.65 | -2.84 |
| ACADS | -1.59 | -1.63 | 0 | -3.21 |
| ACAT2 | -1.57 | -1.42 | 0 | -2.17 |
| ACO1 | -1.28 | 0 | 0 | -2.12 |
| ACOT1 | -1.82 | 0 | 0 | -2.11 |
| ACOT2 | -1.41 | 0 | 0 | 0 |
| ACSL1 | -3.67 | -2.62 | -2.79 | -3.59 |
| ACSL5 | -1.95 | 0 | 0 | -1.11 |
| ACSM2B | -2.91 | -7.16 | -3.95 | -8.07 |
| ACSS1 | 1.78 | 0 | 0 | 2.12 |
| ACTG2 | 2.11 | 0 | -3.62 | 3.6 |
| ACTN4 | 1.71 | 0 | 0 | 1.47 |
| ACY3 | -1.47 | 0 | 0 | -2.37 |
| ADGRG7 | -1.99 | 0 | -4.18 | 0 |
| ADH6 | -2.68 | -6.52 | -2.86 | -7.12 |
| ADI1 | -2.43 | -2.37 | 0 | -3.13 |
| ADRA1A | -1.14 | 0 | -2.86 | -6.63 |
| ADRA1B | -1.36 | -2.54 | 0 | -3.74 |
| AFAP1 | 1.27 | 1.42 | 0 | 2.68 |
| AGAP1 | 1.58 | 1.84 | 0 | 1.13 |
| AGAP6 | 1.41 | 0 | 0 | 0 |
| AGFG1 | 1.23 | 1.42 | 0 | 1.06 |
| AGTR1 | -2.87 | -4 | 0 | -5.15 |
| AGXT2 | -2.85 | -5.99 | 0 | -8.21 |
| AHCTF1 | 1.45 | 0 | 0 | 0 |
| AHNAK | -1.21 | 0 | 0 | 1.08 |
| AIG1 | -1.8 | -1.77 | 0 | -2.93 |
| AJUBA | -1.06 | 0 | 0 | 0 |
| AKR1C4 | -3.57 | -7.52 | -3.75 | -8.5 |
| ALAS1 | -1.69 | -1.78 | 0 | -2.95 |
| ALDH1A1 | -2.71 | -2.68 | 0 | -3.37 |
| ALDH1B1 | -2.23 | -1.36 | 0 | -2.31 |
| ALDH2 | -2.56 | -2.44 | 0 | -3.26 |
| ALDH4A1 | -2.31 | -3.29 | -2.47 | -4.49 |
| ALDH5A1 | -2.12 | -2.52 | 0 | -3.16 |
| ALDH6A1 | -2.93 | -3.18 | -1.96 | -4.47 |
| ALG13 | 1.06 | 0 | 0 | 0 |
| AMBP | -3.21 | -6.54 | 0 | -8.9 |
| ANG | -3.38 | -4.26 | -2.38 | -5.64 |
| ANGPTL3 | -4.27 | -8.03 | 0 | -8.52 |
| ANGPTL6 | -1.9 | -2.92 | 0 | -3.43 |
| ANKLE2 | 1.07 | 0 | 0 | 1.5 |
| ANKRD33 | -1.33 | 0 | 0 | 0 |
| ANKRD36B | 2.23 | 0 | 0 | 1.33 |
| ANKRD46 | -1.53 | 0 | 0 | -1.38 |
| ANXA11 | 1.33 | 0 | 0 | 1.1 |
| AP1G2 | 1.22 | 0 | 1.27 | 1.39 |
| APOH | -4.4 | -8.2 | 0 | -9.07 |
| AQP11 | -1.71 | 0 | 0 | -3.34 |
| AQP12A | -2.78 | 0 | -3.36 | 0 |
| AQP3 | -1.18 | -1.57 | 0 | 0 |
| ARID4B | 1.74 | 0 | 0 | 0 |
| ARRDC4 | -1.28 | 0 | 0 | 0 |
| ASB8 | -1.2 | 0 | 0 | 0 |
| ASGR2 | -3.53 | -3.98 | 0 | -6.6 |
| ASS1 | -2.3 | -4.31 | 0 | -4.17 |
| ATF5 | -3.3 | -2.1 | -1.94 | -3.84 |
| ATIC | 1.08 | 1.38 | 0 | 0 |
| ATP11C | -1.26 | 0 | -1.36 | -1.6 |
| ATP6V1E1 | 1.01 | 0 | 0 | 0 |
| BACE1 | -1.02 | 0 | 0 | -1.15 |
| BAIAP2 | -1.05 | 0 | 0 | -2.27 |
| BBOX1 | -3.4 | -3.7 | 0 | -6.36 |
| BCHE | -3.13 | -4.43 | 0 | -5.48 |
| BCKDHB | -1.78 | -1.9 | 0 | -2.06 |
| BHMT2 | -2.84 | -5.25 | 0 | -6.23 |
| BLVRB | -1.62 | 0 | 0 | -1.26 |
| BOK | -1.14 | 0 | 0 | -2.46 |
| BOLA2 | 1.01 | 0 | 0 | 0 |
| BRWD1 | -1.05 | 0 | 2.52 | 0 |
| BST2 | -2.16 | 0 | 0 | 0 |
| BTBD16 | -1.05 | 0 | 0 | -2.61 |
| BTD | -1.28 | 0 | 0 | -2.38 |
| BUD31 | 1.03 | 0 | 0 | 0 |
| C16orf70 | -1.01 | 0 | 0 | -1.44 |
| C1orf106 | 2.57 | 0 | 2.18 | 4.13 |
| C1orf198 | 1.13 | 0 | 0 | 1.37 |
| C1orf53 | -1.01 | 0 | 0 | -1.68 |
| C2orf68 | 1.04 | 0 | 0 | 0 |
| C3 | -1.03 | -2.48 | 0 | -3.9 |
| C3orf52 | 1.27 | 0 | 1.63 | 3.86 |
| C4A | -1.06 | 0 | 0 | -4.21 |
| C4BPA | -4.19 | -4.69 | 0 | -7.13 |
| C4BPB | -3.8 | -4.53 | 0 | -5.28 |
| C6 | -4.53 | -5.34 | -3.57 | -6.82 |
| C6orf48 | 1.4 | 0 | 0 | 1.52 |
| C8A | -3.83 | -7.18 | -4.1 | -9.01 |
| C8B | -3.89 | -6.91 | -3.94 | -9.46 |
| C9 | -3.79 | -7.9 | -4.27 | -8.76 |
| CAMK2B | -1.24 | 0 | 0 | -3.98 |
| CAPNS1 | 1.35 | 0 | 0 | 1.03 |
| CASP3 | -1.46 | 0 | 0 | 0 |
| CAT | -3.15 | -2.66 | 0 | -3.38 |
| CBX3 | 1.28 | 0 | 0 | 1.12 |
| CBX5 | 1.6 | 0 | 0 | 0 |
| CCDC130 | 1.04 | 0 | 0 | 0 |
| CCDC137 | 1.23 | 0 | 0 | 1.32 |
| CCDC25 | -1.65 | 0 | 0 | -1.12 |
| CCNH | -1.2 | 0 | 0 | 0 |
| CCT6A | 1.1 | 0 | 0 | 1.2 |
| CD160 | -1.06 | 0 | 0 | -2.4 |
| CD24 | 3.11 | 0 | 0 | 3.08 |
| CD36 | -1.43 | -2.95 | -2.57 | -2.58 |
| CD3D | -1.69 | 0 | 0 | 1.53 |
| CD4 | -1.43 | 0 | 0 | 0 |
| CD5L | -1.14 | -5.42 | 0 | -8.02 |
| CDH23 | -1.05 | -1.45 | -2.6 | -2.71 |
| CDHR5 | -1.5 | -4.75 | 0 | -4.81 |
| CDK11A | 1.64 | 0 | 0 | 0 |
| CDKN1B | -1.34 | 0 | 0 | 0 |
| CEACAM1 | -1.9 | 0 | 0 | -1.63 |
| CEP95 | 1.09 | 0 | 0 | 0 |
| CEPT1 | -1.06 | 0 | 0 | 0 |
| CES2 | -1.65 | -1.67 | 0 | -2.04 |
| CFH | -2.32 | -3.07 | -1.81 | -3.77 |
| CFHR1 | -3.93 | -7.39 | -4.51 | -9.59 |
| CFHR3 | -2.66 | -2.92 | -4.51 | -3.91 |
| CFP | -1.12 | -2.92 | 0 | -3.69 |
| CHPT1 | -2.11 | -1.04 | 0 | -1.68 |
| CIDEB | -2.57 | -2.65 | -2.81 | -4.18 |
| CITED2 | -1.23 | 0 | 0 | 0 |
| CKMT1A | 1.19 | 0 | 3.3 | 6.74 |
| CLEC1B | -3.32 | -6.27 | 0 | -8.02 |
| CLEC4G | -2.77 | -5.01 | 0 | -6.37 |
| CLN8 | 1.24 | 0 | 0 | -1.17 |
| CLRN3 | -2.64 | -3.22 | 0 | -3.42 |
| CLSTN1 | 1.58 | 0 | 0 | 2.16 |
| CLYBL | -1.9 | -1.71 | 0 | -2.93 |
| CMPK1 | -1.16 | 0 | 0 | 0 |
| CNBP | -1.74 | 0 | 0 | 0 |
| CNDP1 | -1.71 | 0 | 0 | -7.7 |
| CNGA1 | -1.67 | 0 | 0 | -3.8 |
| COG2 | 1.04 | 0 | 0 | 0 |
| COL7A1 | 1.53 | 0 | 0 | 2.82 |
| CP | -2.97 | -3.9 | 0 | -5.67 |
| CPB2 | -3.65 | -5.87 | 0 | -8.62 |
| CPOX | -1.11 | 0 | 0 | 0 |
| CPT2 | -1.48 | -1.51 | 0 | -2.36 |
| CRCP | 1.05 | 0 | 0 | 0 |
| CREB3L3 | -3.02 | -5.05 | 0 | -6.73 |
| CRHBP | -2.46 | -4.13 | 0 | -6.3 |
| CRYL1 | -2.52 | -2.44 | 0 | -2.58 |
| CSNK1E | 1.51 | 0 | 0 | 1.82 |
| CSTB | 1.17 | 0 | 0 | 1.26 |
| CTDSPL | 1.23 | 0 | 0 | 0 |
| CTH | -1.45 | -3.43 | -2.75 | -5.02 |
| CTNNA1 | 1.19 | 0 | 0 | 1.16 |
| CTSO | -1.56 | 0 | 0 | 0 |
| CTTN | 1.25 | 1.12 | 0 | 1.03 |
| CUL4A | -1.03 | 0 | 0 | 0 |
| CUX1 | 1.2 | 0 | 1.02 | 1.06 |
| CUX2 | -2.4 | -4.04 | -3.53 | -6.91 |
| CXCL10 | -2.76 | 0 | 0 | 0 |
| CXCL2 | -2.26 | 0 | -2.43 | -2.8 |
| CXCL9 | -2.64 | 0 | 0 | 2.91 |
| CYBB | -1.11 | 0 | 0 | 0 |
| CYP26A1 | -2.05 | -3.53 | 0 | -3.81 |
| CYP2A7 | -2.57 | -9.08 | 0 | -7.89 |
| CYP2B6 | -1.26 | -6.81 | 0 | -6.63 |
| CYP2B7P | -1.22 | 0 | 0 | 0 |
| CYP39A1 | -1.71 | -4 | -2.06 | -4.26 |
| CYP4A22 | -1.59 | -5.07 | 0 | -7.77 |
| CYP4F2 | -2.69 | -6.53 | 0 | -7.28 |
| CYP51A1 | -1.57 | 0 | 0 | 0 |
| CYTH2 | 1.88 | 0 | 0 | 0 |
| DAAM1 | -1.06 | 0 | 0 | 0 |
| DBH | -2.88 | 0 | 0 | -5.35 |
| DBI | -1.64 | 0 | 0 | -1.07 |
| DBN1 | 2.28 | 1.88 | 5 | 2.39 |
| DBNDD2 | 2.06 | 0 | 0 | 2.23 |
| DCAF7 | 1.04 | 0 | 0 | 0 |
| DDR1 | 2.75 | 2.12 | 1.89 | 2.64 |
| DDT | -1.41 | -1.41 | 0 | -2.75 |
| DDX17 | 1.98 | 0 | 0 | 0 |
| DEPDC7 | -1.58 | -3.31 | -2.48 | -3.28 |
| DEXI | -1.29 | 0 | 0 | 0 |
| DGAT2 | -1.67 | 0 | -2 | -3.73 |
| DGCR6 | -1.42 | 0 | 0 | 0 |
| DGCR6L | -1.2 | 0 | 0 | -1.3 |
| DHRS3 | -1 | -1.19 | 0 | -1.74 |
| DHRS4 | -1.67 | -1.12 | 0 | -1.79 |
| DHRS4L2 | -1.93 | 0 | 0 | -1.66 |
| DICER1 | -1.45 | 0 | 0 | 0 |
| DIO1 | -1.75 | -5.05 | 0 | -6.87 |
| DKFZp779M0652 | -1.18 | -1.98 | 0 | 0 |
| DMGDH | -1.25 | -6.6 | 0 | -7.4 |
| DNAAF5 | 1.44 | 0 | 0 | 0 |
| DNAJC25 | -1.64 | 0 | -1.03 | -2.1 |
| DNASE1L3 | -3.47 | 0 | -4.59 | -5.41 |
| DNM1L | 1.23 | 1.33 | 0 | 1.01 |
| DPYD | -1.23 | 0 | 0 | -1.22 |
| DTX3 | 1.19 | 0 | 0 | 0 |
| ECHDC1 | -1.11 | 0 | 0 | 0 |
| ECI2 | -2.05 | -1.54 | 0 | -2.68 |
| EI24 | -1.4 | 0 | 0 | 0 |
| EIF2B3 | -1.34 | 0 | 0 | 0 |
| EIF3K | 1.09 | 0 | 0 | 0 |
| EIF3M | -1.13 | 0 | 0 | 1.03 |
| EIF4G3 | 1.02 | 0 | 0 | 0 |
| EIF5 | -1.27 | -1.4 | 0 | -1.86 |
| ELL2 | -1.57 | -1.19 | 0 | -1.33 |
| ELOVL5 | -1.01 | 0 | 0 | 0 |
| ELOVL6 | -1.9 | -1.3 | 0 | -2.4 |
| ENPEP | -1.39 | 0 | 0 | -3.41 |
| ENPP1 | -1.74 | -1.76 | -2.1 | -3.44 |
| ENPP7 | -1.2 | 0 | 0 | -3.24 |
| ENTPD5 | -1.45 | -2.01 | 0 | -3.51 |
| EOMES | -1.17 | 0 | 0 | 0 |
| EPCAM | 3.4 | 0 | 2.24 | 5.13 |
| EPHX2 | -2.31 | -4.37 | -2.19 | -4.16 |
| EPS8L1 | 1.76 | 0 | 0 | 3.46 |
| ERGIC1 | -1.12 | 0 | 0 | 0 |
| ERLIN1 | -1.28 | 0 | 0 | -1.45 |
| ERMAP | -1.15 | 0 | 0 | 0 |
| ESM1 | 1.05 | 0 | 0 | 1.75 |
| ESPN | -2.06 | 0 | 0 | -3.19 |
| ESR1 | -1.54 | 0 | 0 | -3.51 |
| ETFDH | -1.58 | -1.77 | -1.43 | -2.84 |
| ETNPPL | -2.29 | 0 | -4.01 | 0 |
| EVC2 | -2.58 | 0 | -3.68 | 0 |
| EXOC3 | 1.14 | 0 | 0 | 0 |
| F11 | -1.91 | -6.22 | -3.03 | -7.99 |
| F2 | -2.81 | -6.84 | -4.18 | -9.43 |
| FAH | -1.91 | -1.69 | -1.8 | -3.55 |
| FAHD2A | -1.26 | 0 | 0 | -2.39 |
| FAM13A | -1.34 | 0 | 0 | -1.52 |
| FAM162A | -1.83 | 0 | 0 | 0 |
| FAM180A | -1.04 | 0 | 0 | -1.25 |
| FAM210B | -1.21 | 0 | 0 | 0 |
| FAM214A | -1.11 | 0 | 0 | 0 |
| FAM21A | 1.2 | 0 | 0 | 0 |
| FAM83H | 1.61 | 0 | 2.26 | 0 |
| FAM98A | -1.14 | 0 | 0 | 0 |
| FAM99B | -1.13 | -6.11 | 0 | 0 |
| FAP | 1.55 | 3.59 | 2.65 | 5.65 |
| FBRS | 1.27 | 0 | 0 | 0 |
| FBXW7 | 1.31 | 0 | 0 | 0 |
| FCN2 | -2.3 | -6.48 | -3.49 | -7.71 |
| FCN3 | -3.41 | -2.75 | -3.11 | -5.36 |
| FGA | -2.8 | -6.49 | -4.15 | -9.53 |
| FGB | -3.81 | -6.05 | 0 | -8.6 |
| FGD2 | -1.06 | 0 | 0 | 0 |
| FGG | -4.24 | -5.17 | 0 | -8.09 |
| FGGY | -2.07 | -2.38 | 0 | -3.57 |
| FGL1 | -3.15 | -5.63 | 0 | -6.78 |
| FITM1 | -1.26 | 0 | -3.17 | -3.14 |
| FKBP3 | -1.16 | 0 | 0 | 0 |
| FMO4 | -1.63 | -1.64 | 0 | -3.08 |
| FNDC3A | -1.38 | 0 | 0 | 0 |
| FNDC4 | -1.95 | 0 | 0 | -3.02 |
| FNIP2 | -1.19 | 0 | 0 | -1.21 |
| FOLR2 | -1.77 | 0 | 0 | 0 |
| FOXA3 | -2.69 | 0 | 0 | -2.11 |
| FRG1 | -1.32 | 0 | 0 | 0 |
| FTH1P3 | -1.56 | 0 | 0 | 0 |
| FUOM | -2.15 | 0 | 0 | 0 |
| FYTTD1 | -1.01 | 0 | 0 | 0 |
| G3BP1 | -1.44 | 0 | 0 | 0 |
| GAB2 | 1.07 | 0 | 0 | 0 |
| GALT | -1.2 | -1.61 | 0 | -2.26 |
| GARS | 1.36 | 0 | 1.05 | 1.4 |
| GAS2 | -2.21 | -3.59 | 0 | -4.71 |
| GBA3 | -2.93 | 0 | 0 | -5.45 |
| GBE1 | -1.46 | 0 | 0 | -1.88 |
| GBP1 | -2.15 | 0 | 0 | 0 |
| GC | -3.03 | -5.41 | 0 | -8.78 |
| GCC2 | 1.05 | 0 | 0 | 0 |
| GCH1 | -2.09 | -1.7 | 0 | -2.07 |
| GCHFR | -1.7 | 0 | 0 | -2.84 |
| GCKR | -1.91 | 0 | 0 | -5.7 |
| GCLC | -1.88 | -1.46 | 0 | -1.84 |
| GGCX | -1.42 | 0 | 0 | -2.66 |
| GGH | -1.86 | 0 | 0 | -2.75 |
| GGPS1 | 1.12 | 0 | 0 | 0 |
| GHR | -3.64 | -4.03 | -3.3 | -5.92 |
| GIPC1 | 1.25 | 1.29 | 1.31 | 1.16 |
| GJB2 | -1.59 | 0 | 0 | 0 |
| GLIS3 | 1.34 | 0 | 0 | 0 |
| GLOD5 | -1.12 | 0 | 0 | -3.38 |
| GLS2 | -2.34 | -5.91 | -3.06 | -6.9 |
| GLYAT | -2.16 | -8.46 | -3.78 | -9.21 |
| GLYATL1 | -1.95 | -7.02 | -2.83 | -8.16 |
| GMCL1 | 1.59 | 0 | 0 | 0 |
| GNE | -1.98 | -1.69 | 0 | -2.27 |
| GNMT | -2.86 | -4.32 | -3.25 | -6.8 |
| GOLGA8A | 1.72 | 0 | 0 | 1.05 |
| GOLGA8B | 1.63 | 0 | 0 | 0 |
| GOLIM4 | -1.41 | 0 | 0 | 0 |
| GPC6 | -1.61 | 0 | 0 | -1.43 |
| GPHN | -1.24 | 0 | 0 | -1.59 |
| GPSM2 | 1.38 | 2.93 | 0 | 3.73 |
| GPT | -2.92 | -4.01 | 0 | -5.03 |
| GRAMD1A | 1.74 | 1.86 | 0 | 1.17 |
| GRHPR | -1.88 | -1.73 | 0 | -3.07 |
| GSTZ1 | -2.35 | -1.63 | 0 | -2.55 |
| GTF2I | 1.06 | 0 | 0 | 0 |
| GTF2IRD1 | 1.06 | 0 | 2.13 | 1.68 |
| GUSBP2 | 1.66 | 0 | 0 | 0 |
| GYS2 | -1.11 | -7.37 | 0 | -8.4 |
| H2AFY | 1.01 | 0 | 0 | 1.05 |
| H3F3A | 1.01 | 0 | 0 | 0 |
| HAAO | -1.08 | -3.89 | 0 | -4.34 |
| HACD2 | -1.13 | 0 | 0 | 0 |
| HAL | -2.65 | -5.39 | 0 | -7.5 |
| HAO1 | -3.52 | -6.14 | -3.69 | -7.96 |
| HAO2 | -2.96 | -7.76 | -4.05 | -9.26 |
| HBA1 | -2.73 | 0 | 0 | 0 |
| HBA2 | -3.06 | -4.84 | 0 | -2 |
| HBB | -3.17 | -4.6 | 0 | -2.39 |
| HCN4 | -1.3 | 0 | 0 | 1.95 |
| HDAC6 | -1.13 | -1.24 | 0 | -1.6 |
| HDHD2 | -1.21 | 0 | 0 | 0 |
| HEPACAM | -2.62 | -2.62 | 0 | -4.22 |
| HEXIM1 | -1.01 | 0 | 0 | 0 |
| HGD | -1.25 | -3.63 | 0 | -6.74 |
| HGS | 1.28 | 0 | 0 | 1.48 |
| HIF1A | -1.09 | 0 | 0 | 1.08 |
| HIGD1A | -1.84 | 0 | 0 | -1.73 |
| HIST1H1C | -1.47 | 0 | 0 | 0 |
| HM13 | 1.05 | 0 | 0 | 0 |
| HMGB1 | -1.04 | 0 | 0 | 0 |
| HMGCL | -1.79 | 0 | 0 | -2.8 |
| HMGN4 | 1.38 | 0 | 0 | 1.74 |
| HNRNPC | -1.07 | 0 | 0 | 0 |
| HNRNPR | 1.1 | 0 | 0 | 0 |
| HNRNPUL1 | 1.15 | 0 | 0 | 0 |
| HPX | -4.86 | -7.38 | -5.11 | -8.76 |
| HRG | -2 | -9.67 | 0 | -9 |
| HSD17B10 | -1.18 | 0 | 0 | -1.28 |
| HSD17B13 | -3.21 | -6.25 | 0 | -8.14 |
| HSD17B6 | -4.51 | -5.62 | -4.84 | -6.82 |
| HSD17B8 | -1.63 | -1.27 | 0 | -1.92 |
| HSDL2 | -1.55 | -1.52 | 0 | -2.46 |
| HSPE1 | -2.43 | 0 | 0 | 0 |
| HYAL1 | -2.17 | -2.18 | 0 | -4.48 |
| ICA1 | 1.48 | 0 | 0 | 0 |
| IDI1 | -1.23 | 0 | 0 | -1.12 |
| IDUA | 1.5 | 0 | 0 | 0 |
| IFNAR1 | -1.03 | 0 | 0 | 0 |
| IGF1 | -2.42 | -2.28 | 0 | 0 |
| IGF2BP2 | 1.75 | 0 | 0 | 2.28 |
| IGFALS | -3.12 | -5.06 | 0 | -7.05 |
| IGFBP1 | -2.45 | -3.45 | 0 | -7.02 |
| IGSF6 | -1.24 | 0 | 0 | 0 |
| IL10RA | -1.15 | 0 | 0 | 0 |
| IL13RA2 | -1.54 | 0 | 0 | -3.75 |
| IL1R1 | -1.13 | 0 | 0 | 0 |
| IL1RAP | -1.36 | -1.92 | 0 | -3.64 |
| IL2RB | -1.15 | 0 | 0 | 0 |
| INHBE | -3.34 | 0 | 0 | -3.67 |
| IRF8 | -1.45 | 0 | 0 | 0 |
| ISOC1 | -1.71 | -1.35 | 0 | -1.9 |
| ISOC2 | -1.3 | 0 | 0 | -1.49 |
| ITIH4 | -2.27 | -5.69 | -2.45 | -5.92 |
| ITPR3 | 3.03 | 0 | 0 | 4.34 |
| ITPRIPL2 | 1.86 | 0 | 0 | 1.35 |
| JUP | 1.21 | 0 | 1.81 | 0 |
| KAZN | -1.03 | 0 | 0 | 0 |
| KCNA6 | -1.34 | 0 | 0 | 0 |
| KDM8 | -1.34 | 0 | -3.18 | 0 |
| KHK | -2.74 | -4.68 | -2.58 | -4.99 |
| KIAA0922 | -1.07 | 0 | 0 | -1.46 |
| KIAA1161 | -1.04 | 0 | 0 | -2.3 |
| KIFC2 | 1.51 | 0 | 0 | 0 |
| KLF13 | 1.18 | 0 | 0 | 0 |
| KLHDC2 | -1.05 | 0 | 0 | 0 |
| KLHL2 | -1.13 | 0 | 0 | 0 |
| KLKB1 | -2.73 | 0 | -3.5 | -5.78 |
| KRT19 | 3.73 | 0 | 3.08 | 5.99 |
| LAD1 | 2.09 | 0 | 1.98 | 0 |
| LAMA3 | -1.11 | 0 | 0 | 4.4 |
| LAMA5 | 1.7 | 0 | 0 | 1.42 |
| LAMB3 | 1.55 | 2.92 | 3.95 | 3.79 |
| LAMC1 | 1.48 | 1.33 | 0 | 1.89 |
| LAMC2 | 2.61 | 3.27 | 3.88 | 5.56 |
| LARP4 | -1.82 | 0 | 0 | -1.29 |
| LARP4B | 1.2 | 0 | 0 | 0 |
| LBR | 1.41 | 0 | 0 | 0 |
| LCAT | -2.95 | -2.4 | -2.67 | -3.81 |
| LDOC1L | 1.1 | 0 | 0 | 1.6 |
| LEAP2 | -1.81 | -4.34 | 0 | -5.22 |
| LHPP | -1.85 | -1.5 | 0 | -1.59 |
| LHX2 | -1.41 | 0 | 0 | -2.34 |
| LIFR | -1.13 | -2.75 | -2.71 | -2.8 |
| LILRB5 | -1.31 | 0 | 0 | -2.23 |
| LIMS1 | 1.1 | 0 | 0 | 1.53 |
| LINC01348 | -1.31 | 0 | 0 | 0 |
| LLGL2 | 1.28 | 0 | 0 | 0 |
| LOC645166 | 1.63 | 0 | 0 | 0 |
| LOC729970 | -1.12 | 0 | 0 | 0 |
| LPA | -3.11 | -2.72 | 0 | -4.57 |
| LPAR2 | 1.29 | 0 | 1.34 | 2.01 |
| LRCOL1 | -1.43 | 0 | 0 | 0 |
| LRG1 | -2.72 | -2.97 | -2.68 | -5.28 |
| LRIG1 | -1.49 | 0 | -1.45 | 0 |
| LRRC1 | 2.06 | 0 | 1.86 | 4.01 |
| LRRC28 | -1.31 | 0 | 0 | -1.32 |
| MAN1A1 | -1.16 | -1.5 | 0 | -1.5 |
| MAOB | -2.04 | 0 | 0 | -3.78 |
| MAP4K4 | 1.02 | 0 | 0 | 0 |
| MAPK13 | 2.6 | 1.69 | 1.88 | 2.76 |
| MAPK8IP3 | 1.31 | 0 | 0 | 0 |
| MARCO | -2.08 | 0 | -2.9 | -4.45 |
| MARVELD3 | 1.03 | 0 | 2.44 | 0 |
| MASP1 | -1.94 | -5.63 | 0 | -5.97 |
| MAST4 | -1.52 | 0 | 0 | -2.13 |
| MAT1A | -3.42 | -7.68 | -3.91 | -8.42 |
| MATR3 | 1.08 | 0 | 0 | 0 |
| MBL2 | -3.76 | -6.4 | 0 | -7.62 |
| MBNL2 | -1.87 | 0 | 1.82 | 0 |
| MBNL3 | -1.94 | -1.76 | 0 | -1.49 |
| MCTS1 | -1.38 | 0 | 0 | 0 |
| MDK | 2.66 | 0 | 2.85 | 3.4 |
| ME3 | 1.44 | 0 | 0 | 1.27 |
| MEGF9 | -1.12 | 0 | 0 | -1.59 |
| METTL7A | -3 | -3.17 | 0 | -3.51 |
| METTL7B | -1.95 | -2.34 | 0 | -3.24 |
| MFF | 1.07 | 0 | 0 | 0 |
| MFSD10 | 1.98 | 0 | 0 | 1.55 |
| MFSD3 | -1.08 | 0 | 0 | 0 |
| MFSD6 | 1.82 | 0 | 1.18 | 2.79 |
| MIR99AHG | -1.62 | 0 | -1.9 | 0 |
| MMACHC | -1.51 | 0 | 0 | -2.12 |
| MMP11 | 2.3 | 4.91 | 6.11 | 2.97 |
| MPP1 | -1.21 | 0 | 0 | 0 |
| MRPL19 | -1.65 | 0 | 0 | 0 |
| MRPS21 | 1.3 | 0 | 0 | 0 |
| MRPS28 | -1.48 | 0 | 0 | 0 |
| MSMO1 | -2.3 | 0 | 0 | -2.89 |
| MT1E | -1.21 | -2.68 | -3.76 | -3.1 |
| MTA3 | 1.2 | 0 | 1.1 | 1.05 |
| MTHFD1 | -2.04 | -1.51 | -1.32 | -2.28 |
| MTMR3 | -1.13 | 0 | 2.3 | 0 |
| MTPAP | 1.09 | 0 | 0 | 0 |
| MTTP | -3.68 | -7.59 | -3.74 | -7.1 |
| MVK | -1.19 | -1.11 | 0 | -2.13 |
| MVP | 1.23 | 0 | 0 | 1.63 |
| MYO1B | -1.76 | 0 | 0 | -2.49 |
| MYO7A | -1.08 | 0 | 0 | -1.36 |
| N4BP2L1 | -1.81 | -1.98 | 0 | -2.27 |
| NAAA | -1.07 | 0 | 0 | -1.11 |
| NADK2 | -1.97 | 0 | 0 | 0 |
| NAT2 | -3.07 | -2.86 | -4.31 | -5.21 |
| NAT8B | -2.15 | -3.24 | 0 | -5.16 |
| NBPF10 | 1.71 | 0 | 0 | 0 |
| NBPF11 | 1.63 | 0 | 0 | -1.19 |
| NBPF14 | 1.15 | 0 | 0 | 0 |
| NBPF9 | -2.01 | 0 | 0 | 0 |
| NCOR2 | 1.23 | 0 | 0 | 1.27 |
| NDRG3 | 1.72 | 0 | 0 | 1.27 |
| NDUFB9 | -1.39 | 0 | 0 | 0 |
| NECAB2 | -1.35 | 0 | 0 | -4.25 |
| NFAT5 | 1.62 | 0 | 0 | 0 |
| NFATC2IP | 1.15 | 0 | 0 | 0 |
| NFIA | -1.88 | -1.41 | 0 | -1.39 |
| NINJ2 | -1.63 | 0 | 0 | 0 |
| NKX3-1 | -1.08 | 0 | 0 | 0 |
| NMD3 | -1.28 | 0 | 0 | 0 |
| NNT | -1.47 | 0 | 0 | -2.37 |
| NOC3L | -1.18 | 0 | 0 | 0 |
| NOL7 | 1.27 | 0 | 0 | 0 |
| NPC1 | 1.37 | 0 | 0 | 0 |
| NPIPB3 | 1.94 | 0 | 0 | 0 |
| NPIPB5 | 1.78 | 0 | 0 | 0 |
| NR1H3 | -1.42 | 0 | 0 | -1.13 |
| NR1H4 | -1.88 | -2.32 | 0 | -4.82 |
| NR2C2 | 1.29 | 0 | 0 | 0 |
| NSUN6 | -1.52 | -1.67 | 0 | -2.1 |
| NT5DC2 | 1.37 | 0 | 0 | 2.82 |
| NT5E | -1.28 | 0 | 0 | 0 |
| NTHL1 | -1.37 | 0 | 0 | 0 |
| NUDT7 | -1.68 | -2.29 | 0 | -3.08 |
| NUMA1 | 1.16 | 0 | 0 | 0 |
| OBP2B | -1.07 | 0 | 0 | 0 |
| OCEL1 | -1.17 | 0 | 0 | -1.52 |
| OIT3 | -1.82 | -4.46 | 0 | -6.53 |
| ORMDL3 | -1.24 | -1.38 | 0 | -1.53 |
| OSBPL11 | -1.05 | 0 | 0 | 0 |
| OSBPL3 | 1.21 | 1.77 | 2.49 | 3.28 |
| OSBPL8 | -1.18 | 0 | 0 | 0 |
| OSTC | -1.7 | 0 | 0 | 0 |
| OTC | -3.91 | -7.05 | 0 | -8.04 |
| P4HA1 | -2.04 | 0 | 0 | 0 |
| PABPC1 | 1.29 | 0 | 0 | 0 |
| PAH | -2.28 | -6.09 | 0 | -9.32 |
| PALM2 | -1.58 | 0 | 0 | -2.49 |
| PAOX | -1.51 | 0 | 0 | -1.54 |
| PAPSS1 | 1.57 | 0 | 0 | 1.59 |
| PAPSS2 | -1.7 | 0 | 0 | -1.79 |
| PAQR9 | -1.48 | -3.6 | 0 | -5.81 |
| PARP9 | -1.35 | 0 | 0 | 0 |
| PBLD | -1.99 | -1.94 | 0 | -2.87 |
| PC | -2.27 | -1.79 | 0 | -2.89 |
| PCCB | -1.18 | 0 | 0 | -2.04 |
| PCK1 | -2.13 | -7.59 | -4.2 | -7.37 |
| PCK2 | -1.11 | -3.03 | 0 | -4.07 |
| PDCD7 | 1.02 | 0 | 0 | 0 |
| PDGFB | 1 | 0 | 0 | 1.29 |
| PDSS2 | -1.05 | 0 | 0 | 0 |
| PDXP | -2.01 | 0 | -1.35 | -1.79 |
| PEX11A | -1.05 | 0 | 0 | -2.19 |
| PEX11G | -1.41 | 0 | 0 | -2.42 |
| PFKP | 3.08 | 2.06 | 1.85 | 4.02 |
| PGLYRP2 | -4.1 | -4.58 | 0 | -6.27 |
| PGM1 | -1.47 | 0 | 0 | -2.5 |
| PGRMC1 | -1.02 | 0 | 0 | -2.21 |
| PGRMC2 | -1.02 | 0 | 0 | 0 |
| PHLDB2 | -1.26 | 0 | 0 | 0 |
| PHYH | -2.12 | -2.32 | 0 | -4.09 |
| PID1 | -1.36 | -1.94 | 0 | -2.65 |
| PIGC | 1.44 | 0 | 0 | 0 |
| PIK3AP1 | -1.35 | 0 | 0 | -1.42 |
| PIPOX | -3.2 | -2.61 | -3.94 | -4.14 |
| PITX1 | 2.76 | 6.05 | 0 | 8.78 |
| PKP4 | 1.15 | 0 | 0 | 0 |
| PLA2G12B | -2.85 | -5.14 | 0 | -6.19 |
| PLA2G16 | -2.02 | 0 | 0 | 0 |
| PLEK | -1.73 | 0 | 0 | 0 |
| PLGLB1 | -3.44 | -4.92 | 0 | -6.14 |
| PLGLB2 | -1.5 | 0 | 0 | 0 |
| PLIN2 | -2.01 | 0 | 0 | -6.41 |
| PLOD2 | -1.67 | 0 | 0 | 0 |
| PLPP3 | -1.08 | 0 | 0 | 0 |
| PLVAP | 1.44 | 0 | 0 | 2.21 |
| PNPO | -1.26 | 0 | 0 | -1.94 |
| POGZ | 1.03 | 0 | 0 | 0 |
| PPARA | -1.08 | -1.65 | 0 | -2.7 |
| PPFIBP1 | 1.57 | 0 | 0 | 0 |
| PPFIBP2 | -1.52 | 0 | 0 | -1.05 |
| PPIEL | 1 | 0 | 0 | 0 |
| PPP2R1B | -1.63 | -1.31 | 0 | -2.17 |
| PRCP | -1.72 | 0 | 0 | 0 |
| PRG4 | -2.92 | 0 | -4.04 | -5.7 |
| PRKAA1 | -1.01 | 0 | 0 | 0 |
| PRSS55 | -1.18 | 0 | 0 | -1.89 |
| PSD3 | -1.15 | 0 | 0 | -1.54 |
| PSMA3 | -1.12 | 0 | 0 | 0 |
| PTBP3 | 1.22 | 0 | 1.19 | 1.63 |
| PTGES3 | -1.09 | 0 | 0 | 0 |
| PTK7 | 1.24 | 2.1 | 2.94 | 1.93 |
| PTMA | -1.06 | 0 | 0 | 1.13 |
| PTP4A1 | -1.42 | -1.1 | 0 | -2.42 |
| PTPN14 | 1.53 | 0 | 2.18 | 2.1 |
| PURB | 1.01 | 0 | 0 | 0 |
| PUS3 | -1.18 | 0 | 0 | -1.15 |
| PXMP2 | -2.55 | -2.62 | 0 | -3.53 |
| QKI | -1.34 | 0 | 0 | 0 |
| RAB33B | -1.72 | 0 | 0 | 0 |
| RAB3IP | 1.66 | 0 | 0 | 0 |
| RABEPK | -1.02 | 0 | 0 | 0 |
| RABL6 | 1.01 | 0 | 0 | 0 |
| RAD21 | -1.23 | 0 | 0 | 0 |
| RAP2C | -1.05 | 0 | 0 | 0 |
| RAPH1 | -1.09 | -1.31 | 0 | -2.04 |
| RARRES3 | -2.41 | 0 | 0 | 0 |
| RBP5 | -2.05 | -5.19 | 0 | -5.12 |
| RCC2 | 1.19 | 0 | 1.13 | 1.94 |
| RDH11 | -1.29 | 0 | 0 | 0 |
| RDH16 | -4.69 | -4.63 | -5.28 | -5.74 |
| RELN | -2.74 | 0 | 0 | -5.34 |
| RGN | -2.79 | -3.71 | 0 | -6.19 |
| RGPD8 | 1.06 | 0 | 0 | 0 |
| RHBDF1 | 1.7 | 0 | 0 | 1.77 |
| RHOB | -2.49 | -1.74 | -1.65 | -1.89 |
| RHPN2 | 1.23 | 0 | 1.56 | 0 |
| RIDA | -2.63 | 0 | 0 | 0 |
| RIPPLY1 | -1.11 | 0 | 0 | -2.17 |
| RNA18S5 | 1.38 | 0 | 0 | 0 |
| RNASE4 | -4.66 | 0 | -4.99 | -3.31 |
| RND1 | -1.28 | 0 | 0 | -3.14 |
| RNF113A | -1.04 | 0 | 0 | 0 |
| RNF128 | -2.02 | 0 | 0 | 0 |
| RNF19A | 1.12 | 0 | 0 | 0 |
| RNF24 | 1.12 | 0 | 0 | 1.15 |
| RPL15 | 2.41 | 0 | 2.33 | 0 |
| RPL23 | -1.11 | 0 | 0 | 0 |
| RPL23AP64 | -1.24 | 0 | 0 | 0 |
| RPL23AP87 | -1.19 | 0 | 0 | 0 |
| RPL7 | -1.54 | 0 | 0 | 0 |
| RPL9 | -1.26 | 0 | 0 | 0 |
| RPS27 | -1.14 | 0 | 0 | 0 |
| RPS28 | -1.68 | 0 | 0 | 0 |
| RRN3 | -1.01 | 0 | 0 | 0 |
| RSRP1 | 1.39 | 0 | 0 | 0 |
| RTN4 | -2.76 | 0 | 0 | 0 |
| SACM1L | -1.05 | 0 | 0 | 0 |
| SAR1A | -1.06 | 0 | 0 | 0 |
| SAR1B | -1.37 | 0 | 0 | -1.45 |
| SBDS | -1.38 | 0 | 0 | 0 |
| SDSL | -1.29 | 0 | 0 | -1.72 |
| SEC24A | -1.39 | 0 | 0 | 0 |
| SEC24D | -1.13 | 0 | 0 | 0 |
| SEPP1 | -3.54 | -3.83 | -2.13 | -3.6 |
| SEPT2 | 1.1 | 0 | 0 | 0 |
| SERP1 | -1.34 | 0 | 0 | 0 |
| SERPINA10 | -3.62 | -4.4 | -2.99 | -7.64 |
| SERPINA4 | -2.26 | -4.82 | 0 | -6.04 |
| SERPINA5 | -1.93 | -3.59 | 0 | -6.57 |
| SERPINA6 | -2.87 | -4.48 | 0 | -5.71 |
| SERPINA7 | -4.01 | -6.74 | 0 | -8.65 |
| SERPIND1 | -3.59 | 0 | -4.18 | -9.14 |
| SESTD1 | 1.24 | 0 | 0 | 1.27 |
| SEZ6L2 | 2.92 | 1.97 | 1.81 | 3.89 |
| SFXN2 | -1.11 | -1.31 | 0 | -1.98 |
| SGPP2 | 2.72 | 2.2 | 0 | 5.37 |
| SH3GLB2 | 1.54 | 0 | 0 | 1.06 |
| SHE | -1.02 | -2.54 | 0 | -1.46 |
| SHMT1 | -2.04 | -3.39 | -2.28 | -4.74 |
| SLC17A1 | -1 | -3.27 | 0 | -5.9 |
| SLC17A2 | -3.22 | -3.93 | -3.88 | -7.3 |
| SLC17A3 | -2.29 | -2.77 | 0 | -6.06 |
| SLC1A2 | -1.12 | -3.82 | 0 | -6.43 |
| SLC22A1 | -3.49 | -5.16 | -4.7 | -7.35 |
| SLC22A9 | -1.54 | -4.67 | 0 | -4.33 |
| SLC25A13 | -1.93 | 0 | 0 | -2.45 |
| SLC25A15 | -1.65 | -2.24 | -2.03 | -3.78 |
| SLC25A20 | -1.52 | -1.55 | 0 | -2.54 |
| SLC25A25 | -1.09 | 0 | 0 | -2.45 |
| SLC31A1 | -1.22 | 0 | 0 | -2.15 |
| SLC35E2B | 1.36 | 0 | 0 | 0 |
| SLC35F2 | 2.01 | 1.11 | 0 | 4.04 |
| SLC38A2 | -1.99 | 0 | 0 | 0 |
| SLC38A4 | -2.9 | -7 | 0 | -7.84 |
| SLC39A14 | -2.04 | 0 | 0 | -2.94 |
| SLC40A1 | -1 | 0 | 0 | 0 |
| SLC41A2 | -1.18 | 0 | 0 | -2.25 |
| SLC43A1 | -1.06 | -2.41 | 0 | -3.1 |
| SLC44A1 | 1.2 | 0 | 0 | 2.08 |
| SLC47A1 | -2.47 | -4.02 | 0 | -4.81 |
| SLC4A11 | 1.22 | 0 | 1.78 | 3.17 |
| SLC51A | -3.65 | 0 | 0 | 0 |
| SLC6A12 | -2.36 | -2.95 | -2.66 | -4.36 |
| SLC7A2 | -3.1 | -3.77 | -2.69 | -4.47 |
| SLC9A9 | -1.2 | 0 | 0 | 0 |
| SLCO1B3 | -2.88 | 0 | 0 | -4.33 |
| SLCO2B1 | -1.81 | 0 | 0 | -1.92 |
| SLCO3A1 | 1.61 | 0 | 0 | 1.01 |
| SMARCA1 | -1.29 | 0 | 0 | -1.94 |
| SMARCA2 | -1.12 | 0 | 0 | 0 |
| SMIM14 | -1.37 | 0 | 0 | 0 |
| SMOX | 1.5 | 1.59 | 0 | 2.44 |
| SMPDL3A | -1.15 | 0 | 0 | -1.68 |
| SNHG1 | 1.38 | 0 | 0 | 0 |
| SNHG10 | -1.02 | 0 | 0 | 0 |
| SNHG17 | 1.03 | 0 | 0 | 0 |
| SNORA70 | 1.36 | 0 | 0 | 0 |
| SNRNP70 | 1.15 | 0 | 0 | 0 |
| SNRPD3 | 1.12 | 0 | 0 | 0 |
| SNTB1 | -1.54 | 0 | 0 | -2.34 |
| SOD1 | -1.07 | 0 | 0 | -1.7 |
| SORD | -2.67 | -3.3 | 0 | -4.1 |
| SOX4 | 1.69 | 2.22 | 1.89 | 2.02 |
| SP100 | 1.25 | 0 | 0 | 0 |
| SPATA13 | 1.25 | 0 | 0 | 0 |
| SPP1 | 4.48 | 3.33 | 0 | 3.22 |
| SPRYD4 | -1.8 | -1.64 | -1.67 | -2.61 |
| SPTLC3 | -1.19 | -1.51 | 0 | -2 |
| ST6GAL1 | -2.16 | -2.08 | 0 | -2.98 |
| STAB2 | -1.63 | -3.44 | -2.23 | -5.64 |
| STARD5 | -1.91 | 0 | -1.59 | -1.33 |
| STEAP3 | -2.41 | 0 | 0 | -2.83 |
| STK36 | 1.58 | 1.76 | 0 | 0 |
| STX16 | 1.15 | 0 | 0 | 0 |
| SULF2 | -1.04 | 0 | 0 | 1.24 |
| SUMO1P3 | -1.01 | 0 | 0 | 0 |
| SUMO2 | -1.28 | 0 | 0 | 0 |
| SUPT5H | 1.01 | 0 | 0 | 0 |
| SUSD3 | -1.24 | 0 | 0 | 0 |
| SVIL | 1.62 | 0 | 0 | 0 |
| TAOK1 | 1.45 | 0 | 0 | 1.08 |
| TAPT1 | -1.12 | 0 | 0 | -1.47 |
| TAX1BP1 | 1.17 | 0 | 0 | 0 |
| TBC1D14 | 1.28 | 0 | 0 | 0 |
| TBC1D23 | 1.16 | 0 | 0 | 0 |
| TBCB | 1.37 | 0 | 0 | 1.08 |
| TBX15 | -1.85 | 0 | 0 | -4.38 |
| TCEA3 | -1.1 | -1.89 | 0 | -3.07 |
| TCEAL3 | 1.17 | 0 | -1.4 | 0 |
| TCEAL9 | 2.01 | 0 | 0 | 0 |
| TCF3 | 1.42 | 0 | 0 | 1.47 |
| TDO2 | -2.71 | -3.79 | -2.85 | -6.24 |
| TEAD2 | 2.06 | 1.85 | 0 | 0 |
| TFPI | -1.32 | 0 | 0 | -2.33 |
| TGFBRAP1 | 1.6 | 0 | 0 | 1.02 |
| TIA1 | 1.02 | 0 | 0 | 0 |
| TIMM8A | -1.12 | 0 | 0 | 0 |
| TKFC | -1.73 | 0 | 0 | 0 |
| TMED7 | -1.09 | 0 | 0 | 0 |
| TMEM106B | -1.18 | 0 | 0 | 0 |
| TMEM147 | 1.29 | 0 | 1.01 | 0 |
| TMEM184B | 1.16 | 0 | 0 | 1.97 |
| TMEM263 | -1.28 | 0 | 0 | 0 |
| TMEM45A | -2.44 | 0 | 0 | -1.42 |
| TMEM53 | -1.22 | 0 | 0 | -2.16 |
| TMEM54 | 2.35 | 1.51 | 0 | 2.88 |
| TMEM56 | -1.37 | -3.27 | 0 | -4.61 |
| TMEM86B | -1.94 | -1.73 | 0 | -1.35 |
| TMEM87A | 1.08 | 0 | 0 | 1.34 |
| TMIGD3 | -1.3 | 0 | 0 | 0 |
| TNFSF14 | -1.74 | 0 | 0 | -2.9 |
| TP53INP1 | -2.08 | 0 | 0 | -1.63 |
| TPM2 | 2.5 | 0 | -1.87 | 2.87 |
| TPM3 | 1.14 | 1.65 | 0 | 1.09 |
| TPPP3 | 1.12 | 0 | 0 | 2.09 |
| TPR | -3.57 | 0 | 0 | 0 |
| TRA2A | 1.29 | 0 | 0 | 0 |
| TRAK1 | 1.99 | 0 | 0 | 1.09 |
| TRIAP1 | -1.04 | 0 | 0 | 0 |
| TRIM38 | 1 | 0 | 0 | 0 |
| TSC22D1 | -1.09 | 0 | 0 | 0 |
| TSC22D2 | -1.38 | 0 | 0 | 0 |
| TSPAN12 | -1.06 | 0 | 0 | 0 |
| TSPAN3 | 1.27 | 0 | 0 | 1.95 |
| TSPAN6 | -1.07 | 0 | 0 | -2.07 |
| TTC36 | -1.75 | -5.04 | 0 | -7.12 |
| TTYH3 | 1.31 | 0 | 1.8 | 1.08 |
| TUBE1 | -1.06 | -1.25 | 0 | -1.79 |
| TXNL1 | -1.13 | 0 | 0 | 0 |
| U2SURP | 1.24 | 0 | 0 | 0 |
| UAP1 | -1.88 | 0 | -1.18 | -1.13 |
| UBAP2L | 1.46 | 1.08 | 0 | 0 |
| UBE4A | -1.14 | 0 | 0 | 0 |
| UBLCP1 | -1.03 | 0 | 0 | 0 |
| UBXN8 | -1.11 | 0 | 0 | -1.57 |
| UCKL1 | 1.03 | 0 | 0 | 0 |
| UGDH | -1.11 | 0 | 0 | -1.16 |
| UGGT1 | 1.02 | 0 | 0 | 0 |
| UGP2 | -2.22 | 0 | 0 | -2.63 |
| UGT1A1 | -2.74 | 0 | 0 | 0 |
| UGT1A3 | -1.05 | 0 | 0 | 0 |
| UGT1A4 | -2.11 | 0 | 0 | 0 |
| UGT1A7 | -1.09 | 0 | 0 | 0 |
| UGT1A9 | -1.2 | 0 | 0 | 0 |
| UGT2B10 | -4.71 | -5.62 | -4.1 | -6.86 |
| UGT2B11 | -4 | -5.02 | -2.97 | -7.06 |
| UGT2B28 | -2.95 | 0 | 0 | 0 |
| UGT2B4 | -3.69 | -6.18 | 0 | -9.34 |
| UGT3A1 | -1.82 | 0 | 0 | -4.78 |
| USH1C | 1.39 | 0 | 0 | 2.69 |
| USH2A | -1.29 | -5.16 | 0 | -7.1 |
| USP8 | -1.07 | 0 | 0 | 0 |
| VAT1 | 1.29 | 0 | 0 | 0 |
| VCAM1 | -1.02 | 0 | 0 | 0 |
| VCL | 1.2 | 0 | 0 | 1.88 |
| VKORC1 | -1.97 | 0 | 0 | -1.01 |
| VMO1 | -1.19 | 0 | 0 | -1.58 |
| VPS13C | 1.05 | 0 | 2.31 | 0 |
| VPS41 | 1.32 | 0 | 0 | 0 |
| VSNL1 | -1.29 | -2.06 | 0 | -2.85 |
| WRNIP1 | 1.15 | 0 | 1.05 | 0 |
| XDH | -1.86 | -3.41 | 0 | -4.91 |
| YES1 | -1.03 | 0 | 0 | 0 |
| YTHDC1 | 1.64 | 0 | 0 | 0 |
| YWHAE | 1.06 | 0 | 0 | 0 |
| YY1 | -1.35 | 0 | 0 | 0 |
| ZFP36L1 | -1.48 | 0 | 0 | -1.02 |
| ZKSCAN1 | 1.92 | 0 | 0 | -1.2 |
| ZMYND8 | 1.05 | 0 | 0 | 1.26 |
| ZNF260 | 1.1 | 0 | 0 | 0 |
| ZNF281 | -1.45 | 1.19 | 0 | 0 |
| ZNF320 | 1.35 | 0 | 0 | 1.35 |
| ZNF514 | 1.06 | 0 | 0 | 1.06 |
| ZNF526 | 1.62 | 0 | 0 | 0 |
| ZNF544 | 1.07 | 0 | 0 | 1.19 |
| ZNF83 | 1.64 | 0 | 0 | 1.74 |
| ZSCAN9 | 1.02 | 0 | 0 | 0 |
| A1BG | 0 | -6.17 | 0 | -6.09 |
| AASS | 0 | -1.68 | 0 | -3.02 |
| ABCA12 | 0 | 3.85 | 0 | 4.33 |
| ABCA5 | 0 | -1.38 | 0 | -1.87 |
| ABCA6 | 0 | -2.97 | 0 | -3.84 |
| ABCA8 | 0 | -4.01 | -3.57 | -4.01 |
| ABCB1 | 0 | -2.33 | 0 | -1.68 |
| ABCB11 | 0 | -4.48 | 0 | -5.75 |
| ABCB4 | 0 | -4.82 | 0 | -5.72 |
| ABCC1 | 0 | 1.23 | 0 | 2.6 |
| ABCC2 | 0 | -3.22 | 0 | -4.66 |
| ABCC6 | 0 | -3.61 | 0 | -5.46 |
| ABCC6P1 | 0 | -3.44 | 0 | 0 |
| ABCC9 | 0 | -2.1 | 0 | -2.96 |
| ABCG5 | 0 | -5.42 | 0 | -6.93 |
| ABCG8 | 0 | -6.18 | 0 | -7.27 |
| ABHD1 | 0 | -1.51 | -2.53 | -2.26 |
| ABHD6 | 0 | -2.11 | -2.34 | -2.65 |
| ACAA1 | 0 | -2.25 | -1.45 | -3.21 |
| ACAA2 | 0 | -1.93 | 0 | -3.3 |
| ACACB | 0 | -2.59 | -2.24 | -3.8 |
| ACADM | 0 | -1.71 | 0 | -2.9 |
| ACADSB | 0 | -4.09 | 0 | -4.7 |
| ACADVL | 0 | -1.17 | 0 | -1.55 |
| ACAT1 | 0 | -2.36 | -1.66 | -4.12 |
| ACMSD | 0 | -3.92 | 0 | -7.12 |
| ACOT12 | 0 | -5.93 | 0 | -7.19 |
| ACOT8 | 0 | 1.88 | 0 | 1.19 |
| ACOX1 | 0 | -2.37 | 0 | -2.96 |
| ACOX2 | 0 | -2.73 | -3.37 | -4.44 |
| ACSM3 | 0 | -2.48 | 0 | -3.39 |
| ACSM5 | 0 | -4.7 | 0 | -6.37 |
| ACSS3 | 0 | -2.13 | 0 | -2.8 |
| ACVR1 | 0 | 1.37 | 0 | 0 |
| ACY1 | 0 | -2.2 | 0 | -3.05 |
| ADAM12 | 0 | 1.87 | 0 | 2.08 |
| ADAMTS1 | 0 | -2.14 | -3.74 | 0 |
| ADAMTS12 | 0 | 2.51 | 0 | 2.43 |
| ADAMTS13 | 0 | -3.14 | 0 | -3.45 |
| ADAMTS14 | 0 | 3.18 | 0 | 4 |
| ADAMTSL2 | 0 | -2 | 0 | -1.8 |
| ADC | 0 | 1.78 | 0 | 1.65 |
| ADCK3 | 0 | -1.18 | 0 | -1.54 |
| ADH1A | 0 | -7.37 | 0 | -6.49 |
| ADH1B | 0 | -7.84 | -4.03 | -7.18 |
| ADH1C | 0 | -6.75 | 0 | -4.78 |
| ADH4 | 0 | -8.97 | 0 | -8.21 |
| ADHFE1 | 0 | -2.85 | -2.14 | -4.12 |
| ADRA2A | 0 | -2.25 | 0 | 0 |
| ADRB2 | 0 | -2.3 | -1.9 | -1.72 |
| AFM | 0 | -9.11 | 0 | -9.94 |
| AFMID | 0 | -1.53 | 0 | -2.52 |
| AFP | 0 | -3.43 | 0 | -5.52 |
| AGMAT | 0 | -3.39 | 0 | -3.61 |
| AGMO | 0 | -4.52 | 0 | -5 |
| AGPAT9 | 0 | -1.92 | 0 | 0 |
| AGRP | 0 | 2.13 | 0 | 0 |
| AGT | 0 | -2.8 | 0 | -5.12 |
| AGXT | 0 | -8.44 | 0 | -8.18 |
| AGXT2L1 | 0 | -5.56 | 0 | -8.44 |
| AHDC1 | 0 | -1.56 | 0 | -2.16 |
| AHNAK2 | 0 | 4.09 | 0 | 4.44 |
| AHSG | 0 | -8.86 | -4.62 | -8.92 |
| AIF1L | 0 | -2.44 | -1.99 | 0 |
| AKAP7 | 0 | -2.01 | -2.47 | 0 |
| AKIP1 | 0 | 1.12 | 0 | 0 |
| AKR1CL1 | 0 | -4.34 | 0 | 0 |
| AKR1D1 | 0 | -5.49 | 0 | -8.14 |
| AKR7A3 | 0 | -2.38 | 0 | -2.72 |
| AKR7L | 0 | -3.37 | 0 | 0 |
| ALB | 0 | -7.28 | 0 | -7.13 |
| ALDH1L1 | 0 | -5.27 | 0 | -6.68 |
| ALDH3A2 | 0 | -1.81 | 0 | -2.41 |
| ALDH7A1 | 0 | -1.31 | 0 | -2.31 |
| ALDH8A1 | 0 | -4.79 | 0 | -6.5 |
| ALDOA | 0 | 1.44 | 1.82 | 2.44 |
| ALDOB | 0 | -8.89 | 0 | -8.15 |
| ALOX5 | 0 | 1.72 | 0 | 1.78 |
| AMDHD1 | 0 | -5.21 | -3.22 | -6.26 |
| AMPD1 | 0 | -2.8 | 0 | 1.9 |
| AMT | 0 | -1.56 | 0 | -2.43 |
| AMY1C | 0 | -1.19 | -2.82 | -1.54 |
| ANK3 | 0 | -2.17 | -1.12 | 0 |
| ANKIB1 | 0 | 1.64 | 0 | 1.27 |
| ANKRD22 | 0 | 2.54 | 2.41 | 3.81 |
| ANKRD52 | 0 | 1.03 | 0 | 1.38 |
| ANLN | 0 | 4.21 | 2.34 | 5.91 |
| ANO8 | 0 | 2.41 | 0 | 0 |
| ANPEP | 0 | -2.5 | 0 | -2.84 |
| ANXA10 | 0 | -5.91 | 0 | -4.56 |
| ANXA2 | 0 | 2.17 | 1.52 | 3.46 |
| ANXA2P1 | 0 | 1.83 | 0 | 0 |
| ANXA2P3 | 0 | 4.54 | 0 | 0 |
| AOX1 | 0 | -5.12 | -3.79 | -6.33 |
| AP1S1 | 0 | 1.21 | 1.77 | 0 |
| AP1S3 | 0 | 2.14 | 0 | 1.92 |
| AP4M1 | 0 | 1.48 | 0 | 1.1 |
| APCS | 0 | -2.82 | 0 | -5.88 |
| APOA1 | 0 | -10.3 | 0 | -9.97 |
| APOA2 | 0 | -8.69 | 0 | -9.71 |
| APOA4 | 0 | -3.64 | 0 | -6.23 |
| APOA5 | 0 | -5.9 | -4.7 | -7.3 |
| APOB | 0 | -6.23 | 0 | -9.28 |
| APOC1 | 0 | -4.59 | 0 | -4.82 |
| APOC2 | 0 | -5.66 | -3.34 | -7.53 |
| APOC3 | 0 | -10.7 | -4.99 | -9.61 |
| APOC4 | 0 | -5.88 | 0 | -7.61 |
| APOE | 0 | -2.51 | 0 | -1.62 |
| APOF | 0 | -7.15 | -5.08 | -9.36 |
| APOM | 0 | -5.62 | -3.03 | -6.19 |
| AQP7 | 0 | -4.23 | 0 | -4.86 |
| AQP7P1 | 0 | -3.32 | 0 | 0 |
| AQP7P3 | 0 | -3.88 | 0 | 0 |
| AQP9 | 0 | -3.74 | -3.59 | -6 |
| AR | 0 | -3.17 | -2.39 | -5.24 |
| ARG1 | 0 | -8.64 | 0 | -10.1 |
| ARHGAP11B | 0 | 1.73 | 0 | 3.33 |
| ARHGEF26 | 0 | -2.19 | 0 | -3.8 |
| ARL6IP6 | 0 | 1.25 | 0 | 1.73 |
| ARL9 | 0 | 2.05 | 0 | 2.11 |
| ARMC9 | 0 | 2.63 | 0 | 2.83 |
| ARNTL2 | 0 | 2.11 | 0 | 0 |
| ARSE | 0 | -2.05 | 0 | -4.01 |
| ARSI | 0 | 2.46 | 0 | 0 |
| ART4 | 0 | -1.76 | 0 | -2.7 |
| ASGR1 | 0 | -4.91 | 0 | -6.33 |
| ASL | 0 | -1.93 | 0 | -2.89 |
| ASNS | 0 | 1.1 | 0 | 3.26 |
| ASPA | 0 | -2.58 | -2.75 | -3.31 |
| ASPG | 0 | -6.13 | 0 | -6.96 |
| ASPM | 0 | 3.03 | 3.14 | 3.13 |
| ASTN2 | 0 | 1.72 | -1.89 | 0 |
| ATOH8 | 0 | -2.41 | -2.58 | -3.88 |
| ATP1B2 | 0 | -2.38 | -2.15 | -1.41 |
| ATP6 | 0 | -1.87 | 0 | -1.07 |
| ATP6AP1L | 0 | 1.27 | 0 | 1.87 |
| ATP7B | 0 | -1.23 | 0 | -1.87 |
| ATP8 | 0 | -1.83 | 0 | -1.27 |
| AVPR1A | 0 | -3.04 | 0 | -5.07 |
| AZGP1 | 0 | -4.86 | -2.77 | -7.38 |
| BAALC | 0 | 2.75 | 0 | 0 |
| BAAT | 0 | -4.36 | 0 | -8.23 |
| BACE2 | 0 | 1.7 | 0 | 3.26 |
| BBC3 | 0 | 1.18 | 0 | 0 |
| BCL2L1 | 0 | 1.12 | 1.26 | 0 |
| BDH1 | 0 | -5.54 | 0 | -4.92 |
| BEX1 | 0 | -3.19 | 0 | -2.64 |
| BFSP1 | 0 | 1.86 | 0 | 1.6 |
| BHLHE41 | 0 | 1.49 | 2.02 | 4.11 |
| BHMT | 0 | -7.94 | 0 | -8.77 |
| BICD1 | 0 | 1.81 | 0 | 3.68 |
| BLNK | 0 | -1.33 | 0 | -1.07 |
| BLZF1 | 0 | 1.11 | 0 | 0 |
| BMP1 | 0 | 1.36 | 0 | 0 |
| BMP8A | 0 | 3.04 | 0 | 2.51 |
| BMPER | 0 | -4.02 | 0 | -4.08 |
| BMX | 0 | -2.72 | -1.58 | 0 |
| BOLA3 | 0 | 1.6 | 0 | 0 |
| BOP1 | 0 | 1.03 | 0 | 1.3 |
| BPHL | 0 | -1.6 | 0 | -2.71 |
| BRIP1 | 0 | 2.5 | 0 | 1.51 |
| BRP44L | 0 | -1.98 | 0 | -2.74 |
| BUB1 | 0 | 3.27 | 3.11 | 5.03 |
| BUB1B | 0 | 2.64 | 5.38 | 3.64 |
| C10orf114 | 0 | -2.08 | 0 | -3.68 |
| C10orf116 | 0 | -2.79 | 0 | 0 |
| C10orf125 | 0 | -1.65 | 0 | -3.48 |
| C10orf35 | 0 | 1.77 | 1.44 | 1.23 |
| C11orf35 | 0 | -1.09 | 0 | -1.28 |
| C11orf54 | 0 | -2.41 | 0 | -2.56 |
| C12orf32 | 0 | 1.1 | 0 | 0 |
| C12orf49 | 0 | 1.43 | 1.36 | 1.33 |
| C12orf70 | 0 | 2.59 | 0 | 1.85 |
| C12orf75 | 0 | 2.06 | 0 | 3.84 |
| C14orf180 | 0 | -2.9 | 0 | -2.97 |
| C15orf48 | 0 | 3.43 | 0 | 4.29 |
| C15orf52 | 0 | 1.55 | 0 | 1.56 |
| C16orf59 | 0 | 2.51 | 3.37 | 2.57 |
| C16orf88 | 0 | 1.25 | 0 | 1.55 |
| C18orf54 | 0 | 1.69 | 2.17 | 2.28 |
| C19orf21 | 0 | 2.95 | 0 | 6.52 |
| C19orf28 | 0 | 1.55 | 0 | 0 |
| C19orf66 | 0 | -1.18 | 0 | -2.08 |
| C19orf80 | 0 | -6.14 | 0 | -7.32 |
| C1orf112 | 0 | 1.09 | 0 | 0 |
| C1orf115 | 0 | -2.44 | 0 | -3.07 |
| C1orf168 | 0 | -5.72 | 0 | -6.7 |
| C1orf228 | 0 | -1.49 | 0 | 0 |
| C1QTNF1 | 0 | -1.1 | -1.72 | 0 |
| C1QTNF6 | 0 | 1.63 | 1.25 | 0 |
| C1R | 0 | -1.73 | 0 | -2.18 |
| C1S | 0 | -1.92 | 0 | -2.62 |
| C2 | 0 | -2.07 | 0 | -3.1 |
| C20orf118 | 0 | 3.11 | 0 | 3.83 |
| C20orf132 | 0 | -1.47 | 0 | -2 |
| C2CD4A | 0 | 3.74 | 0 | 2.08 |
| C2orf27A | 0 | 2.23 | 2.41 | 3.06 |
| C2orf63 | 0 | 1.31 | 0 | 1.63 |
| C2orf72 | 0 | -3.21 | 0 | -5.7 |
| C3orf23 | 0 | -1.05 | 0 | -2.05 |
| C3orf32 | 0 | -1.8 | 0 | 0 |
| C3orf64 | 0 | 2.04 | 0 | 1.31 |
| C3orf80 | 0 | 2.49 | 0 | 3.24 |
| C3P1 | 0 | -7.14 | 0 | 0 |
| C4B | 0 | -2.68 | 0 | -4.07 |
| C4orf19 | 0 | -1.34 | 0 | -3.48 |
| C4orf34 | 0 | -1.34 | 0 | -2.39 |
| C4orf7 | 0 | 3.32 | 0 | 7.38 |
| C5 | 0 | -3.49 | 0 | -6.16 |
| C5orf27 | 0 | -3.88 | 0 | 0 |
| C5orf4 | 0 | -1.83 | 0 | -2.97 |
| C5orf46 | 0 | 4 | 2.84 | 3.25 |
| C6orf225 | 0 | -1.74 | 0 | -1.52 |
| C7 | 0 | -4.72 | -3.36 | 0 |
| C7orf58 | 0 | -1.66 | 0 | -2.69 |
| C8G | 0 | -3.39 | 0 | -6.1 |
| C8orf60 | 0 | 1.11 | 0 | 1.79 |
| C8orf80 | 0 | -3.35 | 0 | -3.23 |
| C9orf103 | 0 | -1.61 | 0 | -2.48 |
| C9orf140 | 0 | 2.23 | 0 | 2.8 |
| C9orf30 | 0 | 1.15 | 0 | 0 |
| C9orf30-TMEFF1 | 0 | 2.39 | 0 | 2.22 |
| C9orf53 | 0 | 2.33 | 0 | 0 |
| C9orf68 | 0 | -1.8 | 0 | -1.92 |
| CA12 | 0 | 4.85 | 0 | 5.46 |
| CA14 | 0 | -1.83 | 0 | -2.19 |
| CA2 | 0 | -1.14 | 0 | -2.41 |
| CA5A | 0 | -2.21 | -3.87 | -3.11 |
| CABYR | 0 | 1.86 | 0 | 3.68 |
| CACNB3 | 0 | 1.47 | 0 | 2.42 |
| CACYBP | 0 | 1.23 | 0 | 0 |
| CALB2 | 0 | 3.71 | 0 | 5.09 |
| CALU | 0 | 1.32 | 0 | 1.37 |
| CAMK2N2 | 0 | 1.57 | 0 | 0 |
| CAPG | 0 | 2.09 | 2.79 | 4.84 |
| CAPN2 | 0 | 1.31 | 0 | 2.07 |
| CAPN3 | 0 | -1.41 | 0 | -1.5 |
| CAPN5 | 0 | -1.45 | 0 | 0 |
| CAPRIN2 | 0 | 1.69 | 0 | 0 |
| CASC5 | 0 | 2.5 | 0 | 3.08 |
| CBFA2T3 | 0 | -2.49 | 0 | 0 |
| CBR3 | 0 | 2.71 | 0 | 2.91 |
| CBS | 0 | -1.97 | 0 | -4.89 |
| CBX1 | 0 | 1.05 | 0 | 1.2 |
| CCBE1 | 0 | -3.7 | 0 | -3.86 |
| CCDC34 | 0 | 1.57 | 0 | 1.86 |
| CCDC74B | 0 | 2.42 | 2.56 | 3.02 |
| CCDC99 | 0 | 1.25 | 0 | 2.21 |
| CCL14 | 0 | -4.1 | 0 | -3.77 |
| CCL15 | 0 | -2.13 | 0 | -2.37 |
| CCL16 | 0 | -6.71 | -3.38 | -7.79 |
| CCL19 | 0 | -5.12 | -3.33 | 0 |
| CCL21 | 0 | -3.26 | 0 | -1.77 |
| CCNB1 | 0 | 2.33 | 3.41 | 1.4 |
| CCNE1 | 0 | 1.1 | 3.36 | 0 |
| CCNE2 | 0 | 2.03 | 0 | 2.51 |
| CCNJL | 0 | 2.31 | 0 | 0 |
| CCNO | 0 | 2.33 | 2.34 | 4.53 |
| CD209 | 0 | -2.47 | 0 | -2.64 |
| CD276 | 0 | 1.51 | 0 | 0 |
| CD302 | 0 | -2.81 | 0 | -3.3 |
| CD58 | 0 | 1.12 | 0 | 1.76 |
| CDC14C | 0 | -1.12 | 0 | 0 |
| CDC37L1 | 0 | -1.74 | 0 | -1.64 |
| CDC45 | 0 | 2.92 | 0 | 3.34 |
| CDCA2 | 0 | 3.67 | 2.76 | 4.13 |
| CDCA5 | 0 | 3 | 2.78 | 4.39 |
| CDCA8 | 0 | 2.52 | 4.02 | 3.7 |
| CDCP1 | 0 | 2.46 | 2.98 | 4.2 |
| CDH19 | 0 | -2.93 | -4.19 | -3.29 |
| CDH3 | 0 | 3.17 | 6.87 | 5.85 |
| CDK1 | 0 | 3.62 | 2.42 | 3.41 |
| CDKL5 | 0 | 1.04 | 0 | 0 |
| CDKN2A | 0 | 2.16 | 0 | 2.8 |
| CDKN2B | 0 | 3.8 | 2.68 | 2.66 |
| CDKN3 | 0 | 3.17 | 2.5 | 4.55 |
| CDO1 | 0 | -6.3 | -3.73 | -6.33 |
| CDT1 | 0 | 3.83 | 3.35 | 3.53 |
| CEACAM20 | 0 | 2.65 | 0 | 0 |
| CEACAM7 | 0 | 3.14 | 0 | 5.21 |
| CECR2 | 0 | -1.11 | 0 | -4.16 |
| CELSR1 | 0 | -1 | 0 | 2.19 |
| CEND1 | 0 | 1.82 | 0 | 1.31 |
| CENPA | 0 | 3.52 | 0 | 5.56 |
| CENPF | 0 | 3.11 | 3.87 | 4.12 |
| CENPK | 0 | 2.75 | 0 | 3.17 |
| CENPM | 0 | 3.89 | 3.57 | 4.32 |
| CENPN | 0 | 1.97 | 1.34 | 1.74 |
| CENPO | 0 | 2.84 | 0 | 1.38 |
| CENPW | 0 | 2.42 | 0 | 3.22 |
| CEP55 | 0 | 3.9 | 5.59 | 4.43 |
| CEP89 | 0 | 1.88 | 0 | 1.65 |
| CERCAM | 0 | 1.96 | 0 | 3.32 |
| CERS4 | 0 | -1.33 | 0 | -2.48 |
| CES1 | 0 | -5.94 | 0 | -5.99 |
| CES1P2 | 0 | -4.55 | 0 | 0 |
| CFB | 0 | -2.59 | 0 | -4.26 |
| CFHR2 | 0 | -7.99 | 0 | -9.8 |
| CFHR4 | 0 | -7.09 | 0 | -8.34 |
| CFI | 0 | -2.21 | 0 | -4.05 |
| CHAF1B | 0 | 1.31 | 0 | 2.82 |
| CHDH | 0 | -1.27 | 0 | -2.52 |
| CHEK1 | 0 | 3.32 | 4.35 | 3.91 |
| CHN2 | 0 | -2.12 | 0 | -2.4 |
| CHPF | 0 | 1.5 | 1.36 | 0 |
| CHRD | 0 | -2.17 | -2.27 | -3.49 |
| CHRNA5 | 0 | 2.64 | 0 | 3.73 |
| CHST3 | 0 | 2.07 | 0 | 2.3 |
| CHST4 | 0 | -2.82 | 0 | 0 |
| CHST6 | 0 | 3.69 | 0 | -1.17 |
| CKAP2L | 0 | 3.09 | 3.94 | 3.95 |
| CKAP4 | 0 | 1.66 | 0 | 0 |
| CKLF | 0 | 2.91 | 0 | 1.54 |
| CLCN5 | 0 | -1.13 | 0 | -1.49 |
| CLEC10A | 0 | -2.1 | 0 | 0 |
| CLEC3B | 0 | -3.34 | -2.44 | -2.5 |
| CLEC4M | 0 | -2.47 | 0 | -6.37 |
| CLTB | 0 | 1.89 | 0 | 1.51 |
| CLU | 0 | -2.99 | 0 | -4.54 |
| CMTM3 | 0 | 1.56 | 0 | 1.29 |
| CMTM4 | 0 | 1.61 | 0 | 1.33 |
| CNTFR | 0 | -4.22 | -4.15 | -3.18 |
| COBLL1 | 0 | -1.7 | 0 | -2.34 |
| COL10A1 | 0 | 3.88 | 0 | 3.98 |
| COL13A1 | 0 | 3.38 | 0 | 2.19 |
| COL16A1 | 0 | 2 | 0 | 1.48 |
| COL1A1 | 0 | 4.49 | 2.37 | 5.44 |
| COL1A2 | 0 | 2.72 | 0 | 3.84 |
| COL4A1 | 0 | 2.65 | 0 | 2.78 |
| COL4A2 | 0 | 2.34 | 0 | 2.87 |
| COL5A1 | 0 | 2.85 | 0 | 3.62 |
| COL6A3 | 0 | 2.26 | 0 | 2.79 |
| COLEC10 | 0 | -3.75 | 0 | -5.47 |
| COMP | 0 | 6.32 | 3.77 | 6.29 |
| CORIN | 0 | 2.26 | 0 | 1.59 |
| CORO2A | 0 | 1.48 | 1.49 | 2.32 |
| COX1 | 0 | -1.55 | 0 | 0 |
| COX2 | 0 | -1.64 | 0 | 0 |
| COX3 | 0 | -1.42 | 0 | 0 |
| CPAMD8 | 0 | -1.78 | 0 | 0 |
| CPN1 | 0 | -3.17 | 0 | -5.24 |
| CPN2 | 0 | -4.58 | 0 | -5.86 |
| CPNE7 | 0 | 2.72 | 0 | 3.24 |
| CPS1 | 0 | -7.38 | 0 | -6.75 |
| CPS1-IT1 | 0 | -2.99 | 0 | 0 |
| CRMP1 | 0 | 1.71 | 0 | 1.23 |
| CRNDE | 0 | 2.1 | 0 | 0 |
| CRY2 | 0 | -1.08 | -1.28 | -1.57 |
| CSRNP1 | 0 | -1.31 | -2.26 | 0 |
| CST1 | 0 | 4.1 | 6.25 | 5.21 |
| CST2 | 0 | 3.53 | 4.71 | 2.55 |
| CST6 | 0 | 2.89 | 0 | 5.42 |
| CTSL2 | 0 | 2.8 | 0 | 4.32 |
| CXCL11 | 0 | 2.4 | 0 | 4.36 |
| CXCL12 | 0 | -3.69 | -2.26 | -1.75 |
| CYB561 | 0 | 1.27 | 0 | 1.81 |
| CYB561D1 | 0 | 1.4 | 0 | 0 |
| CYB5A | 0 | -3.33 | 0 | -3.86 |
| CYP1A2 | 0 | -6.14 | 0 | -7.89 |
| CYP21A2 | 0 | -1.47 | 0 | -2.56 |
| CYP27A1 | 0 | -3.13 | 0 | -3.75 |
| CYP2A13 | 0 | -5.58 | 0 | -6.26 |
| CYP2C18 | 0 | -2.92 | 0 | -3.85 |
| CYP2C19 | 0 | -5.44 | 0 | -6.17 |
| CYP2C9 | 0 | -5.06 | 0 | -6.47 |
| CYP2D6 | 0 | -3.92 | 0 | -5.98 |
| CYP2E1 | 0 | -7.21 | -4.73 | -8.63 |
| CYP2J2 | 0 | -3.65 | 0 | -5.66 |
| CYP3A4 | 0 | -5.43 | 0 | -7.85 |
| CYP3A5 | 0 | -3.25 | 0 | -4.73 |
| CYP3A7 | 0 | -3.59 | 0 | -7.1 |
| CYP4A11 | 0 | -7.28 | -4.38 | -8.03 |
| CYP4F11 | 0 | -3.26 | 0 | -3.67 |
| CYP4F12 | 0 | -3.73 | 0 | -3.25 |
| CYP4F3 | 0 | -5.74 | 0 | -5.8 |
| CYP4F8 | 0 | -2.32 | 0 | -3.13 |
| CYP4V2 | 0 | -1.74 | 0 | -3.02 |
| CYP7A1 | 0 | -4.23 | 0 | -5.86 |
| CYP7B1 | 0 | -1.57 | 0 | -2.15 |
| CYP8B1 | 0 | -8.92 | -4.56 | -9.72 |
| CYTB | 0 | -1.9 | 0 | -1.06 |
| DAK | 0 | -1.71 | 0 | -3.07 |
| DAO | 0 | -5.81 | 0 | -8.08 |
| DCBLD2 | 0 | 1.82 | 0 | 0 |
| DCTN5 | 0 | 1.74 | 0 | 1.07 |
| DCXR | 0 | -4.1 | -2.34 | -4.41 |
| DDC | 0 | -5.12 | 0 | -5.3 |
| DDI2 | 0 | -1.24 | 0 | -1.17 |
| DECR2 | 0 | -1.29 | 0 | -2.56 |
| DEFA3 | 0 | -4.18 | 0 | -2.73 |
| DENND2C | 0 | -1.51 | 0 | -2.31 |
| DENND3 | 0 | 1.02 | 0 | 1.24 |
| DEPDC1 | 0 | 1.3 | 0 | 3.4 |
| DES | 0 | -1.51 | -4.78 | 0 |
| DHODH | 0 | -2.7 | 0 | -3.38 |
| DHRS1 | 0 | -1.44 | 0 | -2.64 |
| DHRS12 | 0 | -1.07 | 0 | -1.29 |
| DHTKD1 | 0 | -2.08 | 0 | -2.97 |
| DIAPH3 | 0 | 3.98 | 0 | 1.78 |
| DIO2 | 0 | 2.33 | 0 | 3.85 |
| DKFZp434J0226 | 0 | 4.03 | 0 | 0 |
| DKK1 | 0 | 4.18 | 0 | 6.11 |
| DLGAP5 | 0 | 4.13 | 6.18 | 4.84 |
| DNAH1 | 0 | -1.28 | 0 | -1.26 |
| DNAH14 | 0 | 1.57 | 0 | 0 |
| DNAJB13 | 0 | 1.79 | 0 | 0 |
| DNHD1 | 0 | -1.35 | -1.31 | 0 |
| DOCK7 | 0 | 1.29 | 0 | 0 |
| DPCD | 0 | 1.27 | 0 | 1.22 |
| DPF3 | 0 | -1.99 | 0 | -2.52 |
| DPT | 0 | -5.2 | -4.81 | 0 |
| DSN1 | 0 | 1.44 | 0 | 1.12 |
| DSTNP2 | 0 | 2.26 | 0 | 0 |
| DUSP8 | 0 | -2.2 | 0 | -3.27 |
| DUX4 | 0 | -1.13 | 0 | 0 |
| DYNC2LI1 | 0 | 1.64 | 0 | -1.2 |
| E2F3 | 0 | 1 | 1.23 | 1.42 |
| E2F7 | 0 | 3.66 | 0 | 3.79 |
| EBP | 0 | -1.39 | 0 | -2.15 |
| EBPL | 0 | -1.43 | 0 | -1.39 |
| ECH1 | 0 | -1.25 | 0 | -1.84 |
| ECHDC2 | 0 | -2.28 | 0 | -3.02 |
| ECHS1 | 0 | -1.99 | -1.17 | -2.96 |
| EDARADD | 0 | 1.75 | 0 | 0 |
| EDIL3 | 0 | 2.52 | 0 | 5.3 |
| EDNRB | 0 | -2.24 | -2.14 | -2.31 |
| EFNA4 | 0 | 1.81 | 1.44 | 1.44 |
| EGFL6 | 0 | 2.88 | 0 | 6.46 |
| EGFL8 | 0 | -1.19 | 0 | 0 |
| EGLN3 | 0 | 1.91 | 0 | 2.95 |
| EGR1 | 0 | -2.04 | 0 | -2.09 |
| EHHADH | 0 | -3.95 | -2.11 | -5.03 |
| ELFN2 | 0 | 2.52 | 0 | 0 |
| ELOVL2 | 0 | -2.13 | 0 | -4.3 |
| EME1 | 0 | 1.08 | 2.2 | 1.55 |
| EMP2 | 0 | -1.61 | 0 | 0 |
| ENAH | 0 | 1.42 | 0 | 1.36 |
| ENHO | 0 | -2.94 | 0 | -4.78 |
| ENO2 | 0 | 2.46 | 0 | 3.27 |
| ENO3 | 0 | -1.93 | 0 | -4.07 |
| ENTPD8 | 0 | -2.73 | 0 | -3.2 |
| EPB41L4B | 0 | -3.39 | -1.53 | -3.3 |
| EPB41L5 | 0 | -1.66 | 0 | -2.78 |
| EPHA10 | 0 | 2.62 | 0 | 4.9 |
| EPHX1 | 0 | -3.58 | -2.05 | -3.85 |
| ERO1L | 0 | 1.42 | 0 | 1.18 |
| ETFB | 0 | -1.58 | 0 | -2.19 |
| ETNK2 | 0 | -2.92 | 0 | -5.68 |
| ETV1 | 0 | 2.26 | 0 | 1.31 |
| EVC | 0 | 1.59 | 0 | 0 |
| EXOC3L4 | 0 | -2.56 | 0 | -2.91 |
| EXTL1 | 0 | 2.44 | 0 | 1.5 |
| EZH2 | 0 | 1.52 | 1.62 | 2.27 |
| F10 | 0 | -3.04 | 0 | -4.82 |
| F12 | 0 | -6.05 | -3.08 | -5.7 |
| F13B | 0 | -6.93 | 0 | -8.01 |
| F2R | 0 | 1.89 | 0 | 1.38 |
| F2RL2 | 0 | 2.26 | 0 | 0 |
| F5 | 0 | -2.09 | 0 | -5.48 |
| F7 | 0 | -3.36 | 0 | -5.7 |
| F9 | 0 | -8.46 | -4.58 | -10.2 |
| FABP1 | 0 | -9.44 | -4.59 | -7.95 |
| FAM100B | 0 | 1.11 | 0 | 1.64 |
| FAM107A | 0 | -4.39 | -4.21 | 0 |
| FAM108C1 | 0 | 1.74 | 0 | 3.25 |
| FAM110C | 0 | -1.34 | 0 | -1.48 |
| FAM134B | 0 | -1.46 | 0 | -2.05 |
| FAM149A | 0 | -1.43 | 0 | -2.84 |
| FAM151A | 0 | -3.72 | 0 | -4.51 |
| FAM153B | 0 | -2.45 | 0 | 0 |
| FAM155B | 0 | 3.01 | 0 | 0 |
| FAM169A | 0 | -1.45 | 0 | 0 |
| FAM171B | 0 | 1.71 | 0 | 2.38 |
| FAM176A | 0 | -1.97 | 0 | -4.24 |
| FAM189B | 0 | 1.18 | 0 | 1.31 |
| FAM198A | 0 | -1.76 | 0 | -2 |
| FAM19A5 | 0 | 2.55 | 0 | 2.31 |
| FAM20A | 0 | -1.56 | 0 | -2.53 |
| FAM46A | 0 | -1.75 | 0 | 0 |
| FAM57A | 0 | 1.64 | 0 | 1.72 |
| FAM66C | 0 | 1.81 | 0 | 0 |
| FAM78B | 0 | 2.17 | 0 | 0 |
| FAM81A | 0 | 2.57 | 3.32 | 4.36 |
| FAM84A | 0 | 2.46 | 0 | 0 |
| FAM99A | 0 | -8.09 | 0 | 0 |
| FAM9B | 0 | -2.79 | 0 | -4.06 |
| FANCI | 0 | 1.88 | 2.6 | 3.23 |
| FAT1 | 0 | 1.62 | 1.57 | 2.15 |
| FBLN1 | 0 | -2.27 | -3.22 | 0 |
| FBLN5 | 0 | -2.43 | -1.83 | 0 |
| FBP1 | 0 | -3.76 | 0 | -3.88 |
| FBXL18 | 0 | 1.51 | 0 | 1.22 |
| FBXL2 | 0 | 1.7 | 0 | 2.77 |
| FBXO32 | 0 | 2.01 | 0 | 2.89 |
| FBXO41 | 0 | 1.35 | 0 | 2.26 |
| FCN1 | 0 | -1.43 | -2.81 | 0 |
| FCRL1 | 0 | -1.38 | 0 | 0 |
| FCRL2 | 0 | -1.31 | 0 | 0 |
| FDX1 | 0 | -1.29 | 0 | -1.9 |
| FERMT1 | 0 | 1.83 | 3.64 | 2.3 |
| FETUB | 0 | -7.31 | -3.78 | -9.18 |
| FGD6 | 0 | 2.19 | 3.86 | 1.86 |
| FGF1 | 0 | 2.96 | 0 | 2.12 |
| FGFR2 | 0 | -2.13 | 0 | -2.04 |
| FHOD3 | 0 | 2.38 | 0 | 0 |
| FKBP10 | 0 | 1.85 | 0 | 2.36 |
| FKBP14 | 0 | 1.3 | 0 | 1.85 |
| FLJ22763 | 0 | -5.07 | 0 | 0 |
| FLJ27352 | 0 | 1.75 | 0 | 2 |
| FLJ37644 | 0 | -1.65 | 0 | 0 |
| FLJ40606 | 0 | -1.34 | 0 | -1.02 |
| FLJ42875 | 0 | -1.23 | 0 | 0 |
| FMNL2 | 0 | 1.16 | 0 | 1.21 |
| FMO3 | 0 | -3.75 | 0 | -5.42 |
| FMO5 | 0 | -5.24 | 0 | -6 |
| FNDC1 | 0 | 2.58 | 4.41 | 4.6 |
| FOLH1 | 0 | -2.61 | 0 | -4.2 |
| FOLH1B | 0 | -2.31 | 0 | -4.39 |
| FOS | 0 | -2.2 | -1.8 | 0 |
| FOSB | 0 | -4.36 | -4.36 | 0 |
| FOXA2 | 0 | -1.78 | 0 | -4.02 |
| FOXF2 | 0 | 4.48 | 0 | 4.02 |
| FOXM1 | 0 | 3.46 | 0 | 3.53 |
| FOXQ1 | 0 | 1.63 | 0 | 4.44 |
| FRMD5 | 0 | 3.01 | 0 | 4.11 |
| FRMD6 | 0 | 1.52 | 0 | 0 |
| FSD1L | 0 | 1.04 | 0 | 1.56 |
| FST | 0 | -1.89 | 0 | -3.18 |
| FTCD | 0 | -5.95 | 0 | -8.02 |
| FXYD1 | 0 | -3.84 | -4.01 | -4.15 |
| G6PC | 0 | -6.98 | -4 | -9.56 |
| GADD45G | 0 | -2.77 | -3.06 | -3.03 |
| GAL3ST1 | 0 | 2.36 | 0 | 0 |
| GAL3ST4 | 0 | 1.95 | 0 | 2.25 |
| GALM | 0 | -1.14 | 0 | -1.05 |
| GALNT10 | 0 | 1.63 | 0 | 2.45 |
| GALNT9 | 0 | 2.5 | 0 | 0 |
| GAMT | 0 | -2.74 | 0 | -4.85 |
| GAS2L3 | 0 | 1.73 | 0 | 1.07 |
| GATA2 | 0 | -1.31 | -1.43 | 0 |
| GATA4 | 0 | -1.28 | 0 | -2.23 |
| GATM | 0 | -3.94 | 0 | -4.98 |
| GBP7 | 0 | -6.27 | 0 | -7.48 |
| GCAT | 0 | -2.14 | 0 | -2.65 |
| GCDH | 0 | -1.64 | 0 | -2.45 |
| GCGR | 0 | -7.41 | -4.21 | -7.82 |
| GFOD1 | 0 | -1.33 | -1.29 | 0 |
| GFRA1 | 0 | -3.68 | 0 | -3.65 |
| GFRA2 | 0 | -2.55 | -2.19 | -2.09 |
| GGN | 0 | 1.22 | 0 | 0 |
| GINS1 | 0 | 3.03 | 0 | 3.46 |
| GJA1 | 0 | 1.86 | 0 | 3.05 |
| GJA4 | 0 | -2.51 | -2 | 0 |
| GJB1 | 0 | -2.5 | 0 | -4.38 |
| GJB3 | 0 | 3.23 | 4.91 | 4.01 |
| GLDC | 0 | -3.43 | 0 | -5.54 |
| GLT1D1 | 0 | -2.82 | 0 | -5.16 |
| GLT25D1 | 0 | 1.41 | 0 | 1.32 |
| GLTPD2 | 0 | -1.97 | 0 | -3.5 |
| GLUD1 | 0 | -1.7 | 0 | -2.55 |
| GLUD2 | 0 | -1.51 | 0 | -2.48 |
| GLYCTK | 0 | -3.42 | 0 | -4.23 |
| GMPS | 0 | 1.03 | 0 | 1.02 |
| GNAO1 | 0 | -2.84 | 0 | -3.81 |
| GOLM1 | 0 | 1.66 | 0 | 3.02 |
| GOLT1A | 0 | -2.06 | 0 | -4.82 |
| GOT1 | 0 | -1.69 | 0 | -3 |
| GPAM | 0 | -3.3 | 0 | -3.7 |
| GPER | 0 | -2.36 | 0 | -3.34 |
| GPIHBP1 | 0 | -2.62 | -2.27 | 0 |
| GPLD1 | 0 | -4.32 | 0 | -5.14 |
| GPM6B | 0 | -2.16 | -6.23 | 0 |
| GPR172A | 0 | 1.37 | 0 | 2.16 |
| GPR20 | 0 | -2.17 | 0 | 0 |
| GPR56 | 0 | 1.73 | 0 | 3.73 |
| GPT2 | 0 | -3.16 | -2.05 | -4.07 |
| GPX3 | 0 | -3.58 | -2.68 | -2.5 |
| GPX8 | 0 | 2.89 | 0 | 3.42 |
| GRAMD1C | 0 | -2.01 | 0 | -2.5 |
| GRB14 | 0 | -2.81 | 0 | -4.33 |
| GREM1 | 0 | 3.71 | 0 | 3.63 |
| GREM2 | 0 | -5.26 | 0 | -4.79 |
| GSTA2 | 0 | -6.87 | 0 | -6.31 |
| GSTA5 | 0 | -7.29 | -4.1 | -7.19 |
| GSTA7P | 0 | -6.5 | 0 | 0 |
| GSTM5 | 0 | -1.8 | -3.38 | 0 |
| GTSE1 | 0 | 3.11 | 0 | 4.28 |
| GULP1 | 0 | 1.8 | 0 | 3.51 |
| H19 | 0 | -4.24 | 0 | 0 |
| HABP2 | 0 | -3.43 | 0 | -4.82 |
| HADH | 0 | -1.75 | 0 | -1.82 |
| HAGH | 0 | -1.31 | 0 | -2.62 |
| HAMP | 0 | -5.56 | -4.63 | -6.81 |
| HAS3 | 0 | 3.02 | 2.26 | 0 |
| HBD | 0 | -4.43 | 0 | -2.35 |
| HBG1 | 0 | -1.26 | 0 | -2.27 |
| HDC | 0 | -1.39 | -2.39 | 0 |
| HEATR2 | 0 | 1.07 | 0 | 0 |
| HELLS | 0 | 1.34 | 0 | 2.04 |
| HFE2 | 0 | -5.96 | -4.24 | -8.18 |
| HGFAC | 0 | -5.38 | -4.12 | -6.83 |
| HIBADH | 0 | -1.13 | 0 | -1.8 |
| HIBCH | 0 | -1.2 | 0 | -1.19 |
| HIST1H1B | 0 | 1.95 | 0 | 3.76 |
| HIST1H2AA | 0 | 2.19 | 0 | 0 |
| HIST1H2AB | 0 | 1.6 | 0 | 2.67 |
| HIST1H2AC | 0 | 1.51 | 0 | 1.59 |
| HIST1H2AD | 0 | 1.44 | 0 | 1.51 |
| HIST1H2AE | 0 | 1.71 | 0 | 1.98 |
| HIST1H2AH | 0 | 1.15 | 0 | 2.01 |
| HIST1H2AI | 0 | 2.57 | 0 | 4.69 |
| HIST1H2AL | 0 | 2.89 | 0 | 3.79 |
| HIST1H2BC | 0 | 1.21 | 0 | 1.28 |
| HIST1H2BD | 0 | 1.17 | 1.42 | 1.62 |
| HIST1H2BE | 0 | 1.41 | 0 | 1.56 |
| HIST1H2BF | 0 | 1.88 | 0 | 3.21 |
| HIST1H2BG | 0 | 1.58 | 0 | 2.13 |
| HIST1H2BH | 0 | 1.05 | 0 | 1.6 |
| HIST1H2BI | 0 | 1.59 | 0 | 1.88 |
| HIST1H2BJ | 0 | 1.37 | 0 | 2.3 |
| HIST1H2BM | 0 | 1.16 | 0 | 2.01 |
| HIST1H2BO | 0 | 1.05 | 0 | 1.61 |
| HIST1H3B | 0 | 1.63 | 0 | 3.69 |
| HIST1H4A | 0 | 2.11 | 0 | 1.85 |
| HIST2H2AA4 | 0 | 1.85 | 0 | 1.51 |
| HIST2H2BF | 0 | 1.67 | 0 | 2 |
| HJURP | 0 | 4.34 | 2.79 | 4.61 |
| HLF | 0 | -3.39 | 0 | -5.04 |
| HMGA2 | 0 | 4.91 | 0 | 4.28 |
| HN1 | 0 | 1.77 | 0 | 2.69 |
| HN1L | 0 | 1.49 | 1.11 | 0 |
| HNF1A | 0 | -1.92 | 0 | -2.68 |
| HOGA1 | 0 | -3.78 | 0 | -5.74 |
| HOMER2 | 0 | -1.92 | 0 | -2.96 |
| HOMER3 | 0 | 1.63 | 1.52 | 1.45 |
| HOXB2 | 0 | 2.04 | 0 | 2.76 |
| HOXB6 | 0 | 1.77 | 1.79 | 2.46 |
| HP | 0 | -7.2 | 0 | -6.04 |
| HPGD | 0 | -2.63 | 0 | -2.79 |
| HPN | 0 | -3.04 | 0 | -6.48 |
| HPR | 0 | -7.79 | 0 | -7.39 |
| HRAS | 0 | 1.22 | 0 | 1.25 |
| HRC | 0 | -1.63 | -3.53 | 0 |
| HRSP12 | 0 | -3.73 | 0 | -4.49 |
| HSD11B1 | 0 | -3.4 | -3.31 | -4.79 |
| HSD17B2 | 0 | -3.15 | 0 | -3.21 |
| HSD17B4 | 0 | -1.24 | 0 | -2.23 |
| HSPB1 | 0 | 1.12 | 0 | 1.01 |
| HSPB6 | 0 | -1.28 | -3.67 | 0 |
| HTRA3 | 0 | 2.63 | 0 | 3.5 |
| HULC | 0 | -5.45 | 0 | 0 |
| IBSP | 0 | 3.37 | 0 | 0 |
| IDO2 | 0 | -3.86 | 0 | -4.05 |
| IFIT2 | 0 | 1.42 | 0 | 0 |
| IGFBP2 | 0 | -2.52 | 0 | -2.76 |
| IGFL2 | 0 | 5.08 | 5.08 | 6.2 |
| IGJ | 0 | -2.78 | 0 | 0 |
| IL11 | 0 | 3.65 | 0 | 4.6 |
| IL17RB | 0 | -1.66 | 0 | 0 |
| IL18R1 | 0 | -1.69 | 0 | 0 |
| IL27 | 0 | -1.77 | 0 | -2.78 |
| IL33 | 0 | -2.68 | -2.48 | 0 |
| IL6R | 0 | -2.02 | 0 | -2.69 |
| IL6ST | 0 | -1.68 | 0 | -2.26 |
| INHBA | 0 | 2.84 | 0 | 1.68 |
| INMT | 0 | -2.46 | -2.25 | 0 |
| INSIG1 | 0 | -2.41 | -2.3 | -3.1 |
| INTS6 | 0 | -1.42 | 0 | -1.48 |
| IQCA1 | 0 | 2.15 | 0 | 1.9 |
| IQCD | 0 | 1.56 | 0 | 3.01 |
| IQCE | 0 | 1.62 | 0 | 1.21 |
| IQCG | 0 | 1.1 | 0 | 0 |
| IQGAP2 | 0 | -1.1 | 0 | -1.35 |
| IQGAP3 | 0 | 2.51 | 2.71 | 4.12 |
| IQSEC2 | 0 | 1.04 | 0 | 1.82 |
| IRAK1 | 0 | 1 | 0 | 1.34 |
| IRX3 | 0 | 2.34 | 0 | 0 |
| ISG15 | 0 | 1.36 | 0 | 1.43 |
| ITGA11 | 0 | 2.34 | 2.58 | 2.67 |
| ITGA2 | 0 | 3.04 | 2.11 | 2.49 |
| ITGA3 | 0 | 2.09 | 0 | 5.22 |
| ITGA7 | 0 | -1.5 | 0 | 0 |
| ITIH1 | 0 | -7.26 | -4.2 | -7.89 |
| ITIH2 | 0 | -4.22 | 0 | -8.21 |
| ITIH3 | 0 | -4.47 | 0 | -6.62 |
| ITLN1 | 0 | -4.02 | 0 | 0 |
| IVD | 0 | -2 | 0 | -2.86 |
| JAM2 | 0 | -2.24 | -2.74 | 0 |
| JUND | 0 | -1.5 | 0 | 0 |
| KANK4 | 0 | -2.41 | -3.45 | -3.2 |
| KCND2 | 0 | 2.74 | 0 | 3.77 |
| KCND3 | 0 | -1.08 | 0 | -2.74 |
| KCNK17 | 0 | -4.06 | -3.49 | -2.53 |
| KCNN2 | 0 | -1.49 | -2.66 | -3.78 |
| KCTD1 | 0 | 1.19 | 0 | 2.01 |
| KCTD15 | 0 | 1.51 | 0 | 0 |
| KDELR3 | 0 | 2.67 | 1.28 | 2.43 |
| KDM5B | 0 | 1.56 | 1.08 | 1.12 |
| KIAA0101 | 0 | 3.38 | 3.36 | 3.81 |
| KIAA1539 | 0 | 1.05 | 0 | 0 |
| KIAA1549 | 0 | 2.23 | 0 | 1.7 |
| KIAA1609 | 0 | 1.19 | 0 | 2.39 |
| KIAA1683 | 0 | -1.7 | 0 | 0 |
| KIF14 | 0 | 3.16 | 1.93 | 3.34 |
| KIF15 | 0 | 1.78 | 0 | 4.61 |
| KIF23 | 0 | 3.56 | 1.02 | 3.8 |
| KIF24 | 0 | 1.76 | 0 | 2.23 |
| KIF26B | 0 | 3.02 | 0 | 1.6 |
| KIF3C | 0 | 1.98 | 0 | 1.54 |
| KIF4A | 0 | 3.18 | 2.67 | 4.57 |
| KIFC1 | 0 | 3.35 | 0 | 4.56 |
| KLB | 0 | -3.97 | 0 | -6.12 |
| KLF4 | 0 | -1.81 | -3.02 | 1.43 |
| KLF9 | 0 | -1.75 | -1.83 | -1.49 |
| KLRG1 | 0 | -1.27 | 0 | 0 |
| KMO | 0 | -3.17 | -2.99 | -4.11 |
| KNG1 | 0 | -8.42 | -4.18 | -8.91 |
| KNTC1 | 0 | 1.07 | 1.34 | 2.47 |
| KREMEN2 | 0 | 3.12 | 0 | 3.37 |
| KRT14 | 0 | 4.35 | 0 | 8.01 |
| KRT17 | 0 | 4.69 | 4.27 | 6.91 |
| KRT23 | 0 | 2.68 | 0 | 0 |
| KRT42P | 0 | 2.98 | 0 | 0 |
| KRT6B | 0 | 5.57 | 3.03 | 6 |
| KRT86 | 0 | 1.23 | 0 | 0 |
| KRTAP5-10 | 0 | 1.62 | 0 | 0 |
| LAMA1 | 0 | 2.19 | 0 | 2.99 |
| LAMA4 | 0 | 1.29 | 0 | 2.13 |
| LARP1B | 0 | -1.26 | 0 | -1.62 |
| LARP6 | 0 | 1.72 | 0 | 1.73 |
| LBP | 0 | -3.91 | 0 | -6.31 |
| LDHD | 0 | -4.3 | 0 | -4.34 |
| LECT2 | 0 | -7.95 | 0 | -9.91 |
| LEMD1 | 0 | 3.73 | 0 | 4.26 |
| LEPR | 0 | -3.87 | 0 | -4.27 |
| LEPRE1 | 0 | 1.36 | 0 | 0 |
| LEPREL4 | 0 | 1.97 | 0 | 2.67 |
| LGI4 | 0 | -1.9 | -2.94 | -1.29 |
| LGSN | 0 | -3.58 | 0 | -6.04 |
| LIME1 | 0 | -1.34 | 0 | -1.64 |
| LIMK1 | 0 | 1.79 | 1.16 | 2.41 |
| LIN7A | 0 | -1.39 | 0 | -3.35 |
| LINC00152 | 0 | 1.63 | 0 | 0 |
| LINC00261 | 0 | -4.99 | 0 | 0 |
| LINC00340 | 0 | 1.25 | 0 | 0 |
| LIPC | 0 | -3.96 | 0 | -6.1 |
| LIPE | 0 | -1.24 | 0 | 0 |
| LMNB2 | 0 | 2.01 | 0 | 2.79 |
| LOC100130015 | 0 | -1.85 | 0 | 0 |
| LOC100130232 | 0 | -4.86 | 0 | -6.51 |
| LOC100131726 | 0 | -5.91 | 0 | 0 |
| LOC100132724 | 0 | 1.3 | 0 | 0 |
| LOC100134167 | 0 | 3.07 | 0 | 0 |
| LOC100505719 | 0 | -1.13 | 0 | 0 |
| LOC100507055 | 0 | -4.58 | 0 | -5.84 |
| LOC100507203 | 0 | -6.21 | 0 | -7.18 |
| LOC100652805 | 0 | 1.94 | 0 | 0 |
| LOC144571 | 0 | -1.51 | 0 | 0 |
| LOC145837 | 0 | -3.2 | 0 | 0 |
| LOC148709 | 0 | 1.32 | 0 | 0 |
| LOC221442 | 0 | -1.32 | 0 | 0 |
| LOC255167 | 0 | -6.43 | 0 | 0 |
| LOC283352 | 0 | 1.21 | 0 | 1.52 |
| LOC338620 | 0 | 1.47 | 0 | 0 |
| LOC339240 | 0 | -1.75 | 0 | 0 |
| LOC340508 | 0 | 1.47 | 0 | 0 |
| LOC375295 | 0 | 3.47 | 0 | 0 |
| LOC388242 | 0 | 1.61 | 0 | 0 |
| LOC388588 | 0 | -1.46 | 0 | -2.58 |
| LOC541471 | 0 | 1.36 | 0 | 0 |
| LOC553137 | 0 | -2.82 | 0 | -3.75 |
| LOC643988 | 0 | 1.63 | 0 | 3.02 |
| LOC728978 | 0 | 2.65 | 0 | 0 |
| LOC731656 | 0 | -4.31 | 0 | 0 |
| LONRF3 | 0 | -1.4 | 0 | -2.25 |
| LOXL2 | 0 | 3.5 | 0 | 3.02 |
| LPAL2 | 0 | -5.16 | 0 | 0 |
| LRFN4 | 0 | 1.57 | 1.38 | 2.09 |
| LRIG3 | 0 | 1.42 | 0 | 0 |
| LRRC3 | 0 | -1.11 | 0 | -2.75 |
| LRRC49 | 0 | 1.2 | 0 | 1.72 |
| LRRC7 | 0 | -4.03 | 0 | 0 |
| LYPD1 | 0 | 3.66 | 0 | 3.62 |
| LYVE1 | 0 | -2.76 | -3.04 | -3.67 |
| MAGED4B | 0 | 2.56 | 0 | 2.32 |
| MAOA | 0 | -3.22 | 0 | -2.46 |
| MAP2K3 | 0 | -1.12 | 2.38 | 0 |
| MARC1 | 0 | -1.84 | 0 | 0 |
| MARC2 | 0 | -2.17 | 0 | 0 |
| MASP2 | 0 | -6.14 | -3.77 | -7.08 |
| MAST2 | 0 | 1.69 | 0 | 1.31 |
| MATN2 | 0 | -2.27 | -2.4 | 0 |
| MATN3 | 0 | 3.82 | 0 | 4.88 |
| MBL1P | 0 | -2.1 | 0 | 0 |
| MBOAT2 | 0 | 1.73 | 0 | 3.2 |
| MC1R | 0 | 1.21 | 0 | 1.9 |
| MCM2 | 0 | 1.78 | 1.55 | 2.81 |
| MCM4 | 0 | 2.21 | 1.72 | 2.41 |
| MCU | 0 | 1.86 | 0 | 1.55 |
| MDFI | 0 | 1.61 | 0 | 4.4 |
| MED10 | 0 | 1.28 | 0 | 0 |
| MELK | 0 | 4.01 | 2.29 | 5.08 |
| MEMO1 | 0 | 1.02 | 0 | 0 |
| MEX3A | 0 | 2.67 | 0 | 0 |
| MEX3B | 0 | 2.45 | 0 | 2.22 |
| MEX3D | 0 | -1.86 | 1.41 | -2.98 |
| MFAP3L | 0 | -2.24 | 0 | -3.31 |
| MFAP4 | 0 | -3.49 | -3.24 | 0 |
| MFI2 | 0 | 2.71 | 0 | 2.59 |
| MFSD2A | 0 | -2.92 | -2.83 | -4.18 |
| MGAT4C | 0 | -1.93 | 0 | 0 |
| MGC4294 | 0 | 4.64 | 0 | 4.91 |
| MGC4473 | 0 | -1.9 | 0 | 0 |
| MGMT | 0 | -1.35 | -1.33 | -2.21 |
| MGST1 | 0 | -2.33 | 0 | -2.83 |
| MICALCL | 0 | 1.65 | 1.62 | 0 |
| MIR17HG | 0 | -1.6 | 0 | 0 |
| MKI67 | 0 | 2.24 | 1.73 | 2.79 |
| MKL1 | 0 | 1.07 | 1.57 | 1.36 |
| MLF1IP | 0 | 2.19 | 0 | 2.96 |
| MLIP | 0 | -4.8 | 0 | -6.23 |
| MLLT3 | 0 | 1.32 | 0 | 3.12 |
| MLXIPL | 0 | -4.69 | -2.42 | -5.37 |
| MMD | 0 | 1.18 | 0 | 0 |
| MME | 0 | -1.89 | 0 | -2.39 |
| MMP12 | 0 | 3.91 | 0 | 5.49 |
| MMP14 | 0 | 2.17 | 2.36 | 3.02 |
| MMP19 | 0 | -2.07 | 0 | 0 |
| MMP7 | 0 | 2.69 | 0 | 3.95 |
| MND1 | 0 | 3.08 | 0 | 3.48 |
| MNX1 | 0 | 3.88 | 0 | 7.45 |
| MOCS1 | 0 | -2.03 | 0 | -2.35 |
| MORC2 | 0 | 1.46 | 0 | 0 |
| MORN4 | 0 | 1.69 | 0 | 1.05 |
| MPPED1 | 0 | -4.22 | 0 | -6.79 |
| MPST | 0 | -1.32 | 0 | -2.32 |
| MPZL1 | 0 | 1.79 | 0 | 2.1 |
| MSH2 | 0 | 1.05 | 0 | 1.53 |
| MSRA | 0 | -1.63 | 0 | -2.62 |
| MST1 | 0 | -2.88 | 0 | -5.28 |
| MT1A | 0 | -2.11 | -1.88 | -2.26 |
| MT1B | 0 | -2.22 | 0 | -2.42 |
| MT1F | 0 | -3.04 | -2.4 | -3.51 |
| MT1G | 0 | -5.31 | -3.58 | -4.45 |
| MT1H | 0 | -5.27 | -4.14 | -2.47 |
| MT1L | 0 | -2.27 | 0 | 0 |
| MT1M | 0 | -2.38 | -3.99 | -3.8 |
| MT1X | 0 | -2.75 | -2.56 | -3.4 |
| MT2A | 0 | -1.61 | 0 | -1.42 |
| MTHFS | 0 | -1.32 | 0 | -2.3 |
| MTL5 | 0 | 2.29 | 0 | 3.08 |
| MTMR11 | 0 | 1.5 | 0 | 2.87 |
| MTMR14 | 0 | -1.18 | 0 | -2.06 |
| MTSS1 | 0 | -1.53 | 0 | -2.57 |
| MTX2 | 0 | 1.09 | 0 | 0 |
| MUM1L1 | 0 | -2.84 | 0 | -2.77 |
| MUSTN1 | 0 | -2.92 | -4.07 | 0 |
| MUT | 0 | -1.81 | 0 | -2.78 |
| MXRA8 | 0 | 1.77 | 0 | 2.36 |
| MYO19 | 0 | 1.54 | 3.67 | 1.24 |
| MYO1E | 0 | 1.27 | 1.4 | 0 |
| MYOF | 0 | 2.29 | 0 | 3.63 |
| MYRIP | 0 | -3.58 | -2.53 | -4.93 |
| MZT1 | 0 | 1.86 | 0 | 1.99 |
| NACC1 | 0 | 1.01 | 1.5 | 0 |
| NADKD1 | 0 | -2.97 | 0 | -3.58 |
| NAGS | 0 | -1.48 | 0 | -2.92 |
| NAT8 | 0 | -5.33 | 0 | -7.18 |
| NAV1 | 0 | 1.24 | 0 | 2.11 |
| NCEH1 | 0 | 1.54 | 2.12 | 2.08 |
| ND1 | 0 | -1.78 | 0 | 0 |
| ND2 | 0 | -1.78 | 0 | 0 |
| ND3 | 0 | -1.79 | 0 | -1.36 |
| ND4 | 0 | -1.78 | 0 | -1.27 |
| ND4L | 0 | -1.47 | 0 | 0 |
| ND5 | 0 | -1.73 | 0 | -1.07 |
| NDRG2 | 0 | -2.81 | 0 | -3.55 |
| NEK2 | 0 | 2.87 | 0 | 4.28 |
| NEU4 | 0 | -4.59 | 0 | -4.82 |
| NFE2L3 | 0 | 1.71 | 2.15 | 2.96 |
| NMB | 0 | 2 | 0 | 2.21 |
| NME1 | 0 | 1.48 | 0 | 1.41 |
| NMUR1 | 0 | -2.29 | 0 | 0 |
| NOS3 | 0 | -1.02 | -1.76 | 0 |
| NOX4 | 0 | 4.04 | 0 | 4.27 |
| NPAS2 | 0 | 2.4 | 2 | 0 |
| NPM3 | 0 | 1.08 | 0 | 1.23 |
| NPTX1 | 0 | 5.44 | 0 | 5.51 |
| NPW | 0 | -4.49 | 0 | -4.45 |
| NR0B2 | 0 | -4.52 | 0 | -6.12 |
| NR1I2 | 0 | -5.51 | 0 | -5.74 |
| NR1I3 | 0 | -6.47 | 0 | -7.19 |
| NR5A2 | 0 | -1.81 | 0 | -2.61 |
| NRG1 | 0 | -2.51 | 0 | 0 |
| NRGN | 0 | -1.45 | -1.34 | 0 |
| NRP2 | 0 | 1.03 | 0 | 1.1 |
| NTF3 | 0 | -1.59 | -2.58 | -2.18 |
| NTM | 0 | 4.66 | 0 | 5.05 |
| NTRK2 | 0 | -2.66 | -3.3 | 0 |
| NTRK3 | 0 | -4.09 | -2.7 | 0 |
| NUAK1 | 0 | 1.61 | 0 | 0 |
| NUF2 | 0 | 2.93 | 2.86 | 4.49 |
| NUSAP1 | 0 | 1.98 | 2.29 | 2.67 |
| NXPH4 | 0 | 4.19 | 0 | 3.04 |
| OAF | 0 | -2.18 | -1.39 | -2.5 |
| OBFC2B | 0 | 1.86 | 0 | 1.33 |
| ODZ1 | 0 | -4.19 | 0 | -4.26 |
| OGDHL | 0 | -4.99 | 0 | -8.43 |
| OGN | 0 | -3.58 | -5.2 | 0 |
| OIP5 | 0 | 3.18 | 2.85 | 3.75 |
| OLFML2B | 0 | 1.58 | 0 | 2.88 |
| ORM1 | 0 | -6.37 | 0 | -8.13 |
| ORM2 | 0 | -6.22 | 0 | -8.49 |
| OSBPL10 | 0 | 1.2 | 0 | 2.72 |
| OSCP1 | 0 | 1.64 | 0 | 0 |
| OSGIN1 | 0 | -2.39 | 0 | -3.12 |
| OSTalpha | 0 | -5.14 | 0 | -5.48 |
| OXER1 | 0 | -2.42 | 0 | -3.22 |
| OXTR | 0 | 2.07 | 2.99 | 2.69 |
| P4HA2 | 0 | 1.64 | 0 | 1.6 |
| PACS2 | 0 | 1.46 | 0 | 0 |
| PACSIN1 | 0 | -1.42 | 0 | 0 |
| PADI2 | 0 | 2.07 | 0 | 2.94 |
| PAFAH1B3 | 0 | 1.39 | 2.05 | 1.98 |
| PAIP2B | 0 | -2.53 | 0 | -2.44 |
| PALLD | 0 | 1.34 | 0 | 2.34 |
| PALMD | 0 | -2.29 | 0 | -2.04 |
| PANK1 | 0 | -2.48 | 0 | -3.99 |
| PAQR4 | 0 | 2.12 | 0 | 2.83 |
| PCDH7 | 0 | 1.88 | 0 | 3.61 |
| PCDHB2 | 0 | 3.47 | 0 | 3.17 |
| PCNA | 0 | 1.59 | 0 | 1.72 |
| PCSK6 | 0 | -2.27 | 0 | -4.01 |
| PCYT2 | 0 | -1.31 | 0 | -1.95 |
| PDE2A | 0 | -1.71 | -2.34 | -2.15 |
| PDE3B | 0 | -1.6 | 0 | -2.25 |
| PDE4D | 0 | -1.28 | 0 | 2.21 |
| PDK4 | 0 | -3.06 | -3.05 | -3.59 |
| PDLIM7 | 0 | 1.68 | 0 | 2.1 |
| PDPN | 0 | 1.99 | 0 | 5.46 |
| PDZD11 | 0 | 1.5 | 0 | 1.11 |
| PDZD2 | 0 | 1.96 | 0 | 0 |
| PDZK1 | 0 | -2.27 | 0 | -4.2 |
| PDZK1IP1 | 0 | 2.44 | 0 | 3.61 |
| PDZRN4 | 0 | -2.98 | -3.82 | 0 |
| PEBP1 | 0 | -2 | 0 | -2.86 |
| PECR | 0 | -2.29 | 0 | -3.76 |
| PFKFB1 | 0 | -3.4 | 0 | -5.39 |
| PGA3 | 0 | -4.03 | 0 | -5.19 |
| PGM2L1 | 0 | 1.77 | 0 | 1.62 |
| PGM5 | 0 | -2.87 | -3.87 | 0 |
| PHLDA2 | 0 | 2.25 | 2.08 | 3.26 |
| PHLDA3 | 0 | 1.58 | 0 | 1.66 |
| PHTF1 | 0 | 1.21 | 0 | 0 |
| PHYHD1 | 0 | -2.34 | -2.57 | -3.61 |
| PI16 | 0 | -4.15 | -5.49 | 0 |
| PI3 | 0 | 2.26 | 0 | 2.54 |
| PIF1 | 0 | 1.66 | 0 | 2.99 |
| PIGZ | 0 | 1.14 | 0 | 1.69 |
| PIK3R1 | 0 | -1.43 | -1.67 | -1.5 |
| PIK3R3 | 0 | -1.64 | -2.07 | 1.28 |
| PKD2L1 | 0 | 2.1 | 0 | 0 |
| PKLR | 0 | -3.43 | -2.43 | -5.8 |
| PKM2 | 0 | 2.24 | 0 | 4.33 |
| PKMYT1 | 0 | 1.36 | 5.63 | 2.65 |
| PLA2G5 | 0 | -2.37 | 0 | 0 |
| PLAC8 | 0 | -2.06 | 0 | 0 |
| PLAU | 0 | 2.53 | 0 | 3.79 |
| PLCXD3 | 0 | -2.71 | 0 | -2.22 |
| PLD1 | 0 | -1.04 | 0 | -1.37 |
| PLEK2 | 0 | 2.08 | 2.97 | 0 |
| PLG | 0 | -9.24 | 0 | -9.27 |
| PLIN4 | 0 | -3.71 | 0 | -4.52 |
| PLIN5 | 0 | -3.21 | 0 | -3.59 |
| PLP1 | 0 | -2.68 | -3.01 | 0 |
| PLP2 | 0 | 1.82 | 0 | 3.58 |
| PLXNA1 | 0 | 1.45 | 0 | 2.12 |
| PLXNA3 | 0 | 1.19 | 0 | 1.75 |
| PMEPA1 | 0 | 2.07 | 2.32 | 3.55 |
| PML | 0 | 1.8 | 0 | 1.59 |
| PNPLA3 | 0 | -2.23 | 0 | -4.87 |
| PNPLA7 | 0 | -1.71 | 0 | -1.88 |
| PODNL1 | 0 | 2.71 | 0 | 4.75 |
| PON1 | 0 | -5.25 | 0 | -7.78 |
| PON3 | 0 | -4.67 | 0 | -5.91 |
| POR | 0 | -1.46 | 0 | -2.79 |
| POSTN | 0 | 5.79 | 0 | 3.35 |
| PPAP2B | 0 | -1.62 | 0 | -1.98 |
| PPEF1 | 0 | 3.36 | 2.08 | 3.39 |
| PPIAL4A | 0 | 1.52 | 0 | 0 |
| PPIC | 0 | 1.26 | 0 | 0 |
| PPP1R13L | 0 | 1.37 | 1.93 | 1.92 |
| PPP1R14B | 0 | 1.78 | 1.27 | 1.48 |
| PPP1R14C | 0 | 3.02 | 0 | 4.07 |
| PPP1R3C | 0 | -1.65 | 0 | -2.6 |
| PRAP1 | 0 | -3.06 | 0 | -3.99 |
| PRC1 | 0 | 1.9 | 0 | 3.76 |
| PROC | 0 | -5.13 | 0 | -6.84 |
| PRODH2 | 0 | -7 | -4.04 | -7.98 |
| PROS1 | 0 | -2.21 | 0 | -3.36 |
| PROSC | 0 | -1.06 | 0 | 0 |
| PROZ | 0 | -6.32 | -4.13 | -8 |
| PRTFDC1 | 0 | 1.65 | 0 | 2.21 |
| PSAT1 | 0 | -2.53 | 0 | -3.09 |
| PSMC3IP | 0 | 2.12 | 0 | 2.29 |
| PSMD10 | 0 | 1.15 | 0 | 0 |
| PTGDR | 0 | -1.37 | 0 | 0 |
| PTGDS | 0 | -3.1 | 0 | 0 |
| PTGFRN | 0 | 2.29 | 1.23 | 2.38 |
| PTGR1 | 0 | -2.45 | 0 | -2.82 |
| PTH1R | 0 | -2.48 | -2.49 | -3.84 |
| PTHLH | 0 | 2.87 | 0 | 2.34 |
| PTMS | 0 | -1.16 | 0 | 0 |
| PTPRB | 0 | -1.52 | 0 | 0 |
| PTPRU | 0 | 1.5 | 0 | 0 |
| PTTG1 | 0 | 2.12 | 2.11 | 4.11 |
| PTTG2 | 0 | 1.35 | 0 | 3.21 |
| PVRL4 | 0 | 2.63 | 0 | 5.01 |
| PVT1 | 0 | 1.9 | 0 | 3.94 |
| PYCR1 | 0 | 2.38 | 0 | 4.17 |
| PYGB | 0 | 1.36 | 0 | 2.78 |
| PYHIN1 | 0 | -1.4 | 0 | 0 |
| QPRT | 0 | -2.16 | 0 | -2.21 |
| RAB17 | 0 | -2.05 | 0 | -3.9 |
| RAB26 | 0 | -3.2 | 0 | -2.64 |
| RAB34 | 0 | 1.38 | 0 | 2.27 |
| RAB37 | 0 | -1.95 | 0 | -1.12 |
| RAB38 | 0 | 1.43 | 0 | 0 |
| RAB3B | 0 | 3.17 | 3.11 | 2.75 |
| RAB42 | 0 | 2.86 | 0 | 0 |
| RACGAP1 | 0 | 2.26 | 0 | 3.07 |
| RAD18 | 0 | 1.37 | 0 | 1.16 |
| RAD51AP1 | 0 | 2.71 | 2.35 | 3.43 |
| RAI14 | 0 | 1.54 | 0 | 0 |
| RANBP3L | 0 | -4.79 | 0 | -4.53 |
| RARRES2 | 0 | -2.25 | 0 | -2.96 |
| RASAL2 | 0 | 1.38 | 2.53 | 1.46 |
| RASGRP2 | 0 | -1.8 | -1.53 | 0 |
| RASL11A | 0 | -1.57 | 0 | 0 |
| RBM3 | 0 | 1.29 | 0 | 1.08 |
| RBP4 | 0 | -7.46 | -4.47 | -7.78 |
| RCAN2 | 0 | -2.3 | 0 | 0 |
| RCL1 | 0 | -2.23 | -2.19 | -2.72 |
| RDH12 | 0 | -2.5 | 0 | -3.3 |
| RDH5 | 0 | -2.6 | 0 | -2.45 |
| RECQL4 | 0 | 1.91 | 0 | 2.81 |
| REEP6 | 0 | -3.42 | 0 | -5.08 |
| RELL1 | 0 | 1.84 | 0 | 1.06 |
| RERGL | 0 | -3.4 | -4.52 | 0 |
| RFC4 | 0 | 1.43 | 0 | 1.88 |
| RGS7BP | 0 | -2.62 | -2.49 | 0 |
| RHBDF2 | 0 | 1.53 | 0 | 2.11 |
| RHBDL2 | 0 | 2.65 | 0 | 3.54 |
| RHEB | 0 | -1.39 | 0 | 0 |
| RHOV | 0 | 3.04 | 2.39 | 1.96 |
| RHPN1 | 0 | 1.42 | 0 | 0 |
| RIBC2 | 0 | 2.62 | 0 | 3.24 |
| RIMKLA | 0 | 2.48 | 0 | 2.47 |
| RIPK2 | 0 | 1.39 | 1.37 | 1.65 |
| RMI2 | 0 | 2.24 | 2.75 | 3.2 |
| RMND5A | 0 | -1.12 | 0 | -1.03 |
| RN5-8S1 | 0 | -1.69 | 0 | 0 |
| RNF112 | 0 | -1.34 | 0 | 0 |
| RNF215 | 0 | 1.66 | 0 | 0 |
| RNFT2 | 0 | 1.69 | 0 | 1.77 |
| RNU2-2 | 0 | -1.91 | 0 | 0 |
| RORC | 0 | -3.13 | 0 | -4.35 |
| RRM2 | 0 | 2.66 | 2.82 | 3.79 |
| RTP3 | 0 | -7.54 | 0 | -7.83 |
| RUNX1 | 0 | 1.62 | 1.65 | 2.13 |
| RXRA | 0 | -1.68 | -1.21 | -2.06 |
| S100A10 | 0 | 1.49 | 0 | 2.89 |
| S100A11 | 0 | 1.93 | 0 | 3.66 |
| S100A12 | 0 | -2.76 | 0 | 0 |
| S100A16 | 0 | 1.54 | 0 | 1.14 |
| S100A2 | 0 | 2.29 | 0 | 4.64 |
| S100A3 | 0 | 3.22 | 0 | 3.38 |
| S100A6 | 0 | 2.03 | 0 | 4.54 |
| S1PR1 | 0 | -2.08 | -1.76 | 0 |
| SAA1 | 0 | -3.61 | -5.38 | -4.86 |
| SAA2 | 0 | -3.75 | -5.57 | -5.07 |
| SAA4 | 0 | -6.41 | -4.88 | -8.86 |
| SAC3D1 | 0 | 1.18 | 0 | 0 |
| SARDH | 0 | -3.73 | 0 | -5.42 |
| SAT2 | 0 | -1.66 | 0 | -2.24 |
| SC5DL | 0 | -1.3 | 0 | -1.97 |
| SCARA5 | 0 | -4.12 | -4.45 | -1.01 |
| SCARB1 | 0 | -1.77 | 0 | -2.2 |
| SCARNA12 | 0 | -1.52 | 0 | 0 |
| SCARNA16 | 0 | -1.3 | 0 | 0 |
| SCARNA17 | 0 | -2.29 | 0 | 0 |
| SCARNA2 | 0 | -1.67 | 0 | 0 |
| SCARNA9 | 0 | -1.68 | 0 | 0 |
| SCGN | 0 | -3.15 | -3.3 | -3 |
| SCML4 | 0 | -1.48 | 0 | 0 |
| SCN7A | 0 | -3.87 | 0 | -2.32 |
| SCN9A | 0 | -1.49 | 0 | -2.48 |
| SCP2 | 0 | -1.35 | 0 | -4.12 |
| SDHD | 0 | -1.03 | 0 | -1.22 |
| SDS | 0 | -2.26 | -3.63 | -4.55 |
| SELENBP1 | 0 | -3.11 | -2.02 | -2.88 |
| SEMA3C | 0 | 2.48 | 0 | 5.91 |
| SEMA3D | 0 | -1.73 | 0 | 0 |
| SEMA3F | 0 | 1.41 | 0 | 1.95 |
| SEMA3G | 0 | -2.49 | 0 | 0 |
| SEMA4F | 0 | 2.03 | 0 | 1.43 |
| SEPHS2 | 0 | -1.27 | 0 | -2 |
| SERPINA1 | 0 | -2.53 | 0 | -4.76 |
| SERPINC1 | 0 | -9.37 | 0 | -9.62 |
| SERPINF1 | 0 | -2.45 | 0 | -2.3 |
| SERPING1 | 0 | -2.23 | 0 | -3.05 |
| SERPINH1 | 0 | 1.66 | 0 | 2.18 |
| SFN | 0 | 5.19 | 4.87 | 7 |
| SFRP4 | 0 | 3.28 | 0 | 4.7 |
| SFRP5 | 0 | -2.78 | 0 | -3.39 |
| SFXN3 | 0 | 1.16 | 0 | 1.81 |
| SFXN5 | 0 | -1.23 | 0 | -2.05 |
| SGK2 | 0 | -1.9 | 0 | -3.09 |
| SGK223 | 0 | 1.02 | 0 | 2.2 |
| SGOL2 | 0 | 1.53 | 0 | 2.76 |
| SH3BGRL2 | 0 | -1.68 | 0 | -2.03 |
| SH3D21 | 0 | 1.83 | 0 | 2.53 |
| SH3PXD2B | 0 | 1.43 | 0 | 2.32 |
| SHBG | 0 | -5.07 | 0 | -6.26 |
| SHD | 0 | -1.54 | 0 | -2.66 |
| SHPK | 0 | -1.01 | 0 | -1.38 |
| SIK2 | 0 | -1.37 | 0 | -1.89 |
| SIRT5 | 0 | -1.12 | 0 | -1.66 |
| SKA2 | 0 | 1.18 | 0 | 1.33 |
| SKA3 | 0 | 2.46 | 1.89 | 3.31 |
| SLC12A8 | 0 | 2.66 | 0 | 1.28 |
| SLC13A5 | 0 | -6.71 | -4.49 | -7.38 |
| SLC16A3 | 0 | 2.16 | 1.64 | 3.9 |
| SLC17A4 | 0 | -3.89 | 0 | -5.05 |
| SLC19A3 | 0 | -2.42 | 0 | -3.26 |
| SLC22A10 | 0 | -4.02 | 0 | -4.21 |
| SLC22A15 | 0 | 1.43 | 1.49 | 2.53 |
| SLC22A4 | 0 | 1.47 | 0 | 1.69 |
| SLC22A7 | 0 | -4.97 | -4.15 | -7.17 |
| SLC25A10 | 0 | -2.14 | 0 | -2.42 |
| SLC25A18 | 0 | -5.57 | -3.79 | -7.24 |
| SLC25A24 | 0 | 1.65 | 0 | 2.67 |
| SLC25A27 | 0 | -1.85 | 0 | -1.54 |
| SLC25A33 | 0 | -1.32 | 0 | -1.13 |
| SLC25A42 | 0 | -1.75 | 0 | -3.12 |
| SLC25A47 | 0 | -4.6 | -3.44 | -5.19 |
| SLC26A2 | 0 | 1.32 | 0 | 1.95 |
| SLC27A2 | 0 | -4.23 | -3.04 | -4.7 |
| SLC27A5 | 0 | -5.33 | -3.58 | -6.19 |
| SLC28A1 | 0 | -3.73 | 0 | -4.87 |
| SLC2A1 | 0 | 2.32 | 0 | 5.74 |
| SLC2A2 | 0 | -7.23 | 0 | -8.72 |
| SLC2A9 | 0 | -1.46 | 0 | -2.26 |
| SLC30A10 | 0 | -2.44 | 0 | -6.27 |
| SLC30A6 | 0 | 1.85 | 3.82 | 1 |
| SLC35E3 | 0 | 1.24 | 0 | 0 |
| SLC36A1 | 0 | 1.28 | 0 | 1.8 |
| SLC37A4 | 0 | -1.51 | 0 | -2.53 |
| SLC38A3 | 0 | -3.9 | 0 | -5.68 |
| SLC38A6 | 0 | 1.22 | 0 | 1.35 |
| SLC39A10 | 0 | 1.61 | 0 | 1.64 |
| SLC39A5 | 0 | -5.4 | 0 | -5.12 |
| SLC39A6 | 0 | 1.41 | 0 | 0 |
| SLC4A3 | 0 | 3.31 | 0 | 1.76 |
| SLC4A4 | 0 | -1.49 | 0 | -2.48 |
| SLC5A1 | 0 | -2.71 | 0 | 0 |
| SLC6A1 | 0 | -3.05 | 0 | -6.41 |
| SLC6A10P | 0 | 2.02 | 0 | 0 |
| SLC6A13 | 0 | -4.39 | 0 | -5.47 |
| SLC6A16 | 0 | -1.38 | -2.13 | -2.81 |
| SLC6A19 | 0 | -2.27 | 0 | 0 |
| SLC6A6 | 0 | 1.65 | 0 | 3.1 |
| SLC6A8 | 0 | 2.71 | 0 | 3.89 |
| SLC7A1 | 0 | 2.3 | 0 | 3.73 |
| SLC7A11 | 0 | 3.1 | 0 | 6.6 |
| SLC9A3R2 | 0 | -2.07 | 0 | -2.32 |
| SLC9A7P1 | 0 | 1.31 | 0 | 0 |
| SLCO1B1 | 0 | -6.91 | -3.02 | -7.75 |
| SLED1 | 0 | -2.4 | 0 | 0 |
| SLIT3 | 0 | 2.09 | 0 | 2.22 |
| SLITRK6 | 0 | -2.78 | 0 | 0 |
| SLMO1 | 0 | 1.77 | 0 | 2.26 |
| SMC4 | 0 | 1.33 | 1.3 | 1.77 |
| SMG9 | 0 | 1.03 | 1.41 | 1.78 |
| SMOC1 | 0 | -3.11 | 0 | -3.63 |
| SMPD3 | 0 | -2.18 | 0 | 0 |
| SMPX | 0 | 3.91 | 0 | 0 |
| SMS | 0 | 1.18 | 0 | 1.06 |
| SMYD3 | 0 | 1.72 | 0 | 1.16 |
| SNRPD1 | 0 | 1.15 | 0 | 1.55 |
| SNRPE | 0 | 1.42 | 0 | 0 |
| SNX7 | 0 | 1.22 | 0 | 0 |
| SOCS2 | 0 | -1.93 | 0 | 0 |
| SORL1 | 0 | -1.44 | 0 | -2.05 |
| SPC24 | 0 | 2.97 | 0 | 3.37 |
| SPC25 | 0 | 3.54 | 0 | 4.48 |
| SPECC1 | 0 | 2.37 | 0 | 1.94 |
| SPEG | 0 | 2.5 | -2.83 | 0 |
| SPINK1 | 0 | 4.06 | 0 | 4.01 |
| SPINT1 | 0 | 1.74 | 1.99 | 3.73 |
| SPINT2 | 0 | 1.46 | 2 | 4.09 |
| SPIRE1 | 0 | 1.06 | 0 | 0 |
| SPNS3 | 0 | -1.12 | 0 | 0 |
| SPOCD1 | 0 | 5.35 | 0 | 6.28 |
| SPOCK1 | 0 | 4.26 | 0 | 7.77 |
| SPON2 | 0 | 1.56 | 0 | 0 |
| SPP2 | 0 | -8.2 | 0 | -8.41 |
| SPRY1 | 0 | -1.02 | 0 | 1.26 |
| SPTBN5 | 0 | 2.48 | 0 | 0 |
| SRD5A2 | 0 | -5.02 | -3.75 | -7.76 |
| SRL | 0 | -3.11 | 0 | 0 |
| SRPX2 | 0 | 2.11 | 0 | 1.82 |
| SRRM3 | 0 | 2.52 | 0 | -2.24 |
| SSTR1 | 0 | -4.2 | 0 | -3.34 |
| SSTR2 | 0 | -1.55 | 0 | 0 |
| STARD10 | 0 | -2.01 | 0 | -2.39 |
| STAT4 | 0 | -1.75 | 0 | 0 |
| STBD1 | 0 | -1.29 | 0 | -3.18 |
| STC2 | 0 | 2.2 | 0 | 1.94 |
| STEAP4 | 0 | -3.93 | 0 | 0 |
| STIL | 0 | 1.6 | 1.96 | 3.08 |
| STK39 | 0 | 1.94 | 0 | 4.08 |
| STRA6 | 0 | 2.91 | 0 | 4.34 |
| SUCLG2 | 0 | -1.56 | 0 | -1.86 |
| SULF1 | 0 | 3 | 0 | 5.3 |
| SULT1A2 | 0 | -1.55 | 0 | -3.6 |
| SULT2A1 | 0 | -7.02 | -5.26 | -8.47 |
| SVEP1 | 0 | -1.61 | 0 | 0 |
| SYT7 | 0 | -2.86 | 0 | -4.79 |
| TANC2 | 0 | 2.58 | 0 | 2.51 |
| TAT | 0 | -3.14 | -5.17 | -6.06 |
| TBX3 | 0 | -1.66 | 0 | -2.17 |
| TCL1A | 0 | -1.92 | 0 | 0 |
| TCP10L | 0 | -1.23 | 0 | -3.68 |
| TDG | 0 | 2.22 | 0 | 0 |
| TDRD10 | 0 | -1.87 | 0 | -2.16 |
| TF | 0 | -8.42 | -4.23 | -9.07 |
| TFCP2L1 | 0 | 4.73 | 0 | 4.18 |
| TFF3 | 0 | -2.43 | 0 | 0 |
| TFR2 | 0 | -2.17 | 0 | -2.42 |
| TGFB2 | 0 | 3.11 | 3.66 | 2.79 |
| TGFBR1 | 0 | 1.88 | 1.16 | 0 |
| TGFBR2 | 0 | -1.37 | 0 | 0 |
| TGFBR3 | 0 | -2.11 | -1.76 | -1.37 |
| TIMD4 | 0 | -2.13 | 0 | -3.33 |
| TK1 | 0 | 2.78 | 2.11 | 3.09 |
| TLE4 | 0 | -1.35 | 0 | 0 |
| TM4SF5 | 0 | -4.39 | 0 | -4.71 |
| TMED3 | 0 | 1.05 | 0 | 2.39 |
| TMEM101 | 0 | 1.48 | 0 | 0 |
| TMEM130 | 0 | 4.2 | 0 | 3.96 |
| TMEM132A | 0 | 3.29 | 0 | 3.24 |
| TMEM136 | 0 | 1.4 | 0 | 1.7 |
| TMEM144 | 0 | 1.41 | 0 | 2.42 |
| TMEM150C | 0 | -1.53 | 0 | -2.17 |
| TMEM176A | 0 | -2.03 | 0 | -3.14 |
| TMEM176B | 0 | -1.95 | 0 | -2.68 |
| TMEM185B | 0 | 1.09 | 0 | 1.27 |
| TMEM189 | 0 | 1.55 | 0 | 0 |
| TMEM206 | 0 | 1.4 | 0 | 2.14 |
| TMEM238 | 0 | -1.26 | 0 | 0 |
| TMEM44 | 0 | 1.18 | 0 | 1.15 |
| TMEM52 | 0 | -2.04 | 0 | -2.4 |
| TMEM59L | 0 | 1.54 | 0 | 0 |
| TMEM88 | 0 | -1.9 | -1.8 | 0 |
| TMEM97 | 0 | -1.35 | 0 | 0 |
| TMPRSS6 | 0 | -2.06 | 0 | -5.3 |
| TMSB10 | 0 | 1.25 | 0 | 2.29 |
| TMSB15A | 0 | 2.62 | 0 | 1.55 |
| TMTC1 | 0 | -2.4 | 0 | 0 |
| TNFAIP6 | 0 | 5.09 | 0 | 4.55 |
| TNNT1 | 0 | 5.23 | 0 | 5.09 |
| TNXB | 0 | -3.53 | 0 | 1.11 |
| TOB2P1 | 0 | 2.1 | 0 | 0 |
| TOP2A | 0 | 2.95 | 3.8 | 5.66 |
| TP53I3 | 0 | 1.76 | 0 | 1.27 |
| TPM1 | 0 | 1.47 | 0 | 1.54 |
| TPM4 | 0 | 1.12 | 0 | 2.69 |
| TPPP2 | 0 | -5.36 | 0 | -7.14 |
| TREM2 | 0 | 2.34 | 0 | 1.49 |
| TRIM16L | 0 | 1.05 | 0 | 2.17 |
| TRIM46 | 0 | 2.94 | 0 | 2.27 |
| TRIM47 | 0 | 1.37 | 0 | 1.55 |
| TRIM59 | 0 | 2.04 | 0 | 4.31 |
| TRIM6 | 0 | 2.65 | 0 | 0 |
| TRIM65 | 0 | 1.21 | 1.51 | 1.45 |
| TRIP13 | 0 | 3.5 | 2.7 | 2.41 |
| TROAP | 0 | 2.01 | 5.41 | 1.8 |
| TRPV6 | 0 | -2.53 | 0 | 0 |
| TSPAN15 | 0 | 2.02 | 0 | 2.25 |
| TSPAN7 | 0 | -2.41 | -3.24 | -1.86 |
| TSPO2 | 0 | 1.71 | 0 | 1.74 |
| TST | 0 | -1.79 | 0 | -2.99 |
| TTC39A | 0 | 2.83 | 0 | 3.6 |
| TTC39C | 0 | -2.55 | 0 | -3.48 |
| TTN | 0 | -1.62 | 0 | 0 |
| TTPA | 0 | -5.9 | 0 | -7.42 |
| TTR | 0 | -8.89 | -4.29 | -8.22 |
| TUBB3 | 0 | 4.47 | 0 | 5.47 |
| TUFT1 | 0 | 1.24 | 1.72 | 1.8 |
| TWIST1 | 0 | 2.99 | 0 | 3.43 |
| TYMS | 0 | 3.3 | 2.13 | 2.67 |
| UBAP1L | 0 | -1.16 | 0 | -1.05 |
| UBASH3B | 0 | 1.96 | 2.45 | 1.74 |
| UBD | 0 | 2.12 | 1.75 | 1.73 |
| UBE2C | 0 | 3.22 | 0 | 4.87 |
| UBE2T | 0 | 3.34 | 2.3 | 3.6 |
| UBE2Z | 0 | 1.13 | 1.13 | 1.28 |
| UCK2 | 0 | 1.62 | 1.07 | 1.69 |
| UGT1A6 | 0 | -4.19 | 0 | -4.71 |
| UGT1A8 | 0 | -3.84 | 2.83 | -4.95 |
| UGT2B15 | 0 | -4.71 | 0 | -6.04 |
| UGT2B7 | 0 | -7.21 | -3.08 | -7.75 |
| UHRF1 | 0 | 2.19 | 3.11 | 3.57 |
| ULBP1 | 0 | 1.89 | 0 | 2.34 |
| UNCX | 0 | -1.45 | 0 | 0 |
| UPB1 | 0 | -5.26 | -4.2 | -6.78 |
| UROC1 | 0 | -3.77 | 0 | -7.53 |
| VCAN | 0 | 2.49 | 0 | 4.69 |
| VDAC1 | 0 | 1.14 | 0 | 0 |
| VDR | 0 | 2.53 | 0 | 4.18 |
| VSIG10 | 0 | 1.02 | 0 | 0 |
| VSIG2 | 0 | -1.41 | 0 | 0 |
| VTN | 0 | -5.76 | 0 | -7.64 |
| VWA1 | 0 | -2.29 | 0 | -1.89 |
| VWCE | 0 | -2.81 | 0 | -3.92 |
| WDHD1 | 0 | 1.97 | 4.05 | 3.2 |
| WDR41 | 0 | 1.33 | 0 | 0 |
| WDR62 | 0 | 1.27 | 0 | 1.94 |
| WDYHV1 | 0 | 1.05 | 0 | 0 |
| WHSC1 | 0 | 1.92 | 0 | 1.35 |
| WISP2 | 0 | -2.82 | 0 | 0 |
| WNT2 | 0 | 2.96 | 0 | 1.86 |
| WNT6 | 0 | -1.88 | 0 | 0 |
| XLOC_002070 | 0 | -1.03 | 0 | 0 |
| XLOC_004620 | 0 | 2.3 | 0 | 0 |
| XLOC_005285 | 0 | -2.16 | 0 | 0 |
| XLOC_009194 | 0 | -3.3 | 0 | 0 |
| XLOC_009582 | 0 | 2.72 | 0 | 0 |
| XLOC_009765 | 0 | -1.4 | 0 | 0 |
| XLOC_011950 | 0 | 1.6 | 0 | 0 |
| XLOC_012568 | 0 | 3.66 | 0 | 0 |
| XLOC_014327 | 0 | 3.17 | 0 | 0 |
| XPOT | 0 | 1.04 | 0 | 0 |
| XXYLT1 | 0 | 1.16 | 0 | 1.77 |
| ZBTB16 | 0 | -1.98 | -2.59 | -2.71 |
| ZBTB41 | 0 | 1.88 | 0 | 0 |
| ZDHHC11 | 0 | -1.25 | 0 | -2.18 |
| ZDHHC24 | 0 | 1.16 | 1.13 | 1.28 |
| ZEB1-AS1 | 0 | 1.61 | 0 | 0 |
| ZFHX4 | 0 | -1.36 | 0 | -1.69 |
| ZFP36 | 0 | -1.6 | -1.43 | 0 |
| ZG16 | 0 | -7.33 | 0 | -7.48 |
| ZGPAT | 0 | -1.95 | 0 | -2.81 |
| ZMAT1 | 0 | -1.46 | 0 | -1.15 |
| ZNF239 | 0 | 1.57 | 0 | 1.86 |
| ZNF469 | 0 | 2.2 | 0 | 2.39 |
| ZNF532 | 0 | 1.2 | 0 | 2.01 |
| ZNF697 | 0 | 1.15 | 0 | 0 |
| ZNF761 | 0 | 2.31 | 0 | 1.22 |
| ZSCAN20 | 0 | 1.82 | 0 | 1.41 |
| ZWINT | 0 | 2.46 | 1.85 | 3.27 |
| AADAT | 0 | 0 | -1.86 | -3.93 |
| ABCA9 | 0 | 0 | -1.89 | 0 |
| ABCC8 | 0 | 0 | -2.22 | 0 |
| ABCD1 | 0 | 0 | 2.24 | 1.05 |
| ABHD16B | 0 | 0 | 2.64 | 0 |
| ABI3BP | 0 | 0 | -2.19 | 0 |
| ACKR1 | 0 | 0 | -4.9 | 0 |
| ACKR3 | 0 | 0 | -1.84 | 0 |
| ACTA2 | 0 | 0 | -1.38 | 2.34 |
| ACTB | 0 | 0 | 1.07 | 0 |
| ACTC1 | 0 | 0 | -5.01 | 0 |
| ACTL10 | 0 | 0 | 2.4 | 0 |
| ACVR1B | 0 | 0 | 2.08 | 0 |
| ACVRL1 | 0 | 0 | -1.78 | 0 |
| ACYP2 | 0 | 0 | -1.01 | -1.39 |
| ADAM10 | 0 | 0 | 1.43 | 1.05 |
| ADAM8 | 0 | 0 | 3.37 | 1.83 |
| ADAM9 | 0 | 0 | 1.66 | 1.44 |
| ADAMTS4 | 0 | 0 | -2.99 | 0 |
| ADAMTS8 | 0 | 0 | -4.36 | 0 |
| ADAMTSL4 | 0 | 0 | 2.47 | 0 |
| ADAP1 | 0 | 0 | 2.72 | 1.45 |
| ADARB1 | 0 | 0 | -1.67 | 0 |
| ADAT3 | 0 | 0 | 1.27 | 0 |
| ADCY4 | 0 | 0 | -1.51 | 0 |
| ADGRB1 | 0 | 0 | 6.27 | 0 |
| ADGRB3 | 0 | 0 | -2.88 | 0 |
| ADGRG5 | 0 | 0 | 3.16 | 0 |
| ADGRL3 | 0 | 0 | -3.58 | 0 |
| ADIRF | 0 | 0 | -3.04 | 0 |
| ADORA2B | 0 | 0 | 2.54 | 3.57 |
| ADTRP | 0 | 0 | 1.7 | 0 |
| AIFM2 | 0 | 0 | 1.16 | -2.33 |
| AKAP12 | 0 | 0 | -2.07 | 0 |
| AKAP4 | 0 | 0 | 2.25 | 0 |
| AKAP8L | 0 | 0 | 1.02 | 0 |
| AKIRIN1 | 0 | 0 | 2.55 | 0 |
| AKR1E2 | 0 | 0 | 2.42 | 1.69 |
| AKT2 | 0 | 0 | 1.58 | 0 |
| ALDH3B1 | 0 | 0 | 3.75 | 1.54 |
| ALG10B | 0 | 0 | 2.1 | 0 |
| AMDHD2 | 0 | 0 | 1.44 | 0 |
| AMHR2 | 0 | 0 | -1.83 | 0 |
| AMY2A | 0 | 0 | -5.93 | 0 |
| ANGPT1 | 0 | 0 | -2.75 | 0 |
| ANGPTL7 | 0 | 0 | -5 | 0 |
| ANKDD1A | 0 | 0 | -1.93 | 0 |
| ANKHD1-EIF4EBP3 | 0 | 0 | 1.19 | 0 |
| ANKRD1 | 0 | 0 | 3.1 | 0 |
| ANKRD35 | 0 | 0 | -2.54 | 0 |
| ANKS1B | 0 | 0 | -1.74 | 0 |
| ANXA13 | 0 | 0 | 2.92 | 0 |
| AOC3 | 0 | 0 | -2.6 | 0 |
| AP1S2 | 0 | 0 | -1.21 | 0 |
| AP3M1 | 0 | 0 | 7.56 | 0 |
| APBB3 | 0 | 0 | -1.39 | 0 |
| APOBEC3B | 0 | 0 | 2.23 | 0 |
| APOD | 0 | 0 | -5.36 | 0 |
| APOLD1 | 0 | 0 | -2.97 | 0 |
| AQP8 | 0 | 0 | -5.9 | 0 |
| ARFGEF2 | 0 | 0 | 1.67 | 0 |
| ARFIP1 | 0 | 0 | 1.47 | 0 |
| ARHGAP15 | 0 | 0 | -2.09 | 0 |
| ARHGAP27 | 0 | 0 | 3.29 | 1.95 |
| ARHGAP30 | 0 | 0 | 1.65 | 0 |
| ARHGEF10 | 0 | 0 | -1.43 | 1.87 |
| ARHGEF11 | 0 | 0 | 1.06 | 0 |
| ARHGEF18 | 0 | 0 | 1.16 | 0 |
| ARHGEF35 | 0 | 0 | 2.44 | 0 |
| ARHGEF37 | 0 | 0 | -2.17 | 1.23 |
| ARHGEF39 | 0 | 0 | 2.81 | 0 |
| ARHGEF5 | 0 | 0 | 1.85 | 0 |
| ARL14 | 0 | 0 | 2.2 | 4.73 |
| ARL4C | 0 | 0 | 1.54 | 1.71 |
| ARMC7 | 0 | 0 | 1.21 | 0 |
| ARMCX6 | 0 | 0 | 1.06 | 0 |
| ARRDC1 | 0 | 0 | 1.3 | 0 |
| ARSA | 0 | 0 | 1.23 | 0 |
| ARSH | 0 | 0 | 5.3 | 0 |
| ART3 | 0 | 0 | -3.49 | 0 |
| ASAP1 | 0 | 0 | 1.11 | 1.41 |
| ASB4 | 0 | 0 | -1.27 | -3.95 |
| ASB7 | 0 | 0 | 2.37 | 0 |
| ASF1B | 0 | 0 | 3.7 | 1.93 |
| ASH1L | 0 | 0 | 3.43 | 0 |
| ASPHD1 | 0 | 0 | 2.44 | 3.84 |
| ASPHD2 | 0 | 0 | 2.45 | 1.64 |
| ATAD2 | 0 | 0 | 1.23 | 1.04 |
| ATF2 | 0 | 0 | 1.13 | 0 |
| ATG4D | 0 | 0 | 2.05 | 0 |
| ATP1A1 | 0 | 0 | 1.7 | 1.16 |
| ATP1A2 | 0 | 0 | -5.25 | 0 |
| ATP6V1G2 | 0 | 0 | -1.13 | 0 |
| ATP8B1 | 0 | 0 | 1.42 | 0 |
| ATP8B2 | 0 | 0 | -1.45 | 0 |
| AURKA | 0 | 0 | 1.99 | 2.56 |
| AURKB | 0 | 0 | 2.24 | 3.31 |
| AVL9 | 0 | 0 | 1.56 | 0 |
| AVPI1 | 0 | 0 | -1.34 | 0 |
| AXIN1 | 0 | 0 | 1.03 | 0 |
| AZIN2 | 0 | 0 | 1.14 | 0 |
| B3GNT3 | 0 | 0 | 1.72 | 4.43 |
| B3GNTL1 | 0 | 0 | 3.52 | 1.32 |
| B4GALT5 | 0 | 0 | 1.05 | 1.27 |
| BAD | 0 | 0 | 1.12 | 0 |
| BAG1 | 0 | 0 | 1.44 | 0 |
| BAIAP2L1 | 0 | 0 | 2.07 | 0 |
| BAK1 | 0 | 0 | 1.4 | 1.78 |
| BATF | 0 | 0 | 2.27 | 2.53 |
| BATF2 | 0 | 0 | 1.73 | 1.22 |
| BAX | 0 | 0 | 1.45 | 0 |
| BAZ1A | 0 | 0 | 1.04 | 0 |
| BCAN | 0 | 0 | -3.1 | 0 |
| BCAS3 | 0 | 0 | -1.11 | 0 |
| BCAS4 | 0 | 0 | 1.6 | 3.29 |
| BCL6B | 0 | 0 | -1.65 | 0 |
| BCR | 0 | 0 | 1.28 | 1.05 |
| BEST1 | 0 | 0 | -1.88 | 0 |
| BEST4 | 0 | 0 | 3.35 | 0 |
| BEX5 | 0 | 0 | -2.41 | 0 |
| BICDL1 | 0 | 0 | 2.34 | 0 |
| BLM | 0 | 0 | 1.83 | 3.27 |
| BOC | 0 | 0 | -2.34 | 0 |
| BORA | 0 | 0 | 1.62 | 2.61 |
| BRAF | 0 | 0 | 2.18 | 0 |
| BROX | 0 | 0 | 2 | 0 |
| BTBD2 | 0 | 0 | 1.27 | 0 |
| BTG2 | 0 | 0 | -2.24 | 1.59 |
| BTN2A2 | 0 | 0 | 1.36 | 1.46 |
| C10orf82 | 0 | 0 | -2.1 | 0 |
| C11orf1 | 0 | 0 | 3.03 | 0 |
| C11orf80 | 0 | 0 | 1.97 | 2.87 |
| C17orf53 | 0 | 0 | 1.91 | 2.43 |
| C19orf48 | 0 | 0 | 1.21 | 1.68 |
| C1orf116 | 0 | 0 | 2.07 | 3.44 |
| C1QTNF7 | 0 | 0 | -1.8 | 0 |
| C2orf15 | 0 | 0 | 3.47 | 0 |
| C2orf40 | 0 | 0 | -5.61 | 0 |
| C2orf88 | 0 | 0 | -1.83 | 0 |
| C3orf70 | 0 | 0 | -2.91 | 1.27 |
| C4orf3 | 0 | 0 | -1.29 | 0 |
| C5orf22 | 0 | 0 | 1.21 | 0 |
| C6orf223 | 0 | 0 | 1.82 | 0 |
| C9orf163 | 0 | 0 | 3.73 | 0 |
| CA4 | 0 | 0 | -2.59 | 0 |
| CA7 | 0 | 0 | 4.02 | 0 |
| CAB39L | 0 | 0 | -1.66 | 0 |
| CADM2 | 0 | 0 | -3.93 | -2.26 |
| CALD1 | 0 | 0 | -3.55 | 0 |
| CALHM2 | 0 | 0 | -1.19 | 1.43 |
| CAPN15 | 0 | 0 | 1.42 | 0 |
| CARD11 | 0 | 0 | 2.23 | 2.35 |
| CASK | 0 | 0 | 1.54 | 1.18 |
| CASP6 | 0 | 0 | 2.11 | 0 |
| CASQ2 | 0 | 0 | -5.44 | 0 |
| CATSPERB | 0 | 0 | 2.95 | 2.69 |
| CAV1 | 0 | 0 | -1.77 | 1.53 |
| CBLC | 0 | 0 | 1.61 | 0 |
| CBLL1 | 0 | 0 | 1.15 | 0 |
| CBWD1 | 0 | 0 | 3.82 | 0 |
| CBX4 | 0 | 0 | 1.02 | 0 |
| CBX7 | 0 | 0 | -1.16 | 0 |
| CCDC138 | 0 | 0 | 1.55 | 0 |
| CCDC148 | 0 | 0 | 3.7 | 0 |
| CCDC181 | 0 | 0 | 2.75 | 0 |
| CCDC184 | 0 | 0 | -1.47 | 0 |
| CCDC186 | 0 | 0 | 1.25 | 0 |
| CCDC3 | 0 | 0 | -2.2 | 0 |
| CCDC33 | 0 | 0 | 1.8 | 0 |
| CCDC50 | 0 | 0 | 4.21 | 0 |
| CCDC6 | 0 | 0 | 1.09 | 1.21 |
| CCDC74A | 0 | 0 | 2.19 | 0 |
| CCDC9 | 0 | 0 | 1.8 | 0 |
| CCL18 | 0 | 0 | 2.56 | 3.75 |
| CCL23 | 0 | 0 | -3.45 | 0 |
| CCL5 | 0 | 0 | 1.39 | 0 |
| CCM2L | 0 | 0 | -1.79 | 0 |
| CCNA2 | 0 | 0 | 2.54 | 4.1 |
| CCNB2 | 0 | 0 | 3.42 | 4.73 |
| CCNF | 0 | 0 | 3.57 | 2.48 |
| CD151 | 0 | 0 | 1.3 | 0 |
| CD28 | 0 | 0 | 6.05 | 0 |
| CD2AP | 0 | 0 | 1.42 | 1.13 |
| CD3E | 0 | 0 | 1.96 | 0 |
| CD93 | 0 | 0 | -1.35 | 0 |
| CDC20 | 0 | 0 | 2.54 | 2.37 |
| CDH13 | 0 | 0 | -2.33 | 2.63 |
| CDH15 | 0 | 0 | -1.71 | 0 |
| CDH24 | 0 | 0 | 2.16 | 0 |
| CDH5 | 0 | 0 | -1.6 | 0 |
| CDHR4 | 0 | 0 | 1.75 | 3.21 |
| CDK16 | 0 | 0 | 3.65 | 1.21 |
| CDK2 | 0 | 0 | 1.94 | 0 |
| CDK7 | 0 | 0 | 1.1 | 0 |
| CDKN2C | 0 | 0 | -1.17 | 1.95 |
| CDS1 | 0 | 0 | 1.94 | 3.04 |
| CDX1 | 0 | 0 | 5.27 | 0 |
| CEL | 0 | 0 | -9.68 | 0 |
| CELA2A | 0 | 0 | -9.71 | 0 |
| CELA2B | 0 | 0 | -8.9 | 0 |
| CELA3A | 0 | 0 | -10.8 | 0 |
| CELA3B | 0 | 0 | -11.1 | 0 |
| CEMIP | 0 | 0 | 3.34 | 0 |
| CENPL | 0 | 0 | 2.61 | 3 |
| CENPU | 0 | 0 | 1.58 | 0 |
| CEP131 | 0 | 0 | 1.39 | 0 |
| CEP72 | 0 | 0 | 1.96 | 0 |
| CERS6 | 0 | 0 | 1.13 | 0 |
| CES5A | 0 | 0 | -1.43 | -6.14 |
| CFAP61 | 0 | 0 | 1.59 | 0 |
| CFD | 0 | 0 | -2.47 | 0 |
| CHAF1A | 0 | 0 | 1.29 | 1.51 |
| CHGA | 0 | 0 | -4.65 | 0 |
| CHTF18 | 0 | 0 | 1.42 | 1.73 |
| CIITA | 0 | 0 | 1.1 | 2.18 |
| CKAP2 | 0 | 0 | 2.62 | 2.55 |
| CKAP5 | 0 | 0 | 1.1 | 1.48 |
| CLDN5 | 0 | 0 | -1.87 | 0 |
| CLDN7 | 0 | 0 | 2.23 | 0 |
| CLDN9 | 0 | 0 | 3.05 | 0 |
| CLEC14A | 0 | 0 | -1.19 | 0 |
| CLEC1A | 0 | 0 | -2.24 | 0 |
| CLEC7A | 0 | 0 | 3.4 | 0 |
| CLIC4 | 0 | 0 | -2.17 | 0 |
| CLK3 | 0 | 0 | 1.3 | 0 |
| CLPS | 0 | 0 | -12.09 | 0 |
| CMTM5 | 0 | 0 | -1.99 | 0 |
| CNBD2 | 0 | 0 | 6.62 | 0 |
| CNKSR1 | 0 | 0 | 2.36 | 3.43 |
| CNKSR2 | 0 | 0 | 7.17 | 0 |
| CNN1 | 0 | 0 | -3.85 | 2.9 |
| CNN2 | 0 | 0 | 2.79 | 1.45 |
| CNNM1 | 0 | 0 | 2.24 | 0 |
| CNNM4 | 0 | 0 | 1.24 | 1.85 |
| CNOT3 | 0 | 0 | 1.09 | 0 |
| CNOT9 | 0 | 0 | 1.4 | 0 |
| CNP | 0 | 0 | -2.65 | 0 |
| CNRIP1 | 0 | 0 | -1.97 | 0 |
| CNTN2 | 0 | 0 | -1.91 | 0 |
| COL11A1 | 0 | 0 | 4.62 | 0 |
| COL21A1 | 0 | 0 | -2.91 | 0 |
| COL4A6 | 0 | 0 | -2.66 | 0 |
| COL9A1 | 0 | 0 | -1.92 | 0 |
| CORO2B | 0 | 0 | -2.45 | 0 |
| CORO7 | 0 | 0 | 1.16 | 0 |
| COX7A1 | 0 | 0 | -1.86 | 0 |
| CPA1 | 0 | 0 | -11.96 | 0 |
| CPA2 | 0 | 0 | -11.28 | 0 |
| CPB1 | 0 | 0 | -10.99 | 0 |
| CPE | 0 | 0 | -2.53 | 0 |
| CPEB3 | 0 | 0 | -1.48 | -2.58 |
| CPED1 | 0 | 0 | -2.44 | 0 |
| CPM | 0 | 0 | 2.14 | 0 |
| CPXM2 | 0 | 0 | -2.12 | 0 |
| CRAT | 0 | 0 | -1.34 | -2 |
| CRB3 | 0 | 0 | 1.29 | 0 |
| CRISPLD2 | 0 | 0 | -1.78 | 0 |
| CRTAP | 0 | 0 | -1.62 | 1.07 |
| CRYAA | 0 | 0 | -3.95 | -6.03 |
| CRYAB | 0 | 0 | -2.82 | 0 |
| CSNK1D | 0 | 0 | 1.09 | 0 |
| CSPG4 | 0 | 0 | -1.95 | 1.66 |
| CSRP1 | 0 | 0 | -1.38 | 0 |
| CSRP2 | 0 | 0 | -1.75 | 0 |
| CT45A3 | 0 | 0 | 3.16 | 0 |
| CT55 | 0 | 0 | -1.44 | 0 |
| CTBP1 | 0 | 0 | 3.64 | 0 |
| CTDP1 | 0 | 0 | 1.58 | 0 |
| CTNNA2 | 0 | 0 | -2.71 | 0 |
| CTNND1 | 0 | 0 | 1.51 | 0 |
| CTRB2 | 0 | 0 | -10.15 | 0 |
| CTRC | 0 | 0 | -10.82 | -1.91 |
| CTRL | 0 | 0 | -4.25 | 0 |
| CTSA | 0 | 0 | 1.01 | 0 |
| CTSF | 0 | 0 | -1.39 | -1.74 |
| CTSG | 0 | 0 | -2.84 | 0 |
| CTSV | 0 | 0 | 3.98 | 0 |
| CUZD1 | 0 | 0 | -3.99 | 1.84 |
| CXCL8 | 0 | 0 | 2.8 | 0 |
| CXorf38 | 0 | 0 | 1.33 | 0 |
| CYBA | 0 | 0 | 1.37 | 1.69 |
| CYCS | 0 | 0 | -1.18 | 0 |
| CYP27B1 | 0 | 0 | 2.08 | 1.93 |
| CYP2C8 | 0 | 0 | -4.03 | -8.69 |
| CYP2U1 | 0 | 0 | -1.31 | 0 |
| CYP3A43 | 0 | 0 | -3.42 | -4.43 |
| CYR61 | 0 | 0 | -1.73 | 0 |
| CYTL1 | 0 | 0 | -2.31 | 1.86 |
| CYYR1 | 0 | 0 | -1.76 | 0 |
| DAAM2 | 0 | 0 | -2.02 | -1.33 |
| DACH1 | 0 | 0 | -2.03 | 0 |
| DACT3 | 0 | 0 | -2.56 | 0 |
| DAXX | 0 | 0 | 1.14 | 0 |
| DBNDD1 | 0 | 0 | 2.5 | 0 |
| DCAF17 | 0 | 0 | 1.57 | 0 |
| DCD | 0 | 0 | -1.55 | 0 |
| DCT | 0 | 0 | -4.4 | 0 |
| DCUN1D3 | 0 | 0 | -1.11 | 0 |
| DDIAS | 0 | 0 | 1.49 | 0 |
| DDR2 | 0 | 0 | -2.17 | 0 |
| DDX19B | 0 | 0 | 1.12 | 0 |
| DDX39A | 0 | 0 | 1.04 | 1.66 |
| DENND1B | 0 | 0 | 5.69 | 0 |
| DENND2A | 0 | 0 | -1.41 | 0 |
| DENND2D | 0 | 0 | 1.47 | 1.13 |
| DEPDC1B | 0 | 0 | 3.81 | 2.83 |
| DHX37 | 0 | 0 | 1.18 | 0 |
| DIP2A | 0 | 0 | 1.64 | 1.09 |
| DIS3L2 | 0 | 0 | 1.01 | 0 |
| DISP2 | 0 | 0 | 1.79 | -2.14 |
| DIXDC1 | 0 | 0 | -1.63 | 0 |
| DLL1 | 0 | 0 | -1.52 | 0 |
| DLX1 | 0 | 0 | -1.95 | 0 |
| DMPK | 0 | 0 | -1.33 | 1.46 |
| DNAJC5B | 0 | 0 | 2.77 | 0 |
| DNASE1L1 | 0 | 0 | 1.55 | 0 |
| DNASE1L2 | 0 | 0 | 3.24 | 0 |
| DND1 | 0 | 0 | 1.37 | 0 |
| DNM2 | 0 | 0 | 1.26 | 1.16 |
| DNMT3L | 0 | 0 | -2.95 | -6.98 |
| DOCK3 | 0 | 0 | -1.96 | 0 |
| DOCK5 | 0 | 0 | 1.18 | 0 |
| DOK7 | 0 | 0 | 1.67 | 0 |
| DOT1L | 0 | 0 | 1.63 | 0 |
| DPP3 | 0 | 0 | 1.33 | 0 |
| DPYS | 0 | 0 | -3.59 | -7.93 |
| DSG2 | 0 | 0 | 2.36 | 0 |
| DSP | 0 | 0 | 1.68 | 0 |
| DTL | 0 | 0 | 2.04 | 3.58 |
| DTX2 | 0 | 0 | 2.61 | 1.09 |
| DTYMK | 0 | 0 | 1.21 | 1.49 |
| DUSP1 | 0 | 0 | -1.43 | 0 |
| DUSP26 | 0 | 0 | -1.81 | 0 |
| DUSP4 | 0 | 0 | 6.56 | 1.54 |
| DZIP1 | 0 | 0 | -2.31 | 1.87 |
| E2F2 | 0 | 0 | 2.79 | 4.37 |
| EBF1 | 0 | 0 | -2.88 | 0 |
| ECT2 | 0 | 0 | 2.34 | 2.67 |
| EDN2 | 0 | 0 | 2.68 | 0 |
| EEF1A2 | 0 | 0 | 3.7 | 3.77 |
| EFHD1 | 0 | 0 | -2.34 | -2.3 |
| EFS | 0 | 0 | -1.99 | 0 |
| EGFL7 | 0 | 0 | -1.45 | 0 |
| EGFLAM | 0 | 0 | -1.53 | 0 |
| EGR2 | 0 | 0 | -1.97 | 0 |
| EHF | 0 | 0 | 1.67 | 3.07 |
| EID1 | 0 | 0 | -1.73 | 0 |
| ELF3 | 0 | 0 | 1.53 | 0 |
| ELMO3 | 0 | 0 | 2.21 | 0 |
| ELMOD2 | 0 | 0 | 1.07 | 0 |
| ELN | 0 | 0 | -1.61 | 0 |
| EMCN | 0 | 0 | -2.56 | 0 |
| EMP1 | 0 | 0 | -1.75 | 2.74 |
| EMX2 | 0 | 0 | -3.77 | 0 |
| ENGASE | 0 | 0 | 1.62 | 0 |
| ENTPD2 | 0 | 0 | 7.01 | 1.83 |
| ENTPD6 | 0 | 0 | 3.37 | 1 |
| EPAS1 | 0 | 0 | -1.07 | 0 |
| EPHA5 | 0 | 0 | -2.09 | 0 |
| ERCC8 | 0 | 0 | 7.41 | 0 |
| ERI2 | 0 | 0 | 2.94 | -1.31 |
| ERMP1 | 0 | 0 | 1.29 | 0 |
| ERO1A | 0 | 0 | 1.39 | 0 |
| ERO1B | 0 | 0 | -2.31 | 0 |
| ERP27 | 0 | 0 | -4 | 1.76 |
| EYA3 | 0 | 0 | 2.18 | 1.05 |
| F2RL1 | 0 | 0 | 1.59 | 0 |
| F8A1 | 0 | 0 | 2.89 | 0 |
| FAAP24 | 0 | 0 | 1.47 | 0 |
| FABP4 | 0 | 0 | -4.03 | 0 |
| FADS3 | 0 | 0 | -1.46 | 0 |
| FAM109A | 0 | 0 | 1.45 | 0 |
| FAM117B | 0 | 0 | 1.28 | 1.32 |
| FAM126B | 0 | 0 | 1.08 | 0 |
| FAM129A | 0 | 0 | -2.4 | 1.85 |
| FAM150B | 0 | 0 | -2.44 | -1.93 |
| FAM167A | 0 | 0 | 2.49 | 1.87 |
| FAM181B | 0 | 0 | -3.59 | 0 |
| FAM198B | 0 | 0 | -1.51 | 1.25 |
| FAM212A | 0 | 0 | -1.25 | 0 |
| FAM21C | 0 | 0 | 2.21 | 0 |
| FAM222B | 0 | 0 | 1.22 | 0 |
| FAM46B | 0 | 0 | -2.68 | 2.22 |
| FAM60A | 0 | 0 | 1.88 | 1.8 |
| FAM64A | 0 | 0 | 5.39 | 2.67 |
| FAM83B | 0 | 0 | 5.83 | 4.63 |
| FAM83C | 0 | 0 | -1.41 | 0 |
| FAM91A1 | 0 | 0 | 1.84 | 0 |
| FANCC | 0 | 0 | -1.34 | -2.12 |
| FANCD2 | 0 | 0 | 1.59 | 2.35 |
| FANK1 | 0 | 0 | 1.74 | 3.04 |
| FBXL7 | 0 | 0 | -1.73 | 0 |
| FBXL8 | 0 | 0 | 2.49 | 0 |
| FBXO17 | 0 | 0 | 3.42 | -3.01 |
| FCRL3 | 0 | 0 | 1.56 | 0 |
| FEN1 | 0 | 0 | 1.64 | 2 |
| FERMT2 | 0 | 0 | -1.16 | -1.42 |
| FES | 0 | 0 | -1.57 | -1.44 |
| FEZ1 | 0 | 0 | -1.79 | 0 |
| FGD4 | 0 | 0 | 1.14 | 0 |
| FGD5 | 0 | 0 | -1.52 | 0 |
| FHL1 | 0 | 0 | -3.53 | 0 |
| FHL5 | 0 | 0 | -3.78 | 0 |
| FIBCD1 | 0 | 0 | 2.18 | 0 |
| FKBPL | 0 | 0 | 1.14 | 0 |
| FLNC | 0 | 0 | -2.19 | 2.87 |
| FLYWCH1 | 0 | 0 | -2.25 | 0 |
| FMNL3 | 0 | 0 | 4.22 | 1.67 |
| FMO2 | 0 | 0 | -2.33 | 0 |
| FNBP1L | 0 | 0 | 1.02 | 0 |
| FOXF1 | 0 | 0 | -2.91 | 0 |
| FOXO1 | 0 | 0 | -1.12 | 0 |
| FOXP1 | 0 | 0 | 1.72 | 0 |
| FPGS | 0 | 0 | 1.15 | 0 |
| FRZB | 0 | 0 | -2.4 | 0 |
| FXYD6 | 0 | 0 | -2.54 | 0 |
| FZD3 | 0 | 0 | -1.82 | 1.01 |
| FZD4 | 0 | 0 | -1.35 | 0 |
| GABPB2 | 0 | 0 | 2.59 | 0 |
| GABRG3 | 0 | 0 | 4.16 | 0 |
| GAD1 | 0 | 0 | 2.41 | 3.44 |
| GADD45B | 0 | 0 | -1.93 | -1.72 |
| GALE | 0 | 0 | 2.36 | 0 |
| GALNT15 | 0 | 0 | -2.44 | 0 |
| GALNT2 | 0 | 0 | -1.42 | 0 |
| GAREM1 | 0 | 0 | 1.3 | 0 |
| GARNL3 | 0 | 0 | -1.51 | -1.29 |
| GAS1 | 0 | 0 | -2.48 | 0 |
| GAS2L2 | 0 | 0 | -1.92 | 0 |
| GAS7 | 0 | 0 | -2.16 | 0 |
| GBA | 0 | 0 | 1.17 | 0 |
| GBP3 | 0 | 0 | 2.63 | 1.46 |
| GCNT2 | 0 | 0 | 4.82 | -1.58 |
| GET4 | 0 | 0 | 2.85 | 0 |
| GFRA3 | 0 | 0 | -3.62 | 0 |
| GGCT | 0 | 0 | 1.7 | 1.57 |
| GHRL | 0 | 0 | -6.08 | 0 |
| GIMAP1 | 0 | 0 | -1.43 | 0 |
| GIMAP8 | 0 | 0 | -1.21 | 0 |
| GINS2 | 0 | 0 | 1.84 | 2.24 |
| GINS4 | 0 | 0 | 2.01 | 2.52 |
| GJC2 | 0 | 0 | -1.63 | 0 |
| GLB1L | 0 | 0 | 1.07 | 0 |
| GLDN | 0 | 0 | -2.28 | 1.15 |
| GMIP | 0 | 0 | 1.08 | 1.28 |
| GNG11 | 0 | 0 | -1.51 | 0 |
| GNG2 | 0 | 0 | -1.57 | 0 |
| GNLY | 0 | 0 | 3.21 | 1.5 |
| GON4L | 0 | 0 | 2.75 | 0 |
| GPAT4 | 0 | 0 | 1.72 | 0 |
| GPATCH2 | 0 | 0 | 1.31 | 0 |
| GPC3 | 0 | 0 | -2.4 | 0 |
| GPC5 | 0 | 0 | -2.94 | 0 |
| GPD1L | 0 | 0 | -1.14 | 2.38 |
| GPER1 | 0 | 0 | -2.91 | 0 |
| GPM6A | 0 | 0 | -3.35 | -3.76 |
| GPR37L1 | 0 | 0 | -1.87 | 0 |
| GPR4 | 0 | 0 | -1.58 | 0 |
| GPR68 | 0 | 0 | 2.53 | 2.54 |
| GPR87 | 0 | 0 | 4.22 | 0 |
| GRAP2 | 0 | 0 | 1.75 | 0 |
| GRASP | 0 | 0 | -2.3 | 0 |
| GRB7 | 0 | 0 | 1.78 | 0 |
| GRIK2 | 0 | 0 | 3.56 | 0 |
| GRIN2A | 0 | 0 | 2.92 | 0 |
| GRIN2C | 0 | 0 | -2.22 | 0 |
| GRK6 | 0 | 0 | 1.03 | 1.1 |
| GSTA4 | 0 | 0 | -1.01 | 0 |
| GTF2A1 | 0 | 0 | 4.22 | 0 |
| GTF3C1 | 0 | 0 | 1.05 | 0 |
| GTPBP2 | 0 | 0 | 1.74 | 0 |
| GUF1 | 0 | 0 | 1.09 | 0 |
| H6PD | 0 | 0 | -1.68 | -1.74 |
| HAGHL | 0 | 0 | 3.26 | 1.49 |
| HAPLN4 | 0 | 0 | -1.93 | -2.97 |
| HAUS8 | 0 | 0 | 1.12 | 0 |
| HBEGF | 0 | 0 | -1.94 | 2.12 |
| HCP5 | 0 | 0 | 1.38 | 0 |
| HEMGN | 0 | 0 | -1.51 | 0 |
| HEMK1 | 0 | 0 | -1.8 | 0 |
| HEPN1 | 0 | 0 | -5.08 | -3.44 |
| HES5 | 0 | 0 | -2.37 | 0 |
| HEY2 | 0 | 0 | -2.55 | -1.49 |
| HEYL | 0 | 0 | -1.76 | 1.4 |
| HHAT | 0 | 0 | 1.31 | 0 |
| HIST1H1D | 0 | 0 | 1.87 | 2.67 |
| HIST1H2AJ | 0 | 0 | 1.62 | 1.84 |
| HIST1H2AM | 0 | 0 | 2.27 | 1.3 |
| HIST1H4H | 0 | 0 | 1.92 | 1.74 |
| HIST1H4I | 0 | 0 | 4.7 | 2.16 |
| HIST2H2AA3 | 0 | 0 | 1.96 | 0 |
| HIST2H2AC | 0 | 0 | 1.83 | 0 |
| HIST2H2BE | 0 | 0 | 1.86 | 1.51 |
| HIST2H3D | 0 | 0 | 3.47 | 1.69 |
| HIST2H4A | 0 | 0 | 2.01 | 0 |
| HK2 | 0 | 0 | 3.25 | 3.27 |
| HKDC1 | 0 | 0 | 2.71 | 0 |
| HMMR | 0 | 0 | 3.57 | 3.53 |
| HOPX | 0 | 0 | 2.26 | 3.88 |
| HOXA10 | 0 | 0 | 2.51 | 4.11 |
| HOXA4 | 0 | 0 | -2.14 | 0 |
| HOXA5 | 0 | 0 | -1.97 | 0 |
| HOXA9 | 0 | 0 | 2.44 | 3.96 |
| HOXB7 | 0 | 0 | 2.88 | 2.32 |
| HOXB9 | 0 | 0 | 2.49 | 5 |
| HPCAL1 | 0 | 0 | 1.42 | 0 |
| HPS1 | 0 | 0 | 1.92 | 0 |
| HPS3 | 0 | 0 | 1.37 | 1.4 |
| HSD11B1L | 0 | 0 | 1.35 | 0 |
| HSH2D | 0 | 0 | 3.17 | 1.42 |
| HSPA12B | 0 | 0 | -1.71 | 0 |
| HSPB2 | 0 | 0 | -1.61 | 0 |
| HSPBP1 | 0 | 0 | 1.47 | 0 |
| HYAL2 | 0 | 0 | -1.8 | 0 |
| ID1 | 0 | 0 | -2.58 | -1.28 |
| ID3 | 0 | 0 | -1.96 | 0 |
| ID4 | 0 | 0 | -2.27 | 0 |
| IFNW1 | 0 | 0 | 2.39 | 0 |
| IGF2BP3 | 0 | 0 | 2.67 | 3.99 |
| IGFBP6 | 0 | 0 | -2.76 | 2.46 |
| IGSF11 | 0 | 0 | -3.25 | 0 |
| IGSF3 | 0 | 0 | 2.5 | 3.42 |
| IGSF8 | 0 | 0 | 1.51 | 0 |
| IKBKE | 0 | 0 | 1.52 | 1.97 |
| IL17A | 0 | 0 | 10.41 | 0 |
| IL17D | 0 | 0 | -3.31 | 0 |
| IL21R | 0 | 0 | 1.71 | 2.21 |
| IL23A | 0 | 0 | 1.94 | 0 |
| IL6 | 0 | 0 | -4.57 | 0 |
| ILF3 | 0 | 0 | 1.26 | 1.16 |
| INF2 | 0 | 0 | 2.43 | 1.38 |
| IRF6 | 0 | 0 | 1.61 | 0 |
| IRX2 | 0 | 0 | 3.1 | 0 |
| ITGA8 | 0 | 0 | -4.4 | 0 |
| ITM2A | 0 | 0 | -2.82 | 0 |
| ITPKA | 0 | 0 | 2.04 | 0 |
| ITPR1 | 0 | 0 | -1.87 | 0 |
| JAK2 | 0 | 0 | -1.34 | 1.18 |
| JAM3 | 0 | 0 | -1.47 | 1.33 |
| JPH2 | 0 | 0 | -2.63 | 0 |
| JUN | 0 | 0 | -1.24 | 0 |
| KANK3 | 0 | 0 | -1.83 | -1.03 |
| KAT2B | 0 | 0 | -1.14 | -1.28 |
| KAZALD1 | 0 | 0 | -2.98 | 2.33 |
| KCNA5 | 0 | 0 | -4.59 | 0 |
| KCNAB1 | 0 | 0 | -3.46 | -1.41 |
| KCNB1 | 0 | 0 | -1.89 | -3.59 |
| KCNJ8 | 0 | 0 | -1.99 | -2.96 |
| KCNJ9 | 0 | 0 | 3.62 | 0 |
| KCNMA1 | 0 | 0 | -2.62 | 0 |
| KCNMB1 | 0 | 0 | -3.21 | 0 |
| KCNMB4 | 0 | 0 | -2.24 | 0 |
| KCNQ2 | 0 | 0 | 3.18 | 0 |
| KCNT1 | 0 | 0 | -2.87 | 0 |
| KCTD12 | 0 | 0 | -1.65 | 1.46 |
| KDELR1 | 0 | 0 | 1.08 | 0 |
| KDF1 | 0 | 0 | 2.21 | 0 |
| KDM5C | 0 | 0 | 2.96 | 0 |
| KHDRBS3 | 0 | 0 | -1.12 | 0 |
| KIAA0040 | 0 | 0 | 1.28 | 1.06 |
| KIAA0319L | 0 | 0 | 1.53 | 0 |
| KIAA0753 | 0 | 0 | 1.22 | 0 |
| KIAA1524 | 0 | 0 | 2.67 | 0 |
| KIF11 | 0 | 0 | 3.55 | 3.26 |
| KIF20A | 0 | 0 | 3.96 | 3.66 |
| KIF2A | 0 | 0 | 1.09 | 1.96 |
| KIF2C | 0 | 0 | 2.64 | 2.67 |
| KL | 0 | 0 | -2.17 | 1.85 |
| KLC2 | 0 | 0 | 1.36 | 1.68 |
| KLC4 | 0 | 0 | 2.51 | -2.1 |
| KLF10 | 0 | 0 | -1.19 | 0 |
| KLF11 | 0 | 0 | -1.15 | -1.74 |
| KLF15 | 0 | 0 | -1.84 | -1.85 |
| KLF2 | 0 | 0 | -1.58 | 0 |
| KLHL21 | 0 | 0 | -1.05 | 0 |
| KLHL30 | 0 | 0 | -1.72 | 0 |
| KLK1 | 0 | 0 | -3.76 | 6.07 |
| KLRC3 | 0 | 0 | 3.45 | 0 |
| KNOP1 | 0 | 0 | 2.35 | 0 |
| KRT18 | 0 | 0 | 1.64 | 0 |
| KRT222 | 0 | 0 | -2.14 | -2.4 |
| KRT7 | 0 | 0 | 2.13 | 1.54 |
| KRT8 | 0 | 0 | 1.71 | 0 |
| KRT80 | 0 | 0 | 2.69 | 3.64 |
| KRTAP10-2 | 0 | 0 | 3.4 | -1.27 |
| KRTCAP3 | 0 | 0 | 2.1 | 0 |
| LACRT | 0 | 0 | -1.06 | 0 |
| LACTB | 0 | 0 | 1.2 | 0 |
| LAMB4 | 0 | 0 | 2.98 | 0 |
| LARGE1 | 0 | 0 | -1.65 | 0 |
| LCN6 | 0 | 0 | -4.79 | 0 |
| LDAH | 0 | 0 | 1.14 | 0 |
| LDB2 | 0 | 0 | -1.52 | -1.12 |
| LDB3 | 0 | 0 | -2.55 | 0 |
| LDHAL6A | 0 | 0 | 2.99 | 0 |
| LGALS8 | 0 | 0 | 1.81 | 0 |
| LGALS9 | 0 | 0 | 1.81 | 1.22 |
| LGR6 | 0 | 0 | -2.78 | 0 |
| LHFP | 0 | 0 | -1.64 | 0 |
| LHX6 | 0 | 0 | -2.42 | 2.09 |
| LIG1 | 0 | 0 | 1.41 | 1.06 |
| LILRA6 | 0 | 0 | -1.13 | 0 |
| LIMS2 | 0 | 0 | -1.03 | 0 |
| LIN9 | 0 | 0 | 1.24 | 1.61 |
| LINC00341 | 0 | 0 | -2.17 | 0 |
| LMBRD2 | 0 | 0 | 2.47 | 0 |
| LMNB1 | 0 | 0 | 1.29 | 2 |
| LMNTD2 | 0 | 0 | 1.39 | 0 |
| LMO2 | 0 | 0 | -1.17 | 0 |
| LMO3 | 0 | 0 | -3.59 | 0 |
| LMOD1 | 0 | 0 | -2.89 | 0 |
| LMTK2 | 0 | 0 | 1.43 | 0 |
| LOC389834 | 0 | 0 | 2.47 | 0 |
| LOC90768 | 0 | 0 | -2.29 | 0 |
| LOXL4 | 0 | 0 | 1.98 | 0 |
| LPAR1 | 0 | 0 | -2.28 | 0 |
| LPL | 0 | 0 | -2.74 | 0 |
| LRBA | 0 | 0 | 1.53 | 0 |
| LRCH2 | 0 | 0 | -1.38 | 2.08 |
| LRRC61 | 0 | 0 | 1.35 | -1.17 |
| LRRC8E | 0 | 0 | 1.03 | 0 |
| LSM4 | 0 | 0 | 1.04 | 0 |
| LTBP4 | 0 | 0 | -1.68 | 0 |
| LTC4S | 0 | 0 | -1.87 | 0 |
| LYPLA2 | 0 | 0 | 1.38 | 0 |
| LYRM5 | 0 | 0 | -1.06 | -2.26 |
| MAATS1 | 0 | 0 | -1.67 | 0 |
| MACF1 | 0 | 0 | 3.66 | 0 |
| MAD2L1 | 0 | 0 | 2.42 | 3.18 |
| MAFG | 0 | 0 | 1.75 | 1.12 |
| MAG | 0 | 0 | -2.9 | 0 |
| MAGEA8 | 0 | 0 | 4.66 | 0 |
| MAGEB1 | 0 | 0 | -1.04 | 0 |
| MAGEH1 | 0 | 0 | -1.21 | 0 |
| MAL | 0 | 0 | -3.55 | 0 |
| MAL2 | 0 | 0 | 2.61 | 0 |
| MAMDC2 | 0 | 0 | -2.75 | 0 |
| MAMSTR | 0 | 0 | -1.47 | 0 |
| MAN2B1 | 0 | 0 | 1.39 | 0 |
| MAP2K2 | 0 | 0 | 1.01 | 0 |
| MAP3K6 | 0 | 0 | -1.12 | 1.6 |
| MAPKBP1 | 0 | 0 | 1.59 | 0 |
| MARK1 | 0 | 0 | -2.12 | 1.99 |
| MARK2 | 0 | 0 | 2.17 | 1.29 |
| MASTL | 0 | 0 | 2.45 | 1.02 |
| MBTPS2 | 0 | 0 | 1.2 | 0 |
| MCAM | 0 | 0 | -2.7 | 1.31 |
| MCM10 | 0 | 0 | 3.85 | 3.11 |
| MCM3 | 0 | 0 | 1.02 | 1.96 |
| MCM6 | 0 | 0 | 1.83 | 2.36 |
| MCM7 | 0 | 0 | 5.09 | 1.07 |
| MED15 | 0 | 0 | 1.71 | 1.48 |
| MED22 | 0 | 0 | 1.1 | 0 |
| MEDAG | 0 | 0 | -2.94 | 0 |
| MEF2C | 0 | 0 | -1.39 | 0 |
| MEIS1 | 0 | 0 | -1 | 1.43 |
| MELTF | 0 | 0 | 1.33 | 0 |
| MEOX1 | 0 | 0 | -2.8 | 0 |
| MEOX2 | 0 | 0 | -3.44 | 0 |
| MEST | 0 | 0 | 3.3 | 0 |
| MFGE8 | 0 | 0 | -1.32 | 2.34 |
| MFSD12 | 0 | 0 | 1.02 | 0 |
| MGAT4B | 0 | 0 | 2.49 | 0 |
| MIF | 0 | 0 | 1.21 | 0 |
| MINK1 | 0 | 0 | 1.9 | 0 |
| MLANA | 0 | 0 | -4.26 | 0 |
| MLH3 | 0 | 0 | 1.77 | 0 |
| MLLT11 | 0 | 0 | -1.71 | 0 |
| MLST8 | 0 | 0 | 1.01 | 0 |
| MLYCD | 0 | 0 | -1.37 | -2.13 |
| MMP3 | 0 | 0 | 3.34 | 4.02 |
| MMRN1 | 0 | 0 | -4.4 | 0 |
| MMRN2 | 0 | 0 | -2.27 | 0 |
| MPC1 | 0 | 0 | -1.29 | 0 |
| MPL | 0 | 0 | -1.32 | 0 |
| MPZ | 0 | 0 | -5.51 | -1.07 |
| MRC2 | 0 | 0 | 2.39 | 1.69 |
| MRE11A | 0 | 0 | 1.96 | 0 |
| MREG | 0 | 0 | 1.85 | 0 |
| MRGPRF | 0 | 0 | -3.2 | 0 |
| MRPL11 | 0 | 0 | 2.53 | 0 |
| MRPS12 | 0 | 0 | 1.09 | 0 |
| MRS2 | 0 | 0 | 1.5 | 0 |
| MRVI1 | 0 | 0 | -2.65 | 2.22 |
| MSI2 | 0 | 0 | 2.01 | 1.4 |
| MSRB3 | 0 | 0 | -2.17 | 1.98 |
| MST1R | 0 | 0 | 2.93 | 4.43 |
| MT3 | 0 | 0 | -5.83 | 0 |
| MTFP1 | 0 | 0 | 1.66 | 0 |
| MTFR2 | 0 | 0 | 2.21 | 0 |
| MTURN | 0 | 0 | -2.59 | 0 |
| MTUS1 | 0 | 0 | -2.51 | 0 |
| MXRA7 | 0 | 0 | -1.68 | 1.44 |
| MYC | 0 | 0 | -1.78 | 1.92 |
| MYEOV | 0 | 0 | 3.66 | 0 |
| MYH10 | 0 | 0 | -1.73 | 0 |
| MYH11 | 0 | 0 | -3.66 | 0 |
| MYL9 | 0 | 0 | -2.58 | 1.68 |
| MYLK | 0 | 0 | -1.73 | -3.32 |
| MYO10 | 0 | 0 | 1.2 | 0 |
| MYOC | 0 | 0 | -4.97 | 0 |
| MYOT | 0 | 0 | -5.48 | -1.9 |
| MYOZ1 | 0 | 0 | -2.64 | 0 |
| MYOZ3 | 0 | 0 | -2.09 | 0 |
| NAA40 | 0 | 0 | 1.32 | 1.47 |
| NAALAD2 | 0 | 0 | -2.36 | 0 |
| NABP2 | 0 | 0 | 1 | 0 |
| NAP1L3 | 0 | 0 | -1.88 | 0 |
| NAP1L5 | 0 | 0 | -1.84 | 0 |
| NCAM2 | 0 | 0 | -3.21 | 0 |
| NCAPG | 0 | 0 | 3.93 | 3.28 |
| NCF2 | 0 | 0 | 1.67 | 1.24 |
| NDC1 | 0 | 0 | 1.92 | 0 |
| NDUFAF7 | 0 | 0 | 1.43 | 0 |
| NECTIN4 | 0 | 0 | 8.19 | 0 |
| NEK11 | 0 | 0 | 2.07 | 1.75 |
| NELFA | 0 | 0 | 1 | 0 |
| NES | 0 | 0 | -1.7 | 0 |
| NEXN | 0 | 0 | -2.31 | 1.39 |
| NFATC4 | 0 | 0 | -1.46 | 0 |
| NFIL3 | 0 | 0 | -1.53 | 0 |
| NFKB2 | 0 | 0 | 1.12 | 0 |
| NFKBIB | 0 | 0 | 3.82 | 0 |
| NFRKB | 0 | 0 | 1.13 | 0 |
| NFS1 | 0 | 0 | 1.95 | 0 |
| NFYA | 0 | 0 | 1.78 | 0 |
| NGF | 0 | 0 | -2.52 | -1.23 |
| NIM1K | 0 | 0 | -3.81 | 0 |
| NIPBL | 0 | 0 | 1.02 | 0 |
| NKAIN2 | 0 | 0 | -2.88 | -2.31 |
| NLGN2 | 0 | 0 | -1.2 | 0 |
| NLGN4X | 0 | 0 | -1.84 | 0 |
| NLRP8 | 0 | 0 | -1.41 | 0 |
| NME6 | 0 | 0 | 1.04 | 0 |
| NMRK2 | 0 | 0 | -3.55 | 0 |
| NOB1 | 0 | 0 | 1.02 | 1.06 |
| NOL9 | 0 | 0 | -1.31 | 0 |
| NOTUM | 0 | 0 | 4.76 | 0 |
| NOV | 0 | 0 | -2.83 | 3.63 |
| NPAS1 | 0 | 0 | 4.36 | 1.38 |
| NPHP1 | 0 | 0 | 2.27 | 1.35 |
| NPY | 0 | 0 | -4.05 | 0 |
| NR1D2 | 0 | 0 | -1.67 | 0 |
| NR2C1 | 0 | 0 | 1.67 | 0 |
| NR2C2AP | 0 | 0 | 1.41 | 1.32 |
| NR4A2 | 0 | 0 | -3.76 | 0 |
| NRIP2 | 0 | 0 | -2.16 | 0 |
| NRXN1 | 0 | 0 | -2.25 | 0 |
| NRXN3 | 0 | 0 | -3.17 | 1.24 |
| NSUN5P2 | 0 | 0 | 1.76 | 0 |
| NTS | 0 | 0 | -3.22 | 0 |
| NUP50 | 0 | 0 | 1.02 | 0 |
| NXPE3 | 0 | 0 | -2.04 | 0 |
| OCLN | 0 | 0 | 3.82 | 0 |
| OLFM1 | 0 | 0 | -2.55 | -2.31 |
| OLR1 | 0 | 0 | 2.29 | 5.15 |
| OMD | 0 | 0 | -2.68 | 0 |
| OPCML | 0 | 0 | -2.52 | 0 |
| OPN3 | 0 | 0 | 1.65 | -1.06 |
| OR51V1 | 0 | 0 | 2.37 | -1.04 |
| OR52N1 | 0 | 0 | 1.78 | 0 |
| ORC1 | 0 | 0 | 2.91 | 3.19 |
| OSR1 | 0 | 0 | -2.02 | 0 |
| OXCT2 | 0 | 0 | -1.49 | 0 |
| OXT | 0 | 0 | -2.44 | -3.18 |
| P2RX1 | 0 | 0 | -2.2 | 0 |
| P3H4 | 0 | 0 | 1.16 | 0 |
| PAIP1 | 0 | 0 | 1.53 | 0 |
| PAK1 | 0 | 0 | 1.07 | 1.73 |
| PAK6 | 0 | 0 | 1.59 | 2.74 |
| PALM | 0 | 0 | -1.29 | 0 |
| PAQR5 | 0 | 0 | 3.78 | 1.87 |
| PARM1 | 0 | 0 | -2.15 | 2.49 |
| PARPBP | 0 | 0 | 2.76 | 0 |
| PARS2 | 0 | 0 | 1.97 | 0 |
| PBK | 0 | 0 | 4.34 | 4.52 |
| PBX4 | 0 | 0 | 3.78 | 2.94 |
| PCDH9 | 0 | 0 | -2.96 | -1.38 |
| PCDHB10 | 0 | 0 | 4.38 | 3.16 |
| PCDHB4 | 0 | 0 | -2.31 | 1.52 |
| PCDHGA1 | 0 | 0 | 6.4 | 0 |
| PCDHGA2 | 0 | 0 | 5.04 | 0 |
| PCOLCE2 | 0 | 0 | -2.43 | -2.07 |
| PCSK2 | 0 | 0 | -2.32 | 0 |
| PDDC1 | 0 | 0 | 1.37 | 0 |
| PDE1B | 0 | 0 | -1.92 | 0 |
| PDE7A | 0 | 0 | 1.27 | 2.22 |
| PDE9A | 0 | 0 | 5.57 | 0 |
| PDGFRL | 0 | 0 | -2.51 | 3.9 |
| PDIA2 | 0 | 0 | -5.27 | 3.97 |
| PDZD4 | 0 | 0 | -1.97 | 0 |
| PDZD8 | 0 | 0 | 1.07 | 0 |
| PECAM1 | 0 | 0 | -1.21 | 0 |
| PEG3 | 0 | 0 | -1.99 | -2.56 |
| PEMT | 0 | 0 | -1.82 | -3.1 |
| PER1 | 0 | 0 | -2.26 | 0 |
| PERP | 0 | 0 | 1.21 | 0 |
| PEX3 | 0 | 0 | -1.24 | 0 |
| PEX6 | 0 | 0 | 2.6 | 0 |
| PGA5 | 0 | 0 | -2.42 | 0 |
| PHC3 | 0 | 0 | 2.22 | 0 |
| PHF19 | 0 | 0 | -1.22 | 1.85 |
| PIGG | 0 | 0 | 1.06 | 0 |
| PIK3CB | 0 | 0 | 1.16 | 0 |
| PIM3 | 0 | 0 | 1.27 | 0 |
| PITPNM1 | 0 | 0 | 1.03 | 1.03 |
| PITRM1 | 0 | 0 | 1.02 | 0 |
| PKIG | 0 | 0 | -1.01 | 0 |
| PKM | 0 | 0 | 2.12 | 0 |
| PKN1 | 0 | 0 | 1.36 | 0 |
| PKNOX2 | 0 | 0 | -1.25 | 0 |
| PKP3 | 0 | 0 | 7.01 | 6.39 |
| PLA2G1B | 0 | 0 | -10.78 | 0 |
| PLA2G2A | 0 | 0 | -3.99 | 0 |
| PLA2G7 | 0 | 0 | 1.75 | 0 |
| PLAC9 | 0 | 0 | -2.78 | 0 |
| PLEKHB2 | 0 | 0 | 1.25 | 1.34 |
| PLEKHJ1 | 0 | 0 | 1.2 | 0 |
| PLIN1 | 0 | 0 | -2.57 | -3.47 |
| PLIN3 | 0 | 0 | 1.04 | 0 |
| PLK1 | 0 | 0 | 1.8 | 2.03 |
| PLK4 | 0 | 0 | 1.58 | 4.05 |
| PLN | 0 | 0 | -3.85 | 0 |
| PLPP7 | 0 | 0 | -2.43 | 0 |
| PLS1 | 0 | 0 | 1.72 | 0 |
| PMAIP1 | 0 | 0 | 3.58 | 3.25 |
| PMEL | 0 | 0 | -2.87 | -1.67 |
| PMP2 | 0 | 0 | -4.73 | 0 |
| PMP22 | 0 | 0 | -2.05 | 2.6 |
| PNLIP | 0 | 0 | -12.04 | 0 |
| PNLIPRP1 | 0 | 0 | -8.38 | 0 |
| PNLIPRP2 | 0 | 0 | -8.09 | 0 |
| PODN | 0 | 0 | -1.64 | 0 |
| POFUT1 | 0 | 0 | 1.74 | 0 |
| POLD1 | 0 | 0 | 1.36 | 1.5 |
| POLD4 | 0 | 0 | 1.04 | 0 |
| POLQ | 0 | 0 | 2.18 | 3.7 |
| POLR3D | 0 | 0 | 1.02 | 0 |
| POP1 | 0 | 0 | 1.39 | 0 |
| PPARD | 0 | 0 | 1.55 | 0 |
| PPFIA1 | 0 | 0 | 2.57 | 0 |
| PPL | 0 | 0 | 1.45 | 0 |
| PPM1G | 0 | 0 | 4.36 | 1.13 |
| PPP1CA | 0 | 0 | 1.42 | 0 |
| PPP1R14A | 0 | 0 | -2.69 | 0 |
| PPP2R2B | 0 | 0 | -4.19 | 0 |
| PPP2R2C | 0 | 0 | 4.85 | 0 |
| PPP2R3B | 0 | 0 | 1.31 | 1.05 |
| PPP5C | 0 | 0 | 1.57 | 0 |
| PRELID3B | 0 | 0 | 1.76 | 0 |
| PRKAR1B | 0 | 0 | 1.37 | 0 |
| PRMT9 | 0 | 0 | -1.95 | 0 |
| PRPF6 | 0 | 0 | 1.09 | 0 |
| PRPH | 0 | 0 | -3.18 | 1.38 |
| PRPH2 | 0 | 0 | -1.67 | 0 |
| PRR11 | 0 | 0 | 2.02 | 2.04 |
| PRR5 | 0 | 0 | 2.01 | 0 |
| PRR7 | 0 | 0 | 1.9 | 1.01 |
| PRRT1 | 0 | 0 | -1.66 | 0 |
| PRRT2 | 0 | 0 | -1.99 | 1.21 |
| PRSS1 | 0 | 0 | -11.52 | 0 |
| PRSS2 | 0 | 0 | -9.05 | 0 |
| PRSS22 | 0 | 0 | 3.54 | 2.9 |
| PRSS3 | 0 | 0 | -7.52 | 0 |
| PRUNE2 | 0 | 0 | -2.73 | 3.16 |
| PSEN1 | 0 | 0 | 1.22 | 0 |
| PSIP1 | 0 | 0 | -1.13 | 0 |
| PTGIS | 0 | 0 | -2.3 | 0 |
| PTPN22 | 0 | 0 | 1.72 | 1.85 |
| PTPN6 | 0 | 0 | 1.12 | 0 |
| PTRF | 0 | 0 | -1.16 | 2.12 |
| PTX3 | 0 | 0 | -1.71 | 0 |
| PWWP2B | 0 | 0 | 1.16 | 0 |
| PYCARD | 0 | 0 | 1.52 | 2.25 |
| QDPR | 0 | 0 | -1.15 | -3.19 |
| R3HDM4 | 0 | 0 | 1.06 | 0 |
| RAB24 | 0 | 0 | 1.37 | 0 |
| RAB27B | 0 | 0 | 1.36 | 2.19 |
| RAB9B | 0 | 0 | -2.13 | 2.34 |
| RABL2A | 0 | 0 | 1.16 | 0 |
| RABL2B | 0 | 0 | 1.39 | 0 |
| RAC1 | 0 | 0 | 1.91 | 1.06 |
| RAE1 | 0 | 0 | 1.64 | 0 |
| RAI1 | 0 | 0 | 1.42 | 1.32 |
| RALGAPB | 0 | 0 | 1.27 | 0 |
| RANBP3 | 0 | 0 | 2.47 | 0 |
| RAP2B | 0 | 0 | 1.23 | 0 |
| RAPGEF3 | 0 | 0 | -1.95 | 0 |
| RARA | 0 | 0 | -1.96 | 0 |
| RASD1 | 0 | 0 | -2.65 | -2.33 |
| RASEF | 0 | 0 | 2.54 | 3.46 |
| RASGEF1C | 0 | 0 | -3.18 | 0 |
| RASIP1 | 0 | 0 | -1.72 | 0 |
| RASL12 | 0 | 0 | -2.76 | 2.32 |
| RASSF1 | 0 | 0 | -1.39 | 0 |
| RASSF5 | 0 | 0 | 1.69 | 0 |
| RBBP8 | 0 | 0 | -2.44 | 1.18 |
| RBM24 | 0 | 0 | -2.17 | 0 |
| RBM33 | 0 | 0 | 1.65 | 0 |
| RBPJL | 0 | 0 | -4.37 | 0 |
| RBPMS2 | 0 | 0 | -2.64 | -1.55 |
| RCAN1 | 0 | 0 | -1.48 | -2.13 |
| RCAN3 | 0 | 0 | 1.08 | 2.25 |
| RCC1 | 0 | 0 | 1.42 | 1.25 |
| REEP1 | 0 | 0 | -1.92 | 0 |
| REEP3 | 0 | 0 | 1.91 | 0 |
| REG1A | 0 | 0 | -7.34 | 5.45 |
| REG1B | 0 | 0 | -8.38 | 0 |
| REG3G | 0 | 0 | -7.39 | 0 |
| RELB | 0 | 0 | 1.41 | 1.39 |
| REM1 | 0 | 0 | -2.57 | 0 |
| RET | 0 | 0 | -2.3 | 0 |
| RFTN2 | 0 | 0 | -1.84 | 0 |
| RGCC | 0 | 0 | -1.26 | 0 |
| RGMA | 0 | 0 | -2.65 | 2.89 |
| RGS11 | 0 | 0 | -2.36 | 0 |
| RGS12 | 0 | 0 | 3.32 | 0 |
| RGS14 | 0 | 0 | 1.71 | -1.6 |
| RGS2 | 0 | 0 | -2.08 | 1.58 |
| RHAG | 0 | 0 | -2.46 | 0 |
| RHBDD3 | 0 | 0 | 1.06 | 0 |
| RHOJ | 0 | 0 | -1.3 | 0 |
| RIF1 | 0 | 0 | -1.88 | 0 |
| RIMS3 | 0 | 0 | -1.45 | 0 |
| RMI1 | 0 | 0 | 1.43 | 0 |
| RND3 | 0 | 0 | -1.23 | 0 |
| RNF122 | 0 | 0 | -1.88 | 0 |
| RNF123 | 0 | 0 | -1.16 | -1.56 |
| RNF44 | 0 | 0 | 1.05 | 1.17 |
| ROBO4 | 0 | 0 | -1.08 | 0 |
| RPL13 | 0 | 0 | 1.3 | 0 |
| RPRD2 | 0 | 0 | 1.24 | 0 |
| RPS6KA1 | 0 | 0 | 1.54 | 1.12 |
| RSPO3 | 0 | 0 | -2.79 | 0 |
| RTEL1 | 0 | 0 | 2.3 | 0 |
| RUNX1T1 | 0 | 0 | -2.33 | 0 |
| RXRG | 0 | 0 | -1.63 | 0 |
| S100A14 | 0 | 0 | 3.04 | 3.44 |
| S100B | 0 | 0 | -4.67 | 0 |
| S1PR3 | 0 | 0 | -1.59 | 0 |
| SAMD10 | 0 | 0 | 1.72 | 0 |
| SAMD3 | 0 | 0 | 2.81 | 1.34 |
| SAPCD2 | 0 | 0 | 2.41 | 0 |
| SASH1 | 0 | 0 | -1.64 | 0 |
| SBSPON | 0 | 0 | -4.13 | 0 |
| SCARA3 | 0 | 0 | -2.73 | 0 |
| SCGB3A1 | 0 | 0 | -6.88 | 0 |
| SCN4B | 0 | 0 | -2.01 | 0 |
| SCRG1 | 0 | 0 | -4.4 | 0 |
| SDCBP2 | 0 | 0 | 2.07 | 0 |
| SDPR | 0 | 0 | -2.39 | -1.47 |
| SELP | 0 | 0 | -2.72 | 0 |
| SEMA3B | 0 | 0 | -2.44 | 2.43 |
| SEMA6D | 0 | 0 | -2.63 | 0 |
| SERP2 | 0 | 0 | -1.74 | 0 |
| SERPINB1 | 0 | 0 | 1.17 | 2.15 |
| SERPINB5 | 0 | 0 | 4.66 | 7.4 |
| SERPINI2 | 0 | 0 | -7.86 | 1.93 |
| SFMBT1 | 0 | 0 | 1.02 | 0 |
| SFT2D2 | 0 | 0 | 1.42 | 0 |
| SGCA | 0 | 0 | -2 | 0 |
| SGCG | 0 | 0 | -2.19 | 0 |
| SGK1 | 0 | 0 | -1.21 | 0 |
| SH2D3A | 0 | 0 | 3.11 | 2.69 |
| SH2D3C | 0 | 0 | -1.6 | 0 |
| SH3BP1 | 0 | 0 | 1.43 | 1.16 |
| SHB | 0 | 0 | 1.15 | 0 |
| SHC1 | 0 | 0 | 2.26 | 0 |
| SHC4 | 0 | 0 | -3.4 | 0 |
| SIGIRR | 0 | 0 | 1.5 | -1.27 |
| SIGLEC10 | 0 | 0 | 3.13 | 0 |
| SIK1 | 0 | 0 | -1.68 | 0 |
| SIX4 | 0 | 0 | 2.99 | 4.15 |
| SLA2 | 0 | 0 | 2.65 | 1.26 |
| SLC10A1 | 0 | 0 | -4.82 | -7.16 |
| SLC12A6 | 0 | 0 | 1.17 | 0 |
| SLC16A2 | 0 | 0 | -1.45 | -3 |
| SLC22A17 | 0 | 0 | -1.65 | 0 |
| SLC22A18AS | 0 | 0 | 3.49 | 0 |
| SLC24A4 | 0 | 0 | -3.28 | 0 |
| SLC25A43 | 0 | 0 | 2.77 | 0 |
| SLC2A8 | 0 | 0 | -1.08 | 0 |
| SLC35F1 | 0 | 0 | -4.03 | 0 |
| SLC37A3 | 0 | 0 | 2.92 | 1.16 |
| SLC38A11 | 0 | 0 | -1.84 | 0 |
| SLC38A7 | 0 | 0 | 1.11 | 0 |
| SLC39A13 | 0 | 0 | 2.45 | 1.17 |
| SLC45A2 | 0 | 0 | -1.86 | -2.34 |
| SLC50A1 | 0 | 0 | 1.47 | 0 |
| SLC52A2 | 0 | 0 | 1.02 | 0 |
| SLIT2 | 0 | 0 | -1.6 | 2.37 |
| SLITRK2 | 0 | 0 | -2.05 | 0 |
| SLITRK5 | 0 | 0 | -2.91 | 0 |
| SLX1B | 0 | 0 | 1.38 | 0 |
| SMAD9 | 0 | 0 | -2.1 | 0 |
| SMARCC2 | 0 | 0 | 2.12 | 0 |
| SMC2 | 0 | 0 | 1.48 | 1.19 |
| SMC6 | 0 | 0 | 1.37 | 0 |
| SMCO3 | 0 | 0 | -2.57 | 0 |
| SMDT1 | 0 | 0 | -1.07 | 0 |
| SMTN | 0 | 0 | -1.4 | 0 |
| SMYD2 | 0 | 0 | 1.83 | 0 |
| SMYD5 | 0 | 0 | 1.53 | 0 |
| SNAI1 | 0 | 0 | -2.81 | 0 |
| SNCA | 0 | 0 | -2.62 | 1.2 |
| SNX5 | 0 | 0 | 1.55 | 0 |
| SOBP | 0 | 0 | -1.8 | 0 |
| SORBS1 | 0 | 0 | -2.74 | -1.64 |
| SORCS1 | 0 | 0 | -3.5 | 0 |
| SOX10 | 0 | 0 | -4.96 | 0 |
| SOX15 | 0 | 0 | -2.75 | 0 |
| SOX17 | 0 | 0 | -3.2 | 0 |
| SOX18 | 0 | 0 | -2.94 | 0 |
| SOX7 | 0 | 0 | -1.75 | 0 |
| SOX8 | 0 | 0 | -3.18 | 0 |
| SPAM1 | 0 | 0 | -2.13 | 0 |
| SPARCL1 | 0 | 0 | -3.2 | 0 |
| SPATS2 | 0 | 0 | 1.5 | 2.14 |
| SPATS2L | 0 | 0 | 1 | 0 |
| SPRY2 | 0 | 0 | -1.45 | 0 |
| SPTBN1 | 0 | 0 | -1.79 | 0 |
| SRC | 0 | 0 | 1.29 | 0 |
| SRF | 0 | 0 | -1.27 | 0 |
| SRPX | 0 | 0 | -2.7 | 0 |
| SS18 | 0 | 0 | 1.13 | 0 |
| SSBP2 | 0 | 0 | -1.29 | 0 |
| SST | 0 | 0 | -3.38 | 0 |
| ST14 | 0 | 0 | 2.49 | 1.55 |
| ST3GAL3 | 0 | 0 | -1.51 | 0 |
| ST3GAL6 | 0 | 0 | -2.02 | -2.85 |
| ST6GALNAC2 | 0 | 0 | -2.81 | 0 |
| ST6GALNAC6 | 0 | 0 | -1.14 | 0 |
| STAP2 | 0 | 0 | 1.36 | -1.81 |
| STARD8 | 0 | 0 | -1.27 | 0 |
| STAT1 | 0 | 0 | 1.79 | 1.37 |
| STK24 | 0 | 0 | 1.38 | 1.31 |
| STXBP2 | 0 | 0 | 1.31 | 0 |
| SULT1C2 | 0 | 0 | 4.28 | 3.35 |
| SULT2B1 | 0 | 0 | 2.97 | 3.93 |
| SUMF2 | 0 | 0 | 1.14 | 0 |
| SYNM | 0 | 0 | -2.25 | 0 |
| SYNRG | 0 | 0 | 1.12 | 0 |
| SYT11 | 0 | 0 | -1.33 | 1.94 |
| SYT4 | 0 | 0 | -1.98 | 0 |
| SYTL3 | 0 | 0 | 1.72 | 1.13 |
| TACC3 | 0 | 0 | 2.06 | 2.44 |
| TAGLN | 0 | 0 | -1.48 | 2.09 |
| TAGLN2 | 0 | 0 | 1.46 | 1.53 |
| TAP1 | 0 | 0 | 1.13 | 2.76 |
| TAPBP | 0 | 0 | 1.17 | 0 |
| TAS2R4 | 0 | 0 | 2.97 | 0 |
| TBC1D26 | 0 | 0 | 2.81 | 0 |
| TBCCD1 | 0 | 0 | 1.11 | 0 |
| TBRG4 | 0 | 0 | 1.16 | 0 |
| TBXA2R | 0 | 0 | -1.64 | -1.53 |
| TCEAL1 | 0 | 0 | -1.02 | 0 |
| TCEAL2 | 0 | 0 | -5.39 | 0 |
| TCEAL4 | 0 | 0 | -1.51 | 0 |
| TCEAL7 | 0 | 0 | -2.22 | 0 |
| TCEANC2 | 0 | 0 | 1.53 | 0 |
| TCF20 | 0 | 0 | 1.15 | 1.17 |
| TCF21 | 0 | 0 | -1.51 | 0 |
| TEK | 0 | 0 | -1.82 | 0 |
| TES | 0 | 0 | 1.66 | 2.58 |
| TESMIN | 0 | 0 | 2.22 | 0 |
| TET2 | 0 | 0 | 3.67 | 1.38 |
| TEX26 | 0 | 0 | -2.1 | 0 |
| TFAP2C | 0 | 0 | 3.54 | 0 |
| TGFA | 0 | 0 | 2.04 | 2 |
| TGS1 | 0 | 0 | 3.69 | 0 |
| THBD | 0 | 0 | -2.8 | 0 |
| THBS4 | 0 | 0 | -3.66 | 0 |
| THOC6 | 0 | 0 | 1.33 | 0 |
| THRAP3 | 0 | 0 | 1.19 | 0 |
| TICAM1 | 0 | 0 | 1.32 | 0 |
| TIE1 | 0 | 0 | -1.47 | 0 |
| TIMM10 | 0 | 0 | 2.41 | 0 |
| TIMP3 | 0 | 0 | -1.18 | 0 |
| TIMP4 | 0 | 0 | -1.92 | 0 |
| TIPARP | 0 | 0 | -1.29 | 0 |
| TIPRL | 0 | 0 | 1.22 | 0 |
| TLCD1 | 0 | 0 | 1.95 | 0 |
| TLDC2 | 0 | 0 | 3.09 | 0 |
| TLE3 | 0 | 0 | 1.29 | 1.24 |
| TLL1 | 0 | 0 | -3.08 | 0 |
| TMC7 | 0 | 0 | 2.22 | 3.48 |
| TMEM100 | 0 | 0 | -2.52 | 0 |
| TMEM110 | 0 | 0 | -1.5 | -1.27 |
| TMEM139 | 0 | 0 | 2.25 | 0 |
| TMEM189-UBE2V1 | 0 | 0 | 1.88 | 0 |
| TMEM25 | 0 | 0 | -2.65 | -1.48 |
| TMEM259 | 0 | 0 | 1.12 | 0 |
| TMEM35A | 0 | 0 | -2.24 | 0 |
| TMEM41A | 0 | 0 | 1.2 | 0 |
| TMEM51 | 0 | 0 | 1.23 | 2.04 |
| TMEM62 | 0 | 0 | 1.19 | 0 |
| TMEM87B | 0 | 0 | 1.72 | 1.65 |
| TMEM8A | 0 | 0 | 1.28 | 0 |
| TMOD1 | 0 | 0 | -3.4 | -1.38 |
| TMOD4 | 0 | 0 | 2.39 | 1.52 |
| TMPO | 0 | 0 | 2.23 | 0 |
| TMUB2 | 0 | 0 | 2.24 | 0 |
| TNFAIP8L3 | 0 | 0 | -2.19 | 0 |
| TNFRSF12A | 0 | 0 | 1.57 | 0 |
| TNFRSF21 | 0 | 0 | 1.84 | 2.27 |
| TNFSF12 | 0 | 0 | -1.03 | 0 |
| TNFSF8 | 0 | 0 | 2.1 | 0 |
| TOP1MT | 0 | 0 | 1.7 | 0 |
| TP53 | 0 | 0 | 2.05 | 0 |
| TPD52 | 0 | 0 | 1.87 | 0 |
| TPX2 | 0 | 0 | 2.25 | 2.8 |
| TRADD | 0 | 0 | 1.52 | 0 |
| TRAF2 | 0 | 0 | 1.18 | 1.26 |
| TRAF4 | 0 | 0 | 1.23 | 0 |
| TRAIP | 0 | 0 | -1.68 | 2.32 |
| TRIM10 | 0 | 0 | 1.86 | 0 |
| TRIM41 | 0 | 0 | 1.24 | 0 |
| TRIM5 | 0 | 0 | 1.14 | 0 |
| TRIM69 | 0 | 0 | 1.6 | 0 |
| TRMT2B | 0 | 0 | 1.2 | 0 |
| TRPM7 | 0 | 0 | 1.28 | 0 |
| TSGA10 | 0 | 0 | 3.66 | 0 |
| TSLP | 0 | 0 | -1.64 | -3.28 |
| TSPAN18 | 0 | 0 | -2.06 | 0 |
| TSTA3 | 0 | 0 | 1.03 | 0 |
| TSTD2 | 0 | 0 | 1.27 | 0 |
| TTC9C | 0 | 0 | 1.32 | 1.26 |
| TTK | 0 | 0 | 4.57 | 5.19 |
| TTYH2 | 0 | 0 | -1.55 | 0 |
| TUB | 0 | 0 | -2.63 | 0 |
| TXK | 0 | 0 | 2.44 | 0 |
| TYMP | 0 | 0 | 1.76 | 0 |
| TYR | 0 | 0 | -3.18 | 0 |
| UBALD2 | 0 | 0 | 1.59 | 0 |
| UBE2I | 0 | 0 | 3.83 | 1.23 |
| UBL7 | 0 | 0 | 1.67 | 0 |
| UFD1L | 0 | 0 | 1.78 | 0 |
| UNC5A | 0 | 0 | 3.02 | 0 |
| UNC5B | 0 | 0 | 2.94 | 1.98 |
| UNC93B1 | 0 | 0 | 2.33 | 1.12 |
| USF1 | 0 | 0 | 1.05 | 0 |
| USMG5 | 0 | 0 | 2.29 | 0 |
| USP12 | 0 | 0 | 1.53 | 0 |
| USP28 | 0 | 0 | 7.95 | 0 |
| USP29 | 0 | 0 | -2.36 | 0 |
| UST | 0 | 0 | -1.86 | 1.11 |
| UTP14A | 0 | 0 | 1.06 | 1.11 |
| VASP | 0 | 0 | 2.36 | 1.55 |
| VEPH1 | 0 | 0 | 3.13 | 1.82 |
| VEZT | 0 | 0 | 1.15 | 0 |
| VGLL3 | 0 | 0 | -2.71 | 2.6 |
| VIP | 0 | 0 | -4.16 | 0 |
| VIPR2 | 0 | 0 | -3.1 | 0 |
| VWF | 0 | 0 | -2.21 | 2.57 |
| WASF3 | 0 | 0 | -1.58 | 0 |
| WBSCR27 | 0 | 0 | 3.35 | 0 |
| WDR24 | 0 | 0 | 1.06 | 0 |
| WDR3 | 0 | 0 | 2.02 | 0 |
| WDR34 | 0 | 0 | 1.46 | 0 |
| WDR4 | 0 | 0 | 3.47 | 1.19 |
| WDR66 | 0 | 0 | 1.84 | 0 |
| WFDC1 | 0 | 0 | -2.31 | 0 |
| WHSC1L1 | 0 | 0 | 1.42 | 0 |
| WIPF2 | 0 | 0 | 1.09 | 1.14 |
| XIAP | 0 | 0 | 1.11 | 0 |
| XKR4 | 0 | 0 | -4.98 | 0 |
| XPNPEP1 | 0 | 0 | 1.61 | 1.16 |
| YARS2 | 0 | 0 | 1.02 | 0 |
| YDJC | 0 | 0 | 2.3 | 1.19 |
| YWHAZ | 0 | 0 | 1.16 | 1.69 |
| ZBED2 | 0 | 0 | 2.85 | 4.98 |
| ZBED4 | 0 | 0 | 1.1 | 1.12 |
| ZBED6CL | 0 | 0 | 1.17 | 0 |
| ZBTB32 | 0 | 0 | 2.74 | 3.21 |
| ZBTB47 | 0 | 0 | -1.57 | 0 |
| ZC3H3 | 0 | 0 | 1.67 | 0 |
| ZC3HAV1 | 0 | 0 | 1.64 | 1.43 |
| ZCCHC12 | 0 | 0 | 1.52 | 0 |
| ZCCHC13 | 0 | 0 | 2.59 | 0 |
| ZCCHC24 | 0 | 0 | -1.53 | -1.28 |
| ZDHHC12 | 0 | 0 | 1.21 | 0 |
| ZDHHC13 | 0 | 0 | 1.24 | 2.7 |
| ZEB2 | 0 | 0 | -1.68 | 0 |
| ZFAND3 | 0 | 0 | 1.12 | 0 |
| ZFAND5 | 0 | 0 | -1.06 | 0 |
| ZFX | 0 | 0 | 1.46 | 0 |
| ZHX3 | 0 | 0 | -1.32 | -1.25 |
| ZMAT3 | 0 | 0 | 1.12 | 0 |
| ZNF10 | 0 | 0 | -1.3 | 0 |
| ZNF165 | 0 | 0 | 2 | 0 |
| ZNF174 | 0 | 0 | 1.44 | 0 |
| ZNF205 | 0 | 0 | 1.16 | 0 |
| ZNF217 | 0 | 0 | 1.39 | 0 |
| ZNF296 | 0 | 0 | 1.25 | 1.07 |
| ZNF3 | 0 | 0 | 1.53 | 0 |
| ZNF331 | 0 | 0 | -3.39 | 0 |
| ZNF365 | 0 | 0 | 3.82 | 3.35 |
| ZNF367 | 0 | 0 | -1.56 | 0 |
| ZNF385D | 0 | 0 | -2.59 | 0 |
| ZNF414 | 0 | 0 | 1.67 | 0 |
| ZNF416 | 0 | 0 | 1.16 | 0 |
| ZNF473 | 0 | 0 | 1.21 | 0 |
| ZNF530 | 0 | 0 | 1.77 | 1.88 |
| ZNF536 | 0 | 0 | -1.53 | 0 |
| ZNF566 | 0 | 0 | 4.44 | 0 |
| ZNF567 | 0 | 0 | 1.58 | 1.16 |
| ZNF580 | 0 | 0 | 1.79 | 1.26 |
| ZNF581 | 0 | 0 | 1.51 | 1.37 |
| ZNF596 | 0 | 0 | 3.18 | 0 |
| ZNF605 | 0 | 0 | 1.72 | 0 |
| ZNF606 | 0 | 0 | 1.42 | 0 |
| ZNF669 | 0 | 0 | 1.63 | 1.28 |
| ZSCAN2 | 0 | 0 | 2.84 | 0 |
| AACS | 0 | 0 | 0 | 1.74 |
| AAGAB | 0 | 0 | 0 | 1.28 |
| AATF | 0 | 0 | 0 | 1.12 |
| AATK | 0 | 0 | 0 | 2.49 |
| ABCA1 | 0 | 0 | 0 | -1.34 |
| ABCA3 | 0 | 0 | 0 | 1.19 |
| ABCA7 | 0 | 0 | 0 | 1.66 |
| ABCB9 | 0 | 0 | 0 | 2.84 |
| ABCC11 | 0 | 0 | 0 | -4.82 |
| ABCC4 | 0 | 0 | 0 | 2.2 |
| ABCC5 | 0 | 0 | 0 | 1.04 |
| ABCD3 | 0 | 0 | 0 | -1.71 |
| ABCF2 | 0 | 0 | 0 | 1.04 |
| ABCG1 | 0 | 0 | 0 | 1.33 |
| ABCG2 | 0 | 0 | 0 | -2.73 |
| ABHD11 | 0 | 0 | 0 | 1.3 |
| ABHD14B | 0 | 0 | 0 | -1.41 |
| ABHD15 | 0 | 0 | 0 | -1.01 |
| ABHD2 | 0 | 0 | 0 | -1.28 |
| ABL1 | 0 | 0 | 0 | 1.26 |
| ABL2 | 0 | 0 | 0 | 1.03 |
| ABLIM2 | 0 | 0 | 0 | 1.98 |
| ABO | 0 | 0 | 0 | 3.06 |
| ABR | 0 | 0 | 0 | 2.25 |
| ABTB2 | 0 | 0 | 0 | -1.67 |
| ACADL | 0 | 0 | 0 | -5.25 |
| ACAN | 0 | 0 | 0 | 1.98 |
| ACBD4 | 0 | 0 | 0 | -2.07 |
| ACBD5 | 0 | 0 | 0 | -1.16 |
| ACBD6 | 0 | 0 | 0 | 1.08 |
| ACCN2 | 0 | 0 | 0 | 3.14 |
| ACD | 0 | 0 | 0 | 1.27 |
| ACER3 | 0 | 0 | 0 | 1.03 |
| ACLY | 0 | 0 | 0 | 1.02 |
| ACOT11 | 0 | 0 | 0 | 2.13 |
| ACOT13 | 0 | 0 | 0 | -1.78 |
| ACOT4 | 0 | 0 | 0 | -1.94 |
| ACOT6 | 0 | 0 | 0 | -3.36 |
| ACOT9 | 0 | 0 | 0 | 1.04 |
| ACOX3 | 0 | 0 | 0 | -1.01 |
| ACOXL | 0 | 0 | 0 | 4.02 |
| ACPL2 | 0 | 0 | 0 | 1.17 |
| ACPP | 0 | 0 | 0 | 2.22 |
| ACSL4 | 0 | 0 | 0 | 1.59 |
| ACSL6 | 0 | 0 | 0 | -1.89 |
| ACSM1 | 0 | 0 | 0 | -2.11 |
| ACSM2A | 0 | 0 | 0 | -5.65 |
| ACTBL2 | 0 | 0 | 0 | 1.08 |
| ACTG1 | 0 | 0 | 0 | 1.74 |
| ACTL6A | 0 | 0 | 0 | 1.14 |
| ACTN1 | 0 | 0 | 0 | 1.14 |
| ACTN3 | 0 | 0 | 0 | 1.43 |
| ACTR3 | 0 | 0 | 0 | 1.46 |
| ACTR3B | 0 | 0 | 0 | -1.3 |
| ACTR3C | 0 | 0 | 0 | -1.19 |
| ACVR1C | 0 | 0 | 0 | -1.22 |
| ACYP1 | 0 | 0 | 0 | 1.8 |
| ADA | 0 | 0 | 0 | 1.73 |
| ADAM15 | 0 | 0 | 0 | 1.69 |
| ADAM19 | 0 | 0 | 0 | 1.59 |
| ADAM22 | 0 | 0 | 0 | 1.89 |
| ADAM23 | 0 | 0 | 0 | 2.55 |
| ADAM28 | 0 | 0 | 0 | 2.7 |
| ADAM32 | 0 | 0 | 0 | 2.32 |
| ADAMDEC1 | 0 | 0 | 0 | 4.8 |
| ADAMTS17 | 0 | 0 | 0 | -2.8 |
| ADAMTS18 | 0 | 0 | 0 | 2.33 |
| ADAMTS6 | 0 | 0 | 0 | 2.83 |
| ADAMTSL3 | 0 | 0 | 0 | -3.41 |
| ADAT2 | 0 | 0 | 0 | 1.11 |
| ADCY1 | 0 | 0 | 0 | -3.78 |
| ADCY10 | 0 | 0 | 0 | -2.75 |
| ADCY3 | 0 | 0 | 0 | 2.11 |
| ADCY7 | 0 | 0 | 0 | 1.54 |
| ADCY9 | 0 | 0 | 0 | -1.21 |
| ADD2 | 0 | 0 | 0 | 1.66 |
| ADD3 | 0 | 0 | 0 | 1.33 |
| ADIPOR2 | 0 | 0 | 0 | -1.15 |
| ADK | 0 | 0 | 0 | -2.01 |
| ADRA2B | 0 | 0 | 0 | -3.2 |
| ADRBK2 | 0 | 0 | 0 | 1.29 |
| ADSS | 0 | 0 | 0 | 1.01 |
| ADSSL1 | 0 | 0 | 0 | -2.26 |
| AEBP1 | 0 | 0 | 0 | 2.53 |
| AFAP1L2 | 0 | 0 | 0 | 1.83 |
| AFF2 | 0 | 0 | 0 | 2.23 |
| AFF3 | 0 | 0 | 0 | -2 |
| AGAP2 | 0 | 0 | 0 | 1.2 |
| AGBL2 | 0 | 0 | 0 | -1.55 |
| AGBL4 | 0 | 0 | 0 | 1.18 |
| AGFG2 | 0 | 0 | 0 | -2.04 |
| AGL | 0 | 0 | 0 | -1.83 |
| AGPAT2 | 0 | 0 | 0 | -1.22 |
| AGPAT4 | 0 | 0 | 0 | 1.93 |
| AGR2 | 0 | 0 | 0 | 6.98 |
| AGR3 | 0 | 0 | 0 | 5.86 |
| AGRN | 0 | 0 | 0 | 1.77 |
| AGTRAP | 0 | 0 | 0 | 1.63 |
| AIFM1 | 0 | 0 | 0 | -1.55 |
| AIM1L | 0 | 0 | 0 | 4.59 |
| AK3 | 0 | 0 | 0 | -1.68 |
| AK4 | 0 | 0 | 0 | -1.88 |
| AKAP9 | 0 | 0 | 0 | 1.13 |
| AKR1B1 | 0 | 0 | 0 | 2.21 |
| AKR1C1 | 0 | 0 | 0 | -3.52 |
| AKT3 | 0 | 0 | 0 | 1.22 |
| ALAD | 0 | 0 | 0 | -2.52 |
| ALDH18A1 | 0 | 0 | 0 | 1.25 |
| ALDH9A1 | 0 | 0 | 0 | -1.54 |
| ALDOC | 0 | 0 | 0 | -1.82 |
| ALKBH7 | 0 | 0 | 0 | -1.32 |
| ALLC | 0 | 0 | 0 | -3.77 |
| ALOX5AP | 0 | 0 | 0 | 2.41 |
| ALPK1 | 0 | 0 | 0 | 1.14 |
| ALPL | 0 | 0 | 0 | -2.89 |
| ALS2 | 0 | 0 | 0 | -1.22 |
| AMACR | 0 | 0 | 0 | -2.88 |
| AMFR | 0 | 0 | 0 | -1.05 |
| AMIGO2 | 0 | 0 | 0 | 2.15 |
| AMIGO3 | 0 | 0 | 0 | 1.29 |
| AMMECR1 | 0 | 0 | 0 | 1.26 |
| AMN | 0 | 0 | 0 | -1.17 |
| AMN1 | 0 | 0 | 0 | 1.13 |
| AMPD3 | 0 | 0 | 0 | 2.34 |
| ANAPC1 | 0 | 0 | 0 | 1.15 |
| ANGPTL1 | 0 | 0 | 0 | -1.65 |
| ANGPTL4 | 0 | 0 | 0 | -2.58 |
| ANK1 | 0 | 0 | 0 | 1.88 |
| ANK2 | 0 | 0 | 0 | -1.29 |
| ANKRD13B | 0 | 0 | 0 | 2.73 |
| ANKRD13D | 0 | 0 | 0 | 1.17 |
| ANKRD18A | 0 | 0 | 0 | 1.46 |
| ANKRD18B | 0 | 0 | 0 | 1.61 |
| ANKRD19P | 0 | 0 | 0 | 1.05 |
| ANKRD20A2 | 0 | 0 | 0 | 1.48 |
| ANKRD20A4 | 0 | 0 | 0 | -2.12 |
| ANKRD24 | 0 | 0 | 0 | -1.21 |
| ANKRD27 | 0 | 0 | 0 | 1.45 |
| ANKRD32 | 0 | 0 | 0 | 1.47 |
| ANKRD34A | 0 | 0 | 0 | 1.18 |
| ANKRD36 | 0 | 0 | 0 | 1.29 |
| ANKRD36BP2 | 0 | 0 | 0 | 1.82 |
| ANKRD43 | 0 | 0 | 0 | -2.33 |
| ANKRD55 | 0 | 0 | 0 | -3.22 |
| ANKRD56 | 0 | 0 | 0 | -2.99 |
| ANKRD57 | 0 | 0 | 0 | -1.02 |
| ANKRD58 | 0 | 0 | 0 | 1.04 |
| ANKS4B | 0 | 0 | 0 | -2.75 |
| ANKS6 | 0 | 0 | 0 | 2.41 |
| ANO10 | 0 | 0 | 0 | 1 |
| ANO4 | 0 | 0 | 0 | 3.3 |
| ANO6 | 0 | 0 | 0 | 1.13 |
| ANO9 | 0 | 0 | 0 | 2.61 |
| ANP32E | 0 | 0 | 0 | 1.1 |
| ANTXR1 | 0 | 0 | 0 | 3.01 |
| ANXA1 | 0 | 0 | 0 | 2.87 |
| ANXA3 | 0 | 0 | 0 | 4.17 |
| ANXA5 | 0 | 0 | 0 | 1.58 |
| ANXA9 | 0 | 0 | 0 | -3.61 |
| AP1M2 | 0 | 0 | 0 | 2.99 |
| AP2B1 | 0 | 0 | 0 | 1.31 |
| AP3B2 | 0 | 0 | 0 | 3.11 |
| AP3M2 | 0 | 0 | 0 | 2.2 |
| AP4B1 | 0 | 0 | 0 | 1.03 |
| APBA1 | 0 | 0 | 0 | -3.03 |
| APC2 | 0 | 0 | 0 | 1.41 |
| APCDD1L | 0 | 0 | 0 | 3.71 |
| APEX2 | 0 | 0 | 0 | 1.09 |
| APH1B | 0 | 0 | 0 | 1.29 |
| APLN | 0 | 0 | 0 | 2.71 |
| APOBEC3C | 0 | 0 | 0 | 2.1 |
| APOBEC3F | 0 | 0 | 0 | 1.31 |
| APOBEC3G | 0 | 0 | 0 | 1.61 |
| APOBEC3H | 0 | 0 | 0 | 2.17 |
| APOL5 | 0 | 0 | 0 | -2.81 |
| AQP1 | 0 | 0 | 0 | 1.55 |
| AQP4 | 0 | 0 | 0 | -2.66 |
| AQPEP | 0 | 0 | 0 | 1.55 |
| AREG | 0 | 0 | 0 | 4.27 |
| ARF3 | 0 | 0 | 0 | 1.1 |
| ARHGAP1 | 0 | 0 | 0 | 1.4 |
| ARHGAP10 | 0 | 0 | 0 | -1.24 |
| ARHGAP11A | 0 | 0 | 0 | 2.21 |
| ARHGAP18 | 0 | 0 | 0 | 2.43 |
| ARHGAP22 | 0 | 0 | 0 | 2.09 |
| ARHGAP23 | 0 | 0 | 0 | 1.53 |
| ARHGAP33 | 0 | 0 | 0 | 1.12 |
| ARHGAP39 | 0 | 0 | 0 | 1.5 |
| ARHGAP4 | 0 | 0 | 0 | 1.49 |
| ARHGAP8 | 0 | 0 | 0 | 3.34 |
| ARHGDIB | 0 | 0 | 0 | 1 |
| ARHGEF1 | 0 | 0 | 0 | 1.26 |
| ARHGEF10L | 0 | 0 | 0 | -1.81 |
| ARHGEF12 | 0 | 0 | 0 | -1 |
| ARHGEF19 | 0 | 0 | 0 | 1.57 |
| ARHGEF2 | 0 | 0 | 0 | 1.84 |
| ARHGEF3 | 0 | 0 | 0 | 1.37 |
| ARHGEF38 | 0 | 0 | 0 | 4.54 |
| ARHGEF4 | 0 | 0 | 0 | 4.18 |
| ARHGEF40 | 0 | 0 | 0 | -1.16 |
| ARID3C | 0 | 0 | 0 | -3.35 |
| ARID5B | 0 | 0 | 0 | 1.55 |
| ARL2 | 0 | 0 | 0 | 1.88 |
| ARL4A | 0 | 0 | 0 | 1.21 |
| ARL4D | 0 | 0 | 0 | -2.5 |
| ARL6 | 0 | 0 | 0 | 1.05 |
| ARL6IP5 | 0 | 0 | 0 | 1.1 |
| ARMC3 | 0 | 0 | 0 | 3.41 |
| ARMC5 | 0 | 0 | 0 | -1.05 |
| ARMC6 | 0 | 0 | 0 | -1.24 |
| ARMCX2 | 0 | 0 | 0 | 1.57 |
| ARPC1B | 0 | 0 | 0 | 1.71 |
| ARPC2 | 0 | 0 | 0 | 1.36 |
| ARPC4-TTLL3 | 0 | 0 | 0 | 1.37 |
| ARPC5 | 0 | 0 | 0 | 1.03 |
| ARPP19 | 0 | 0 | 0 | 1.17 |
| ARPP21 | 0 | 0 | 0 | -3.69 |
| ARSD | 0 | 0 | 0 | -1.29 |
| ARSF | 0 | 0 | 0 | -1.46 |
| ARSJ | 0 | 0 | 0 | 1.71 |
| ART1 | 0 | 0 | 0 | -1.92 |
| ARVCF | 0 | 0 | 0 | -1.38 |
| AS3MT | 0 | 0 | 0 | -3.93 |
| ASAP2 | 0 | 0 | 0 | 2.57 |
| ASB13 | 0 | 0 | 0 | -1.58 |
| ASB14 | 0 | 0 | 0 | 1.17 |
| ASB2 | 0 | 0 | 0 | 1.57 |
| ASB9 | 0 | 0 | 0 | -2 |
| ASPDH | 0 | 0 | 0 | -4.22 |
| ATAD3C | 0 | 0 | 0 | -1.72 |
| ATAD5 | 0 | 0 | 0 | 2.16 |
| ATAT1 | 0 | 0 | 0 | 1.03 |
| ATG2A | 0 | 0 | 0 | -1.22 |
| ATG9B | 0 | 0 | 0 | 2.15 |
| ATL1 | 0 | 0 | 0 | 2.47 |
| ATL3 | 0 | 0 | 0 | 1.5 |
| ATMIN | 0 | 0 | 0 | 1.42 |
| ATN1 | 0 | 0 | 0 | 1.28 |
| ATOH7 | 0 | 0 | 0 | -3.16 |
| ATP10A | 0 | 0 | 0 | 3.41 |
| ATP10B | 0 | 0 | 0 | 3.47 |
| ATP13A2 | 0 | 0 | 0 | 1.25 |
| ATP1B3 | 0 | 0 | 0 | 2.79 |
| ATP2A3 | 0 | 0 | 0 | 1.73 |
| ATP2B2 | 0 | 0 | 0 | -5.38 |
| ATP2B4 | 0 | 0 | 0 | 1 |
| ATP2C1 | 0 | 0 | 0 | 3.11 |
| ATP2C2 | 0 | 0 | 0 | 4.85 |
| ATP5J | 0 | 0 | 0 | -1.03 |
| ATP6V0A4 | 0 | 0 | 0 | 1.85 |
| ATP6V0D2 | 0 | 0 | 0 | 1.61 |
| ATP6V0E2 | 0 | 0 | 0 | -1.66 |
| ATP6V1E2 | 0 | 0 | 0 | 1.29 |
| ATP6V1F | 0 | 0 | 0 | 1.05 |
| ATP8A1 | 0 | 0 | 0 | 1.71 |
| ATP8A2 | 0 | 0 | 0 | 3.91 |
| ATRN | 0 | 0 | 0 | -1.87 |
| ATXN2L | 0 | 0 | 0 | 1.14 |
| AUH | 0 | 0 | 0 | -1.12 |
| AVP | 0 | 0 | 0 | -1 |
| AXDND1 | 0 | 0 | 0 | 2.23 |
| AXIN2 | 0 | 0 | 0 | 1.27 |
| AZI1 | 0 | 0 | 0 | 1.79 |
| B3GALNT1 | 0 | 0 | 0 | 1.2 |
| B3GALT4 | 0 | 0 | 0 | 1.92 |
| B3GALT5 | 0 | 0 | 0 | 3.18 |
| B3GAT1 | 0 | 0 | 0 | -3.03 |
| B3GNT5 | 0 | 0 | 0 | 3.64 |
| B3GNT9 | 0 | 0 | 0 | 1.54 |
| B4GALNT1 | 0 | 0 | 0 | 2.58 |
| B4GALNT3 | 0 | 0 | 0 | 2.8 |
| B4GALNT4 | 0 | 0 | 0 | 2.93 |
| B4GALT4 | 0 | 0 | 0 | 1.08 |
| B7H6 | 0 | 0 | 0 | 1.65 |
| BAG3 | 0 | 0 | 0 | 1.16 |
| BAI2 | 0 | 0 | 0 | 1.52 |
| BAIAP2L2 | 0 | 0 | 0 | 1.96 |
| BAIAP3 | 0 | 0 | 0 | -1.89 |
| BANK1 | 0 | 0 | 0 | 3.05 |
| BARD1 | 0 | 0 | 0 | 2.56 |
| BARX2 | 0 | 0 | 0 | 2.83 |
| BBS12 | 0 | 0 | 0 | 2.16 |
| BBS7 | 0 | 0 | 0 | 1.65 |
| BCAR1 | 0 | 0 | 0 | 1.03 |
| BCAS1 | 0 | 0 | 0 | 5.03 |
| BCAT1 | 0 | 0 | 0 | 2.3 |
| BCAT2 | 0 | 0 | 0 | 1.58 |
| BCKDHA | 0 | 0 | 0 | -1.46 |
| BCKDK | 0 | 0 | 0 | -1.04 |
| BCL11A | 0 | 0 | 0 | 2.59 |
| BCL11B | 0 | 0 | 0 | 2.39 |
| BCL2A1 | 0 | 0 | 0 | 2.5 |
| BCL2L10 | 0 | 0 | 0 | -3.16 |
| BCL2L14 | 0 | 0 | 0 | 2.27 |
| BCL2L15 | 0 | 0 | 0 | 2.22 |
| BCL9 | 0 | 0 | 0 | 1.03 |
| BCL9L | 0 | 0 | 0 | 1.23 |
| BCO2 | 0 | 0 | 0 | -3.94 |
| BDH2 | 0 | 0 | 0 | -2.09 |
| BDKRB1 | 0 | 0 | 0 | 4 |
| BDKRB2 | 0 | 0 | 0 | 3.55 |
| BDNF | 0 | 0 | 0 | 2.6 |
| BEND3 | 0 | 0 | 0 | 1.3 |
| BEND6 | 0 | 0 | 0 | 2.45 |
| BEND7 | 0 | 0 | 0 | -1.74 |
| BFSP2 | 0 | 0 | 0 | 2.8 |
| BICC1 | 0 | 0 | 0 | 1.89 |
| BIK | 0 | 0 | 0 | 4.3 |
| BIRC3 | 0 | 0 | 0 | 2.17 |
| BIRC5 | 0 | 0 | 0 | 3.92 |
| BLVRA | 0 | 0 | 0 | 1.58 |
| BMP10 | 0 | 0 | 0 | -2.31 |
| BMP2 | 0 | 0 | 0 | 1.32 |
| BMP4 | 0 | 0 | 0 | 2.22 |
| BMP5 | 0 | 0 | 0 | -2.47 |
| BMP7 | 0 | 0 | 0 | 2.8 |
| BMP8B | 0 | 0 | 0 | 2.82 |
| BNC2 | 0 | 0 | 0 | 2.54 |
| BNIP3 | 0 | 0 | 0 | -2.76 |
| BOLA2B | 0 | 0 | 0 | 1.44 |
| BPIFB1 | 0 | 0 | 0 | 3.23 |
| BPTF | 0 | 0 | 0 | 1.05 |
| BRCA1 | 0 | 0 | 0 | 1.57 |
| BRCA2 | 0 | 0 | 0 | 2.35 |
| BRCC3 | 0 | 0 | 0 | 1.34 |
| BRIX1 | 0 | 0 | 0 | 1.1 |
| BRMS1 | 0 | 0 | 0 | 1.04 |
| BRP44 | 0 | 0 | 0 | -1.76 |
| BRSK2 | 0 | 0 | 0 | 1.86 |
| BSG | 0 | 0 | 0 | 1.03 |
| BSN | 0 | 0 | 0 | -2.2 |
| BSPRY | 0 | 0 | 0 | 2.86 |
| BTBD10 | 0 | 0 | 0 | 1.26 |
| BTBD8 | 0 | 0 | 0 | 1.23 |
| BTF3L4 | 0 | 0 | 0 | 1.04 |
| BTG3 | 0 | 0 | 0 | 1.92 |
| BTN3A1 | 0 | 0 | 0 | 1.13 |
| BTN3A2 | 0 | 0 | 0 | 1.6 |
| BTNL9 | 0 | 0 | 0 | -2.28 |
| BUB3 | 0 | 0 | 0 | 1 |
| BVES | 0 | 0 | 0 | 1.45 |
| BYSL | 0 | 0 | 0 | 1.02 |
| BZW2 | 0 | 0 | 0 | 1.82 |
| C10orf11 | 0 | 0 | 0 | -1.86 |
| C10orf113 | 0 | 0 | 0 | 2.09 |
| C10orf140 | 0 | 0 | 0 | -3.75 |
| C10orf32 | 0 | 0 | 0 | -1.04 |
| C10orf47 | 0 | 0 | 0 | -2.16 |
| C10orf54 | 0 | 0 | 0 | 1.21 |
| C10orf55 | 0 | 0 | 0 | 2.99 |
| C10orf57 | 0 | 0 | 0 | -1.7 |
| C10orf58 | 0 | 0 | 0 | -1.77 |
| C10orf68 | 0 | 0 | 0 | 1.52 |
| C10orf81 | 0 | 0 | 0 | 4.16 |
| C10orf96 | 0 | 0 | 0 | -1.03 |
| C11orf20 | 0 | 0 | 0 | 2.34 |
| C11orf41 | 0 | 0 | 0 | 3.58 |
| C11orf49 | 0 | 0 | 0 | 1.67 |
| C11orf52 | 0 | 0 | 0 | -1.95 |
| C11orf53 | 0 | 0 | 0 | 2.16 |
| C11orf63 | 0 | 0 | 0 | 2.18 |
| C11orf70 | 0 | 0 | 0 | 3.56 |
| C11orf71 | 0 | 0 | 0 | -1.36 |
| C11orf82 | 0 | 0 | 0 | 2.45 |
| C11orf84 | 0 | 0 | 0 | 1.64 |
| C11orf93 | 0 | 0 | 0 | 2.84 |
| C12orf48 | 0 | 0 | 0 | 2.54 |
| C12orf63 | 0 | 0 | 0 | 3.15 |
| C13orf15 | 0 | 0 | 0 | 2.01 |
| C13orf27 | 0 | 0 | 0 | -1.39 |
| C13orf33 | 0 | 0 | 0 | 3.34 |
| C14orf105 | 0 | 0 | 0 | -2.59 |
| C14orf135 | 0 | 0 | 0 | 1.03 |
| C14orf41 | 0 | 0 | 0 | -1.69 |
| C14orf45 | 0 | 0 | 0 | 1.41 |
| C15orf17 | 0 | 0 | 0 | 1.13 |
| C15orf23 | 0 | 0 | 0 | 1.33 |
| C15orf27 | 0 | 0 | 0 | 1.92 |
| C15orf39 | 0 | 0 | 0 | 1.85 |
| C15orf41 | 0 | 0 | 0 | 1.07 |
| C15orf42 | 0 | 0 | 0 | 5.07 |
| C15orf43 | 0 | 0 | 0 | -1.95 |
| C15orf53 | 0 | 0 | 0 | 2.29 |
| C15orf57 | 0 | 0 | 0 | 1.06 |
| C16orf47 | 0 | 0 | 0 | 1.08 |
| C16orf57 | 0 | 0 | 0 | 1.86 |
| C16orf74 | 0 | 0 | 0 | 2.11 |
| C16orf93 | 0 | 0 | 0 | 1.62 |
| C17orf107 | 0 | 0 | 0 | -1.48 |
| C17orf28 | 0 | 0 | 0 | 2.78 |
| C17orf59 | 0 | 0 | 0 | 1.01 |
| C17orf61 | 0 | 0 | 0 | -1.25 |
| C17orf63 | 0 | 0 | 0 | 1.4 |
| C17orf76 | 0 | 0 | 0 | 2.06 |
| C17orf82 | 0 | 0 | 0 | 1.51 |
| C17orf99 | 0 | 0 | 0 | 4.29 |
| C18orf45 | 0 | 0 | 0 | 1.04 |
| C18orf62 | 0 | 0 | 0 | -1.51 |
| C19orf12 | 0 | 0 | 0 | -1.23 |
| C19orf33 | 0 | 0 | 0 | 6.51 |
| C19orf40 | 0 | 0 | 0 | 1.86 |
| C19orf45 | 0 | 0 | 0 | 1.31 |
| C19orf46 | 0 | 0 | 0 | -1.48 |
| C19orf81 | 0 | 0 | 0 | 1.54 |
| C1orf110 | 0 | 0 | 0 | 1.61 |
| C1orf135 | 0 | 0 | 0 | 2.87 |
| C1orf151-NBL1 | 0 | 0 | 0 | 4.27 |
| C1orf159 | 0 | 0 | 0 | 1.17 |
| C1orf173 | 0 | 0 | 0 | -2.4 |
| C1orf195 | 0 | 0 | 0 | 1.48 |
| C1orf201 | 0 | 0 | 0 | 1.51 |
| C1orf204 | 0 | 0 | 0 | 2.78 |
| C1orf216 | 0 | 0 | 0 | 1.2 |
| C1orf226 | 0 | 0 | 0 | -2.17 |
| C1orf61 | 0 | 0 | 0 | 1.34 |
| C1orf70 | 0 | 0 | 0 | 1.65 |
| C1orf81 | 0 | 0 | 0 | 4.23 |
| C1orf9 | 0 | 0 | 0 | 1.04 |
| C1orf93 | 0 | 0 | 0 | 1.97 |
| C1orf94 | 0 | 0 | 0 | 2.33 |
| C1orf96 | 0 | 0 | 0 | 1.06 |
| C1QTNF4 | 0 | 0 | 0 | -2.77 |
| C1QTNF9 | 0 | 0 | 0 | -1.46 |
| C1QTNF9B-AS1 | 0 | 0 | 0 | 1.67 |
| C1RL | 0 | 0 | 0 | -2.61 |
| C20orf103 | 0 | 0 | 0 | 3.66 |
| C20orf108 | 0 | 0 | 0 | -1.07 |
| C20orf112 | 0 | 0 | 0 | 1.45 |
| C20orf123 | 0 | 0 | 0 | 1.45 |
| C20orf134 | 0 | 0 | 0 | 2.26 |
| C20orf177 | 0 | 0 | 0 | 1.26 |
| C20orf195 | 0 | 0 | 0 | 4.2 |
| C20orf196 | 0 | 0 | 0 | 1.51 |
| C20orf26 | 0 | 0 | 0 | 2.34 |
| C20orf27 | 0 | 0 | 0 | 1.15 |
| C20orf3 | 0 | 0 | 0 | -2.01 |
| C20orf54 | 0 | 0 | 0 | 4.05 |
| C20orf62 | 0 | 0 | 0 | -1.4 |
| C20orf96 | 0 | 0 | 0 | 1.04 |
| C21orf30 | 0 | 0 | 0 | -2.58 |
| C21orf33 | 0 | 0 | 0 | -1.7 |
| C21orf56 | 0 | 0 | 0 | 1.78 |
| C21orf58 | 0 | 0 | 0 | 1.07 |
| C21orf63 | 0 | 0 | 0 | 1.5 |
| C22orf13 | 0 | 0 | 0 | -1.18 |
| C22orf36 | 0 | 0 | 0 | -1.74 |
| C2CD3 | 0 | 0 | 0 | 1.04 |
| C2orf16 | 0 | 0 | 0 | -1.51 |
| C2orf47 | 0 | 0 | 0 | -1.4 |
| C2orf55 | 0 | 0 | 0 | 3.28 |
| C2orf65 | 0 | 0 | 0 | 2.64 |
| C2orf67 | 0 | 0 | 0 | 1.2 |
| C2orf77 | 0 | 0 | 0 | -1.09 |
| C2orf81 | 0 | 0 | 0 | 1.4 |
| C2orf82 | 0 | 0 | 0 | -2.86 |
| C3orf14 | 0 | 0 | 0 | 2.29 |
| C3orf36 | 0 | 0 | 0 | 1.67 |
| C3orf37 | 0 | 0 | 0 | -1.41 |
| C4orf21 | 0 | 0 | 0 | 1.32 |
| C4orf37 | 0 | 0 | 0 | -1.19 |
| C4orf46 | 0 | 0 | 0 | 1.97 |
| C4orf47 | 0 | 0 | 0 | 2.1 |
| C4orf48 | 0 | 0 | 0 | 2.68 |
| C5orf30 | 0 | 0 | 0 | 2.67 |
| C5orf34 | 0 | 0 | 0 | 2.82 |
| C5orf35 | 0 | 0 | 0 | -1.12 |
| C5orf39 | 0 | 0 | 0 | 1.91 |
| C5orf54 | 0 | 0 | 0 | 1.68 |
| C5orf58 | 0 | 0 | 0 | -1.46 |
| C5orf60 | 0 | 0 | 0 | -1.49 |
| C5orf62 | 0 | 0 | 0 | 2.21 |
| C6orf115 | 0 | 0 | 0 | 1.02 |
| C6orf132 | 0 | 0 | 0 | 2.19 |
| C6orf145 | 0 | 0 | 0 | -2.49 |
| C6orf168 | 0 | 0 | 0 | 2 |
| C6orf170 | 0 | 0 | 0 | 2.15 |
| C6orf174 | 0 | 0 | 0 | 2.01 |
| C6orf222 | 0 | 0 | 0 | 5.36 |
| C6orf26 | 0 | 0 | 0 | 1.18 |
| C6orf99 | 0 | 0 | 0 | 2.48 |
| C7orf10 | 0 | 0 | 0 | -2.89 |
| C7orf23 | 0 | 0 | 0 | 1.92 |
| C7orf44 | 0 | 0 | 0 | 1.02 |
| C7orf53 | 0 | 0 | 0 | -1.26 |
| C7orf55 | 0 | 0 | 0 | -1.49 |
| C7orf60 | 0 | 0 | 0 | 1.45 |
| C7orf69 | 0 | 0 | 0 | 2.59 |
| C7orf70 | 0 | 0 | 0 | 1.11 |
| C8orf40 | 0 | 0 | 0 | -1.1 |
| C8orf42 | 0 | 0 | 0 | -1.75 |
| C8orf46 | 0 | 0 | 0 | -4.5 |
| C8orf68 | 0 | 0 | 0 | -1.92 |
| C8orf82 | 0 | 0 | 0 | -1.43 |
| C9orf100 | 0 | 0 | 0 | 2.67 |
| C9orf116 | 0 | 0 | 0 | 1.42 |
| C9orf117 | 0 | 0 | 0 | 2.07 |
| C9orf125 | 0 | 0 | 0 | 2.42 |
| C9orf131 | 0 | 0 | 0 | -1.14 |
| C9orf16 | 0 | 0 | 0 | 1.17 |
| C9orf167 | 0 | 0 | 0 | 2.06 |
| C9orf4 | 0 | 0 | 0 | 1.49 |
| C9orf40 | 0 | 0 | 0 | 1.25 |
| C9orf7 | 0 | 0 | 0 | -1.77 |
| C9orf86 | 0 | 0 | 0 | 1.5 |
| C9orf89 | 0 | 0 | 0 | 1.09 |
| C9orf9 | 0 | 0 | 0 | 1.3 |
| C9orf93 | 0 | 0 | 0 | 1.59 |
| C9orf95 | 0 | 0 | 0 | -1.11 |
| CA11 | 0 | 0 | 0 | 1.07 |
| CA5B | 0 | 0 | 0 | 1.13 |
| CA6 | 0 | 0 | 0 | -1.19 |
| CA8 | 0 | 0 | 0 | 3.17 |
| CA9 | 0 | 0 | 0 | 2.89 |
| CABIN1 | 0 | 0 | 0 | 1.35 |
| CACNA1A | 0 | 0 | 0 | 2.41 |
| CACNA1B | 0 | 0 | 0 | -1.01 |
| CACNA1C | 0 | 0 | 0 | 2.61 |
| CACNA1H | 0 | 0 | 0 | -2.17 |
| CACNA1I | 0 | 0 | 0 | 2.89 |
| CACNA2D1 | 0 | 0 | 0 | 1.25 |
| CACNB1 | 0 | 0 | 0 | 2.51 |
| CACNB4 | 0 | 0 | 0 | 2.87 |
| CACNG4 | 0 | 0 | 0 | 2.59 |
| CACNG6 | 0 | 0 | 0 | 2.51 |
| CAD | 0 | 0 | 0 | 1.47 |
| CADM1 | 0 | 0 | 0 | -2.68 |
| CADPS | 0 | 0 | 0 | 2.34 |
| CALHM3 | 0 | 0 | 0 | 3.45 |
| CALML6 | 0 | 0 | 0 | -1 |
| CAMK1 | 0 | 0 | 0 | -1.17 |
| CAMKK1 | 0 | 0 | 0 | 1.73 |
| CAMSAP2 | 0 | 0 | 0 | 1.03 |
| CAMSAP3 | 0 | 0 | 0 | -1.87 |
| CAMTA1 | 0 | 0 | 0 | 2.08 |
| CANT1 | 0 | 0 | 0 | 1.42 |
| CAP1 | 0 | 0 | 0 | 1.15 |
| CAPN1 | 0 | 0 | 0 | 1.24 |
| CAPN11 | 0 | 0 | 0 | 1.03 |
| CAPN13 | 0 | 0 | 0 | 2.02 |
| CAPN8 | 0 | 0 | 0 | 4.96 |
| CAPNS2 | 0 | 0 | 0 | 1.02 |
| CAPRIN1 | 0 | 0 | 0 | 1.18 |
| CAPZA1 | 0 | 0 | 0 | 1.19 |
| CAPZB | 0 | 0 | 0 | 1.46 |
| CARD16 | 0 | 0 | 0 | 1.21 |
| CARD17 | 0 | 0 | 0 | 1.25 |
| CARM1 | 0 | 0 | 0 | -1.4 |
| CASP1 | 0 | 0 | 0 | 1.52 |
| CASP10 | 0 | 0 | 0 | 1 |
| CASP14L | 0 | 0 | 0 | -3.04 |
| CASP2 | 0 | 0 | 0 | 1.5 |
| CASP4 | 0 | 0 | 0 | 1.28 |
| CASP5 | 0 | 0 | 0 | 1.26 |
| CASP8 | 0 | 0 | 0 | 1.32 |
| CASZ1 | 0 | 0 | 0 | 1.28 |
| CATSPER1 | 0 | 0 | 0 | 3.61 |
| CBFB | 0 | 0 | 0 | 1.37 |
| CBL | 0 | 0 | 0 | 1.1 |
| CBR1 | 0 | 0 | 0 | -1.13 |
| CBR4 | 0 | 0 | 0 | -1.77 |
| CBX2 | 0 | 0 | 0 | 2.22 |
| CBX8 | 0 | 0 | 0 | 1.51 |
| CC2D2A | 0 | 0 | 0 | 1.45 |
| CC2D2B | 0 | 0 | 0 | 1.98 |
| CCBP2 | 0 | 0 | 0 | -2.5 |
| CCDC102A | 0 | 0 | 0 | 1.56 |
| CCDC103 | 0 | 0 | 0 | 1.74 |
| CCDC104 | 0 | 0 | 0 | 1.23 |
| CCDC106 | 0 | 0 | 0 | -1.19 |
| CCDC109B | 0 | 0 | 0 | 2.68 |
| CCDC110 | 0 | 0 | 0 | 1.32 |
| CCDC112 | 0 | 0 | 0 | 2.3 |
| CCDC113 | 0 | 0 | 0 | 2.03 |
| CCDC114 | 0 | 0 | 0 | 2.13 |
| CCDC125 | 0 | 0 | 0 | -1.08 |
| CCDC13 | 0 | 0 | 0 | -1.44 |
| CCDC136 | 0 | 0 | 0 | 1.21 |
| CCDC14 | 0 | 0 | 0 | 1.1 |
| CCDC144A | 0 | 0 | 0 | 1.56 |
| CCDC147 | 0 | 0 | 0 | 1.77 |
| CCDC150 | 0 | 0 | 0 | -1.51 |
| CCDC153 | 0 | 0 | 0 | 1.34 |
| CCDC158 | 0 | 0 | 0 | -3.33 |
| CCDC164 | 0 | 0 | 0 | 3.11 |
| CCDC165 | 0 | 0 | 0 | 2.07 |
| CCDC169 | 0 | 0 | 0 | 1.31 |
| CCDC19 | 0 | 0 | 0 | 3.25 |
| CCDC24 | 0 | 0 | 0 | 1.24 |
| CCDC28B | 0 | 0 | 0 | 1.43 |
| CCDC30 | 0 | 0 | 0 | 1.15 |
| CCDC38 | 0 | 0 | 0 | -4.24 |
| CCDC40 | 0 | 0 | 0 | 1.63 |
| CCDC41 | 0 | 0 | 0 | 1.12 |
| CCDC42 | 0 | 0 | 0 | -1.15 |
| CCDC43 | 0 | 0 | 0 | 1.09 |
| CCDC57 | 0 | 0 | 0 | -1.25 |
| CCDC64 | 0 | 0 | 0 | 2.42 |
| CCDC73 | 0 | 0 | 0 | -1.36 |
| CCDC77 | 0 | 0 | 0 | 1.12 |
| CCDC80 | 0 | 0 | 0 | 3.12 |
| CCDC81 | 0 | 0 | 0 | 1.88 |
| CCDC85A | 0 | 0 | 0 | 1.95 |
| CCDC88B | 0 | 0 | 0 | 1.61 |
| CCDC88C | 0 | 0 | 0 | 1.56 |
| CCDC89 | 0 | 0 | 0 | 1.45 |
| CCDC97 | 0 | 0 | 0 | 1.29 |
| CCL11 | 0 | 0 | 0 | 3.75 |
| CCL13 | 0 | 0 | 0 | 3.55 |
| CCL17 | 0 | 0 | 0 | 1.1 |
| CCL26 | 0 | 0 | 0 | 3.63 |
| CCL8 | 0 | 0 | 0 | 2.44 |
| CCND2 | 0 | 0 | 0 | 2.46 |
| CCNG2 | 0 | 0 | 0 | 1.31 |
| CCNI2 | 0 | 0 | 0 | 3.5 |
| CCNK | 0 | 0 | 0 | 1.07 |
| CCP110 | 0 | 0 | 0 | 1.05 |
| CCR10 | 0 | 0 | 0 | 1.44 |
| CCR4 | 0 | 0 | 0 | 1.72 |
| CCR5 | 0 | 0 | 0 | 1.5 |
| CCR8 | 0 | 0 | 0 | 2.69 |
| CCRL2 | 0 | 0 | 0 | 1.8 |
| CCRN4L | 0 | 0 | 0 | -1.76 |
| CCS | 0 | 0 | 0 | -1.19 |
| CD109 | 0 | 0 | 0 | 3.81 |
| CD14 | 0 | 0 | 0 | -1.81 |
| CD1A | 0 | 0 | 0 | 2.1 |
| CD1D | 0 | 0 | 0 | -1.29 |
| CD2 | 0 | 0 | 0 | 1.52 |
| CD200 | 0 | 0 | 0 | 3.47 |
| CD248 | 0 | 0 | 0 | 2.96 |
| CD27 | 0 | 0 | 0 | 1.61 |
| CD2BP2 | 0 | 0 | 0 | 1.07 |
| CD300LF | 0 | 0 | 0 | 1.88 |
| CD34 | 0 | 0 | 0 | 1.91 |
| CD37 | 0 | 0 | 0 | 1.87 |
| CD3EAP | 0 | 0 | 0 | 1.28 |
| CD44 | 0 | 0 | 0 | 2.42 |
| CD47 | 0 | 0 | 0 | 1.21 |
| CD5 | 0 | 0 | 0 | 1.66 |
| CD55 | 0 | 0 | 0 | 1.95 |
| CD70 | 0 | 0 | 0 | 2.57 |
| CD74 | 0 | 0 | 0 | 1.22 |
| CD8B | 0 | 0 | 0 | 1.7 |
| CD9 | 0 | 0 | 0 | 1.77 |
| CD96 | 0 | 0 | 0 | 1.43 |
| CD97 | 0 | 0 | 0 | 1.56 |
| CDA | 0 | 0 | 0 | -1.91 |
| CDADC1 | 0 | 0 | 0 | -1.04 |
| CDC14A | 0 | 0 | 0 | 1.2 |
| CDC14B | 0 | 0 | 0 | -1.74 |
| CDC25A | 0 | 0 | 0 | 1.24 |
| CDC25B | 0 | 0 | 0 | 1.63 |
| CDC25C | 0 | 0 | 0 | 3.94 |
| CDC34 | 0 | 0 | 0 | -1.04 |
| CDC42 | 0 | 0 | 0 | 1.16 |
| CDC42BPG | 0 | 0 | 0 | 1.72 |
| CDC42EP2 | 0 | 0 | 0 | 1.53 |
| CDC42EP3 | 0 | 0 | 0 | 1.37 |
| CDC42SE2 | 0 | 0 | 0 | 1.15 |
| CDC7 | 0 | 0 | 0 | 2.39 |
| CDCA3 | 0 | 0 | 0 | 1.95 |
| CDCA4 | 0 | 0 | 0 | 3.1 |
| CDCA7 | 0 | 0 | 0 | 6.4 |
| CDCA7L | 0 | 0 | 0 | 1.56 |
| CDH11 | 0 | 0 | 0 | 4.32 |
| CDH17 | 0 | 0 | 0 | 4.27 |
| CDH2 | 0 | 0 | 0 | -2.76 |
| CDH6 | 0 | 0 | 0 | 1.6 |
| CDHR2 | 0 | 0 | 0 | -2.14 |
| CDHR3 | 0 | 0 | 0 | -2.52 |
| CDK2AP1 | 0 | 0 | 0 | 1.21 |
| CDK4 | 0 | 0 | 0 | 1.02 |
| CDK5 | 0 | 0 | 0 | 1.45 |
| CDKL1 | 0 | 0 | 0 | 1.01 |
| CDKN2D | 0 | 0 | 0 | 1.65 |
| CDNF | 0 | 0 | 0 | -2.34 |
| CDR2L | 0 | 0 | 0 | 2.36 |
| CDRT1 | 0 | 0 | 0 | 1.88 |
| CDX2 | 0 | 0 | 0 | 3.69 |
| CDYL | 0 | 0 | 0 | 1.18 |
| CEACAM5 | 0 | 0 | 0 | 6.4 |
| CEACAM6 | 0 | 0 | 0 | 8.29 |
| CEBPA | 0 | 0 | 0 | -3.07 |
| CEBPE | 0 | 0 | 0 | 1.62 |
| CECR6 | 0 | 0 | 0 | 1.05 |
| CECR9 | 0 | 0 | 0 | -1.65 |
| CELF2 | 0 | 0 | 0 | 1.36 |
| CELF4 | 0 | 0 | 0 | 2.07 |
| CELSR2 | 0 | 0 | 0 | 2.13 |
| CELSR3 | 0 | 0 | 0 | 2.09 |
| CENPE | 0 | 0 | 0 | 3.74 |
| CENPH | 0 | 0 | 0 | 3 |
| CENPI | 0 | 0 | 0 | 2.3 |
| CENPJ | 0 | 0 | 0 | 1.26 |
| CENPP | 0 | 0 | 0 | 2.07 |
| CENPQ | 0 | 0 | 0 | 1.15 |
| CEP128 | 0 | 0 | 0 | 1.98 |
| CEP135 | 0 | 0 | 0 | 1.78 |
| CEP152 | 0 | 0 | 0 | 2.4 |
| CEP19 | 0 | 0 | 0 | 1.37 |
| CEP250 | 0 | 0 | 0 | 1.12 |
| CEP290 | 0 | 0 | 0 | 1.18 |
| CEP41 | 0 | 0 | 0 | 2.19 |
| CEP68 | 0 | 0 | 0 | 1.16 |
| CEP97 | 0 | 0 | 0 | 1.84 |
| CERKL | 0 | 0 | 0 | 1.45 |
| CERS2 | 0 | 0 | 0 | -2.13 |
| CES3 | 0 | 0 | 0 | -2.02 |
| CES4A | 0 | 0 | 0 | -2.91 |
| CETN2 | 0 | 0 | 0 | 1.04 |
| CETP | 0 | 0 | 0 | -4.12 |
| CFHR5 | 0 | 0 | 0 | -6.55 |
| CFL1 | 0 | 0 | 0 | 1.48 |
| CFL2 | 0 | 0 | 0 | -2.47 |
| CGB | 0 | 0 | 0 | 1.25 |
| CGNL1 | 0 | 0 | 0 | -3.04 |
| CGREF1 | 0 | 0 | 0 | 1.07 |
| CHAD | 0 | 0 | 0 | -1.77 |
| CHADL | 0 | 0 | 0 | -1.18 |
| CHCHD10 | 0 | 0 | 0 | -1.55 |
| CHD3 | 0 | 0 | 0 | 2.52 |
| CHD5 | 0 | 0 | 0 | 1.43 |
| CHEK2 | 0 | 0 | 0 | 1 |
| CHIC1 | 0 | 0 | 0 | 1.23 |
| CHML | 0 | 0 | 0 | 1.27 |
| CHMP3 | 0 | 0 | 0 | 1.09 |
| CHMP4B | 0 | 0 | 0 | 1.16 |
| CHN1 | 0 | 0 | 0 | 3.13 |
| CHRFAM7A | 0 | 0 | 0 | 1.53 |
| CHRNA3 | 0 | 0 | 0 | 2.4 |
| CHRNA4 | 0 | 0 | 0 | -3.86 |
| CHRNA7 | 0 | 0 | 0 | 3.54 |
| CHRNB1 | 0 | 0 | 0 | -1.04 |
| CHRNE | 0 | 0 | 0 | -1.06 |
| CHST1 | 0 | 0 | 0 | 1.48 |
| CHST10 | 0 | 0 | 0 | 2.33 |
| CHST11 | 0 | 0 | 0 | 2.49 |
| CHST12 | 0 | 0 | 0 | 1.46 |
| CHST13 | 0 | 0 | 0 | -1.04 |
| CHST2 | 0 | 0 | 0 | 2.66 |
| CHST7 | 0 | 0 | 0 | -1.66 |
| CHST9 | 0 | 0 | 0 | -3.95 |
| CHSY1 | 0 | 0 | 0 | 1.42 |
| CIB2 | 0 | 0 | 0 | 2.29 |
| CILP2 | 0 | 0 | 0 | 2.26 |
| CIT | 0 | 0 | 0 | 2.52 |
| CIZ1 | 0 | 0 | 0 | 1.21 |
| CKM | 0 | 0 | 0 | 1.29 |
| CKS2 | 0 | 0 | 0 | 2.4 |
| CLCF1 | 0 | 0 | 0 | 2.29 |
| CLCN4 | 0 | 0 | 0 | -1.09 |
| CLDN1 | 0 | 0 | 0 | -2.52 |
| CLDN11 | 0 | 0 | 0 | 1.97 |
| CLDN14 | 0 | 0 | 0 | -4.62 |
| CLDN18 | 0 | 0 | 0 | 5.55 |
| CLDN4 | 0 | 0 | 0 | 4.34 |
| CLEC11A | 0 | 0 | 0 | 1.36 |
| CLEC18C | 0 | 0 | 0 | 1.57 |
| CLEC2D | 0 | 0 | 0 | 1.02 |
| CLEC5A | 0 | 0 | 0 | 2.45 |
| CLIC1 | 0 | 0 | 0 | 2.12 |
| CLIC3 | 0 | 0 | 0 | 3.57 |
| CLIC5 | 0 | 0 | 0 | 2.07 |
| CLIP2 | 0 | 0 | 0 | 1.81 |
| CLIP3 | 0 | 0 | 0 | 1.43 |
| CLIP4 | 0 | 0 | 0 | 1.77 |
| CLMN | 0 | 0 | 0 | -1.51 |
| CLMP | 0 | 0 | 0 | 3.33 |
| CLOCK | 0 | 0 | 0 | 1.3 |
| CLPX | 0 | 0 | 0 | -1.13 |
| CLSPN | 0 | 0 | 0 | 4.43 |
| CMBL | 0 | 0 | 0 | -2.97 |
| CMIP | 0 | 0 | 0 | 1.4 |
| CMTM1 | 0 | 0 | 0 | 1.4 |
| CMTM7 | 0 | 0 | 0 | 1.92 |
| CMTM8 | 0 | 0 | 0 | -2 |
| CMYA5 | 0 | 0 | 0 | -2.4 |
| CNIH4 | 0 | 0 | 0 | 1.02 |
| CNKSR3 | 0 | 0 | 0 | -1.91 |
| CNOT6 | 0 | 0 | 0 | 1.41 |
| CNPY1 | 0 | 0 | 0 | -1.09 |
| CNPY4 | 0 | 0 | 0 | 1 |
| CNR1 | 0 | 0 | 0 | 2.5 |
| CNTLN | 0 | 0 | 0 | -1.44 |
| CNTN3 | 0 | 0 | 0 | -3.86 |
| CNTRL | 0 | 0 | 0 | 1.03 |
| COBL | 0 | 0 | 0 | -1.2 |
| COG5 | 0 | 0 | 0 | 1.2 |
| COL12A1 | 0 | 0 | 0 | 3.34 |
| COL15A1 | 0 | 0 | 0 | 4.73 |
| COL17A1 | 0 | 0 | 0 | 5.5 |
| COL18A1 | 0 | 0 | 0 | -1.79 |
| COL22A1 | 0 | 0 | 0 | 3.81 |
| COL24A1 | 0 | 0 | 0 | 2.32 |
| COL25A1 | 0 | 0 | 0 | -2.39 |
| COL27A1 | 0 | 0 | 0 | -1.26 |
| COL3A1 | 0 | 0 | 0 | 3.61 |
| COL5A2 | 0 | 0 | 0 | 3.26 |
| COL6A1 | 0 | 0 | 0 | 1.62 |
| COL6A2 | 0 | 0 | 0 | 1.85 |
| COL6A5 | 0 | 0 | 0 | 1.11 |
| COL8A1 | 0 | 0 | 0 | 5.06 |
| COL8A2 | 0 | 0 | 0 | 2.14 |
| COLEC11 | 0 | 0 | 0 | -2.1 |
| COLEC12 | 0 | 0 | 0 | 3.01 |
| COMMD8 | 0 | 0 | 0 | 1.05 |
| COMT | 0 | 0 | 0 | -1.66 |
| COQ10A | 0 | 0 | 0 | -1.71 |
| COTL1 | 0 | 0 | 0 | 1.47 |
| COX6A2 | 0 | 0 | 0 | -3.25 |
| CPA3 | 0 | 0 | 0 | 2.59 |
| CPA5 | 0 | 0 | 0 | 1.07 |
| CPEB4 | 0 | 0 | 0 | -1.06 |
| CPNE5 | 0 | 0 | 0 | 2.41 |
| CPT1A | 0 | 0 | 0 | -1.55 |
| CPT1C | 0 | 0 | 0 | 2.26 |
| CPXM1 | 0 | 0 | 0 | 3.73 |
| CPZ | 0 | 0 | 0 | 3.23 |
| CR2 | 0 | 0 | 0 | 4.67 |
| CRABP1 | 0 | 0 | 0 | 5.62 |
| CRABP2 | 0 | 0 | 0 | 3.12 |
| CRADD | 0 | 0 | 0 | -1.8 |
| CRHR1 | 0 | 0 | 0 | -1.73 |
| CRIP1 | 0 | 0 | 0 | 3.52 |
| CRIP3 | 0 | 0 | 0 | -1.4 |
| CRLF1 | 0 | 0 | 0 | 2.55 |
| CRLF3 | 0 | 0 | 0 | 1.13 |
| CROCC | 0 | 0 | 0 | -1.26 |
| CROCCP2 | 0 | 0 | 0 | -1.49 |
| CROT | 0 | 0 | 0 | -1.44 |
| CRP | 0 | 0 | 0 | -5.83 |
| CRYGN | 0 | 0 | 0 | 2.03 |
| CRYM | 0 | 0 | 0 | -2.36 |
| CRYZ | 0 | 0 | 0 | -2.48 |
| CS | 0 | 0 | 0 | 1.09 |
| CSAD | 0 | 0 | 0 | -2.2 |
| CSDA | 0 | 0 | 0 | 2.64 |
| CSE1L | 0 | 0 | 0 | 1.03 |
| CSF2RA | 0 | 0 | 0 | 1.67 |
| CSGALNACT2 | 0 | 0 | 0 | 1.14 |
| CSMD2 | 0 | 0 | 0 | 1.72 |
| CSTF2 | 0 | 0 | 0 | 1.5 |
| CT45A1 | 0 | 0 | 0 | 1.26 |
| CT45A5 | 0 | 0 | 0 | 1.32 |
| CTAGE1 | 0 | 0 | 0 | -1.45 |
| CTAGE15P | 0 | 0 | 0 | -1.05 |
| CTAGE5 | 0 | 0 | 0 | -1.64 |
| CTBP2 | 0 | 0 | 0 | 1.08 |
| CTBS | 0 | 0 | 0 | -1.03 |
| CTGF | 0 | 0 | 0 | 2.75 |
| CTHRC1 | 0 | 0 | 0 | 6.47 |
| CTIF | 0 | 0 | 0 | -1 |
| CTLA4 | 0 | 0 | 0 | 3.36 |
| CTNNA3 | 0 | 0 | 0 | -3.95 |
| CTPS | 0 | 0 | 0 | -1.25 |
| CTSC | 0 | 0 | 0 | 1.64 |
| CTSE | 0 | 0 | 0 | 4.41 |
| CTSK | 0 | 0 | 0 | 2.45 |
| CTTNBP2NL | 0 | 0 | 0 | 1.71 |
| CUBN | 0 | 0 | 0 | 1.62 |
| CUEDC1 | 0 | 0 | 0 | 1.49 |
| CWC27 | 0 | 0 | 0 | 1.02 |
| CXCL13 | 0 | 0 | 0 | 3.89 |
| CXCL17 | 0 | 0 | 0 | 3.79 |
| CXCL3 | 0 | 0 | 0 | 3.67 |
| CXCL5 | 0 | 0 | 0 | 3.68 |
| CXCR3 | 0 | 0 | 0 | 2.1 |
| CXCR4 | 0 | 0 | 0 | 2.63 |
| CXCR5 | 0 | 0 | 0 | 1.78 |
| CXCR7 | 0 | 0 | 0 | 1.58 |
| CXorf23 | 0 | 0 | 0 | 1.04 |
| CXorf36 | 0 | 0 | 0 | 1.64 |
| CXorf49B | 0 | 0 | 0 | -1.18 |
| CXorf64 | 0 | 0 | 0 | -1.54 |
| CXorf65 | 0 | 0 | 0 | 1.7 |
| CXorf66 | 0 | 0 | 0 | -3.77 |
| CXorf68 | 0 | 0 | 0 | -1.41 |
| CXXC5 | 0 | 0 | 0 | -1.1 |
| CYB5D2 | 0 | 0 | 0 | -1.23 |
| CYB5R4 | 0 | 0 | 0 | 1.03 |
| CYBRD1 | 0 | 0 | 0 | 1.32 |
| CYFIP2 | 0 | 0 | 0 | -1.64 |
| CYP11A1 | 0 | 0 | 0 | -1.22 |
| CYP17A1 | 0 | 0 | 0 | -1.12 |
| CYP1A1 | 0 | 0 | 0 | -3.58 |
| CYP2S1 | 0 | 0 | 0 | 2.48 |
| CYP4B1 | 0 | 0 | 0 | 2.64 |
| CYP4F22 | 0 | 0 | 0 | -3.94 |
| CYP4Z1 | 0 | 0 | 0 | -2.62 |
| CYSLTR2 | 0 | 0 | 0 | 2.13 |
| CYTH3 | 0 | 0 | 0 | 1.7 |
| DAGLA | 0 | 0 | 0 | 1.45 |
| DAND5 | 0 | 0 | 0 | 2.07 |
| DAPK2 | 0 | 0 | 0 | 1.32 |
| DAPL1 | 0 | 0 | 0 | 3.21 |
| DAPP1 | 0 | 0 | 0 | 2.06 |
| DBF4 | 0 | 0 | 0 | 1.74 |
| DBF4B | 0 | 0 | 0 | 1.96 |
| DBT | 0 | 0 | 0 | -1.34 |
| DCAF11 | 0 | 0 | 0 | -2.16 |
| DCAF13 | 0 | 0 | 0 | 1.03 |
| DCAF15 | 0 | 0 | 0 | 1.01 |
| DCAF16 | 0 | 0 | 0 | 1.46 |
| DCAF4L2 | 0 | 0 | 0 | -4.59 |
| DCAF6 | 0 | 0 | 0 | -1.17 |
| DCAF8L2 | 0 | 0 | 0 | 2.21 |
| DCDC1 | 0 | 0 | 0 | -1.8 |
| DCDC2C | 0 | 0 | 0 | -1.22 |
| DCDC5 | 0 | 0 | 0 | -4.76 |
| DCK | 0 | 0 | 0 | 1.74 |
| DCLK1 | 0 | 0 | 0 | 2.64 |
| DCLRE1B | 0 | 0 | 0 | 1.67 |
| DCLRE1C | 0 | 0 | 0 | 1.4 |
| DCPS | 0 | 0 | 0 | -1.91 |
| DCTPP1 | 0 | 0 | 0 | 1.43 |
| DCUN1D4 | 0 | 0 | 0 | -1.09 |
| DCUN1D5 | 0 | 0 | 0 | 1.12 |
| DDAH1 | 0 | 0 | 0 | 1.97 |
| DDHD1 | 0 | 0 | 0 | 1.29 |
| DDO | 0 | 0 | 0 | -1.77 |
| DDTL | 0 | 0 | 0 | -2.52 |
| DDX11 | 0 | 0 | 0 | 1.04 |
| DDX26B | 0 | 0 | 0 | 1.83 |
| DDX27 | 0 | 0 | 0 | 1.27 |
| DDX50 | 0 | 0 | 0 | 1.16 |
| DDX60 | 0 | 0 | 0 | 1.09 |
| DDX60L | 0 | 0 | 0 | 1.02 |
| DECR1 | 0 | 0 | 0 | -2.19 |
| DEF6 | 0 | 0 | 0 | 1.2 |
| DEFA7P | 0 | 0 | 0 | -1.32 |
| DEFB1 | 0 | 0 | 0 | -3.22 |
| DENND1C | 0 | 0 | 0 | 1.46 |
| DENND5B | 0 | 0 | 0 | -1.44 |
| DERA | 0 | 0 | 0 | -1.15 |
| DERL3 | 0 | 0 | 0 | 1.66 |
| DFNA5 | 0 | 0 | 0 | 1.27 |
| DFNB31 | 0 | 0 | 0 | 1.67 |
| DGKA | 0 | 0 | 0 | 2.84 |
| DGKH | 0 | 0 | 0 | 2.43 |
| DGKI | 0 | 0 | 0 | 2.06 |
| DGKQ | 0 | 0 | 0 | 1.22 |
| DGKZ | 0 | 0 | 0 | 1.48 |
| DHCR24 | 0 | 0 | 0 | -3.33 |
| DHCR7 | 0 | 0 | 0 | -1.55 |
| DHRS2 | 0 | 0 | 0 | -3.46 |
| DHRS4L1 | 0 | 0 | 0 | -1.71 |
| DHRS9 | 0 | 0 | 0 | 2.26 |
| DHX57 | 0 | 0 | 0 | 1.06 |
| DIO3 | 0 | 0 | 0 | 2.57 |
| DIRAS1 | 0 | 0 | 0 | 1.71 |
| DKC1 | 0 | 0 | 0 | 1.22 |
| DKFZP586K1520 | 0 | 0 | 0 | 1.73 |
| DKFZp686L13185 | 0 | 0 | 0 | 1.67 |
| DKK2 | 0 | 0 | 0 | 3.46 |
| DKK3 | 0 | 0 | 0 | 1.62 |
| DLC1 | 0 | 0 | 0 | -1.11 |
| DLEC1 | 0 | 0 | 0 | 1.5 |
| DLG3 | 0 | 0 | 0 | 2.95 |
| DLG5 | 0 | 0 | 0 | 1.86 |
| DLGAP2 | 0 | 0 | 0 | -1.03 |
| DLK2 | 0 | 0 | 0 | 1.67 |
| DLL3 | 0 | 0 | 0 | 2.6 |
| DLX4 | 0 | 0 | 0 | 3.22 |
| DLX5 | 0 | 0 | 0 | 3.1 |
| DMBT1 | 0 | 0 | 0 | 4.31 |
| DMBX1 | 0 | 0 | 0 | 2.08 |
| DMC1 | 0 | 0 | 0 | 1.1 |
| DMD | 0 | 0 | 0 | -2.86 |
| DMRT2 | 0 | 0 | 0 | -1.1 |
| DMRTA1 | 0 | 0 | 0 | -2.69 |
| DMRTA2 | 0 | 0 | 0 | 2.51 |
| DMRTC1 | 0 | 0 | 0 | 1.69 |
| DNAH17 | 0 | 0 | 0 | 2.35 |
| DNAH6 | 0 | 0 | 0 | -2.57 |
| DNAH7 | 0 | 0 | 0 | 2.66 |
| DNAI2 | 0 | 0 | 0 | 2.26 |
| DNAJA4 | 0 | 0 | 0 | 2.9 |
| DNAJB3 | 0 | 0 | 0 | -3.44 |
| DNAJB6 | 0 | 0 | 0 | 1.14 |
| DNAJB9 | 0 | 0 | 0 | -1.21 |
| DNAJC10 | 0 | 0 | 0 | 1.62 |
| DNAJC12 | 0 | 0 | 0 | -2.83 |
| DNAJC18 | 0 | 0 | 0 | 1.49 |
| DNAJC19 | 0 | 0 | 0 | -1.19 |
| DNAJC2 | 0 | 0 | 0 | 1.01 |
| DNAJC22 | 0 | 0 | 0 | -2.27 |
| DNAJC28 | 0 | 0 | 0 | -1.42 |
| DNAJC9 | 0 | 0 | 0 | 1.25 |
| DNAL1 | 0 | 0 | 0 | 1.44 |
| DNALI1 | 0 | 0 | 0 | -2.12 |
| DNASE1 | 0 | 0 | 0 | 1.76 |
| DNM3 | 0 | 0 | 0 | 2.75 |
| DNMT1 | 0 | 0 | 0 | 1.69 |
| DNMT3A | 0 | 0 | 0 | 1.11 |
| DNMT3B | 0 | 0 | 0 | 1.4 |
| DNTTIP1 | 0 | 0 | 0 | 1.01 |
| DOC2A | 0 | 0 | 0 | 2.15 |
| DOC2B | 0 | 0 | 0 | 1.3 |
| DOCK11 | 0 | 0 | 0 | 1.62 |
| DOCK8 | 0 | 0 | 0 | 1.18 |
| DOK1 | 0 | 0 | 0 | 1.5 |
| DOK3 | 0 | 0 | 0 | 1.55 |
| DPEP1 | 0 | 0 | 0 | 2.98 |
| DPPA4 | 0 | 0 | 0 | -1.83 |
| DPY19L2 | 0 | 0 | 0 | 1.36 |
| DPYSL3 | 0 | 0 | 0 | 3.07 |
| DPYSL4 | 0 | 0 | 0 | 3.02 |
| DQX1 | 0 | 0 | 0 | 3.79 |
| DR1 | 0 | 0 | 0 | 1.08 |
| DRAM1 | 0 | 0 | 0 | 1.84 |
| DRD2 | 0 | 0 | 0 | 2.09 |
| DSC2 | 0 | 0 | 0 | 1.22 |
| DSC3 | 0 | 0 | 0 | 3.49 |
| DSCAM | 0 | 0 | 0 | -2.17 |
| DSCC1 | 0 | 0 | 0 | 2.38 |
| DSE | 0 | 0 | 0 | 1.2 |
| DSG1 | 0 | 0 | 0 | -2.15 |
| DSG3 | 0 | 0 | 0 | 5.25 |
| DSPP | 0 | 0 | 0 | 2.46 |
| DTD1 | 0 | 0 | 0 | 1.46 |
| DTX1 | 0 | 0 | 0 | -3.21 |
| DUOX1 | 0 | 0 | 0 | 3.48 |
| DUOX2 | 0 | 0 | 0 | 2.49 |
| DUOXA1 | 0 | 0 | 0 | 2.74 |
| DUOXA2 | 0 | 0 | 0 | 2.67 |
| DUSP10 | 0 | 0 | 0 | -1.68 |
| DUSP16 | 0 | 0 | 0 | -1.29 |
| DUSP18 | 0 | 0 | 0 | 2.16 |
| DUSP6 | 0 | 0 | 0 | -1.08 |
| DUSP7 | 0 | 0 | 0 | 1.39 |
| DUT | 0 | 0 | 0 | 1 |
| DYNC1H1 | 0 | 0 | 0 | 1.25 |
| DYNC2H1 | 0 | 0 | 0 | 1.73 |
| DYNLT1 | 0 | 0 | 0 | 1.23 |
| DYRK2 | 0 | 0 | 0 | 1.87 |
| DYX1C1 | 0 | 0 | 0 | 1.24 |
| DZANK1 | 0 | 0 | 0 | 1.2 |
| DZIP1L | 0 | 0 | 0 | 2.38 |
| E2F1 | 0 | 0 | 0 | 3.68 |
| E2F4 | 0 | 0 | 0 | 1.06 |
| E2F5 | 0 | 0 | 0 | 1.91 |
| E2F8 | 0 | 0 | 0 | 4.64 |
| EAF2 | 0 | 0 | 0 | 1.84 |
| ECEL1 | 0 | 0 | 0 | 2.44 |
| ECHDC3 | 0 | 0 | 0 | -3.85 |
| ECI1 | 0 | 0 | 0 | -1.54 |
| ECM2 | 0 | 0 | 0 | -2.43 |
| EDEM1 | 0 | 0 | 0 | -1.01 |
| EDN1 | 0 | 0 | 0 | 2.38 |
| EDNRA | 0 | 0 | 0 | 1.22 |
| EED | 0 | 0 | 0 | 1.09 |
| EEF1E1 | 0 | 0 | 0 | 1.07 |
| EFCAB1 | 0 | 0 | 0 | -1.39 |
| EFCAB11 | 0 | 0 | 0 | 1.12 |
| EFCAB4B | 0 | 0 | 0 | 1.01 |
| EFCAB7 | 0 | 0 | 0 | 1.09 |
| EFEMP1 | 0 | 0 | 0 | 2.72 |
| EFEMP2 | 0 | 0 | 0 | 1.46 |
| EFHA2 | 0 | 0 | 0 | -2.3 |
| EFHC1 | 0 | 0 | 0 | 1.46 |
| EFHD2 | 0 | 0 | 0 | 1.62 |
| EFNA5 | 0 | 0 | 0 | 2.53 |
| EFNB1 | 0 | 0 | 0 | 1.05 |
| EFNB2 | 0 | 0 | 0 | 1.77 |
| EGFR | 0 | 0 | 0 | -1.34 |
| EGR4 | 0 | 0 | 0 | 3.33 |
| EHBP1 | 0 | 0 | 0 | -1.04 |
| EHBP1L1 | 0 | 0 | 0 | 1.46 |
| EHD2 | 0 | 0 | 0 | 1.98 |
| EHD3 | 0 | 0 | 0 | -1.28 |
| EHMT2 | 0 | 0 | 0 | 1.15 |
| EID2B | 0 | 0 | 0 | 1.25 |
| EID3 | 0 | 0 | 0 | 1.15 |
| EIF2C2 | 0 | 0 | 0 | 1.08 |
| EIF3D | 0 | 0 | 0 | 1.04 |
| EIF3H | 0 | 0 | 0 | 1.05 |
| EIF4A3 | 0 | 0 | 0 | 1.11 |
| EIF4EBP2 | 0 | 0 | 0 | -1.06 |
| EIF5A2 | 0 | 0 | 0 | 2.25 |
| EIF5AL1 | 0 | 0 | 0 | -2.09 |
| ELF4 | 0 | 0 | 0 | 2.6 |
| ELK3 | 0 | 0 | 0 | 1.37 |
| ELL3 | 0 | 0 | 0 | 1.72 |
| ELOVL4 | 0 | 0 | 0 | 1.78 |
| ELOVL7 | 0 | 0 | 0 | 2.69 |
| EMB | 0 | 0 | 0 | 1.8 |
| EME2 | 0 | 0 | 0 | 1.24 |
| EMILIN2 | 0 | 0 | 0 | 2.96 |
| EML2 | 0 | 0 | 0 | 1.62 |
| EMP3 | 0 | 0 | 0 | 1.69 |
| EN1 | 0 | 0 | 0 | 1.42 |
| ENDOD1 | 0 | 0 | 0 | 1.6 |
| ENDOG | 0 | 0 | 0 | -1.15 |
| ENO1 | 0 | 0 | 0 | 1.24 |
| ENOPH1 | 0 | 0 | 0 | 1.15 |
| ENOSF1 | 0 | 0 | 0 | -1.09 |
| ENOX1 | 0 | 0 | 0 | 3.41 |
| ENPP3 | 0 | 0 | 0 | -3.81 |
| ENPP5 | 0 | 0 | 0 | 2.27 |
| ENTPD1 | 0 | 0 | 0 | 1.99 |
| ENTPD3 | 0 | 0 | 0 | 3.24 |
| EPHB2 | 0 | 0 | 0 | 2.86 |
| EPHB3 | 0 | 0 | 0 | 1.85 |
| EPHB6 | 0 | 0 | 0 | 1.14 |
| EPHX4 | 0 | 0 | 0 | 3.84 |
| EPN3 | 0 | 0 | 0 | 4.09 |
| EPO | 0 | 0 | 0 | -2.69 |
| EPOR | 0 | 0 | 0 | -1.21 |
| EPPK1 | 0 | 0 | 0 | 2.1 |
| EPRS | 0 | 0 | 0 | 1.12 |
| EPS8L3 | 0 | 0 | 0 | 1.58 |
| EPSTI1 | 0 | 0 | 0 | 1.68 |
| EPYC | 0 | 0 | 0 | 1.74 |
| ERBB3 | 0 | 0 | 0 | -1.86 |
| ERC2 | 0 | 0 | 0 | 3.5 |
| ERCC6L | 0 | 0 | 0 | 2.14 |
| EREG | 0 | 0 | 0 | 3.28 |
| ERI1 | 0 | 0 | 0 | 1.22 |
| ERLEC1 | 0 | 0 | 0 | 1.06 |
| ERMN | 0 | 0 | 0 | 1.66 |
| ERN2 | 0 | 0 | 0 | 5.97 |
| ERO1LB | 0 | 0 | 0 | -1.91 |
| ERRFI1 | 0 | 0 | 0 | -2.5 |
| ERV3-1 | 0 | 0 | 0 | 1.63 |
| ERVFRD-1 | 0 | 0 | 0 | -1.84 |
| ERVMER34-1 | 0 | 0 | 0 | 2.13 |
| ESCO2 | 0 | 0 | 0 | 3.23 |
| ESR2 | 0 | 0 | 0 | 1.75 |
| ESRP1 | 0 | 0 | 0 | 3.83 |
| ESYT3 | 0 | 0 | 0 | -1.07 |
| ETFA | 0 | 0 | 0 | -1.48 |
| ETS2 | 0 | 0 | 0 | -1.19 |
| ETV4 | 0 | 0 | 0 | 1.9 |
| ETV6 | 0 | 0 | 0 | 1.31 |
| ETV7 | 0 | 0 | 0 | 2.87 |
| EVI2A | 0 | 0 | 0 | 1.25 |
| EVPLL | 0 | 0 | 0 | -1.29 |
| EXO1 | 0 | 0 | 0 | 4.2 |
| EXOC1 | 0 | 0 | 0 | 1.08 |
| EXOC6B | 0 | 0 | 0 | 1.04 |
| EXOG | 0 | 0 | 0 | 1.3 |
| EXPH5 | 0 | 0 | 0 | -1.6 |
| EYA1 | 0 | 0 | 0 | -1.82 |
| EYS | 0 | 0 | 0 | -2.06 |
| EZR | 0 | 0 | 0 | 2.73 |
| F13A1 | 0 | 0 | 0 | 3.12 |
| F2RL3 | 0 | 0 | 0 | 1.42 |
| F3 | 0 | 0 | 0 | 3.91 |
| F8 | 0 | 0 | 0 | -1.31 |
| FA2H | 0 | 0 | 0 | 6.38 |
| FAAH | 0 | 0 | 0 | -2.05 |
| FAAH2 | 0 | 0 | 0 | -1.41 |
| FABP5 | 0 | 0 | 0 | 2.83 |
| FADS6 | 0 | 0 | 0 | -2.5 |
| FAHD1 | 0 | 0 | 0 | -1.65 |
| FAIM2 | 0 | 0 | 0 | 1.73 |
| FAM101A | 0 | 0 | 0 | 3.43 |
| FAM102B | 0 | 0 | 0 | 1.46 |
| FAM105A | 0 | 0 | 0 | 1.71 |
| FAM110A | 0 | 0 | 0 | 1.81 |
| FAM110B | 0 | 0 | 0 | -1.95 |
| FAM111A | 0 | 0 | 0 | 1.15 |
| FAM111B | 0 | 0 | 0 | 2.25 |
| FAM113B | 0 | 0 | 0 | 1.14 |
| FAM115C | 0 | 0 | 0 | 1.72 |
| FAM118A | 0 | 0 | 0 | 1.28 |
| FAM118B | 0 | 0 | 0 | 1.04 |
| FAM120A | 0 | 0 | 0 | 1.16 |
| FAM122B | 0 | 0 | 0 | 1.18 |
| FAM122C | 0 | 0 | 0 | 1.09 |
| FAM127C | 0 | 0 | 0 | 1.61 |
| FAM129B | 0 | 0 | 0 | 2.4 |
| FAM131B | 0 | 0 | 0 | 2.24 |
| FAM132B | 0 | 0 | 0 | 1.48 |
| FAM135A | 0 | 0 | 0 | 1.83 |
| FAM155A | 0 | 0 | 0 | 2.77 |
| FAM157B | 0 | 0 | 0 | 1.86 |
| FAM159A | 0 | 0 | 0 | 1.85 |
| FAM160B2 | 0 | 0 | 0 | 1.08 |
| FAM164A | 0 | 0 | 0 | 2.13 |
| FAM164C | 0 | 0 | 0 | -1.72 |
| FAM170B | 0 | 0 | 0 | -1.96 |
| FAM176B | 0 | 0 | 0 | 1.5 |
| FAM183A | 0 | 0 | 0 | 3.28 |
| FAM184A | 0 | 0 | 0 | -1.23 |
| FAM188B | 0 | 0 | 0 | 1.39 |
| FAM190A | 0 | 0 | 0 | 2.77 |
| FAM195A | 0 | 0 | 0 | -1.58 |
| FAM19A4 | 0 | 0 | 0 | -3.9 |
| FAM206A | 0 | 0 | 0 | 1.3 |
| FAM208A | 0 | 0 | 0 | 1.17 |
| FAM20C | 0 | 0 | 0 | -1.21 |
| FAM24B | 0 | 0 | 0 | 1.9 |
| FAM26E | 0 | 0 | 0 | 1.87 |
| FAM27A | 0 | 0 | 0 | 1.61 |
| FAM27E3 | 0 | 0 | 0 | 2.02 |
| FAM35A | 0 | 0 | 0 | -1.19 |
| FAM3D | 0 | 0 | 0 | 3.99 |
| FAM40B | 0 | 0 | 0 | 2.86 |
| FAM49B | 0 | 0 | 0 | 1.5 |
| FAM54A | 0 | 0 | 0 | 3.81 |
| FAM59B | 0 | 0 | 0 | 3.13 |
| FAM63B | 0 | 0 | 0 | 1.35 |
| FAM65C | 0 | 0 | 0 | -2.46 |
| FAM69B | 0 | 0 | 0 | -1.43 |
| FAM70A | 0 | 0 | 0 | 3.11 |
| FAM71E1 | 0 | 0 | 0 | 1.05 |
| FAM72A | 0 | 0 | 0 | 3.22 |
| FAM72D | 0 | 0 | 0 | 3.29 |
| FAM75C2 | 0 | 0 | 0 | -1.32 |
| FAM81B | 0 | 0 | 0 | 3.25 |
| FAM82A1 | 0 | 0 | 0 | -2.13 |
| FAM83A | 0 | 0 | 0 | 3.75 |
| FAM83D | 0 | 0 | 0 | 2.37 |
| FAM83E | 0 | 0 | 0 | 3.73 |
| FAM8A1 | 0 | 0 | 0 | -1.79 |
| FAM90A1 | 0 | 0 | 0 | 2.2 |
| FAM92B | 0 | 0 | 0 | 2.16 |
| FAM98B | 0 | 0 | 0 | 1.1 |
| FANCA | 0 | 0 | 0 | 1.1 |
| FANCB | 0 | 0 | 0 | 1.8 |
| FANCE | 0 | 0 | 0 | 2.06 |
| FANCG | 0 | 0 | 0 | 1.65 |
| FANCM | 0 | 0 | 0 | 1.06 |
| FAR1 | 0 | 0 | 0 | 1.85 |
| FAR2 | 0 | 0 | 0 | 2.25 |
| FARP1 | 0 | 0 | 0 | -1.17 |
| FASN | 0 | 0 | 0 | -1.42 |
| FBF1 | 0 | 0 | 0 | 1.42 |
| FBL | 0 | 0 | 0 | 1.31 |
| FBLIM1 | 0 | 0 | 0 | 2.1 |
| FBLN2 | 0 | 0 | 0 | 1.97 |
| FBLN7 | 0 | 0 | 0 | -1.27 |
| FBN1 | 0 | 0 | 0 | 2.17 |
| FBN2 | 0 | 0 | 0 | 1.88 |
| FBXL16 | 0 | 0 | 0 | 3.21 |
| FBXL22 | 0 | 0 | 0 | 1.76 |
| FBXO25 | 0 | 0 | 0 | -1.1 |
| FBXO27 | 0 | 0 | 0 | -1.38 |
| FBXO31 | 0 | 0 | 0 | -1.13 |
| FBXO5 | 0 | 0 | 0 | 1.76 |
| FBXW9 | 0 | 0 | 0 | 1.16 |
| FCAMR | 0 | 0 | 0 | -3.8 |
| FCGBP | 0 | 0 | 0 | 1.72 |
| FCGR1B | 0 | 0 | 0 | 2.37 |
| FCGR2A | 0 | 0 | 0 | 2.06 |
| FCGRT | 0 | 0 | 0 | -1.18 |
| FCHO1 | 0 | 0 | 0 | 1.52 |
| FCRL5 | 0 | 0 | 0 | 4.3 |
| FECH | 0 | 0 | 0 | -1.38 |
| FERMT3 | 0 | 0 | 0 | 1.17 |
| FGD1 | 0 | 0 | 0 | 1.23 |
| FGF11 | 0 | 0 | 0 | 2.13 |
| FGF14 | 0 | 0 | 0 | -1.28 |
| FGF18 | 0 | 0 | 0 | 1.18 |
| FGF21 | 0 | 0 | 0 | -3.23 |
| FGF9 | 0 | 0 | 0 | 3.71 |
| FGFBP1 | 0 | 0 | 0 | 6.52 |
| FGFBP3 | 0 | 0 | 0 | 1.16 |
| FGFR3 | 0 | 0 | 0 | -3.05 |
| FGFR4 | 0 | 0 | 0 | -1.18 |
| FGFRL1 | 0 | 0 | 0 | -1.93 |
| FH | 0 | 0 | 0 | -2.12 |
| FHAD1 | 0 | 0 | 0 | 3.07 |
| FHDC1 | 0 | 0 | 0 | 3.6 |
| FHL2 | 0 | 0 | 0 | 2.83 |
| FHL3 | 0 | 0 | 0 | 1.05 |
| FHOD1 | 0 | 0 | 0 | 1.72 |
| FIBIN | 0 | 0 | 0 | 1.87 |
| FIG4 | 0 | 0 | 0 | 1.13 |
| FIGN | 0 | 0 | 0 | -1.57 |
| FIGNL1 | 0 | 0 | 0 | 1.45 |
| FIGNL2 | 0 | 0 | 0 | -1.76 |
| FILIP1L | 0 | 0 | 0 | 1.83 |
| FJX1 | 0 | 0 | 0 | 1.74 |
| FKBP1A | 0 | 0 | 0 | 1.12 |
| FKBP9 | 0 | 0 | 0 | 1.53 |
| FKTN | 0 | 0 | 0 | 1.07 |
| FLJ11710 | 0 | 0 | 0 | 1.19 |
| FLJ12334 | 0 | 0 | 0 | 1.29 |
| FLJ13744 | 0 | 0 | 0 | 2.74 |
| FLJ20518 | 0 | 0 | 0 | 1.7 |
| FLJ25917 | 0 | 0 | 0 | 4.66 |
| FLJ31356 | 0 | 0 | 0 | 1.81 |
| FLJ31713 | 0 | 0 | 0 | -1.49 |
| FLJ32224 | 0 | 0 | 0 | 1.09 |
| FLJ32255 | 0 | 0 | 0 | 2.41 |
| FLJ33996 | 0 | 0 | 0 | -1.84 |
| FLJ36848 | 0 | 0 | 0 | -2.06 |
| FLJ37798 | 0 | 0 | 0 | -1.16 |
| FLJ38109 | 0 | 0 | 0 | 1.61 |
| FLJ39051 | 0 | 0 | 0 | 1.77 |
| FLJ41455 | 0 | 0 | 0 | 1.58 |
| FLJ42280 | 0 | 0 | 0 | -3.61 |
| FLJ45139 | 0 | 0 | 0 | -4.57 |
| FLJ46875 | 0 | 0 | 0 | 1.02 |
| FLNA | 0 | 0 | 0 | 2.15 |
| FLT3LG | 0 | 0 | 0 | 1.04 |
| FLT4 | 0 | 0 | 0 | -1.48 |
| FLVCR1 | 0 | 0 | 0 | 1.59 |
| FMN1 | 0 | 0 | 0 | 1.51 |
| FMNL1 | 0 | 0 | 0 | 1.62 |
| FMOD | 0 | 0 | 0 | 2.23 |
| FMR1NB | 0 | 0 | 0 | -1.22 |
| FN3K | 0 | 0 | 0 | -2.17 |
| FNDC5 | 0 | 0 | 0 | -4.8 |
| FOSL2 | 0 | 0 | 0 | 2.07 |
| FOXC2 | 0 | 0 | 0 | 2.2 |
| FOXD1 | 0 | 0 | 0 | 3.46 |
| FOXD4 | 0 | 0 | 0 | 1.32 |
| FOXJ2 | 0 | 0 | 0 | 1.01 |
| FOXK1 | 0 | 0 | 0 | 1.47 |
| FOXK2 | 0 | 0 | 0 | 1.16 |
| FOXL1 | 0 | 0 | 0 | 4.41 |
| FOXL2 | 0 | 0 | 0 | 1.41 |
| FOXN4 | 0 | 0 | 0 | -3.53 |
| FOXP2 | 0 | 0 | 0 | -1.93 |
| FOXRED1 | 0 | 0 | 0 | -1.33 |
| FOXS1 | 0 | 0 | 0 | 1.99 |
| FRAS1 | 0 | 0 | 0 | 2.8 |
| FRAT1 | 0 | 0 | 0 | -1.41 |
| FREM2 | 0 | 0 | 0 | -2.39 |
| FRMD3 | 0 | 0 | 0 | 1.53 |
| FRMD4B | 0 | 0 | 0 | -1.07 |
| FRMD8 | 0 | 0 | 0 | 1.33 |
| FRMPD3 | 0 | 0 | 0 | 1.12 |
| FRRS1 | 0 | 0 | 0 | -2.14 |
| FRY | 0 | 0 | 0 | -1.17 |
| FSCN1 | 0 | 0 | 0 | 1.63 |
| FSIP1 | 0 | 0 | 0 | 1.61 |
| FSTL1 | 0 | 0 | 0 | 2.3 |
| FSTL3 | 0 | 0 | 0 | 1.94 |
| FSTL5 | 0 | 0 | 0 | -1.33 |
| FTSJ1 | 0 | 0 | 0 | 1.5 |
| FUBP1 | 0 | 0 | 0 | 1.32 |
| FUNDC1 | 0 | 0 | 0 | 1.08 |
| FURIN | 0 | 0 | 0 | -2.03 |
| FUT1 | 0 | 0 | 0 | 1.15 |
| FUT2 | 0 | 0 | 0 | 6.27 |
| FUT4 | 0 | 0 | 0 | 2.06 |
| FUT8 | 0 | 0 | 0 | 3.14 |
| FXN | 0 | 0 | 0 | -1.14 |
| FXYD2 | 0 | 0 | 0 | -1.56 |
| FXYD3 | 0 | 0 | 0 | 2.42 |
| FXYD5 | 0 | 0 | 0 | 1.34 |
| FZD1 | 0 | 0 | 0 | 1.67 |
| FZD10 | 0 | 0 | 0 | 3.05 |
| FZD2 | 0 | 0 | 0 | 2.67 |
| FZD6 | 0 | 0 | 0 | 2.99 |
| FZD7 | 0 | 0 | 0 | 2.56 |
| G0S2 | 0 | 0 | 0 | -1.86 |
| G2E3 | 0 | 0 | 0 | 1.27 |
| G6PC3 | 0 | 0 | 0 | 1.2 |
| G6PD | 0 | 0 | 0 | 2.85 |
| GABARAPL1 | 0 | 0 | 0 | -1.82 |
| GABBR1 | 0 | 0 | 0 | 1.02 |
| GABRD | 0 | 0 | 0 | 1.21 |
| GABRE | 0 | 0 | 0 | 1.54 |
| GABRG1 | 0 | 0 | 0 | -1.51 |
| GADD45A | 0 | 0 | 0 | -1.26 |
| GALC | 0 | 0 | 0 | 1.35 |
| GALK1 | 0 | 0 | 0 | -2.49 |
| GALNT12 | 0 | 0 | 0 | 5.36 |
| GALNT3 | 0 | 0 | 0 | 2.91 |
| GALNT5 | 0 | 0 | 0 | 3.31 |
| GALNT6 | 0 | 0 | 0 | 2.39 |
| GALNT7 | 0 | 0 | 0 | 3.47 |
| GALP | 0 | 0 | 0 | -2.24 |
| GALR2 | 0 | 0 | 0 | -1.08 |
| GANC | 0 | 0 | 0 | 1.13 |
| GAP43 | 0 | 0 | 0 | 2.7 |
| GATS | 0 | 0 | 0 | 1.16 |
| GBAS | 0 | 0 | 0 | 1.4 |
| GBP2 | 0 | 0 | 0 | 1.86 |
| GBP5 | 0 | 0 | 0 | 1.93 |
| GCK | 0 | 0 | 0 | -1.47 |
| GCNT1 | 0 | 0 | 0 | 1.65 |
| GCNT3 | 0 | 0 | 0 | 4.93 |
| GCOM1 | 0 | 0 | 0 | 1.08 |
| GCSH | 0 | 0 | 0 | -1.74 |
| GDF2 | 0 | 0 | 0 | -4.8 |
| GDPD1 | 0 | 0 | 0 | 1.77 |
| GDPD3 | 0 | 0 | 0 | 1.84 |
| GDPD4 | 0 | 0 | 0 | -4.97 |
| GEM | 0 | 0 | 0 | 1.91 |
| GFI1 | 0 | 0 | 0 | 1.69 |
| GFOD2 | 0 | 0 | 0 | -1.32 |
| GFPT1 | 0 | 0 | 0 | 1.15 |
| GFPT2 | 0 | 0 | 0 | 4 |
| GGTLC2 | 0 | 0 | 0 | -1.11 |
| GHITM | 0 | 0 | 0 | -1.08 |
| GINS3 | 0 | 0 | 0 | 2.2 |
| GIPC2 | 0 | 0 | 0 | -2.08 |
| GIT1 | 0 | 0 | 0 | 1.43 |
| GJA5 | 0 | 0 | 0 | 2.19 |
| GJB4 | 0 | 0 | 0 | 6.83 |
| GJB5 | 0 | 0 | 0 | 5.39 |
| GJC1 | 0 | 0 | 0 | 2.1 |
| GK | 0 | 0 | 0 | -1.51 |
| GK5 | 0 | 0 | 0 | 1.57 |
| GKAP1 | 0 | 0 | 0 | -1.21 |
| GLA | 0 | 0 | 0 | 1.36 |
| GLI1 | 0 | 0 | 0 | 2.36 |
| GLI2 | 0 | 0 | 0 | 2.16 |
| GLI3 | 0 | 0 | 0 | 2.37 |
| GLI4 | 0 | 0 | 0 | 1.03 |
| GLIPR1 | 0 | 0 | 0 | 1.77 |
| GLIPR2 | 0 | 0 | 0 | 1.91 |
| GLIS2 | 0 | 0 | 0 | 2.23 |
| GLMN | 0 | 0 | 0 | 1.16 |
| GLS | 0 | 0 | 0 | 1.36 |
| GLT8D2 | 0 | 0 | 0 | 2.12 |
| GLTPD1 | 0 | 0 | 0 | -1.1 |
| GLYATL3 | 0 | 0 | 0 | -2.16 |
| GM2A | 0 | 0 | 0 | 1.26 |
| GMNC | 0 | 0 | 0 | -3.55 |
| GNA12 | 0 | 0 | 0 | 1.16 |
| GNA14 | 0 | 0 | 0 | -1.05 |
| GNA15 | 0 | 0 | 0 | 1.58 |
| GNAT2 | 0 | 0 | 0 | -1.18 |
| GNG4 | 0 | 0 | 0 | 3.88 |
| GNL3L | 0 | 0 | 0 | 1.08 |
| GNPDA2 | 0 | 0 | 0 | 1.44 |
| GNPNAT1 | 0 | 0 | 0 | -1.24 |
| GNPTAB | 0 | 0 | 0 | 1.48 |
| GNRH1 | 0 | 0 | 0 | 1.09 |
| GOLGA3 | 0 | 0 | 0 | 1.17 |
| GOLGA6A | 0 | 0 | 0 | -3.59 |
| GOLGA6C | 0 | 0 | 0 | -4.19 |
| GOLPH3L | 0 | 0 | 0 | 1.29 |
| GOT2 | 0 | 0 | 0 | -2.29 |
| GP1BB | 0 | 0 | 0 | 1.42 |
| GPA33 | 0 | 0 | 0 | 3.53 |
| GPATCH4 | 0 | 0 | 0 | 1.01 |
| GPATCH8 | 0 | 0 | 0 | 1.03 |
| GPC2 | 0 | 0 | 0 | 1.16 |
| GPC4 | 0 | 0 | 0 | 1.91 |
| GPD1 | 0 | 0 | 0 | -3.14 |
| GPD2 | 0 | 0 | 0 | 2.25 |
| GPNMB | 0 | 0 | 0 | 2.2 |
| GPR110 | 0 | 0 | 0 | 4.45 |
| GPR114 | 0 | 0 | 0 | 1.21 |
| GPR115 | 0 | 0 | 0 | 2.59 |
| GPR123 | 0 | 0 | 0 | -1.42 |
| GPR124 | 0 | 0 | 0 | 1.27 |
| GPR125 | 0 | 0 | 0 | -2.74 |
| GPR126 | 0 | 0 | 0 | -2.61 |
| GPR128 | 0 | 0 | 0 | -3.03 |
| GPR141 | 0 | 0 | 0 | 2.08 |
| GPR146 | 0 | 0 | 0 | -2.4 |
| GPR160 | 0 | 0 | 0 | 2.55 |
| GPR161 | 0 | 0 | 0 | 1.92 |
| GPR162 | 0 | 0 | 0 | -1.32 |
| GPR182 | 0 | 0 | 0 | -5.26 |
| GPR19 | 0 | 0 | 0 | 3.92 |
| GPR27 | 0 | 0 | 0 | 2.25 |
| GPR35 | 0 | 0 | 0 | 2.37 |
| GPR62 | 0 | 0 | 0 | -1.33 |
| GPR63 | 0 | 0 | 0 | 1.41 |
| GPR75 | 0 | 0 | 0 | -1.08 |
| GPR84 | 0 | 0 | 0 | 1.04 |
| GPR88 | 0 | 0 | 0 | -4.32 |
| GPR98 | 0 | 0 | 0 | -5.76 |
| GPRASP2 | 0 | 0 | 0 | -1.31 |
| GPRC5A | 0 | 0 | 0 | 6.66 |
| GPRC5C | 0 | 0 | 0 | -3.99 |
| GPRIN1 | 0 | 0 | 0 | 3.69 |
| GPRIN3 | 0 | 0 | 0 | 1.73 |
| GPSM1 | 0 | 0 | 0 | 1.46 |
| GRAMD1B | 0 | 0 | 0 | 3.11 |
| GREB1 | 0 | 0 | 0 | -1.24 |
| GRHL1 | 0 | 0 | 0 | -1.65 |
| GRIA3 | 0 | 0 | 0 | -4.08 |
| GRIK3 | 0 | 0 | 0 | -1.1 |
| GRIN2D | 0 | 0 | 0 | 2.82 |
| GRIP1 | 0 | 0 | 0 | 2.37 |
| GRM1 | 0 | 0 | 0 | 1.6 |
| GRM8 | 0 | 0 | 0 | -2.19 |
| GRN | 0 | 0 | 0 | 1.12 |
| GRPEL2 | 0 | 0 | 0 | 1.08 |
| GRTP1 | 0 | 0 | 0 | -2.94 |
| GSC | 0 | 0 | 0 | 3.62 |
| GSG2 | 0 | 0 | 0 | 3.44 |
| GSN | 0 | 0 | 0 | 1.46 |
| GSTCD | 0 | 0 | 0 | 1.51 |
| GSTK1 | 0 | 0 | 0 | -1.21 |
| GSTO1 | 0 | 0 | 0 | -1.4 |
| GSTP1 | 0 | 0 | 0 | 3.41 |
| GSTT2 | 0 | 0 | 0 | -1.68 |
| GSTT2B | 0 | 0 | 0 | -1.65 |
| GTF2F2 | 0 | 0 | 0 | 1.04 |
| GTF3C2 | 0 | 0 | 0 | 1.13 |
| GTPBP1 | 0 | 0 | 0 | 1.03 |
| GTPBP4 | 0 | 0 | 0 | 1.1 |
| GXYLT2 | 0 | 0 | 0 | 4.12 |
| GYG1 | 0 | 0 | 0 | 1.33 |
| GYG2 | 0 | 0 | 0 | -2.73 |
| GYS1 | 0 | 0 | 0 | 1.15 |
| H2AFJ | 0 | 0 | 0 | 1.51 |
| H2AFV | 0 | 0 | 0 | 1.5 |
| H2AFX | 0 | 0 | 0 | 2.25 |
| H2AFY2 | 0 | 0 | 0 | 1.12 |
| H2AFZ | 0 | 0 | 0 | 1.71 |
| HACL1 | 0 | 0 | 0 | -1.63 |
| HADHB | 0 | 0 | 0 | -1.49 |
| HAPLN3 | 0 | 0 | 0 | 3.7 |
| HAUS1 | 0 | 0 | 0 | 1.04 |
| HAUS2 | 0 | 0 | 0 | 1.16 |
| HAUS5 | 0 | 0 | 0 | 1.32 |
| HBCBP | 0 | 0 | 0 | -2.02 |
| HBM | 0 | 0 | 0 | -1.16 |
| HCFC1R1 | 0 | 0 | 0 | 1.21 |
| HDAC1 | 0 | 0 | 0 | 1 |
| HDAC11 | 0 | 0 | 0 | 1.07 |
| HDAC2 | 0 | 0 | 0 | 1.02 |
| HDAC7 | 0 | 0 | 0 | 1.76 |
| HDDC2 | 0 | 0 | 0 | 1.26 |
| HDGFRP3 | 0 | 0 | 0 | 2.91 |
| HDHD3 | 0 | 0 | 0 | -1.21 |
| HEATR1 | 0 | 0 | 0 | 1.31 |
| HEATR6 | 0 | 0 | 0 | 1.05 |
| HEATR7A | 0 | 0 | 0 | 2.27 |
| HEATR7B1 | 0 | 0 | 0 | -6.14 |
| HEBP1 | 0 | 0 | 0 | -1.17 |
| HEG1 | 0 | 0 | 0 | 1.34 |
| HENMT1 | 0 | 0 | 0 | 2.31 |
| HEPH | 0 | 0 | 0 | 2.09 |
| HERC6 | 0 | 0 | 0 | 1.32 |
| HES2 | 0 | 0 | 0 | 3.88 |
| HES4 | 0 | 0 | 0 | 2 |
| HGF | 0 | 0 | 0 | -1.76 |
| HHEX | 0 | 0 | 0 | -2.39 |
| HHIP | 0 | 0 | 0 | -3.09 |
| HHLA2 | 0 | 0 | 0 | 2.95 |
| HIATL1 | 0 | 0 | 0 | 1.15 |
| HIGD1C | 0 | 0 | 0 | -2.21 |
| HILPDA | 0 | 0 | 0 | 1.88 |
| HINT2 | 0 | 0 | 0 | -1.35 |
| HIPK2 | 0 | 0 | 0 | -1.48 |
| HIST1H1E | 0 | 0 | 0 | 1.16 |
| HIST1H2AG | 0 | 0 | 0 | 3.5 |
| HIST1H2AK | 0 | 0 | 0 | 1.77 |
| HIST1H2BB | 0 | 0 | 0 | 2 |
| HIST1H2BK | 0 | 0 | 0 | 1.82 |
| HIST1H2BL | 0 | 0 | 0 | 1.63 |
| HIST1H2BN | 0 | 0 | 0 | 1.02 |
| HIST1H3C | 0 | 0 | 0 | 1.22 |
| HIST1H3D | 0 | 0 | 0 | 3.08 |
| HIST1H3E | 0 | 0 | 0 | 2.08 |
| HIST1H3F | 0 | 0 | 0 | 3.31 |
| HIST1H3G | 0 | 0 | 0 | 2.62 |
| HIST1H3H | 0 | 0 | 0 | 2.77 |
| HIST1H3I | 0 | 0 | 0 | 3.39 |
| HIST1H3J | 0 | 0 | 0 | 2.59 |
| HIST1H4D | 0 | 0 | 0 | 2.11 |
| HIST1H4E | 0 | 0 | 0 | 1.54 |
| HIST1H4F | 0 | 0 | 0 | 1.69 |
| HIST1H4G | 0 | 0 | 0 | 1.29 |
| HIST1H4K | 0 | 0 | 0 | 1.11 |
| HIST1H4L | 0 | 0 | 0 | 2.3 |
| HIST2H2AB | 0 | 0 | 0 | 1.23 |
| HIST2H3A | 0 | 0 | 0 | 4.22 |
| HIST2H4B | 0 | 0 | 0 | 1.8 |
| HIST3H2A | 0 | 0 | 0 | 3.89 |
| HIST3H2BB | 0 | 0 | 0 | 1.7 |
| HIST4H4 | 0 | 0 | 0 | 1.27 |
| HIVEP2 | 0 | 0 | 0 | 1.02 |
| HIVEP3 | 0 | 0 | 0 | 1.06 |
| HK1 | 0 | 0 | 0 | 2.22 |
| HKR1 | 0 | 0 | 0 | 1.06 |
| HLA-A | 0 | 0 | 0 | 1.04 |
| HLA-B | 0 | 0 | 0 | 1.18 |
| HLA-DMA | 0 | 0 | 0 | 1.7 |
| HLA-DMB | 0 | 0 | 0 | 1.59 |
| HLA-DOA | 0 | 0 | 0 | 1.72 |
| HLA-DPA1 | 0 | 0 | 0 | 1.2 |
| HLA-DQA1 | 0 | 0 | 0 | 1.56 |
| HLA-DQB1 | 0 | 0 | 0 | 1.72 |
| HLA-DQB2 | 0 | 0 | 0 | 1.58 |
| HLA-DRA | 0 | 0 | 0 | 1.24 |
| HLA-DRB1 | 0 | 0 | 0 | 1.28 |
| HLA-F | 0 | 0 | 0 | 1.3 |
| HMGA1 | 0 | 0 | 0 | 3.79 |
| HMGA1P4 | 0 | 0 | 0 | 1.55 |
| HMGB2 | 0 | 0 | 0 | 2.33 |
| HMGCLL1 | 0 | 0 | 0 | -2.31 |
| HMGCS1 | 0 | 0 | 0 | -1.81 |
| HMGCS2 | 0 | 0 | 0 | -7.22 |
| HMGN1 | 0 | 0 | 0 | 1.26 |
| HMGN5 | 0 | 0 | 0 | -1.54 |
| HNF4A | 0 | 0 | 0 | -3.12 |
| HNMT | 0 | 0 | 0 | -1.64 |
| HNRNPA1 | 0 | 0 | 0 | 1.11 |
| HNRNPA1L2 | 0 | 0 | 0 | 1.09 |
| HNRNPA3 | 0 | 0 | 0 | 1.13 |
| HNRPLL | 0 | 0 | 0 | 1.02 |
| HOOK1 | 0 | 0 | 0 | -2.14 |
| HORMAD2 | 0 | 0 | 0 | -2.46 |
| HOXA2 | 0 | 0 | 0 | 1.91 |
| HOXA3 | 0 | 0 | 0 | 3.05 |
| HOXA6 | 0 | 0 | 0 | 3.29 |
| HOXA7 | 0 | 0 | 0 | 3.4 |
| HOXB13 | 0 | 0 | 0 | 5.28 |
| HOXB3 | 0 | 0 | 0 | 2.59 |
| HOXB5 | 0 | 0 | 0 | 2.7 |
| HOXB8 | 0 | 0 | 0 | 4.13 |
| HOXC4 | 0 | 0 | 0 | 3.12 |
| HOXC6 | 0 | 0 | 0 | 3.57 |
| HOXC8 | 0 | 0 | 0 | 3.42 |
| HOXC9 | 0 | 0 | 0 | 5.67 |
| HOXD1 | 0 | 0 | 0 | 2.81 |
| HOXD10 | 0 | 0 | 0 | 2.5 |
| HOXD3 | 0 | 0 | 0 | 1.86 |
| HOXD8 | 0 | 0 | 0 | 2.54 |
| HPCA | 0 | 0 | 0 | 2.29 |
| HPD | 0 | 0 | 0 | -8.27 |
| HPDL | 0 | 0 | 0 | 3.16 |
| HPSE | 0 | 0 | 0 | 1.14 |
| HR | 0 | 0 | 0 | 2.54 |
| HRASLS2 | 0 | 0 | 0 | 2.05 |
| HRCT1 | 0 | 0 | 0 | 1.64 |
| HRK | 0 | 0 | 0 | 3.73 |
| HS2ST1 | 0 | 0 | 0 | -1.1 |
| HS3ST1 | 0 | 0 | 0 | 3.08 |
| HS3ST2 | 0 | 0 | 0 | 2.37 |
| HS3ST3B1 | 0 | 0 | 0 | -3.07 |
| HSBP1L1 | 0 | 0 | 0 | -1.18 |
| HSD17B11 | 0 | 0 | 0 | -1.41 |
| HSD17B14 | 0 | 0 | 0 | -1.7 |
| HSD17B3 | 0 | 0 | 0 | -2.89 |
| HSD17B7 | 0 | 0 | 0 | -1.14 |
| HSD3B1 | 0 | 0 | 0 | -2.28 |
| HSD3B7 | 0 | 0 | 0 | -1.23 |
| HSF1 | 0 | 0 | 0 | 1.01 |
| HSF2BP | 0 | 0 | 0 | 1.64 |
| HSPA1B | 0 | 0 | 0 | 1.92 |
| HSPA2 | 0 | 0 | 0 | 2.5 |
| HSPA6 | 0 | 0 | 0 | 1.34 |
| HSPB9 | 0 | 0 | 0 | -1.86 |
| HSPBAP1 | 0 | 0 | 0 | 1.27 |
| HSPG2 | 0 | 0 | 0 | 1.11 |
| HSPH1 | 0 | 0 | 0 | 1.42 |
| HTATIP2 | 0 | 0 | 0 | 1.11 |
| HTR1D | 0 | 0 | 0 | 2.43 |
| HTR1F | 0 | 0 | 0 | 2.26 |
| HTR6 | 0 | 0 | 0 | -1.33 |
| HTRA4 | 0 | 0 | 0 | 3.26 |
| HUS1B | 0 | 0 | 0 | 1.13 |
| HYI | 0 | 0 | 0 | -1.08 |
| IAPP | 0 | 0 | 0 | -1.33 |
| IARS | 0 | 0 | 0 | 1.22 |
| ICAM3 | 0 | 0 | 0 | -1.31 |
| ICOS | 0 | 0 | 0 | 2.11 |
| ID2 | 0 | 0 | 0 | -2.17 |
| IDH1 | 0 | 0 | 0 | -1.63 |
| IDH2 | 0 | 0 | 0 | -1.27 |
| IDO1 | 0 | 0 | 0 | 3.67 |
| IER3 | 0 | 0 | 0 | 1.17 |
| IER5L | 0 | 0 | 0 | 1.74 |
| IFI16 | 0 | 0 | 0 | 2.1 |
| IFI27 | 0 | 0 | 0 | 1.75 |
| IFI27L1 | 0 | 0 | 0 | 1.6 |
| IFI27L2 | 0 | 0 | 0 | 2.58 |
| IFI30 | 0 | 0 | 0 | 1.5 |
| IFITM10 | 0 | 0 | 0 | -1.45 |
| IFNA21 | 0 | 0 | 0 | -3.15 |
| IFNE | 0 | 0 | 0 | 3.64 |
| IFNGR2 | 0 | 0 | 0 | 1.34 |
| IFT52 | 0 | 0 | 0 | 1.5 |
| IFT80 | 0 | 0 | 0 | 1.28 |
| IFT88 | 0 | 0 | 0 | -1.21 |
| IGANRP | 0 | 0 | 0 | 1.94 |
| IGBP1 | 0 | 0 | 0 | 1.3 |
| IGDCC3 | 0 | 0 | 0 | 1.9 |
| IGDCC4 | 0 | 0 | 0 | 3.5 |
| IGF1R | 0 | 0 | 0 | 2.27 |
| IGF2 | 0 | 0 | 0 | -3.23 |
| IGFBP3 | 0 | 0 | 0 | -1.92 |
| IGFBP4 | 0 | 0 | 0 | -1.14 |
| IGFBP5 | 0 | 0 | 0 | 1.96 |
| IGFBP7 | 0 | 0 | 0 | 1.12 |
| IGFL3 | 0 | 0 | 0 | 2.2 |
| IGLL1 | 0 | 0 | 0 | 2.72 |
| IGLL5 | 0 | 0 | 0 | 2.55 |
| IGSF1 | 0 | 0 | 0 | 1.64 |
| IGSF23 | 0 | 0 | 0 | -4.52 |
| IGSF5 | 0 | 0 | 0 | -1.44 |
| IGSF9 | 0 | 0 | 0 | -1.47 |
| IKBIP | 0 | 0 | 0 | 1.11 |
| IKBKB | 0 | 0 | 0 | 1.31 |
| IKZF2 | 0 | 0 | 0 | 1.32 |
| IL11RA | 0 | 0 | 0 | -1.21 |
| IL12A | 0 | 0 | 0 | 1.39 |
| IL12B | 0 | 0 | 0 | 1.91 |
| IL15 | 0 | 0 | 0 | 1.44 |
| IL17B | 0 | 0 | 0 | 1.83 |
| IL17C | 0 | 0 | 0 | 1.76 |
| IL17RC | 0 | 0 | 0 | -1.79 |
| IL17RD | 0 | 0 | 0 | 2.13 |
| IL18 | 0 | 0 | 0 | 2.31 |
| IL1RN | 0 | 0 | 0 | -1.44 |
| IL20RA | 0 | 0 | 0 | 3.1 |
| IL27RA | 0 | 0 | 0 | 1.27 |
| IL28RA | 0 | 0 | 0 | -1.51 |
| IL2RA | 0 | 0 | 0 | 3.1 |
| IL2RG | 0 | 0 | 0 | 1.85 |
| IL31RA | 0 | 0 | 0 | 1.54 |
| IL3RA | 0 | 0 | 0 | 1.7 |
| IL4I1 | 0 | 0 | 0 | 2.69 |
| IMPDH1 | 0 | 0 | 0 | 1.81 |
| IMPDH2 | 0 | 0 | 0 | 1.38 |
| IMPG2 | 0 | 0 | 0 | 1.28 |
| INCENP | 0 | 0 | 0 | 2.38 |
| INHBB | 0 | 0 | 0 | -1.98 |
| INHBC | 0 | 0 | 0 | -2.26 |
| INPP4A | 0 | 0 | 0 | 1.13 |
| INPP4B | 0 | 0 | 0 | 2.26 |
| INPP5D | 0 | 0 | 0 | 1.4 |
| INPP5F | 0 | 0 | 0 | 1.34 |
| INPP5J | 0 | 0 | 0 | 2.08 |
| INS-IGF2 | 0 | 0 | 0 | -4.96 |
| INSR | 0 | 0 | 0 | -2 |
| INTS2 | 0 | 0 | 0 | 1.31 |
| INTS8 | 0 | 0 | 0 | 1.28 |
| INTS9 | 0 | 0 | 0 | 1.36 |
| INTU | 0 | 0 | 0 | 1.66 |
| IP6K3 | 0 | 0 | 0 | -2.6 |
| IQCB1 | 0 | 0 | 0 | 1.47 |
| IQCC | 0 | 0 | 0 | 1.42 |
| IQCK | 0 | 0 | 0 | 1.16 |
| IQGAP1 | 0 | 0 | 0 | 2.01 |
| IRAK3 | 0 | 0 | 0 | 1.19 |
| IRF4 | 0 | 0 | 0 | 2.26 |
| IRS2 | 0 | 0 | 0 | -1.6 |
| ISG20 | 0 | 0 | 0 | 2.06 |
| ISL1 | 0 | 0 | 0 | 4.08 |
| ISLR | 0 | 0 | 0 | 1.87 |
| ISLR2 | 0 | 0 | 0 | 1.31 |
| ISPD | 0 | 0 | 0 | -1.58 |
| ISY1-RAB43 | 0 | 0 | 0 | -1.37 |
| ITCH | 0 | 0 | 0 | -1.62 |
| ITFG3 | 0 | 0 | 0 | 1.82 |
| ITGA4 | 0 | 0 | 0 | 1.1 |
| ITGA6 | 0 | 0 | 0 | 2.94 |
| ITGA9 | 0 | 0 | 0 | -1.09 |
| ITGAD | 0 | 0 | 0 | -3.17 |
| ITGAE | 0 | 0 | 0 | 1.48 |
| ITGAV | 0 | 0 | 0 | 1.76 |
| ITGB1BP1 | 0 | 0 | 0 | 1.44 |
| ITGB2 | 0 | 0 | 0 | 1.15 |
| ITGB3BP | 0 | 0 | 0 | 1.36 |
| ITGB4 | 0 | 0 | 0 | 5.68 |
| ITGB6 | 0 | 0 | 0 | 4.62 |
| ITGB8 | 0 | 0 | 0 | 3.98 |
| ITGBL1 | 0 | 0 | 0 | 2.67 |
| ITLN2 | 0 | 0 | 0 | -2.32 |
| ITM2C | 0 | 0 | 0 | 1.77 |
| ITPKB | 0 | 0 | 0 | 1.81 |
| ITPR2 | 0 | 0 | 0 | -2.34 |
| IVNS1ABP | 0 | 0 | 0 | 1.08 |
| IYD | 0 | 0 | 0 | -4.03 |
| IZUMO4 | 0 | 0 | 0 | -1.51 |
| JAG1 | 0 | 0 | 0 | 2.25 |
| JAG2 | 0 | 0 | 0 | 2.52 |
| JAK3 | 0 | 0 | 0 | 1.62 |
| JMJD6 | 0 | 0 | 0 | 1.13 |
| JOSD2 | 0 | 0 | 0 | 1.09 |
| JPH1 | 0 | 0 | 0 | 3.35 |
| JRKL | 0 | 0 | 0 | 1.53 |
| JSRP1 | 0 | 0 | 0 | 3.53 |
| KAL1 | 0 | 0 | 0 | 2.72 |
| KALRN | 0 | 0 | 0 | -2.7 |
| KANK1 | 0 | 0 | 0 | -2.43 |
| KATNB1 | 0 | 0 | 0 | 1.16 |
| KBTBD11 | 0 | 0 | 0 | -1.84 |
| KBTBD13 | 0 | 0 | 0 | -3.01 |
| KCNAB2 | 0 | 0 | 0 | 1.14 |
| KCNC2 | 0 | 0 | 0 | -3.06 |
| KCND1 | 0 | 0 | 0 | 1.08 |
| KCNE3 | 0 | 0 | 0 | 2.08 |
| KCNE4 | 0 | 0 | 0 | 2.54 |
| KCNG1 | 0 | 0 | 0 | 1.78 |
| KCNH2 | 0 | 0 | 0 | 1.81 |
| KCNH8 | 0 | 0 | 0 | 2.59 |
| KCNJ14 | 0 | 0 | 0 | 1.3 |
| KCNK10 | 0 | 0 | 0 | 2.77 |
| KCNK6 | 0 | 0 | 0 | 1.18 |
| KCNN3 | 0 | 0 | 0 | 3.07 |
| KCNN4 | 0 | 0 | 0 | 4.92 |
| KCNQ1 | 0 | 0 | 0 | 1.28 |
| KCNS1 | 0 | 0 | 0 | 1.44 |
| KCNT2 | 0 | 0 | 0 | -2.05 |
| KCP | 0 | 0 | 0 | 1.85 |
| KCTD10 | 0 | 0 | 0 | 1.14 |
| KCTD11 | 0 | 0 | 0 | 1.17 |
| KCTD13 | 0 | 0 | 0 | 1.56 |
| KCTD16 | 0 | 0 | 0 | 3.63 |
| KCTD17 | 0 | 0 | 0 | 1.54 |
| KCTD21 | 0 | 0 | 0 | -1.12 |
| KDELC2 | 0 | 0 | 0 | 1.3 |
| KDM4D | 0 | 0 | 0 | 1.95 |
| KDM4DL | 0 | 0 | 0 | -1.23 |
| KDR | 0 | 0 | 0 | -1.59 |
| KHDC1 | 0 | 0 | 0 | 2.92 |
| KIAA0146 | 0 | 0 | 0 | 1.07 |
| KIAA0226 | 0 | 0 | 0 | 1.1 |
| KIAA0247 | 0 | 0 | 0 | 1.12 |
| KIAA0319 | 0 | 0 | 0 | 2.29 |
| KIAA0408 | 0 | 0 | 0 | 1.9 |
| KIAA0513 | 0 | 0 | 0 | 1 |
| KIAA0564 | 0 | 0 | 0 | -1.18 |
| KIAA0664 | 0 | 0 | 0 | -1.69 |
| KIAA0889 | 0 | 0 | 0 | 1.33 |
| KIAA0907 | 0 | 0 | 0 | 1.13 |
| KIAA1199 | 0 | 0 | 0 | 2.16 |
| KIAA1211 | 0 | 0 | 0 | 2.29 |
| KIAA1244 | 0 | 0 | 0 | 2.5 |
| KIAA1324 | 0 | 0 | 0 | 3.06 |
| KIAA1377 | 0 | 0 | 0 | 2.31 |
| KIAA1407 | 0 | 0 | 0 | 1.32 |
| KIAA1456 | 0 | 0 | 0 | -2.18 |
| KIAA1462 | 0 | 0 | 0 | 2.09 |
| KIAA1522 | 0 | 0 | 0 | 1.78 |
| KIAA1644 | 0 | 0 | 0 | 2.51 |
| KIAA1797 | 0 | 0 | 0 | 1.15 |
| KIAA1826 | 0 | 0 | 0 | 1.09 |
| KIAA1841 | 0 | 0 | 0 | 1.77 |
| KIAA2018 | 0 | 0 | 0 | -1.19 |
| KIF12 | 0 | 0 | 0 | -1.99 |
| KIF16B | 0 | 0 | 0 | 1.18 |
| KIF18A | 0 | 0 | 0 | 4.79 |
| KIF18B | 0 | 0 | 0 | 2.53 |
| KIF20B | 0 | 0 | 0 | 1.39 |
| KIF21B | 0 | 0 | 0 | 1.22 |
| KIF22 | 0 | 0 | 0 | 1.82 |
| KIF3A | 0 | 0 | 0 | 1.72 |
| KIF5A | 0 | 0 | 0 | 1.04 |
| KIF5B | 0 | 0 | 0 | 1.35 |
| KIF6 | 0 | 0 | 0 | -1.14 |
| KIF7 | 0 | 0 | 0 | 1.95 |
| KIF9 | 0 | 0 | 0 | 1.05 |
| KIRREL | 0 | 0 | 0 | 1.65 |
| KIRREL-IT1 | 0 | 0 | 0 | 1.86 |
| KIRREL2 | 0 | 0 | 0 | 2.03 |
| KISS1R | 0 | 0 | 0 | 1.67 |
| KIT | 0 | 0 | 0 | 1.92 |
| KITLG | 0 | 0 | 0 | 2.75 |
| KLF12 | 0 | 0 | 0 | -1.42 |
| KLF5 | 0 | 0 | 0 | 4.63 |
| KLF7 | 0 | 0 | 0 | 1.49 |
| KLHDC10 | 0 | 0 | 0 | -1.22 |
| KLHDC7B | 0 | 0 | 0 | 2.44 |
| KLHDC9 | 0 | 0 | 0 | -1.87 |
| KLHL14 | 0 | 0 | 0 | -1.92 |
| KLHL15 | 0 | 0 | 0 | -1.2 |
| KLHL18 | 0 | 0 | 0 | 1 |
| KLHL35 | 0 | 0 | 0 | 1.45 |
| KLHL6 | 0 | 0 | 0 | 1.67 |
| KLHL7 | 0 | 0 | 0 | 1.09 |
| KLK10 | 0 | 0 | 0 | 1.33 |
| KLK11 | 0 | 0 | 0 | 4.44 |
| KLK6 | 0 | 0 | 0 | 4.42 |
| KLK7 | 0 | 0 | 0 | 4.91 |
| KLK8 | 0 | 0 | 0 | 4.78 |
| KLRB1 | 0 | 0 | 0 | -1.08 |
| KLRC1 | 0 | 0 | 0 | 1.57 |
| KLRF1 | 0 | 0 | 0 | -2.44 |
| KPNA2 | 0 | 0 | 0 | 2.29 |
| KPNB1 | 0 | 0 | 0 | 1.06 |
| KRT10 | 0 | 0 | 0 | 1.12 |
| KRT13 | 0 | 0 | 0 | 1.51 |
| KRT16 | 0 | 0 | 0 | 4.04 |
| KRT6A | 0 | 0 | 0 | 5.75 |
| KRT6C | 0 | 0 | 0 | 5.33 |
| KRTAP10-4 | 0 | 0 | 0 | -1.24 |
| KRTAP10-5 | 0 | 0 | 0 | -1.98 |
| KRTAP10-9 | 0 | 0 | 0 | -1.1 |
| KRTAP19-8 | 0 | 0 | 0 | -1.03 |
| KRTAP2-4 | 0 | 0 | 0 | -2.05 |
| KRTAP4-1 | 0 | 0 | 0 | -1.32 |
| KRTAP5-6 | 0 | 0 | 0 | -1.33 |
| KSR1 | 0 | 0 | 0 | 1.62 |
| L1CAM | 0 | 0 | 0 | 3.74 |
| L2HGDH | 0 | 0 | 0 | -1.09 |
| L3MBTL1 | 0 | 0 | 0 | 1.02 |
| L3MBTL4 | 0 | 0 | 0 | -1.73 |
| LAIR1 | 0 | 0 | 0 | 1.81 |
| LAIR2 | 0 | 0 | 0 | 2.1 |
| LAMB1 | 0 | 0 | 0 | 2.13 |
| LAMP2 | 0 | 0 | 0 | -1.54 |
| LAMP3 | 0 | 0 | 0 | 3.02 |
| LAPTM4B | 0 | 0 | 0 | 2.08 |
| LAPTM5 | 0 | 0 | 0 | 1.3 |
| LAT | 0 | 0 | 0 | 1.33 |
| LAT2 | 0 | 0 | 0 | 1.12 |
| LAX1 | 0 | 0 | 0 | 2.08 |
| LAYN | 0 | 0 | 0 | 2.53 |
| LBH | 0 | 0 | 0 | 1.72 |
| LBX2 | 0 | 0 | 0 | -1.49 |
| LCA5 | 0 | 0 | 0 | 1.13 |
| LCE2D | 0 | 0 | 0 | -1.75 |
| LCK | 0 | 0 | 0 | 1.52 |
| LCMT1 | 0 | 0 | 0 | 1.48 |
| LCN12 | 0 | 0 | 0 | -2.44 |
| LCTL | 0 | 0 | 0 | 1.83 |
| LDHB | 0 | 0 | 0 | 2.34 |
| LDLRAD2 | 0 | 0 | 0 | 2.31 |
| LDLRAD3 | 0 | 0 | 0 | 1.79 |
| LEF1 | 0 | 0 | 0 | 4.13 |
| LENG9 | 0 | 0 | 0 | 1.14 |
| LEP | 0 | 0 | 0 | 2.01 |
| LETM2 | 0 | 0 | 0 | 1.69 |
| LFNG | 0 | 0 | 0 | 2.53 |
| LGALS1 | 0 | 0 | 0 | 1.78 |
| LGALS3 | 0 | 0 | 0 | 2.51 |
| LGALS7 | 0 | 0 | 0 | 3.34 |
| LGALS9C | 0 | 0 | 0 | 1.86 |
| LGI2 | 0 | 0 | 0 | 2.31 |
| LGR4 | 0 | 0 | 0 | -1.27 |
| LHFPL2 | 0 | 0 | 0 | 2.51 |
| LIF | 0 | 0 | 0 | 2.67 |
| LIMA1 | 0 | 0 | 0 | 2.24 |
| LIMK2 | 0 | 0 | 0 | 1.47 |
| LIMS3L | 0 | 0 | 0 | 2.03 |
| LINGO4 | 0 | 0 | 0 | -1.28 |
| LIPG | 0 | 0 | 0 | -2.64 |
| LIPH | 0 | 0 | 0 | 5.07 |
| LIPJ | 0 | 0 | 0 | -1.02 |
| LIPM | 0 | 0 | 0 | 1.23 |
| LMAN1 | 0 | 0 | 0 | -1.23 |
| LMCD1 | 0 | 0 | 0 | 2.17 |
| LMNA | 0 | 0 | 0 | 1.27 |
| LMO4 | 0 | 0 | 0 | 1.95 |
| LMO7 | 0 | 0 | 0 | 3.39 |
| LMTK3 | 0 | 0 | 0 | 3.74 |
| LNPEP | 0 | 0 | 0 | 1.06 |
| LOC100126447 | 0 | 0 | 0 | -1.85 |
| LOC100127983 | 0 | 0 | 0 | 2.02 |
| LOC100128108 | 0 | 0 | 0 | 1.05 |
| LOC100128242 | 0 | 0 | 0 | 2.78 |
| LOC100128278 | 0 | 0 | 0 | 1.02 |
| LOC100128281 | 0 | 0 | 0 | 2.3 |
| LOC100128386 | 0 | 0 | 0 | 2.27 |
| LOC100128498 | 0 | 0 | 0 | -1.05 |
| LOC100128670 | 0 | 0 | 0 | 1.41 |
| LOC100128697 | 0 | 0 | 0 | 1.03 |
| LOC100128979 | 0 | 0 | 0 | 1.95 |
| LOC100128997 | 0 | 0 | 0 | 1.33 |
| LOC100129110 | 0 | 0 | 0 | 1.09 |
| LOC100129119 | 0 | 0 | 0 | -2.46 |
| LOC100129171 | 0 | 0 | 0 | 2.93 |
| LOC100129233 | 0 | 0 | 0 | 1.04 |
| LOC100129268 | 0 | 0 | 0 | 2.74 |
| LOC100129292 | 0 | 0 | 0 | -1.05 |
| LOC100129380 | 0 | 0 | 0 | 1.83 |
| LOC100129393 | 0 | 0 | 0 | 1.16 |
| LOC100129397 | 0 | 0 | 0 | 1.49 |
| LOC100129449 | 0 | 0 | 0 | 1.73 |
| LOC100129480 | 0 | 0 | 0 | 3.39 |
| LOC100129502 | 0 | 0 | 0 | 1.29 |
| LOC100129717 | 0 | 0 | 0 | 1.33 |
| LOC100129940 | 0 | 0 | 0 | 3.64 |
| LOC100130009 | 0 | 0 | 0 | 3.36 |
| LOC100130097 | 0 | 0 | 0 | -2.34 |
| LOC100130269 | 0 | 0 | 0 | -2.45 |
| LOC100130278 | 0 | 0 | 0 | -2.59 |
| LOC100130357 | 0 | 0 | 0 | 1.08 |
| LOC100130373 | 0 | 0 | 0 | 1.29 |
| LOC100130433 | 0 | 0 | 0 | -1.16 |
| LOC100130503 | 0 | 0 | 0 | -2.97 |
| LOC100130547 | 0 | 0 | 0 | 1.38 |
| LOC100130580 | 0 | 0 | 0 | 2.16 |
| LOC100130587 | 0 | 0 | 0 | -4.42 |
| LOC100130811 | 0 | 0 | 0 | 3.3 |
| LOC100130890 | 0 | 0 | 0 | 1.06 |
| LOC100130916 | 0 | 0 | 0 | -1.55 |
| LOC100130927 | 0 | 0 | 0 | -1.37 |
| LOC100130930 | 0 | 0 | 0 | 1.1 |
| LOC100131043 | 0 | 0 | 0 | 2.35 |
| LOC100131094 | 0 | 0 | 0 | -1.29 |
| LOC100131176 | 0 | 0 | 0 | 1.85 |
| LOC100131234 | 0 | 0 | 0 | 1.16 |
| LOC100131354 | 0 | 0 | 0 | 2.35 |
| LOC100131541 | 0 | 0 | 0 | 1.88 |
| LOC100131756 | 0 | 0 | 0 | -4.08 |
| LOC100131774 | 0 | 0 | 0 | 2.28 |
| LOC100131792 | 0 | 0 | 0 | 1.35 |
| LOC100131796 | 0 | 0 | 0 | -3.45 |
| LOC100131820 | 0 | 0 | 0 | 1.86 |
| LOC100131910 | 0 | 0 | 0 | 1.76 |
| LOC100132005 | 0 | 0 | 0 | -1.45 |
| LOC100132319 | 0 | 0 | 0 | -1.22 |
| LOC100132330 | 0 | 0 | 0 | 3.26 |
| LOC100132439 | 0 | 0 | 0 | 1.34 |
| LOC100132686 | 0 | 0 | 0 | -1.11 |
| LOC100132815 | 0 | 0 | 0 | -1.28 |
| LOC100132859 | 0 | 0 | 0 | 1.42 |
| LOC100132909 | 0 | 0 | 0 | 1.94 |
| LOC100133039 | 0 | 0 | 0 | 1.03 |
| LOC100133190 | 0 | 0 | 0 | 2.64 |
| LOC100271836 | 0 | 0 | 0 | -1.49 |
| LOC100286925 | 0 | 0 | 0 | 1.15 |
| LOC100287063 | 0 | 0 | 0 | 1.02 |
| LOC100287195 | 0 | 0 | 0 | 1.1 |
| LOC100287437 | 0 | 0 | 0 | 2.71 |
| LOC100287482 | 0 | 0 | 0 | 3.24 |
| LOC100287628 | 0 | 0 | 0 | -2.16 |
| LOC100288092 | 0 | 0 | 0 | 1.89 |
| LOC100288368 | 0 | 0 | 0 | -1 |
| LOC100288601 | 0 | 0 | 0 | -1.12 |
| LOC100288602 | 0 | 0 | 0 | 1.09 |
| LOC100288884 | 0 | 0 | 0 | 1.02 |
| LOC100289092 | 0 | 0 | 0 | 3 |
| LOC100289186 | 0 | 0 | 0 | -1.1 |
| LOC100289187 | 0 | 0 | 0 | 1.02 |
| LOC100289388 | 0 | 0 | 0 | 1.27 |
| LOC100292409 | 0 | 0 | 0 | 1.19 |
| LOC100292922 | 0 | 0 | 0 | 2.26 |
| LOC100293612 | 0 | 0 | 0 | -1.26 |
| LOC100293962 | 0 | 0 | 0 | 1.34 |
| LOC100499183 | 0 | 0 | 0 | 1.63 |
| LOC100505473 | 0 | 0 | 0 | 2.25 |
| LOC100505498 | 0 | 0 | 0 | 1.83 |
| LOC100505500 | 0 | 0 | 0 | 1.63 |
| LOC100505501 | 0 | 0 | 0 | 1.8 |
| LOC100505508 | 0 | 0 | 0 | -3.69 |
| LOC100505531 | 0 | 0 | 0 | -1.37 |
| LOC100505535 | 0 | 0 | 0 | 5.17 |
| LOC100505550 | 0 | 0 | 0 | -2.52 |
| LOC100505551 | 0 | 0 | 0 | -1.4 |
| LOC100505566 | 0 | 0 | 0 | 3.8 |
| LOC100505570 | 0 | 0 | 0 | -5.47 |
| LOC100505574 | 0 | 0 | 0 | -2.17 |
| LOC100505585 | 0 | 0 | 0 | 3.76 |
| LOC100505592 | 0 | 0 | 0 | 2.62 |
| LOC100505616 | 0 | 0 | 0 | 1.17 |
| LOC100505634 | 0 | 0 | 0 | 1.55 |
| LOC100505637 | 0 | 0 | 0 | 1.89 |
| LOC100505657 | 0 | 0 | 0 | -1.49 |
| LOC100505661 | 0 | 0 | 0 | 1.59 |
| LOC100505663 | 0 | 0 | 0 | -5.35 |
| LOC100505668 | 0 | 0 | 0 | 3.44 |
| LOC100505725 | 0 | 0 | 0 | 1.15 |
| LOC100505727 | 0 | 0 | 0 | -2.28 |
| LOC100505737 | 0 | 0 | 0 | -1.81 |
| LOC100505754 | 0 | 0 | 0 | -1.01 |
| LOC100505787 | 0 | 0 | 0 | 2.22 |
| LOC100505794 | 0 | 0 | 0 | 1.25 |
| LOC100505813 | 0 | 0 | 0 | 1.92 |
| LOC100505821 | 0 | 0 | 0 | -2.53 |
| LOC100505841 | 0 | 0 | 0 | 1.23 |
| LOC100505863 | 0 | 0 | 0 | -3.08 |
| LOC100505880 | 0 | 0 | 0 | -1.99 |
| LOC100505906 | 0 | 0 | 0 | 1.74 |
| LOC100505908 | 0 | 0 | 0 | 2.48 |
| LOC100505938 | 0 | 0 | 0 | 2.26 |
| LOC100505946 | 0 | 0 | 0 | 4.16 |
| LOC100505987 | 0 | 0 | 0 | -1.83 |
| LOC100505988 | 0 | 0 | 0 | 2.14 |
| LOC100505994 | 0 | 0 | 0 | 2.68 |
| LOC100506006 | 0 | 0 | 0 | -1.8 |
| LOC100506021 | 0 | 0 | 0 | -1.34 |
| LOC100506024 | 0 | 0 | 0 | 2.04 |
| LOC100506027 | 0 | 0 | 0 | 5.83 |
| LOC100506030 | 0 | 0 | 0 | 1.01 |
| LOC100506075 | 0 | 0 | 0 | 1.9 |
| LOC100506110 | 0 | 0 | 0 | 1.58 |
| LOC100506119 | 0 | 0 | 0 | 1.45 |
| LOC100506120 | 0 | 0 | 0 | 2.25 |
| LOC100506130 | 0 | 0 | 0 | 3.63 |
| LOC100506191 | 0 | 0 | 0 | 1.66 |
| LOC100506245 | 0 | 0 | 0 | 1.62 |
| LOC100506252 | 0 | 0 | 0 | -1.44 |
| LOC100506286 | 0 | 0 | 0 | 1.77 |
| LOC100506295 | 0 | 0 | 0 | -1.08 |
| LOC100506299 | 0 | 0 | 0 | -2.42 |
| LOC100506303 | 0 | 0 | 0 | 4.18 |
| LOC100506310 | 0 | 0 | 0 | 1.58 |
| LOC100506319 | 0 | 0 | 0 | -1.18 |
| LOC100506328 | 0 | 0 | 0 | -3.86 |
| LOC100506361 | 0 | 0 | 0 | 1.62 |
| LOC100506379 | 0 | 0 | 0 | 1.76 |
| LOC100506380 | 0 | 0 | 0 | -1.96 |
| LOC100506392 | 0 | 0 | 0 | 1.02 |
| LOC100506408 | 0 | 0 | 0 | -1.6 |
| LOC100506411 | 0 | 0 | 0 | 2.41 |
| LOC100506412 | 0 | 0 | 0 | -1.04 |
| LOC100506415 | 0 | 0 | 0 | -3.27 |
| LOC100506451 | 0 | 0 | 0 | -1.64 |
| LOC100506465 | 0 | 0 | 0 | 2.76 |
| LOC100506467 | 0 | 0 | 0 | -1.31 |
| LOC100506473 | 0 | 0 | 0 | 1.27 |
| LOC100506518 | 0 | 0 | 0 | -1.32 |
| LOC100506546 | 0 | 0 | 0 | 1.21 |
| LOC100506553 | 0 | 0 | 0 | -3.72 |
| LOC100506561 | 0 | 0 | 0 | 1.91 |
| LOC100506580 | 0 | 0 | 0 | -4.29 |
| LOC100506591 | 0 | 0 | 0 | 1.92 |
| LOC100506629 | 0 | 0 | 0 | 1.51 |
| LOC100506673 | 0 | 0 | 0 | -1.56 |
| LOC100506691 | 0 | 0 | 0 | 1.4 |
| LOC100506694 | 0 | 0 | 0 | 1.19 |
| LOC100506705 | 0 | 0 | 0 | 1.64 |
| LOC100506740 | 0 | 0 | 0 | 1.65 |
| LOC100506775 | 0 | 0 | 0 | 2.5 |
| LOC100506798 | 0 | 0 | 0 | 3.86 |
| LOC100506802 | 0 | 0 | 0 | 1.63 |
| LOC100506831 | 0 | 0 | 0 | 1.29 |
| LOC100506847 | 0 | 0 | 0 | 2.2 |
| LOC100506859 | 0 | 0 | 0 | 2.45 |
| LOC100506861 | 0 | 0 | 0 | -1.26 |
| LOC100506881 | 0 | 0 | 0 | 2.07 |
| LOC100506898 | 0 | 0 | 0 | -2.3 |
| LOC100506901 | 0 | 0 | 0 | 1.6 |
| LOC100506910 | 0 | 0 | 0 | 1.18 |
| LOC100506912 | 0 | 0 | 0 | -2.08 |
| LOC100506914 | 0 | 0 | 0 | 2.34 |
| LOC100506924 | 0 | 0 | 0 | -1.13 |
| LOC100506930 | 0 | 0 | 0 | 1.23 |
| LOC100506934 | 0 | 0 | 0 | 2.52 |
| LOC100506942 | 0 | 0 | 0 | -2.07 |
| LOC100507008 | 0 | 0 | 0 | -8.53 |
| LOC100507025 | 0 | 0 | 0 | 3.47 |
| LOC100507039 | 0 | 0 | 0 | 4.13 |
| LOC100507094 | 0 | 0 | 0 | 1.56 |
| LOC100507101 | 0 | 0 | 0 | 3.91 |
| LOC100507133 | 0 | 0 | 0 | 1.35 |
| LOC100507136 | 0 | 0 | 0 | -1.44 |
| LOC100507144 | 0 | 0 | 0 | 2.24 |
| LOC100507149 | 0 | 0 | 0 | -1.17 |
| LOC100507164 | 0 | 0 | 0 | 2.05 |
| LOC100507165 | 0 | 0 | 0 | 3.27 |
| LOC100507175 | 0 | 0 | 0 | 2.17 |
| LOC100507199 | 0 | 0 | 0 | -1.07 |
| LOC100507241 | 0 | 0 | 0 | -1.33 |
| LOC100507245 | 0 | 0 | 0 | 1.59 |
| LOC100507263 | 0 | 0 | 0 | 1.64 |
| LOC100507286 | 0 | 0 | 0 | 4.28 |
| LOC100507288 | 0 | 0 | 0 | -5.39 |
| LOC100507336 | 0 | 0 | 0 | -1.17 |
| LOC100507347 | 0 | 0 | 0 | 1.97 |
| LOC100507364 | 0 | 0 | 0 | -1.04 |
| LOC100507367 | 0 | 0 | 0 | 1.04 |
| LOC100507372 | 0 | 0 | 0 | 1.25 |
| LOC100507373 | 0 | 0 | 0 | 1.05 |
| LOC100507375 | 0 | 0 | 0 | 2.78 |
| LOC100507378 | 0 | 0 | 0 | 4.87 |
| LOC100507398 | 0 | 0 | 0 | 1.03 |
| LOC100507420 | 0 | 0 | 0 | 4.12 |
| LOC100507421 | 0 | 0 | 0 | 1.47 |
| LOC100507425 | 0 | 0 | 0 | 1.08 |
| LOC100507429 | 0 | 0 | 0 | 2.13 |
| LOC100507448 | 0 | 0 | 0 | -1.3 |
| LOC100507449 | 0 | 0 | 0 | 1.26 |
| LOC100507452 | 0 | 0 | 0 | 1.46 |
| LOC100507458 | 0 | 0 | 0 | 1.29 |
| LOC100507460 | 0 | 0 | 0 | 2.89 |
| LOC100507464 | 0 | 0 | 0 | -2.23 |
| LOC100507474 | 0 | 0 | 0 | 4.26 |
| LOC100507503 | 0 | 0 | 0 | 1.13 |
| LOC100507507 | 0 | 0 | 0 | 1.77 |
| LOC100507511 | 0 | 0 | 0 | -2.96 |
| LOC100507554 | 0 | 0 | 0 | 1.22 |
| LOC100507562 | 0 | 0 | 0 | -1.26 |
| LOC100507580 | 0 | 0 | 0 | 1.68 |
| LOC100507591 | 0 | 0 | 0 | -1.54 |
| LOC100507599 | 0 | 0 | 0 | 1.64 |
| LOC100507603 | 0 | 0 | 0 | -2.2 |
| LOC100507619 | 0 | 0 | 0 | 1.45 |
| LOC100507637 | 0 | 0 | 0 | 1.33 |
| LOC100507656 | 0 | 0 | 0 | -1.74 |
| LOC100507664 | 0 | 0 | 0 | -6.77 |
| LOC100507759 | 0 | 0 | 0 | 1.62 |
| LOC100507800 | 0 | 0 | 0 | 1.75 |
| LOC100507904 | 0 | 0 | 0 | 1.13 |
| LOC100507930 | 0 | 0 | 0 | 3.23 |
| LOC100507959 | 0 | 0 | 0 | 1.46 |
| LOC100508226 | 0 | 0 | 0 | -1.01 |
| LOC100508233 | 0 | 0 | 0 | 2.38 |
| LOC100508383 | 0 | 0 | 0 | -1.34 |
| LOC100508384 | 0 | 0 | 0 | -2.82 |
| LOC100508885 | 0 | 0 | 0 | -1.37 |
| LOC100509075 | 0 | 0 | 0 | -1.41 |
| LOC100509105 | 0 | 0 | 0 | -1.15 |
| LOC100509315 | 0 | 0 | 0 | 1.66 |
| LOC100509620 | 0 | 0 | 0 | -2.33 |
| LOC100509635 | 0 | 0 | 0 | 2.61 |
| LOC100509671 | 0 | 0 | 0 | 1.29 |
| LOC100509780 | 0 | 0 | 0 | 2.35 |
| LOC100652727 | 0 | 0 | 0 | 1.53 |
| LOC100652730 | 0 | 0 | 0 | -2.04 |
| LOC100652740 | 0 | 0 | 0 | 2.3 |
| LOC100652751 | 0 | 0 | 0 | -1.38 |
| LOC100652763 | 0 | 0 | 0 | -1.41 |
| LOC100652764 | 0 | 0 | 0 | 1.05 |
| LOC100652793 | 0 | 0 | 0 | 1.07 |
| LOC100652802 | 0 | 0 | 0 | -1.76 |
| LOC100652823 | 0 | 0 | 0 | -2.8 |
| LOC100652837 | 0 | 0 | 0 | 1.28 |
| LOC100652867 | 0 | 0 | 0 | 1.55 |
| LOC100652877 | 0 | 0 | 0 | 1.01 |
| LOC100652921 | 0 | 0 | 0 | -1.87 |
| LOC100652927 | 0 | 0 | 0 | 1.09 |
| LOC100652931 | 0 | 0 | 0 | -1.19 |
| LOC100652935 | 0 | 0 | 0 | 1.27 |
| LOC100652965 | 0 | 0 | 0 | 2.2 |
| LOC100652966 | 0 | 0 | 0 | -5.85 |
| LOC100652967 | 0 | 0 | 0 | -1.86 |
| LOC100652974 | 0 | 0 | 0 | -2.15 |
| LOC100652983 | 0 | 0 | 0 | -2.84 |
| LOC100652988 | 0 | 0 | 0 | 1.8 |
| LOC100652995 | 0 | 0 | 0 | -2.69 |
| LOC100653080 | 0 | 0 | 0 | -1.36 |
| LOC100653103 | 0 | 0 | 0 | -2.45 |
| LOC100653149 | 0 | 0 | 0 | 4.23 |
| LOC100653210 | 0 | 0 | 0 | 2.89 |
| LOC100653221 | 0 | 0 | 0 | 1.38 |
| LOC100653224 | 0 | 0 | 0 | -1.16 |
| LOC100653284 | 0 | 0 | 0 | -1.61 |
| LOC100653515 | 0 | 0 | 0 | 1.31 |
| LOC128322 | 0 | 0 | 0 | 1.07 |
| LOC145694 | 0 | 0 | 0 | 3.21 |
| LOC149351 | 0 | 0 | 0 | -1.22 |
| LOC158402 | 0 | 0 | 0 | 1.33 |
| LOC253805 | 0 | 0 | 0 | -1.1 |
| LOC280665 | 0 | 0 | 0 | -1.23 |
| LOC283028 | 0 | 0 | 0 | 3.32 |
| LOC283143 | 0 | 0 | 0 | 1.49 |
| LOC283454 | 0 | 0 | 0 | 3.22 |
| LOC283516 | 0 | 0 | 0 | -2.33 |
| LOC283588 | 0 | 0 | 0 | 1.06 |
| LOC283710 | 0 | 0 | 0 | 4.1 |
| LOC283731 | 0 | 0 | 0 | 2.33 |
| LOC283854 | 0 | 0 | 0 | -1.64 |
| LOC284219 | 0 | 0 | 0 | 1.81 |
| LOC284260 | 0 | 0 | 0 | -1.02 |
| LOC284408 | 0 | 0 | 0 | 2.24 |
| LOC284513 | 0 | 0 | 0 | 1.88 |
| LOC284581 | 0 | 0 | 0 | -2.27 |
| LOC284669 | 0 | 0 | 0 | -3.72 |
| LOC285095 | 0 | 0 | 0 | -1.71 |
| LOC285181 | 0 | 0 | 0 | -4.41 |
| LOC285389 | 0 | 0 | 0 | -2.5 |
| LOC285419 | 0 | 0 | 0 | 2.3 |
| LOC286087 | 0 | 0 | 0 | -2.56 |
| LOC286109 | 0 | 0 | 0 | 1 |
| LOC286382 | 0 | 0 | 0 | -1.16 |
| LOC339988 | 0 | 0 | 0 | 1.9 |
| LOC340340 | 0 | 0 | 0 | 3.79 |
| LOC388282 | 0 | 0 | 0 | -2.69 |
| LOC388630 | 0 | 0 | 0 | -1.17 |
| LOC388849 | 0 | 0 | 0 | 1.11 |
| LOC390940 | 0 | 0 | 0 | 1.43 |
| LOC399875 | 0 | 0 | 0 | -1.14 |
| LOC400684 | 0 | 0 | 0 | 2.14 |
| LOC400706 | 0 | 0 | 0 | 2.12 |
| LOC400768 | 0 | 0 | 0 | 2.97 |
| LOC401188 | 0 | 0 | 0 | -1.97 |
| LOC401847 | 0 | 0 | 0 | 2.75 |
| LOC440132 | 0 | 0 | 0 | 1.78 |
| LOC440330 | 0 | 0 | 0 | -1.81 |
| LOC440934 | 0 | 0 | 0 | 2.27 |
| LOC440993 | 0 | 0 | 0 | -1.24 |
| LOC441268 | 0 | 0 | 0 | 2.08 |
| LOC550643 | 0 | 0 | 0 | 1.46 |
| LOC642366 | 0 | 0 | 0 | 2.74 |
| LOC643037 | 0 | 0 | 0 | -3.83 |
| LOC644366 | 0 | 0 | 0 | -1.63 |
| LOC644841 | 0 | 0 | 0 | -1.06 |
| LOC644961 | 0 | 0 | 0 | 1.58 |
| LOC645195 | 0 | 0 | 0 | 2.11 |
| LOC645261 | 0 | 0 | 0 | -1.06 |
| LOC646851 | 0 | 0 | 0 | 1.44 |
| LOC646976 | 0 | 0 | 0 | -2.2 |
| LOC647086 | 0 | 0 | 0 | 1.05 |
| LOC650293 | 0 | 0 | 0 | 1.7 |
| LOC654841 | 0 | 0 | 0 | 1.37 |
| LOC727916 | 0 | 0 | 0 | -1.88 |
| LOC728052 | 0 | 0 | 0 | -2.58 |
| LOC728147 | 0 | 0 | 0 | 1.1 |
| LOC728208 | 0 | 0 | 0 | -5.91 |
| LOC728218 | 0 | 0 | 0 | -5.27 |
| LOC728290 | 0 | 0 | 0 | -6.28 |
| LOC728392 | 0 | 0 | 0 | 1.39 |
| LOC728485 | 0 | 0 | 0 | 1.39 |
| LOC728715 | 0 | 0 | 0 | 2.86 |
| LOC728763 | 0 | 0 | 0 | -2.39 |
| LOC729040 | 0 | 0 | 0 | 1.3 |
| LOC729658 | 0 | 0 | 0 | -1.32 |
| LOC729680 | 0 | 0 | 0 | 3.48 |
| LOC729732 | 0 | 0 | 0 | 2.14 |
| LOC729860 | 0 | 0 | 0 | 2.03 |
| LOC729870 | 0 | 0 | 0 | 2.68 |
| LOC730338 | 0 | 0 | 0 | -1.03 |
| LOC731282 | 0 | 0 | 0 | -1.87 |
| LOC81691 | 0 | 0 | 0 | 1.06 |
| LONP2 | 0 | 0 | 0 | -1.9 |
| LOR | 0 | 0 | 0 | 1.48 |
| LOX | 0 | 0 | 0 | 3.95 |
| LOXL1 | 0 | 0 | 0 | 3.58 |
| LOXL3 | 0 | 0 | 0 | 1.39 |
| LPAR5 | 0 | 0 | 0 | 1.78 |
| LPCAT1 | 0 | 0 | 0 | 1.44 |
| LPCAT2 | 0 | 0 | 0 | 1.09 |
| LPCAT4 | 0 | 0 | 0 | 2.95 |
| LPHN2 | 0 | 0 | 0 | -1.09 |
| LPIN2 | 0 | 0 | 0 | -1.76 |
| LPPR1 | 0 | 0 | 0 | -4.02 |
| LPPR4 | 0 | 0 | 0 | 2.79 |
| LRAT | 0 | 0 | 0 | -4.07 |
| LRIG2 | 0 | 0 | 0 | 1.2 |
| LRP10 | 0 | 0 | 0 | 1.27 |
| LRP11 | 0 | 0 | 0 | 1.5 |
| LRP12 | 0 | 0 | 0 | 2.22 |
| LRP3 | 0 | 0 | 0 | -2.11 |
| LRP5 | 0 | 0 | 0 | -1.45 |
| LRP5L | 0 | 0 | 0 | -1.16 |
| LRP6 | 0 | 0 | 0 | -1.1 |
| LRP8 | 0 | 0 | 0 | 4.32 |
| LRR1 | 0 | 0 | 0 | 1.21 |
| LRRC14 | 0 | 0 | 0 | 1.2 |
| LRRC15 | 0 | 0 | 0 | 3.58 |
| LRRC16A | 0 | 0 | 0 | 2.33 |
| LRRC2 | 0 | 0 | 0 | -3.13 |
| LRRC20 | 0 | 0 | 0 | -1.71 |
| LRRC23 | 0 | 0 | 0 | 1.78 |
| LRRC34 | 0 | 0 | 0 | 1.05 |
| LRRC36 | 0 | 0 | 0 | 1.89 |
| LRRC37A2 | 0 | 0 | 0 | 1.56 |
| LRRC37A3 | 0 | 0 | 0 | 1.26 |
| LRRC37B | 0 | 0 | 0 | 1.13 |
| LRRC38 | 0 | 0 | 0 | 1.42 |
| LRRC55 | 0 | 0 | 0 | -2.69 |
| LRRC57 | 0 | 0 | 0 | 1.18 |
| LRRC6 | 0 | 0 | 0 | 1.21 |
| LRRC69 | 0 | 0 | 0 | 1.34 |
| LRRC8B | 0 | 0 | 0 | 1.07 |
| LRRCC1 | 0 | 0 | 0 | 1.23 |
| LRRFIP1 | 0 | 0 | 0 | -1.63 |
| LRRIQ1 | 0 | 0 | 0 | 2.56 |
| LRRK1 | 0 | 0 | 0 | 1.63 |
| LRRN3 | 0 | 0 | 0 | -1.02 |
| LRRTM4 | 0 | 0 | 0 | -3.3 |
| LSAMP | 0 | 0 | 0 | 2.12 |
| LSM11 | 0 | 0 | 0 | 1.02 |
| LSM2 | 0 | 0 | 0 | 1.23 |
| LSMD1 | 0 | 0 | 0 | 2.18 |
| LSP1 | 0 | 0 | 0 | 1.86 |
| LSS | 0 | 0 | 0 | -1.48 |
| LTB | 0 | 0 | 0 | 1.44 |
| LTBP1 | 0 | 0 | 0 | 1.55 |
| LTBP2 | 0 | 0 | 0 | 3.37 |
| LUC7L3 | 0 | 0 | 0 | 1 |
| LUM | 0 | 0 | 0 | 2.91 |
| LUZP1 | 0 | 0 | 0 | 1.58 |
| LUZP2 | 0 | 0 | 0 | 2.1 |
| LXN | 0 | 0 | 0 | 3.13 |
| LY6D | 0 | 0 | 0 | 5.87 |
| LY6G6D | 0 | 0 | 0 | -1.05 |
| LY6G6F | 0 | 0 | 0 | -1.83 |
| LY6H | 0 | 0 | 0 | 2.84 |
| LY6K | 0 | 0 | 0 | 3.82 |
| LY75 | 0 | 0 | 0 | 2.95 |
| LYAR | 0 | 0 | 0 | 1.02 |
| LYN | 0 | 0 | 0 | 1.05 |
| LYNX1 | 0 | 0 | 0 | -1.4 |
| LYPD3 | 0 | 0 | 0 | 3.92 |
| LYPD5 | 0 | 0 | 0 | 3.61 |
| LYRM1 | 0 | 0 | 0 | -1.5 |
| LZTS1 | 0 | 0 | 0 | 2.12 |
| LZTS2 | 0 | 0 | 0 | 1.04 |
| MACC1 | 0 | 0 | 0 | 4.01 |
| MACROD1 | 0 | 0 | 0 | -1.15 |
| MACROD2 | 0 | 0 | 0 | -1.49 |
| MAD1L1 | 0 | 0 | 0 | -1.07 |
| MAF | 0 | 0 | 0 | -1.18 |
| MAGEA2B | 0 | 0 | 0 | 1.75 |
| MAGEL2 | 0 | 0 | 0 | 2.34 |
| MAGI1 | 0 | 0 | 0 | -1.33 |
| MAGI2-IT1 | 0 | 0 | 0 | -1.71 |
| MAGIX | 0 | 0 | 0 | -2.06 |
| MALL | 0 | 0 | 0 | 3.32 |
| MAMDC4 | 0 | 0 | 0 | -1.72 |
| MAML2 | 0 | 0 | 0 | 1.9 |
| MAN1C1 | 0 | 0 | 0 | -1.8 |
| MAN2A2 | 0 | 0 | 0 | -1.32 |
| MAP1A | 0 | 0 | 0 | 2.15 |
| MAP1B | 0 | 0 | 0 | 2.17 |
| MAP3K12 | 0 | 0 | 0 | 1.45 |
| MAP4 | 0 | 0 | 0 | 1.03 |
| MAP4K5 | 0 | 0 | 0 | 1.24 |
| MAP6 | 0 | 0 | 0 | 1 |
| MAP7D1 | 0 | 0 | 0 | 1.39 |
| MAP9 | 0 | 0 | 0 | 2.6 |
| MAPK12 | 0 | 0 | 0 | 1.86 |
| MAPK15 | 0 | 0 | 0 | 1.54 |
| MAPK3 | 0 | 0 | 0 | 1.26 |
| MAPK8IP2 | 0 | 0 | 0 | 1.4 |
| MAPRE1 | 0 | 0 | 0 | 1.63 |
| MARCKS | 0 | 0 | 0 | 1.8 |
| MARCKSL1 | 0 | 0 | 0 | 1.53 |
| MARVELD1 | 0 | 0 | 0 | 2.38 |
| MARVELD2 | 0 | 0 | 0 | -1.71 |
| MB21D1 | 0 | 0 | 0 | 2.13 |
| MB21D2 | 0 | 0 | 0 | 2.6 |
| MBD5 | 0 | 0 | 0 | 1.09 |
| MCC | 0 | 0 | 0 | -1.28 |
| MCCC1 | 0 | 0 | 0 | -1.51 |
| MCCC2 | 0 | 0 | 0 | -1.34 |
| MCEE | 0 | 0 | 0 | -1.48 |
| MCHR1 | 0 | 0 | 0 | -1.88 |
| MCM5 | 0 | 0 | 0 | 1.41 |
| MCM8 | 0 | 0 | 0 | 2.11 |
| MCOLN3 | 0 | 0 | 0 | 1.35 |
| MCTP1 | 0 | 0 | 0 | 1.56 |
| MCTP2 | 0 | 0 | 0 | 3.11 |
| MDM1 | 0 | 0 | 0 | 1.22 |
| MECOM | 0 | 0 | 0 | 2.84 |
| MED12L | 0 | 0 | 0 | 1.04 |
| MED25 | 0 | 0 | 0 | 1.14 |
| MED27 | 0 | 0 | 0 | 1.02 |
| MEF2B | 0 | 0 | 0 | 1.21 |
| MEF2BNB | 0 | 0 | 0 | 1.66 |
| MEGF10 | 0 | 0 | 0 | -1.18 |
| MEGF11 | 0 | 0 | 0 | 1.1 |
| MEGF6 | 0 | 0 | 0 | 1.81 |
| MEI1 | 0 | 0 | 0 | 2.23 |
| MEP1B | 0 | 0 | 0 | -2.29 |
| METRNL | 0 | 0 | 0 | 1.85 |
| METTL20 | 0 | 0 | 0 | -1.67 |
| METTL21CP1 | 0 | 0 | 0 | -1.12 |
| METTL4 | 0 | 0 | 0 | 1.23 |
| METTL8 | 0 | 0 | 0 | 1.16 |
| METTL9 | 0 | 0 | 0 | 1.54 |
| MFAP2 | 0 | 0 | 0 | 3.02 |
| MFAP5 | 0 | 0 | 0 | 3.23 |
| MGAT3 | 0 | 0 | 0 | 5.43 |
| MGAT4A | 0 | 0 | 0 | 1.5 |
| MGAT5 | 0 | 0 | 0 | 1.67 |
| MGAT5B | 0 | 0 | 0 | 2.41 |
| MGC11082 | 0 | 0 | 0 | 1.52 |
| MGC15705 | 0 | 0 | 0 | 1.54 |
| MGC24103 | 0 | 0 | 0 | 2.59 |
| MGC40069 | 0 | 0 | 0 | 1.18 |
| MGP | 0 | 0 | 0 | 2.05 |
| MGST2 | 0 | 0 | 0 | -1.79 |
| MIA | 0 | 0 | 0 | 3.96 |
| MIA2 | 0 | 0 | 0 | -3.78 |
| MIA3 | 0 | 0 | 0 | -1.64 |
| MICAL1 | 0 | 0 | 0 | 1.57 |
| MICALL1 | 0 | 0 | 0 | 1.26 |
| MICALL2 | 0 | 0 | 0 | 1.49 |
| MICB | 0 | 0 | 0 | 1.12 |
| MICU1 | 0 | 0 | 0 | -1.31 |
| MIER2 | 0 | 0 | 0 | 1.13 |
| MILR1 | 0 | 0 | 0 | 1.41 |
| MIP | 0 | 0 | 0 | -1.88 |
| MIPOL1 | 0 | 0 | 0 | 1.11 |
| MIR205HG | 0 | 0 | 0 | 5.54 |
| MIS18A | 0 | 0 | 0 | 1.46 |
| MIS18BP1 | 0 | 0 | 0 | 1.63 |
| MITD1 | 0 | 0 | 0 | 1.1 |
| MITF | 0 | 0 | 0 | 1.41 |
| MLF1 | 0 | 0 | 0 | 3.12 |
| MLKL | 0 | 0 | 0 | 1.74 |
| MLLT6 | 0 | 0 | 0 | 1.01 |
| MMAA | 0 | 0 | 0 | -1.86 |
| MMAB | 0 | 0 | 0 | -2.32 |
| MMGT1 | 0 | 0 | 0 | 1.12 |
| MMP1 | 0 | 0 | 0 | 6.82 |
| MMP10 | 0 | 0 | 0 | 3.75 |
| MMP16 | 0 | 0 | 0 | 2.24 |
| MMP2 | 0 | 0 | 0 | 2.67 |
| MMP28 | 0 | 0 | 0 | 1.17 |
| MMP9 | 0 | 0 | 0 | 2.32 |
| MMS22L | 0 | 0 | 0 | 2.37 |
| MN1 | 0 | 0 | 0 | 3.37 |
| MNS1 | 0 | 0 | 0 | 2.19 |
| MOB1A | 0 | 0 | 0 | 1.02 |
| MOB1B | 0 | 0 | 0 | -1.26 |
| MOB3A | 0 | 0 | 0 | 1.24 |
| MOB3B | 0 | 0 | 0 | 1.8 |
| MOCOS | 0 | 0 | 0 | -2.42 |
| MOCS2 | 0 | 0 | 0 | -1.08 |
| MOGAT1 | 0 | 0 | 0 | -3.43 |
| MOGAT2 | 0 | 0 | 0 | -3.94 |
| MOK | 0 | 0 | 0 | 1.36 |
| MORN3 | 0 | 0 | 0 | 1.41 |
| MOXD1 | 0 | 0 | 0 | 2.56 |
| MPDU1 | 0 | 0 | 0 | -1 |
| MPDZ | 0 | 0 | 0 | -3.05 |
| MPHOSPH6 | 0 | 0 | 0 | 1.1 |
| MPND | 0 | 0 | 0 | -1.21 |
| MPP2 | 0 | 0 | 0 | 2.39 |
| MPP3 | 0 | 0 | 0 | 2.59 |
| MPP7 | 0 | 0 | 0 | 1.9 |
| MPV17 | 0 | 0 | 0 | 1.27 |
| MPV17L | 0 | 0 | 0 | -1.87 |
| MRAP2 | 0 | 0 | 0 | 2.72 |
| MRAS | 0 | 0 | 0 | 1.32 |
| MRO | 0 | 0 | 0 | -3.21 |
| MRPL17 | 0 | 0 | 0 | 1.03 |
| MRPL46 | 0 | 0 | 0 | -1.11 |
| MRPL52 | 0 | 0 | 0 | 1.41 |
| MRPL54 | 0 | 0 | 0 | -1.15 |
| MRPS17 | 0 | 0 | 0 | -1.04 |
| MRPS23 | 0 | 0 | 0 | 1.03 |
| MRTO4 | 0 | 0 | 0 | 1.37 |
| MS4A2 | 0 | 0 | 0 | 2.62 |
| MS4A8B | 0 | 0 | 0 | 4.67 |
| MSC | 0 | 0 | 0 | 1.91 |
| MSH5 | 0 | 0 | 0 | 1.22 |
| MSL3 | 0 | 0 | 0 | 1.28 |
| MSLN | 0 | 0 | 0 | 6.32 |
| MSMP | 0 | 0 | 0 | 1.73 |
| MSN | 0 | 0 | 0 | 1.46 |
| MST4 | 0 | 0 | 0 | 1.68 |
| MSTO1 | 0 | 0 | 0 | 1.21 |
| MSX1 | 0 | 0 | 0 | 3.34 |
| MSX2 | 0 | 0 | 0 | 1.69 |
| MTBP | 0 | 0 | 0 | 2.01 |
| MTCH2 | 0 | 0 | 0 | -1.62 |
| MTHFD1L | 0 | 0 | 0 | 1.54 |
| MTHFD2 | 0 | 0 | 0 | 3.6 |
| MTHFD2L | 0 | 0 | 0 | -3.71 |
| MTMR1 | 0 | 0 | 0 | 1 |
| MTMR2 | 0 | 0 | 0 | 1.8 |
| MTMR7 | 0 | 0 | 0 | -1.91 |
| MTRNR2L3 | 0 | 0 | 0 | -1.53 |
| MTRNR2L7 | 0 | 0 | 0 | -1.74 |
| MTSS1L | 0 | 0 | 0 | -2.14 |
| MTUS2 | 0 | 0 | 0 | -3.03 |
| MUC1 | 0 | 0 | 0 | 2.82 |
| MUC12 | 0 | 0 | 0 | 3.22 |
| MUC13 | 0 | 0 | 0 | 4.96 |
| MUC16 | 0 | 0 | 0 | 3.43 |
| MUC4 | 0 | 0 | 0 | 5.04 |
| MUC5AC | 0 | 0 | 0 | 3.69 |
| MURC | 0 | 0 | 0 | 1.15 |
| MX1 | 0 | 0 | 0 | 1.23 |
| MX2 | 0 | 0 | 0 | 2.01 |
| MXD1 | 0 | 0 | 0 | 1.35 |
| MXI1 | 0 | 0 | 0 | -2.03 |
| MXRA5 | 0 | 0 | 0 | 2.43 |
| MYADM | 0 | 0 | 0 | 1.01 |
| MYB | 0 | 0 | 0 | 2.37 |
| MYBL2 | 0 | 0 | 0 | 1.66 |
| MYBPC2 | 0 | 0 | 0 | 1.62 |
| MYBPHL | 0 | 0 | 0 | 1.71 |
| MYCL1 | 0 | 0 | 0 | -2.01 |
| MYCN | 0 | 0 | 0 | 1.94 |
| MYCT1 | 0 | 0 | 0 | -1.28 |
| MYEF2 | 0 | 0 | 0 | 3.13 |
| MYH6 | 0 | 0 | 0 | 1.78 |
| MYH7B | 0 | 0 | 0 | -1.29 |
| MYH9 | 0 | 0 | 0 | 1.05 |
| MYL10 | 0 | 0 | 0 | 1.54 |
| MYL3 | 0 | 0 | 0 | -1.43 |
| MYLIP | 0 | 0 | 0 | 1.4 |
| MYO15A | 0 | 0 | 0 | -1.96 |
| MYO16 | 0 | 0 | 0 | -4 |
| MYO1D | 0 | 0 | 0 | 1.39 |
| MYO1G | 0 | 0 | 0 | 1.94 |
| MYO5A | 0 | 0 | 0 | 1.5 |
| MYO5C | 0 | 0 | 0 | 1.58 |
| MYO9B | 0 | 0 | 0 | 1.09 |
| MYOM1 | 0 | 0 | 0 | -2.58 |
| MYOM2 | 0 | 0 | 0 | -1.56 |
| MYOM3 | 0 | 0 | 0 | 2.89 |
| NAALADL1 | 0 | 0 | 0 | 1.12 |
| NACAD | 0 | 0 | 0 | 1.3 |
| NAMPT | 0 | 0 | 0 | -1.45 |
| NANOS3 | 0 | 0 | 0 | 1.1 |
| NANS | 0 | 0 | 0 | 1.16 |
| NAP1L1 | 0 | 0 | 0 | 1.21 |
| NAT1 | 0 | 0 | 0 | -1.29 |
| NAT8L | 0 | 0 | 0 | 2.45 |
| NAV2 | 0 | 0 | 0 | -1.55 |
| NAV3 | 0 | 0 | 0 | 2.1 |
| NBEA | 0 | 0 | 0 | 2.32 |
| NBEAL2 | 0 | 0 | 0 | 1.26 |
| NBPF3 | 0 | 0 | 0 | -2.55 |
| NCAM1 | 0 | 0 | 0 | -1.7 |
| NCAPD2 | 0 | 0 | 0 | 1.96 |
| NCAPG2 | 0 | 0 | 0 | 2.12 |
| NCAPH2 | 0 | 0 | 0 | 1.01 |
| NCDN | 0 | 0 | 0 | 1.41 |
| NCK2 | 0 | 0 | 0 | 1.48 |
| NCKAP5 | 0 | 0 | 0 | -1.81 |
| NCL | 0 | 0 | 0 | 1.06 |
| NCOA3 | 0 | 0 | 0 | 1.06 |
| NCOA4 | 0 | 0 | 0 | -1.22 |
| NCS1 | 0 | 0 | 0 | 2.29 |
| NDC80 | 0 | 0 | 0 | 3.57 |
| NDE1 | 0 | 0 | 0 | 2.19 |
| NDFIP1 | 0 | 0 | 0 | -1.09 |
| NDOR1 | 0 | 0 | 0 | 1.08 |
| NDRG1 | 0 | 0 | 0 | 1.97 |
| NDST1 | 0 | 0 | 0 | -1.18 |
| NDST3 | 0 | 0 | 0 | -3.8 |
| NDUFA4L2 | 0 | 0 | 0 | 1.78 |
| NDUFAF1 | 0 | 0 | 0 | -1.03 |
| NDUFC2 | 0 | 0 | 0 | -1.29 |
| NDUFS7 | 0 | 0 | 0 | -1.06 |
| NEBL | 0 | 0 | 0 | 2.75 |
| NEDD1 | 0 | 0 | 0 | 1.57 |
| NEDD9 | 0 | 0 | 0 | 1.64 |
| NEIL1 | 0 | 0 | 0 | -1.72 |
| NEIL3 | 0 | 0 | 0 | 2.6 |
| NEK5 | 0 | 0 | 0 | 1.21 |
| NEK8 | 0 | 0 | 0 | 1.38 |
| NELL2 | 0 | 0 | 0 | 2.38 |
| NET1 | 0 | 0 | 0 | 1.12 |
| NETO2 | 0 | 0 | 0 | 2.96 |
| NFIC | 0 | 0 | 0 | -1.33 |
| NFKBID | 0 | 0 | 0 | 1.48 |
| NHS | 0 | 0 | 0 | 3.86 |
| NID2 | 0 | 0 | 0 | 1.85 |
| NIN | 0 | 0 | 0 | 1.01 |
| NIPAL1 | 0 | 0 | 0 | -1.77 |
| NIPAL3 | 0 | 0 | 0 | 1.52 |
| NIPAL4 | 0 | 0 | 0 | 3.68 |
| NIPSNAP1 | 0 | 0 | 0 | -2.35 |
| NIT2 | 0 | 0 | 0 | -1.61 |
| NKD2 | 0 | 0 | 0 | 2.18 |
| NKIRAS1 | 0 | 0 | 0 | -1.11 |
| NKX3-2 | 0 | 0 | 0 | 3.92 |
| NLGN3 | 0 | 0 | 0 | 1.24 |
| NLRC5 | 0 | 0 | 0 | 1.4 |
| NLRP11 | 0 | 0 | 0 | -1.52 |
| NLRP2 | 0 | 0 | 0 | 3.6 |
| NLRP7 | 0 | 0 | 0 | 2.26 |
| NME2 | 0 | 0 | 0 | 1.36 |
| NMI | 0 | 0 | 0 | 1.01 |
| NMNAT2 | 0 | 0 | 0 | 2.69 |
| NMU | 0 | 0 | 0 | 6.93 |
| NNMT | 0 | 0 | 0 | -1.94 |
| NOD1 | 0 | 0 | 0 | 1.71 |
| NOG | 0 | 0 | 0 | 2.94 |
| NOL12 | 0 | 0 | 0 | 1.03 |
| NOL4 | 0 | 0 | 0 | -3.32 |
| NOP2 | 0 | 0 | 0 | 1.06 |
| NOP56 | 0 | 0 | 0 | 1.17 |
| NOS1AP | 0 | 0 | 0 | -1.34 |
| NOTCH3 | 0 | 0 | 0 | 2.85 |
| NOX1 | 0 | 0 | 0 | 2.38 |
| NPC1L1 | 0 | 0 | 0 | -3.08 |
| NPC2 | 0 | 0 | 0 | 1.08 |
| NPDC1 | 0 | 0 | 0 | 1.13 |
| NPEPL1 | 0 | 0 | 0 | 1.45 |
| NPEPPS | 0 | 0 | 0 | 1.01 |
| NPHP4 | 0 | 0 | 0 | 1.85 |
| NPM1 | 0 | 0 | 0 | 1.14 |
| NPNT | 0 | 0 | 0 | 1.65 |
| NPSA | 0 | 0 | 0 | -1.1 |
| NPTN | 0 | 0 | 0 | 1.17 |
| NPY1R | 0 | 0 | 0 | -1.43 |
| NQO1 | 0 | 0 | 0 | 5.5 |
| NQO2 | 0 | 0 | 0 | -1.48 |
| NR3C2 | 0 | 0 | 0 | -2.01 |
| NRAP | 0 | 0 | 0 | -5.49 |
| NRARP | 0 | 0 | 0 | 2.24 |
| NRBP2 | 0 | 0 | 0 | -1.95 |
| NRG4 | 0 | 0 | 0 | -2.22 |
| NRIP3 | 0 | 0 | 0 | 1.71 |
| NRL | 0 | 0 | 0 | -1.13 |
| NRM | 0 | 0 | 0 | 1.41 |
| NRSN2 | 0 | 0 | 0 | 1.89 |
| NRTN | 0 | 0 | 0 | -3.74 |
| NSAP11 | 0 | 0 | 0 | -1.91 |
| NSMAF | 0 | 0 | 0 | 1.63 |
| NT5C | 0 | 0 | 0 | 1.02 |
| NT5C3 | 0 | 0 | 0 | 1.44 |
| NT5DC1 | 0 | 0 | 0 | -1 |
| NT5DC4 | 0 | 0 | 0 | 2.04 |
| NTN1 | 0 | 0 | 0 | -3.34 |
| NTNG2 | 0 | 0 | 0 | 1.97 |
| NUAK2 | 0 | 0 | 0 | 1.03 |
| NUDT1 | 0 | 0 | 0 | 2.41 |
| NUDT12 | 0 | 0 | 0 | -1.22 |
| NUDT13 | 0 | 0 | 0 | -1.39 |
| NUDT16 | 0 | 0 | 0 | -1.16 |
| NUDT17 | 0 | 0 | 0 | 1.04 |
| NUDT21 | 0 | 0 | 0 | 1.26 |
| NUDT6 | 0 | 0 | 0 | -1.51 |
| NUDT8 | 0 | 0 | 0 | -1.42 |
| NUMBL | 0 | 0 | 0 | 1.56 |
| NUP155 | 0 | 0 | 0 | 1.12 |
| NUP205 | 0 | 0 | 0 | 1.23 |
| NUP43 | 0 | 0 | 0 | 1.09 |
| NUP62 | 0 | 0 | 0 | 1.39 |
| NUP93 | 0 | 0 | 0 | 1.76 |
| NUTF2 | 0 | 0 | 0 | 1.04 |
| NVL | 0 | 0 | 0 | 1.02 |
| NXN | 0 | 0 | 0 | 1.17 |
| NXNL2 | 0 | 0 | 0 | 1.42 |
| NXT1 | 0 | 0 | 0 | 1.39 |
| OAS3 | 0 | 0 | 0 | 1.51 |
| OBFC1 | 0 | 0 | 0 | 1.25 |
| OBFC2A | 0 | 0 | 0 | 1.28 |
| OBSCN | 0 | 0 | 0 | 2.4 |
| OBSL1 | 0 | 0 | 0 | -1.12 |
| ODAM | 0 | 0 | 0 | 2.88 |
| ODF2 | 0 | 0 | 0 | 1.06 |
| ODF2L | 0 | 0 | 0 | 2.63 |
| ODZ4 | 0 | 0 | 0 | 2.64 |
| OGFRL1 | 0 | 0 | 0 | 1.57 |
| OK/SW-CL.36 | 0 | 0 | 0 | -1.32 |
| OLA1 | 0 | 0 | 0 | 1.13 |
| OLFM2 | 0 | 0 | 0 | -2.54 |
| OLFM4 | 0 | 0 | 0 | 7.6 |
| OLFML2A | 0 | 0 | 0 | 2.28 |
| ONECUT1 | 0 | 0 | 0 | -6.05 |
| OPHN1 | 0 | 0 | 0 | 1.29 |
| OPLAH | 0 | 0 | 0 | -1.83 |
| OR11H6 | 0 | 0 | 0 | -1.03 |
| OR13A1 | 0 | 0 | 0 | 2.44 |
| OR13C4 | 0 | 0 | 0 | -1.19 |
| OR13C5 | 0 | 0 | 0 | -1.67 |
| OR1E1 | 0 | 0 | 0 | -1.95 |
| OR2D2 | 0 | 0 | 0 | 1.33 |
| OR2D3 | 0 | 0 | 0 | -1.17 |
| OR2T1 | 0 | 0 | 0 | -1.12 |
| OR4D10 | 0 | 0 | 0 | -2.29 |
| OR4N4 | 0 | 0 | 0 | 2.26 |
| OR51E1 | 0 | 0 | 0 | 3.03 |
| OR51E2 | 0 | 0 | 0 | 1.12 |
| OR52M1 | 0 | 0 | 0 | -1.02 |
| ORAI3 | 0 | 0 | 0 | -1.1 |
| ORC6 | 0 | 0 | 0 | 2.6 |
| OSBP2 | 0 | 0 | 0 | 1.6 |
| OSBPL1A | 0 | 0 | 0 | 1.28 |
| OSBPL5 | 0 | 0 | 0 | 1.99 |
| OSBPL6 | 0 | 0 | 0 | -2.6 |
| OSBPL7 | 0 | 0 | 0 | 2.96 |
| OSCAR | 0 | 0 | 0 | -1.75 |
| OSM | 0 | 0 | 0 | 2.28 |
| OSR2 | 0 | 0 | 0 | 1.86 |
| OTUD3 | 0 | 0 | 0 | 1.22 |
| OTUD6A | 0 | 0 | 0 | -1.37 |
| OTX1 | 0 | 0 | 0 | 2.17 |
| OVGP1 | 0 | 0 | 0 | -2.11 |
| OVOL1 | 0 | 0 | 0 | 4.98 |
| OVOL2 | 0 | 0 | 0 | 4.06 |
| OXCT1 | 0 | 0 | 0 | 2.73 |
| P2RX5 | 0 | 0 | 0 | 2.05 |
| P2RY12 | 0 | 0 | 0 | -1.64 |
| P2RY6 | 0 | 0 | 0 | 1.47 |
| P39189 | 0 | 0 | 0 | -3.9 |
| PABPC3 | 0 | 0 | 0 | 1.02 |
| PABPC4L | 0 | 0 | 0 | 1.74 |
| PACRG | 0 | 0 | 0 | -1.06 |
| PACRGL | 0 | 0 | 0 | 1.04 |
| PACS1 | 0 | 0 | 0 | 1.42 |
| PACSIN3 | 0 | 0 | 0 | -1.57 |
| PADI1 | 0 | 0 | 0 | 6.36 |
| PAEP | 0 | 0 | 0 | 2.75 |
| PAGE2 | 0 | 0 | 0 | -1.13 |
| PAGE2B | 0 | 0 | 0 | -1.04 |
| PAGE4 | 0 | 0 | 0 | -2.02 |
| PALM3 | 0 | 0 | 0 | -2.85 |
| PAM | 0 | 0 | 0 | 1.57 |
| PAPLN | 0 | 0 | 0 | 1.47 |
| PAPOLG | 0 | 0 | 0 | 1.12 |
| PAPPA | 0 | 0 | 0 | 2.71 |
| PARP12 | 0 | 0 | 0 | 1.07 |
| PARP8 | 0 | 0 | 0 | 1.38 |
| PARVA | 0 | 0 | 0 | 1.04 |
| PARVB | 0 | 0 | 0 | 1.39 |
| PASK | 0 | 0 | 0 | 1.11 |
| PATL2 | 0 | 0 | 0 | 1.26 |
| PAX6 | 0 | 0 | 0 | 4.2 |
| PAX8 | 0 | 0 | 0 | 1.58 |
| PAX9 | 0 | 0 | 0 | 2.69 |
| PBX1 | 0 | 0 | 0 | 1.05 |
| PCBD1 | 0 | 0 | 0 | -2.28 |
| PCBP3 | 0 | 0 | 0 | 1.32 |
| PCBP3-OT1 | 0 | 0 | 0 | 2.23 |
| PCCA | 0 | 0 | 0 | -2.79 |
| PCDH17 | 0 | 0 | 0 | 1.34 |
| PCDH20 | 0 | 0 | 0 | -2.91 |
| PCDHA10 | 0 | 0 | 0 | 1.4 |
| PCDHA11 | 0 | 0 | 0 | 1.45 |
| PCDHA12 | 0 | 0 | 0 | 2.32 |
| PCDHA4 | 0 | 0 | 0 | 2.24 |
| PCDHA6 | 0 | 0 | 0 | 1.98 |
| PCDHB11 | 0 | 0 | 0 | 1.09 |
| PCDHB14 | 0 | 0 | 0 | 1.55 |
| PCNXL2 | 0 | 0 | 0 | 2.97 |
| PCP4L1 | 0 | 0 | 0 | -2.67 |
| PCSK1 | 0 | 0 | 0 | 2.64 |
| PCSK5 | 0 | 0 | 0 | 3.02 |
| PCSK7 | 0 | 0 | 0 | 1.04 |
| PCSK9 | 0 | 0 | 0 | -2.26 |
| PCTP | 0 | 0 | 0 | -2.19 |
| PCYOX1 | 0 | 0 | 0 | -1.81 |
| PCYOX1L | 0 | 0 | 0 | 2.42 |
| PDCD2L | 0 | 0 | 0 | 1.15 |
| PDCL | 0 | 0 | 0 | 1.07 |
| PDE10A | 0 | 0 | 0 | 1.8 |
| PDE11A | 0 | 0 | 0 | -4.36 |
| PDE4A | 0 | 0 | 0 | 1.58 |
| PDE4DIP | 0 | 0 | 0 | -1.2 |
| PDE5A | 0 | 0 | 0 | 1.99 |
| PDE6C | 0 | 0 | 0 | -1.15 |
| PDE8A | 0 | 0 | 0 | -1.03 |
| PDGFA | 0 | 0 | 0 | 2.19 |
| PDGFRB | 0 | 0 | 0 | 1.78 |
| PDIA5 | 0 | 0 | 0 | -1.42 |
| PDK1 | 0 | 0 | 0 | 1.35 |
| PDLIM3 | 0 | 0 | 0 | 2.62 |
| PDLIM4 | 0 | 0 | 0 | 2.76 |
| PDP1 | 0 | 0 | 0 | 1.9 |
| PDZD3 | 0 | 0 | 0 | 3.61 |
| PDZRN3 | 0 | 0 | 0 | 2.16 |
| PEA15 | 0 | 0 | 0 | 1.66 |
| PEAK1 | 0 | 0 | 0 | 1.21 |
| PELI1 | 0 | 0 | 0 | 1.53 |
| PELI2 | 0 | 0 | 0 | 1.31 |
| PEPD | 0 | 0 | 0 | -1.44 |
| PER3 | 0 | 0 | 0 | -1.08 |
| PEX19 | 0 | 0 | 0 | -1.28 |
| PFDN1 | 0 | 0 | 0 | 1 |
| PFDN4 | 0 | 0 | 0 | 1.54 |
| PFKFB2 | 0 | 0 | 0 | 1.89 |
| PFKFB3 | 0 | 0 | 0 | 1.19 |
| PFKM | 0 | 0 | 0 | 1.73 |
| PFN3 | 0 | 0 | 0 | -2.8 |
| PGCP | 0 | 0 | 0 | -1.08 |
| PGK1 | 0 | 0 | 0 | 1.02 |
| PGP | 0 | 0 | 0 | -1.07 |
| PGPEP1 | 0 | 0 | 0 | -1.33 |
| PGPEP1L | 0 | 0 | 0 | -1.35 |
| PGS1 | 0 | 0 | 0 | 1.13 |
| PHEX | 0 | 0 | 0 | 2.19 |
| PHF15 | 0 | 0 | 0 | 1.39 |
| PHF6 | 0 | 0 | 0 | 1.01 |
| PHGDH | 0 | 0 | 0 | -3 |
| PHIP | 0 | 0 | 0 | 1.01 |
| PHKA2 | 0 | 0 | 0 | -1.64 |
| PHLDB1 | 0 | 0 | 0 | 1.92 |
| PHLDB3 | 0 | 0 | 0 | -1.29 |
| PHLPP1 | 0 | 0 | 0 | -2.08 |
| PHTF2 | 0 | 0 | 0 | 1.12 |
| PHYHIPL | 0 | 0 | 0 | -1.43 |
| PI15 | 0 | 0 | 0 | 3.93 |
| PI4K2B | 0 | 0 | 0 | -1.06 |
| PIAS3 | 0 | 0 | 0 | 1.45 |
| PIEZO1 | 0 | 0 | 0 | 1.19 |
| PIGS | 0 | 0 | 0 | 1.24 |
| PIGV | 0 | 0 | 0 | -1.1 |
| PIGW | 0 | 0 | 0 | 1.04 |
| PIGX | 0 | 0 | 0 | 1.07 |
| PIK3C2B | 0 | 0 | 0 | 1.05 |
| PIK3C2G | 0 | 0 | 0 | -2.87 |
| PIK3CG | 0 | 0 | 0 | 1.42 |
| PIK3IP1 | 0 | 0 | 0 | 1.42 |
| PIM2 | 0 | 0 | 0 | 2.07 |
| PINK1 | 0 | 0 | 0 | -1.51 |
| PIP5K1B | 0 | 0 | 0 | 4.28 |
| PIP5K1C | 0 | 0 | 0 | 1.24 |
| PIP5KL1 | 0 | 0 | 0 | 1.71 |
| PITPNM2 | 0 | 0 | 0 | -1.81 |
| PITX2 | 0 | 0 | 0 | 3.03 |
| PIWIL4 | 0 | 0 | 0 | 1.99 |
| PJA1 | 0 | 0 | 0 | 1.14 |
| PKDCC | 0 | 0 | 0 | 1.79 |
| PKHD1 | 0 | 0 | 0 | -2.87 |
| PKIA | 0 | 0 | 0 | 2.13 |
| PKN3 | 0 | 0 | 0 | 1.74 |
| PLA1A | 0 | 0 | 0 | -2.63 |
| PLA2G10 | 0 | 0 | 0 | 5.54 |
| PLA2G12A | 0 | 0 | 0 | -1.15 |
| PLA2G4A | 0 | 0 | 0 | 2.65 |
| PLA2G4D | 0 | 0 | 0 | 2.04 |
| PLA2R1 | 0 | 0 | 0 | 1.9 |
| PLAC1 | 0 | 0 | 0 | 1.37 |
| PLAG1 | 0 | 0 | 0 | 1.76 |
| PLAGL2 | 0 | 0 | 0 | 1 |
| PLAT | 0 | 0 | 0 | 3.39 |
| PLAUR | 0 | 0 | 0 | 3.02 |
| PLB1 | 0 | 0 | 0 | 1.56 |
| PLBD1 | 0 | 0 | 0 | 3.16 |
| PLCB1 | 0 | 0 | 0 | 1.02 |
| PLCB3 | 0 | 0 | 0 | 1.89 |
| PLCB4 | 0 | 0 | 0 | 3.22 |
| PLCD3 | 0 | 0 | 0 | 3.78 |
| PLCE1 | 0 | 0 | 0 | 2.39 |
| PLCG1 | 0 | 0 | 0 | 1.2 |
| PLCH1 | 0 | 0 | 0 | 2.86 |
| PLEC | 0 | 0 | 0 | 1.15 |
| PLEKHA5 | 0 | 0 | 0 | -1.71 |
| PLEKHA8 | 0 | 0 | 0 | 1.34 |
| PLEKHB1 | 0 | 0 | 0 | 2.07 |
| PLEKHG2 | 0 | 0 | 0 | 1.7 |
| PLEKHG4 | 0 | 0 | 0 | 3.17 |
| PLEKHG5 | 0 | 0 | 0 | 1.32 |
| PLEKHH2 | 0 | 0 | 0 | 1.55 |
| PLEKHM1 | 0 | 0 | 0 | 1.09 |
| PLEKHN1 | 0 | 0 | 0 | 2.8 |
| PLEKHO1 | 0 | 0 | 0 | 1.32 |
| PLSCR1 | 0 | 0 | 0 | 1.07 |
| PLSCR4 | 0 | 0 | 0 | -1 |
| PLXDC1 | 0 | 0 | 0 | 1.45 |
| PLXDC2 | 0 | 0 | 0 | 1.97 |
| PLXNA4 | 0 | 0 | 0 | 1.6 |
| PLXNB1 | 0 | 0 | 0 | -1.3 |
| PLXNB3 | 0 | 0 | 0 | 2.46 |
| PLXNC1 | 0 | 0 | 0 | 2.28 |
| PM20D2 | 0 | 0 | 0 | 1.17 |
| PMCH | 0 | 0 | 0 | 2.61 |
| PMFBP1 | 0 | 0 | 0 | 1.67 |
| PMM1 | 0 | 0 | 0 | -1.08 |
| PMPCA | 0 | 0 | 0 | -1.14 |
| PNMA1 | 0 | 0 | 0 | 2.42 |
| PNMA5 | 0 | 0 | 0 | -2.11 |
| PNMA6A | 0 | 0 | 0 | -1.84 |
| PNMA6C | 0 | 0 | 0 | -3.14 |
| PNPLA5 | 0 | 0 | 0 | -1.01 |
| POC5 | 0 | 0 | 0 | 1.13 |
| PODXL | 0 | 0 | 0 | 2.32 |
| POF1B | 0 | 0 | 0 | 2.95 |
| POLA1 | 0 | 0 | 0 | 1.46 |
| POLA2 | 0 | 0 | 0 | 1.59 |
| POLD3 | 0 | 0 | 0 | 1.4 |
| POLR1A | 0 | 0 | 0 | 1.03 |
| POLR1E | 0 | 0 | 0 | -1.45 |
| POLR2E | 0 | 0 | 0 | -1.87 |
| POLR2G | 0 | 0 | 0 | 1.03 |
| POLR3K | 0 | 0 | 0 | 1.02 |
| POTEB | 0 | 0 | 0 | 2.77 |
| POTED | 0 | 0 | 0 | 3.39 |
| POTEE | 0 | 0 | 0 | 1.51 |
| POTEF | 0 | 0 | 0 | 1.56 |
| POTEM | 0 | 0 | 0 | 1.53 |
| POU2AF1 | 0 | 0 | 0 | 2.71 |
| POU5F1 | 0 | 0 | 0 | 1.04 |
| PPAP2C | 0 | 0 | 0 | 1.95 |
| PPAPDC1B | 0 | 0 | 0 | 1.14 |
| PPARGC1A | 0 | 0 | 0 | -4.02 |
| PPAT | 0 | 0 | 0 | 1.11 |
| PPDPF | 0 | 0 | 0 | 1.9 |
| PPFIA4 | 0 | 0 | 0 | 3.01 |
| PPIF | 0 | 0 | 0 | -1.22 |
| PPIH | 0 | 0 | 0 | 1.36 |
| PPIL1 | 0 | 0 | 0 | 1.01 |
| PPIL6 | 0 | 0 | 0 | 1.47 |
| PPIP5K1 | 0 | 0 | 0 | 1.26 |
| PPM1J | 0 | 0 | 0 | 1.15 |
| PPM1M | 0 | 0 | 0 | 1.06 |
| PPM1N | 0 | 0 | 0 | 1.13 |
| PPME1 | 0 | 0 | 0 | 1.03 |
| PPP1CC | 0 | 0 | 0 | 1.03 |
| PPP1R12A | 0 | 0 | 0 | 1.14 |
| PPP1R16A | 0 | 0 | 0 | -1 |
| PPP1R18 | 0 | 0 | 0 | 1.2 |
| PPP1R1A | 0 | 0 | 0 | -4.3 |
| PPP1R1B | 0 | 0 | 0 | 3.87 |
| PPP1R1C | 0 | 0 | 0 | -2.14 |
| PPP1R2 | 0 | 0 | 0 | 1.01 |
| PPP1R27 | 0 | 0 | 0 | -1.57 |
| PPP1R32 | 0 | 0 | 0 | -1.63 |
| PPP1R36 | 0 | 0 | 0 | 2.88 |
| PPP1R37 | 0 | 0 | 0 | 1.24 |
| PPP1R3B | 0 | 0 | 0 | -2.5 |
| PPP1R3D | 0 | 0 | 0 | 1.23 |
| PPP1R3G | 0 | 0 | 0 | -1.28 |
| PPP1R9A | 0 | 0 | 0 | 2.25 |
| PPP1R9B | 0 | 0 | 0 | 1.38 |
| PPP2R1A | 0 | 0 | 0 | 1.09 |
| PPP4R1 | 0 | 0 | 0 | 1.19 |
| PPP4R4 | 0 | 0 | 0 | -1.39 |
| PPPDE1 | 0 | 0 | 0 | 1.18 |
| PPT1 | 0 | 0 | 0 | 1.06 |
| PQLC1 | 0 | 0 | 0 | -2.08 |
| PQLC3 | 0 | 0 | 0 | 1.17 |
| PRADC1 | 0 | 0 | 0 | -1.49 |
| PRAF2 | 0 | 0 | 0 | 1.05 |
| PRAME | 0 | 0 | 0 | 2.48 |
| PRAMEF10 | 0 | 0 | 0 | -3.35 |
| PRAMEF13 | 0 | 0 | 0 | -1.9 |
| PRAMEF7 | 0 | 0 | 0 | -1.3 |
| PRDM1 | 0 | 0 | 0 | 2.52 |
| PRDM13 | 0 | 0 | 0 | -1.01 |
| PRDM15 | 0 | 0 | 0 | 1.15 |
| PRDM5 | 0 | 0 | 0 | 1.91 |
| PRDM6 | 0 | 0 | 0 | 1.49 |
| PRDM8 | 0 | 0 | 0 | 1.04 |
| PRDX3 | 0 | 0 | 0 | -1.45 |
| PRDX6 | 0 | 0 | 0 | -1.67 |
| PRELID2 | 0 | 0 | 0 | 1.93 |
| PRG2 | 0 | 0 | 0 | -2.2 |
| PRICKLE1 | 0 | 0 | 0 | 1.81 |
| PRIM2 | 0 | 0 | 0 | 1.73 |
| PRKAB1 | 0 | 0 | 0 | 1.24 |
| PRKAB2 | 0 | 0 | 0 | -1.21 |
| PRKACB | 0 | 0 | 0 | 1.37 |
| PRKAG2 | 0 | 0 | 0 | -1.04 |
| PRKCD | 0 | 0 | 0 | 1.11 |
| PRKCDBP | 0 | 0 | 0 | 2.6 |
| PRKCI | 0 | 0 | 0 | 1.33 |
| PRKCQ | 0 | 0 | 0 | 1.31 |
| PRKD2 | 0 | 0 | 0 | 1.09 |
| PRKDC | 0 | 0 | 0 | 1.34 |
| PRKX | 0 | 0 | 0 | 1.48 |
| PRKXP1 | 0 | 0 | 0 | 1.24 |
| PRLR | 0 | 0 | 0 | -2.68 |
| PRMT1 | 0 | 0 | 0 | 1.44 |
| PRMT2 | 0 | 0 | 0 | 1.13 |
| PRMT3 | 0 | 0 | 0 | 1.19 |
| PROCA1 | 0 | 0 | 0 | 1.04 |
| PROCR | 0 | 0 | 0 | 2.43 |
| PROKR1 | 0 | 0 | 0 | -1.61 |
| PROM2 | 0 | 0 | 0 | 2.73 |
| PROX1 | 0 | 0 | 0 | -5.41 |
| PRR12 | 0 | 0 | 0 | 1.18 |
| PRR15L | 0 | 0 | 0 | 3.43 |
| PRR16 | 0 | 0 | 0 | 3.16 |
| PRR18 | 0 | 0 | 0 | -3.31 |
| PRR19 | 0 | 0 | 0 | 1.8 |
| PRR22 | 0 | 0 | 0 | -1.42 |
| PRR5-ARHGAP8 | 0 | 0 | 0 | 2.03 |
| PRR5L | 0 | 0 | 0 | 1.86 |
| PRRC2A | 0 | 0 | 0 | 1.08 |
| PRRT3 | 0 | 0 | 0 | 1.08 |
| PRRX1 | 0 | 0 | 0 | 4.38 |
| PRRX2 | 0 | 0 | 0 | 4.81 |
| PRSS12 | 0 | 0 | 0 | 2.27 |
| PRSS16 | 0 | 0 | 0 | 2.22 |
| PRSS23 | 0 | 0 | 0 | 2.22 |
| PRSS42 | 0 | 0 | 0 | -1.94 |
| PRSS45 | 0 | 0 | 0 | -2.89 |
| PRSS50 | 0 | 0 | 0 | -2.64 |
| PRSS54 | 0 | 0 | 0 | 1.1 |
| PSCA | 0 | 0 | 0 | 6.4 |
| PSMB8 | 0 | 0 | 0 | 1.15 |
| PSMB9 | 0 | 0 | 0 | 1.96 |
| PSME3 | 0 | 0 | 0 | 1.18 |
| PSMG3 | 0 | 0 | 0 | 1.46 |
| PSORS1C1 | 0 | 0 | 0 | 2.45 |
| PSPC1 | 0 | 0 | 0 | 1.12 |
| PSRC1 | 0 | 0 | 0 | 1.91 |
| PSTPIP1 | 0 | 0 | 0 | 1.22 |
| PTAFR | 0 | 0 | 0 | 1.58 |
| PTCH2 | 0 | 0 | 0 | -1.08 |
| PTGER3 | 0 | 0 | 0 | 2.46 |
| PTGER4 | 0 | 0 | 0 | 1.32 |
| PTGES | 0 | 0 | 0 | 3.95 |
| PTGR2 | 0 | 0 | 0 | -1.37 |
| PTGS1 | 0 | 0 | 0 | 1.43 |
| PTP4A3 | 0 | 0 | 0 | 1.89 |
| PTPDC1 | 0 | 0 | 0 | 1.65 |
| PTPLA | 0 | 0 | 0 | 1.63 |
| PTPLAD2 | 0 | 0 | 0 | 1.38 |
| PTPN1 | 0 | 0 | 0 | 1.13 |
| PTPN12 | 0 | 0 | 0 | 1.58 |
| PTPN13 | 0 | 0 | 0 | 1.59 |
| PTPN3 | 0 | 0 | 0 | -2.88 |
| PTPN7 | 0 | 0 | 0 | 1.55 |
| PTPRA | 0 | 0 | 0 | 1.01 |
| PTPRD | 0 | 0 | 0 | -2.4 |
| PTPRE | 0 | 0 | 0 | 1.14 |
| PTPRN2 | 0 | 0 | 0 | 2.57 |
| PTPRO | 0 | 0 | 0 | 1.04 |
| PTPRR | 0 | 0 | 0 | 5.6 |
| PTPRT | 0 | 0 | 0 | -1.42 |
| PUS10 | 0 | 0 | 0 | -1.39 |
| PVALB | 0 | 0 | 0 | -3.07 |
| PVRL1 | 0 | 0 | 0 | 1.34 |
| PVRL3 | 0 | 0 | 0 | -1.36 |
| PWWP2A | 0 | 0 | 0 | 1.12 |
| PXN | 0 | 0 | 0 | 1.01 |
| PYDC1 | 0 | 0 | 0 | 2.09 |
| PYGL | 0 | 0 | 0 | -2.46 |
| PYGO1 | 0 | 0 | 0 | 1.79 |
| PZP | 0 | 0 | 0 | -5.98 |
| Q46FH2 | 0 | 0 | 0 | 1.33 |
| Q8IWW8 | 0 | 0 | 0 | -4.35 |
| Q8YNJ1 | 0 | 0 | 0 | -1.34 |
| Q9BVX4 | 0 | 0 | 0 | -1.82 |
| QPCT | 0 | 0 | 0 | 1.62 |
| QSER1 | 0 | 0 | 0 | 1.07 |
| QSOX1 | 0 | 0 | 0 | 1.01 |
| QTRTD1 | 0 | 0 | 0 | 1.07 |
| R3HDM1 | 0 | 0 | 0 | 1.17 |
| RAB11A | 0 | 0 | 0 | 1.02 |
| RAB11FIP1 | 0 | 0 | 0 | 1.56 |
| RAB11FIP5 | 0 | 0 | 0 | 1.01 |
| RAB19 | 0 | 0 | 0 | 1.65 |
| RAB23 | 0 | 0 | 0 | 1.82 |
| RAB25 | 0 | 0 | 0 | 4.93 |
| RAB31 | 0 | 0 | 0 | 2.43 |
| RAB33A | 0 | 0 | 0 | 1.47 |
| RAB36 | 0 | 0 | 0 | 2.07 |
| RAB3D | 0 | 0 | 0 | 2.32 |
| RAB3GAP1 | 0 | 0 | 0 | 1.01 |
| RAB43 | 0 | 0 | 0 | -1.37 |
| RAB44 | 0 | 0 | 0 | 2.74 |
| RAB7B | 0 | 0 | 0 | 1.84 |
| RABEP2 | 0 | 0 | 0 | 1.08 |
| RAC2 | 0 | 0 | 0 | 1.43 |
| RAD51 | 0 | 0 | 0 | 3.4 |
| RAD51B | 0 | 0 | 0 | 1.04 |
| RAD51D | 0 | 0 | 0 | 1.17 |
| RAD54L | 0 | 0 | 0 | 4.37 |
| RADIL | 0 | 0 | 0 | 1.96 |
| RAET1E | 0 | 0 | 0 | 2.21 |
| RALA | 0 | 0 | 0 | 1.44 |
| RALGAPA2 | 0 | 0 | 0 | -1.28 |
| RALGPS1 | 0 | 0 | 0 | 2.02 |
| RALGPS2 | 0 | 0 | 0 | -1.24 |
| RALY | 0 | 0 | 0 | 1.23 |
| RAMP1 | 0 | 0 | 0 | -1.24 |
| RAMP3 | 0 | 0 | 0 | -1.2 |
| RAN | 0 | 0 | 0 | 1.54 |
| RANBP10 | 0 | 0 | 0 | -1.03 |
| RANBP17 | 0 | 0 | 0 | 1.58 |
| RAP1GAP2 | 0 | 0 | 0 | 1.76 |
| RAP2A | 0 | 0 | 0 | 1.09 |
| RAPGEF4 | 0 | 0 | 0 | -2.22 |
| RAPGEF6 | 0 | 0 | 0 | 1.52 |
| RAPGEFL1 | 0 | 0 | 0 | 1.97 |
| RARG | 0 | 0 | 0 | 1.69 |
| RARRES1 | 0 | 0 | 0 | 3.72 |
| RASA2 | 0 | 0 | 0 | 1.16 |
| RASA3 | 0 | 0 | 0 | 2.12 |
| RASA4 | 0 | 0 | 0 | 1.47 |
| RASAL1 | 0 | 0 | 0 | 2.61 |
| RASGEF1A | 0 | 0 | 0 | 2.17 |
| RASGEF1B | 0 | 0 | 0 | -1.48 |
| RASGRF2 | 0 | 0 | 0 | 1.19 |
| RASGRP1 | 0 | 0 | 0 | 2.03 |
| RASL11B | 0 | 0 | 0 | 1.54 |
| RASSF10 | 0 | 0 | 0 | 3.81 |
| RASSF3 | 0 | 0 | 0 | 1 |
| RASSF6 | 0 | 0 | 0 | 1.77 |
| RAVER1 | 0 | 0 | 0 | 1.66 |
| RAVER2 | 0 | 0 | 0 | 2.04 |
| RB1 | 0 | 0 | 0 | 1.05 |
| RBKS | 0 | 0 | 0 | -2.55 |
| RBL1 | 0 | 0 | 0 | 1.72 |
| RBM11 | 0 | 0 | 0 | 1.37 |
| RBM38 | 0 | 0 | 0 | 1.12 |
| RBMX | 0 | 0 | 0 | 1.05 |
| RBMX2 | 0 | 0 | 0 | 1.61 |
| RBPJ | 0 | 0 | 0 | 1.19 |
| RCN2 | 0 | 0 | 0 | 1.66 |
| RCN3 | 0 | 0 | 0 | 1.15 |
| RCOR2 | 0 | 0 | 0 | 2.4 |
| RDBP | 0 | 0 | 0 | 1.33 |
| RDM1 | 0 | 0 | 0 | 2.55 |
| REC8 | 0 | 0 | 0 | 1.57 |
| RECQL | 0 | 0 | 0 | 1.12 |
| RECQL5 | 0 | 0 | 0 | 1.22 |
| REEP2 | 0 | 0 | 0 | 2.32 |
| REEP4 | 0 | 0 | 0 | 1.56 |
| RELL2 | 0 | 0 | 0 | 1.12 |
| RELT | 0 | 0 | 0 | 1.1 |
| REM2 | 0 | 0 | 0 | 1.42 |
| REPS2 | 0 | 0 | 0 | -1.92 |
| RETSAT | 0 | 0 | 0 | -1.5 |
| REXO1L1 | 0 | 0 | 0 | -2.31 |
| RFC3 | 0 | 0 | 0 | 1.05 |
| RFPL1 | 0 | 0 | 0 | -2.18 |
| RFPL4A | 0 | 0 | 0 | -3.62 |
| RFT1 | 0 | 0 | 0 | 1.54 |
| RFWD3 | 0 | 0 | 0 | 1.42 |
| RFX3 | 0 | 0 | 0 | 1.42 |
| RFX5 | 0 | 0 | 0 | 1.22 |
| RFX8 | 0 | 0 | 0 | 1.88 |
| RFXANK | 0 | 0 | 0 | 1.31 |
| RFXAP | 0 | 0 | 0 | 1.05 |
| RGAG4 | 0 | 0 | 0 | -1.08 |
| RGL3 | 0 | 0 | 0 | -1.44 |
| RGS1 | 0 | 0 | 0 | 2.48 |
| RGS10 | 0 | 0 | 0 | 2.12 |
| RGS13 | 0 | 0 | 0 | 2.2 |
| RGS16 | 0 | 0 | 0 | 1.69 |
| RGS17 | 0 | 0 | 0 | 2.41 |
| RGS19 | 0 | 0 | 0 | 1.31 |
| RGS20 | 0 | 0 | 0 | 3.63 |
| RGS22 | 0 | 0 | 0 | 2.6 |
| RGS9BP | 0 | 0 | 0 | 1.46 |
| RHBG | 0 | 0 | 0 | -3.77 |
| RHEBL1 | 0 | 0 | 0 | 1.61 |
| RHOA | 0 | 0 | 0 | 1.09 |
| RHOBTB2 | 0 | 0 | 0 | 1.34 |
| RHOF | 0 | 0 | 0 | 1.84 |
| RHOH | 0 | 0 | 0 | 1.35 |
| RHOQ | 0 | 0 | 0 | 1.09 |
| RIBC1 | 0 | 0 | 0 | 1.26 |
| RIC3 | 0 | 0 | 0 | -2.02 |
| RILP | 0 | 0 | 0 | -1.54 |
| RIMBP3 | 0 | 0 | 0 | 2.5 |
| RIMKLB | 0 | 0 | 0 | 1.48 |
| RIMS2 | 0 | 0 | 0 | 3.04 |
| RIN1 | 0 | 0 | 0 | 2.6 |
| RIN2 | 0 | 0 | 0 | 1.6 |
| RIPK3 | 0 | 0 | 0 | 2.2 |
| RLN1 | 0 | 0 | 0 | 2.97 |
| RLN2 | 0 | 0 | 0 | 3.64 |
| RNASE1 | 0 | 0 | 0 | 1.65 |
| RNASE2 | 0 | 0 | 0 | 2.02 |
| RNASE3 | 0 | 0 | 0 | 1.83 |
| RNASEH2A | 0 | 0 | 0 | 1.77 |
| RNASEH2B | 0 | 0 | 0 | 1.36 |
| RNASEL | 0 | 0 | 0 | 1.36 |
| RND2 | 0 | 0 | 0 | -3.48 |
| RNF125 | 0 | 0 | 0 | -1.53 |
| RNF130 | 0 | 0 | 0 | -1.35 |
| RNF135 | 0 | 0 | 0 | 1.59 |
| RNF145 | 0 | 0 | 0 | 2.05 |
| RNF152 | 0 | 0 | 0 | -2.62 |
| RNF157 | 0 | 0 | 0 | 2.21 |
| RNF165 | 0 | 0 | 0 | -2.77 |
| RNF180 | 0 | 0 | 0 | -1.3 |
| RNF182 | 0 | 0 | 0 | 1.98 |
| RNF19B | 0 | 0 | 0 | 1.3 |
| RNF208 | 0 | 0 | 0 | -1.05 |
| RNF212 | 0 | 0 | 0 | -1.27 |
| RNF213 | 0 | 0 | 0 | 1.94 |
| RNF219 | 0 | 0 | 0 | 1.22 |
| RNF34 | 0 | 0 | 0 | 1.24 |
| RNFT1 | 0 | 0 | 0 | -1.03 |
| RNPS1 | 0 | 0 | 0 | 1.29 |
| ROBO1 | 0 | 0 | 0 | 1.41 |
| ROPN1 | 0 | 0 | 0 | -1.26 |
| ROR1 | 0 | 0 | 0 | 2.22 |
| ROR2 | 0 | 0 | 0 | 2.31 |
| RORA | 0 | 0 | 0 | -1.92 |
| RPGRIP1 | 0 | 0 | 0 | -1 |
| RPGRIP1L | 0 | 0 | 0 | 2.96 |
| RPH3AL | 0 | 0 | 0 | -1.53 |
| RPIA | 0 | 0 | 0 | 1.86 |
| RPL13A | 0 | 0 | 0 | 1.02 |
| RPL18 | 0 | 0 | 0 | 1.04 |
| RPL18A | 0 | 0 | 0 | 1.43 |
| RPL22L1 | 0 | 0 | 0 | 1.34 |
| RPL23A | 0 | 0 | 0 | 1.03 |
| RPL27A | 0 | 0 | 0 | 1.77 |
| RPL28 | 0 | 0 | 0 | 1.18 |
| RPL36A | 0 | 0 | 0 | 1.1 |
| RPL39L | 0 | 0 | 0 | 2.84 |
| RPL8 | 0 | 0 | 0 | 1.13 |
| RPRD1A | 0 | 0 | 0 | 1.04 |
| RPRML | 0 | 0 | 0 | 1.91 |
| RPS17 | 0 | 0 | 0 | -1.02 |
| RPS18 | 0 | 0 | 0 | 1.05 |
| RPS19 | 0 | 0 | 0 | 1.12 |
| RPS2 | 0 | 0 | 0 | 1.01 |
| RPS21 | 0 | 0 | 0 | 1.03 |
| RPS3 | 0 | 0 | 0 | 1.46 |
| RPS5 | 0 | 0 | 0 | 1.05 |
| RPS6KA4 | 0 | 0 | 0 | 1.38 |
| RPSA | 0 | 0 | 0 | 1.13 |
| RRAS | 0 | 0 | 0 | 1.72 |
| RRP9 | 0 | 0 | 0 | 1.07 |
| RRS1 | 0 | 0 | 0 | 1.41 |
| RSAD2 | 0 | 0 | 0 | 1.32 |
| RSC1A1 | 0 | 0 | 0 | -1.29 |
| RSPH1 | 0 | 0 | 0 | 2.89 |
| RSPH10B | 0 | 0 | 0 | -2.32 |
| RSPH3 | 0 | 0 | 0 | 1.29 |
| RTKN | 0 | 0 | 0 | -1.11 |
| RTKN2 | 0 | 0 | 0 | 3.23 |
| RTN1 | 0 | 0 | 0 | 2.32 |
| RTN2 | 0 | 0 | 0 | 1.31 |
| RTN4RL1 | 0 | 0 | 0 | -1.89 |
| RUFY4 | 0 | 0 | 0 | 1.19 |
| RUNDC1 | 0 | 0 | 0 | 1.32 |
| RUNDC3B | 0 | 0 | 0 | -3.97 |
| RUNX2 | 0 | 0 | 0 | 2.12 |
| RUNX3 | 0 | 0 | 0 | 1.68 |
| RUVBL1 | 0 | 0 | 0 | 1.47 |
| RYR1 | 0 | 0 | 0 | 1.79 |
| RYR2 | 0 | 0 | 0 | 1.67 |
| S100A4 | 0 | 0 | 0 | 2.27 |
| S100P | 0 | 0 | 0 | 6.34 |
| SACS | 0 | 0 | 0 | 1.73 |
| SAE1 | 0 | 0 | 0 | 1.44 |
| SALL1 | 0 | 0 | 0 | -6.04 |
| SAMD1 | 0 | 0 | 0 | 1.13 |
| SAMD13 | 0 | 0 | 0 | 2.88 |
| SAMD15 | 0 | 0 | 0 | 2.08 |
| SAMD4A | 0 | 0 | 0 | -1.44 |
| SAMD9 | 0 | 0 | 0 | 2.25 |
| SAMD9L | 0 | 0 | 0 | 2.08 |
| SAP30 | 0 | 0 | 0 | 1.49 |
| SASS6 | 0 | 0 | 0 | 1.48 |
| SBK1 | 0 | 0 | 0 | 1.54 |
| SBK2 | 0 | 0 | 0 | -1.55 |
| SBNO2 | 0 | 0 | 0 | 1.13 |
| SCARNA23 | 0 | 0 | 0 | 1.16 |
| SCCPDH | 0 | 0 | 0 | -1.56 |
| SCD | 0 | 0 | 0 | -1.39 |
| SCD5 | 0 | 0 | 0 | 1.7 |
| SCEL | 0 | 0 | 0 | 3.93 |
| SCG2 | 0 | 0 | 0 | 2.92 |
| SCGB2A1 | 0 | 0 | 0 | 3.4 |
| SCIN | 0 | 0 | 0 | 4.43 |
| SCLT1 | 0 | 0 | 0 | 1.06 |
| SCLY | 0 | 0 | 0 | -1.67 |
| SCML1 | 0 | 0 | 0 | -1.41 |
| SCN3B | 0 | 0 | 0 | 1.78 |
| SCN8A | 0 | 0 | 0 | -2.3 |
| SCPEP1 | 0 | 0 | 0 | 1.52 |
| SCRN1 | 0 | 0 | 0 | 3.11 |
| SCXA | 0 | 0 | 0 | 1.06 |
| SDC1 | 0 | 0 | 0 | -1.02 |
| SDC2 | 0 | 0 | 0 | -3.36 |
| SDHA | 0 | 0 | 0 | -1.46 |
| SDHB | 0 | 0 | 0 | -1.13 |
| SDHC | 0 | 0 | 0 | -1.31 |
| SDR16C5 | 0 | 0 | 0 | 3.92 |
| SEBOX | 0 | 0 | 0 | -1.25 |
| SEC14L2 | 0 | 0 | 0 | -4.01 |
| SEC14L3 | 0 | 0 | 0 | -5.58 |
| SEC14L4 | 0 | 0 | 0 | -4.86 |
| SEC16B | 0 | 0 | 0 | -2.81 |
| SECTM1 | 0 | 0 | 0 | 1.31 |
| SEL1L | 0 | 0 | 0 | -1.07 |
| SEL1L3 | 0 | 0 | 0 | 2.65 |
| SELM | 0 | 0 | 0 | 2.2 |
| SELO | 0 | 0 | 0 | -1.85 |
| SEMA3A | 0 | 0 | 0 | 3.44 |
| SEMA3E | 0 | 0 | 0 | 1.81 |
| SEMA4D | 0 | 0 | 0 | 2.19 |
| SEMA4G | 0 | 0 | 0 | -2.43 |
| SEMA7A | 0 | 0 | 0 | 2.13 |
| SEPN1 | 0 | 0 | 0 | 1.33 |
| SEPSECS | 0 | 0 | 0 | -1.64 |
| SEPW1 | 0 | 0 | 0 | 1.27 |
| SEPX1 | 0 | 0 | 0 | -1.89 |
| SERAC1 | 0 | 0 | 0 | 1.16 |
| SERF1A | 0 | 0 | 0 | 1.31 |
| SERF1B | 0 | 0 | 0 | 1.11 |
| SERINC5 | 0 | 0 | 0 | -1.32 |
| SERPINA11 | 0 | 0 | 0 | -9.19 |
| SERPINA2 | 0 | 0 | 0 | -2.83 |
| SERPINA3 | 0 | 0 | 0 | -4.7 |
| SERPINB3 | 0 | 0 | 0 | 4.71 |
| SERPINB4 | 0 | 0 | 0 | 3.88 |
| SERPINB6 | 0 | 0 | 0 | 1.24 |
| SERPINE2 | 0 | 0 | 0 | 2.97 |
| SERPINF2 | 0 | 0 | 0 | -5.82 |
| SERTAD4 | 0 | 0 | 0 | 4.04 |
| SET | 0 | 0 | 0 | 1.08 |
| SETD4 | 0 | 0 | 0 | 1.16 |
| SETP20 | 0 | 0 | 0 | 1.09 |
| SF3B4 | 0 | 0 | 0 | 1.02 |
| SFI1 | 0 | 0 | 0 | 1.69 |
| SFMBT2 | 0 | 0 | 0 | 1.57 |
| SFR1 | 0 | 0 | 0 | 1.47 |
| SFRP2 | 0 | 0 | 0 | 5.52 |
| SFT2D1 | 0 | 0 | 0 | 1.63 |
| SFTA2 | 0 | 0 | 0 | 3.66 |
| SFXN1 | 0 | 0 | 0 | -1.75 |
| SGCB | 0 | 0 | 0 | 1.32 |
| SGIP1 | 0 | 0 | 0 | 1.99 |
| SGOL1 | 0 | 0 | 0 | 2.33 |
| SH2B2 | 0 | 0 | 0 | 1.28 |
| SH2D2A | 0 | 0 | 0 | 1.94 |
| SH2D4A | 0 | 0 | 0 | -1.15 |
| SH3BGRL | 0 | 0 | 0 | 1.02 |
| SH3BGRL3 | 0 | 0 | 0 | 1.1 |
| SH3GL3 | 0 | 0 | 0 | 2.04 |
| SH3KBP1 | 0 | 0 | 0 | 1.63 |
| SH3TC2 | 0 | 0 | 0 | 2.45 |
| SH3YL1 | 0 | 0 | 0 | 1.74 |
| SHANK2 | 0 | 0 | 0 | -3.28 |
| SHANK3 | 0 | 0 | 0 | -1.05 |
| SHC2 | 0 | 0 | 0 | -2.45 |
| SHCBP1 | 0 | 0 | 0 | 5.34 |
| SHF | 0 | 0 | 0 | -2.65 |
| SHH | 0 | 0 | 0 | -1.08 |
| SHISA2 | 0 | 0 | 0 | 3.08 |
| SHKBP1 | 0 | 0 | 0 | 1.39 |
| SHMT2 | 0 | 0 | 0 | -1.42 |
| SHOX2 | 0 | 0 | 0 | 1.56 |
| SHROOM1 | 0 | 0 | 0 | -1.93 |
| SHROOM2 | 0 | 0 | 0 | -1.82 |
| SIDT1 | 0 | 0 | 0 | 2.16 |
| SIGLEC11 | 0 | 0 | 0 | -2.93 |
| SIGLEC12 | 0 | 0 | 0 | 1.81 |
| SIGMAR1 | 0 | 0 | 0 | -1.26 |
| SIM2 | 0 | 0 | 0 | 1.03 |
| SIPA1L2 | 0 | 0 | 0 | 1.48 |
| SIPA1L3 | 0 | 0 | 0 | 1.52 |
| SIRPB2 | 0 | 0 | 0 | 1.29 |
| SIRPG | 0 | 0 | 0 | 1.43 |
| SIRT3 | 0 | 0 | 0 | -1.19 |
| SIRT4 | 0 | 0 | 0 | -1.57 |
| SIRT7 | 0 | 0 | 0 | 1.1 |
| SIX1 | 0 | 0 | 0 | 5.73 |
| SKA1 | 0 | 0 | 0 | 4.06 |
| SKAP1 | 0 | 0 | 0 | -1.4 |
| SKAP2 | 0 | 0 | 0 | 1.66 |
| SLAMF7 | 0 | 0 | 0 | 1.81 |
| SLAMF8 | 0 | 0 | 0 | 2.38 |
| SLC10A2 | 0 | 0 | 0 | 1.73 |
| SLC10A3 | 0 | 0 | 0 | 1.24 |
| SLC10A4 | 0 | 0 | 0 | 1.64 |
| SLC10A5 | 0 | 0 | 0 | -3.64 |
| SLC11A1 | 0 | 0 | 0 | 2.31 |
| SLC12A1 | 0 | 0 | 0 | -1.99 |
| SLC12A9 | 0 | 0 | 0 | 1.12 |
| SLC13A3 | 0 | 0 | 0 | -2.96 |
| SLC15A2 | 0 | 0 | 0 | 1.66 |
| SLC16A10 | 0 | 0 | 0 | -2.36 |
| SLC16A12 | 0 | 0 | 0 | -2.37 |
| SLC16A13 | 0 | 0 | 0 | -2.27 |
| SLC16A5 | 0 | 0 | 0 | 2.05 |
| SLC16A6 | 0 | 0 | 0 | 1.29 |
| SLC17A9 | 0 | 0 | 0 | -2.08 |
| SLC19A2 | 0 | 0 | 0 | -1.43 |
| SLC1A1 | 0 | 0 | 0 | -2.67 |
| SLC1A3 | 0 | 0 | 0 | 1.72 |
| SLC1A4 | 0 | 0 | 0 | 1.53 |
| SLC1A5 | 0 | 0 | 0 | 3.42 |
| SLC22A14 | 0 | 0 | 0 | -1.12 |
| SLC22A16 | 0 | 0 | 0 | 2.35 |
| SLC22A18 | 0 | 0 | 0 | -1.24 |
| SLC22A23 | 0 | 0 | 0 | -1.31 |
| SLC22A24 | 0 | 0 | 0 | -1.59 |
| SLC22A25 | 0 | 0 | 0 | -3.68 |
| SLC22A3 | 0 | 0 | 0 | -5.33 |
| SLC23A1 | 0 | 0 | 0 | -4.01 |
| SLC23A2 | 0 | 0 | 0 | -2.14 |
| SLC24A3 | 0 | 0 | 0 | 3.03 |
| SLC25A1 | 0 | 0 | 0 | -1.34 |
| SLC25A12 | 0 | 0 | 0 | 2.35 |
| SLC25A16 | 0 | 0 | 0 | -1.26 |
| SLC25A19 | 0 | 0 | 0 | 1.42 |
| SLC25A23 | 0 | 0 | 0 | -1.05 |
| SLC25A30 | 0 | 0 | 0 | -1.72 |
| SLC25A34 | 0 | 0 | 0 | -1.6 |
| SLC25A35 | 0 | 0 | 0 | 1.21 |
| SLC25A36 | 0 | 0 | 0 | 2.33 |
| SLC25A4 | 0 | 0 | 0 | -1.49 |
| SLC25A6 | 0 | 0 | 0 | 1.63 |
| SLC26A1 | 0 | 0 | 0 | -2.85 |
| SLC26A6 | 0 | 0 | 0 | 1.57 |
| SLC26A9 | 0 | 0 | 0 | 3.62 |
| SLC27A3 | 0 | 0 | 0 | -1.3 |
| SLC28A3 | 0 | 0 | 0 | 2.83 |
| SLC29A2 | 0 | 0 | 0 | 1.14 |
| SLC2A10 | 0 | 0 | 0 | -1.46 |
| SLC2A11 | 0 | 0 | 0 | 1.26 |
| SLC2A14 | 0 | 0 | 0 | 1.04 |
| SLC2A3 | 0 | 0 | 0 | 2.35 |
| SLC2A4 | 0 | 0 | 0 | -2.5 |
| SLC2A5 | 0 | 0 | 0 | 4.95 |
| SLC30A1 | 0 | 0 | 0 | -1.69 |
| SLC34A1 | 0 | 0 | 0 | -2.39 |
| SLC35D1 | 0 | 0 | 0 | -2.37 |
| SLC35F3 | 0 | 0 | 0 | 3.11 |
| SLC35F5 | 0 | 0 | 0 | 1.03 |
| SLC37A1 | 0 | 0 | 0 | 2.08 |
| SLC38A1 | 0 | 0 | 0 | 2.86 |
| SLC38A5 | 0 | 0 | 0 | 1.56 |
| SLC39A4 | 0 | 0 | 0 | 2.03 |
| SLC41A1 | 0 | 0 | 0 | 1.58 |
| SLC41A3 | 0 | 0 | 0 | 1.07 |
| SLC43A2 | 0 | 0 | 0 | 1.21 |
| SLC43A3 | 0 | 0 | 0 | -1.43 |
| SLC44A2 | 0 | 0 | 0 | 1.34 |
| SLC44A4 | 0 | 0 | 0 | 5.78 |
| SLC45A1 | 0 | 0 | 0 | 1.03 |
| SLC45A4 | 0 | 0 | 0 | 2.94 |
| SLC46A1 | 0 | 0 | 0 | -1.93 |
| SLC46A3 | 0 | 0 | 0 | -1.7 |
| SLC4A10 | 0 | 0 | 0 | -1.49 |
| SLC4A5 | 0 | 0 | 0 | 1.29 |
| SLC4A7 | 0 | 0 | 0 | 1.57 |
| SLC4A8 | 0 | 0 | 0 | 1.66 |
| SLC5A6 | 0 | 0 | 0 | -1.55 |
| SLC5A9 | 0 | 0 | 0 | -2.73 |
| SLC6A14 | 0 | 0 | 0 | 3.94 |
| SLC6A2 | 0 | 0 | 0 | -1.95 |
| SLC6A20 | 0 | 0 | 0 | 3.42 |
| SLC6A5 | 0 | 0 | 0 | -1.33 |
| SLC6A9 | 0 | 0 | 0 | 2.34 |
| SLC7A5 | 0 | 0 | 0 | 1.82 |
| SLC7A6 | 0 | 0 | 0 | 2.06 |
| SLC7A6OS | 0 | 0 | 0 | 1.1 |
| SLC7A7 | 0 | 0 | 0 | 1.61 |
| SLC7A9 | 0 | 0 | 0 | -3.03 |
| SLC9A1 | 0 | 0 | 0 | 1.6 |
| SLC9B2 | 0 | 0 | 0 | -2.08 |
| SLCO1A2 | 0 | 0 | 0 | -2.86 |
| SLCO1B7 | 0 | 0 | 0 | -1.84 |
| SLCO4A1 | 0 | 0 | 0 | 3.28 |
| SLCO4C1 | 0 | 0 | 0 | -5.28 |
| SLFN11 | 0 | 0 | 0 | 1.89 |
| SLFN12 | 0 | 0 | 0 | 1.87 |
| SLFN13 | 0 | 0 | 0 | 2.23 |
| SLIT1 | 0 | 0 | 0 | -3.95 |
| SLITRK3 | 0 | 0 | 0 | -4.64 |
| SLITRK4 | 0 | 0 | 0 | 3.32 |
| SLMAP | 0 | 0 | 0 | 1.01 |
| SLN | 0 | 0 | 0 | 4.05 |
| SLX4 | 0 | 0 | 0 | 1.14 |
| SMAD3 | 0 | 0 | 0 | 1.06 |
| SMARCA4 | 0 | 0 | 0 | 1.47 |
| SMARCD3 | 0 | 0 | 0 | 1.43 |
| SMCHD1 | 0 | 0 | 0 | 1.2 |
| SMCR7L | 0 | 0 | 0 | -1.11 |
| SMO | 0 | 0 | 0 | -1.93 |
| SMPD1 | 0 | 0 | 0 | -1.73 |
| SMPD2 | 0 | 0 | 0 | 1.21 |
| SMPDL3B | 0 | 0 | 0 | 1.62 |
| SMURF2 | 0 | 0 | 0 | 1.55 |
| SNAR-C3 | 0 | 0 | 0 | -1.06 |
| SNCAIP | 0 | 0 | 0 | 1.84 |
| SNORA60 | 0 | 0 | 0 | -1.07 |
| SNPH | 0 | 0 | 0 | 1.21 |
| SNRPA | 0 | 0 | 0 | 1.3 |
| SNRPB | 0 | 0 | 0 | 1.53 |
| SNRPD2 | 0 | 0 | 0 | 1.3 |
| SNTB2 | 0 | 0 | 0 | 1.26 |
| SNTG1 | 0 | 0 | 0 | -3.5 |
| SNX12 | 0 | 0 | 0 | 1.02 |
| SNX20 | 0 | 0 | 0 | 1.63 |
| SNX22 | 0 | 0 | 0 | -1.36 |
| SNX29 | 0 | 0 | 0 | 1.11 |
| SOAT2 | 0 | 0 | 0 | -1.85 |
| SOCS4 | 0 | 0 | 0 | 1.04 |
| SOD3 | 0 | 0 | 0 | 1.54 |
| SORBS2 | 0 | 0 | 0 | -3.71 |
| SORT1 | 0 | 0 | 0 | 2.26 |
| SOS1-IT1 | 0 | 0 | 0 | 1.06 |
| SOX11 | 0 | 0 | 0 | 2.57 |
| SOX5 | 0 | 0 | 0 | -2.73 |
| SOX6 | 0 | 0 | 0 | -1.46 |
| SOX9 | 0 | 0 | 0 | 2.67 |
| SP110 | 0 | 0 | 0 | 1.23 |
| SP140 | 0 | 0 | 0 | 1.7 |
| SP140L | 0 | 0 | 0 | 1.14 |
| SP4 | 0 | 0 | 0 | 1.25 |
| SP6 | 0 | 0 | 0 | 3.04 |
| SPA17 | 0 | 0 | 0 | 2.15 |
| SPAG17 | 0 | 0 | 0 | 2.73 |
| SPAG4 | 0 | 0 | 0 | 1.78 |
| SPARC | 0 | 0 | 0 | 1.66 |
| SPATA12 | 0 | 0 | 0 | 2 |
| SPATA17 | 0 | 0 | 0 | 3.41 |
| SPATA22 | 0 | 0 | 0 | -1.11 |
| SPATA5 | 0 | 0 | 0 | 1.2 |
| SPDEF | 0 | 0 | 0 | 1.99 |
| SPDYE4 | 0 | 0 | 0 | -1.73 |
| SPEF1 | 0 | 0 | 0 | 1.47 |
| SPEF2 | 0 | 0 | 0 | 1.74 |
| SPIC | 0 | 0 | 0 | -2.05 |
| SPIN4 | 0 | 0 | 0 | 2.15 |
| SPINK13 | 0 | 0 | 0 | 2.59 |
| SPINK2 | 0 | 0 | 0 | 1.65 |
| SPINK4 | 0 | 0 | 0 | 4.75 |
| SPINK5 | 0 | 0 | 0 | 2.82 |
| SPOCK2 | 0 | 0 | 0 | 1.3 |
| SPON1 | 0 | 0 | 0 | 2.68 |
| SPRED1 | 0 | 0 | 0 | 1.81 |
| SPRED3 | 0 | 0 | 0 | 2.51 |
| SPSB1 | 0 | 0 | 0 | 1.28 |
| SPSB2 | 0 | 0 | 0 | 1.33 |
| SPSB3 | 0 | 0 | 0 | -1.01 |
| SPTBN2 | 0 | 0 | 0 | -1.14 |
| SPTLC1 | 0 | 0 | 0 | 1.23 |
| SPTLC2 | 0 | 0 | 0 | 1.42 |
| SRCIN1 | 0 | 0 | 0 | -3.01 |
| SRD5A1 | 0 | 0 | 0 | -2.27 |
| SRD5A3 | 0 | 0 | 0 | 1.62 |
| SRGAP1 | 0 | 0 | 0 | 2.45 |
| SRGAP2 | 0 | 0 | 0 | 1.01 |
| SRI | 0 | 0 | 0 | 1.84 |
| SRM | 0 | 0 | 0 | 1.06 |
| SRPK1 | 0 | 0 | 0 | 1.06 |
| SS18L1 | 0 | 0 | 0 | -1.2 |
| SSC5D | 0 | 0 | 0 | 1.63 |
| SSH1 | 0 | 0 | 0 | 1.11 |
| SSH2 | 0 | 0 | 0 | 1.05 |
| SSPN | 0 | 0 | 0 | 3.24 |
| SSRP1 | 0 | 0 | 0 | 1.19 |
| SSTR4 | 0 | 0 | 0 | -1.32 |
| ST13 | 0 | 0 | 0 | -1.03 |
| ST3GAL1 | 0 | 0 | 0 | -1.6 |
| ST3GAL4 | 0 | 0 | 0 | 1.28 |
| ST6GALNAC1 | 0 | 0 | 0 | 4.17 |
| ST6GALNAC4 | 0 | 0 | 0 | 1.23 |
| ST6GALNAC5 | 0 | 0 | 0 | 1.89 |
| ST7 | 0 | 0 | 0 | -1.13 |
| ST8SIA1 | 0 | 0 | 0 | 1.78 |
| STAC | 0 | 0 | 0 | 1.96 |
| STAG3 | 0 | 0 | 0 | -1.5 |
| STAMBPL1 | 0 | 0 | 0 | 2.09 |
| STAP1 | 0 | 0 | 0 | 2.17 |
| STARD3NL | 0 | 0 | 0 | 1.21 |
| STAT5A | 0 | 0 | 0 | 1.31 |
| STC1 | 0 | 0 | 0 | 1.9 |
| STEAP2 | 0 | 0 | 0 | 2.04 |
| STIP1 | 0 | 0 | 0 | 1.37 |
| STK11IP | 0 | 0 | 0 | 1.02 |
| STK17A | 0 | 0 | 0 | 1.15 |
| STK17B | 0 | 0 | 0 | 2.01 |
| STK32B | 0 | 0 | 0 | 2.22 |
| STK32C | 0 | 0 | 0 | 1.48 |
| STK38 | 0 | 0 | 0 | 1.1 |
| STK38L | 0 | 0 | 0 | 1.1 |
| STK4 | 0 | 0 | 0 | -1.22 |
| STMN1 | 0 | 0 | 0 | 2.52 |
| STMN2 | 0 | 0 | 0 | 2.56 |
| STMN3 | 0 | 0 | 0 | 1.64 |
| STON1 | 0 | 0 | 0 | 1.38 |
| STOX1 | 0 | 0 | 0 | 1.58 |
| STRADB | 0 | 0 | 0 | -2.22 |
| STRBP | 0 | 0 | 0 | 1.01 |
| STX1A | 0 | 0 | 0 | 2.6 |
| STX1B | 0 | 0 | 0 | -2.21 |
| STX3 | 0 | 0 | 0 | 1.27 |
| STX6 | 0 | 0 | 0 | 1.74 |
| STXBP1 | 0 | 0 | 0 | 1.53 |
| STXBP4 | 0 | 0 | 0 | 1.12 |
| STXBP5 | 0 | 0 | 0 | 1.61 |
| STYK1 | 0 | 0 | 0 | 4.88 |
| SUCLG1 | 0 | 0 | 0 | -1.02 |
| SULT1A4 | 0 | 0 | 0 | -1.73 |
| SUN3 | 0 | 0 | 0 | 1.19 |
| SUOX | 0 | 0 | 0 | -1.76 |
| SUPT16H | 0 | 0 | 0 | 1.26 |
| SUPT3H | 0 | 0 | 0 | 1.62 |
| SUSD1 | 0 | 0 | 0 | 1.21 |
| SUSD2 | 0 | 0 | 0 | 1.93 |
| SUV420H2 | 0 | 0 | 0 | 1.13 |
| SVOPL | 0 | 0 | 0 | 1.28 |
| SWAP70 | 0 | 0 | 0 | 1.34 |
| SYBU | 0 | 0 | 0 | -1.82 |
| SYCP2 | 0 | 0 | 0 | 2.72 |
| SYCP2L | 0 | 0 | 0 | 1.5 |
| SYDE2 | 0 | 0 | 0 | -1.29 |
| SYK | 0 | 0 | 0 | 2.06 |
| SYN2 | 0 | 0 | 0 | 1.99 |
| SYNC | 0 | 0 | 0 | 1.55 |
| SYNDIG1 | 0 | 0 | 0 | 5.21 |
| SYNE2 | 0 | 0 | 0 | 2.07 |
| SYNGAP1 | 0 | 0 | 0 | 1.19 |
| SYNGR2 | 0 | 0 | 0 | 1.34 |
| SYPL2 | 0 | 0 | 0 | -3.52 |
| SYT10 | 0 | 0 | 0 | -1.23 |
| SYT12 | 0 | 0 | 0 | -1.58 |
| SYT17 | 0 | 0 | 0 | -1.73 |
| SYT2 | 0 | 0 | 0 | -2.3 |
| SYT8 | 0 | 0 | 0 | 3.1 |
| SYT9 | 0 | 0 | 0 | -3.75 |
| SYTL1 | 0 | 0 | 0 | 2.62 |
| SYTL2 | 0 | 0 | 0 | 1.91 |
| SYTL4 | 0 | 0 | 0 | -1.6 |
| TACC1 | 0 | 0 | 0 | -1.25 |
| TACO1 | 0 | 0 | 0 | -1.23 |
| TACSTD2 | 0 | 0 | 0 | 3.31 |
| TAF1A | 0 | 0 | 0 | 1.2 |
| TAF1D | 0 | 0 | 0 | 1.04 |
| TAF4B | 0 | 0 | 0 | 3.26 |
| TAF6 | 0 | 0 | 0 | 1.15 |
| TAP2 | 0 | 0 | 0 | 2.16 |
| TARBP1 | 0 | 0 | 0 | 1.29 |
| TARS2 | 0 | 0 | 0 | 1.78 |
| TAS2R60 | 0 | 0 | 0 | -1.18 |
| TASP1 | 0 | 0 | 0 | 1.02 |
| TAX1BP3 | 0 | 0 | 0 | 1.87 |
| TBC1D10B | 0 | 0 | 0 | 1.14 |
| TBC1D2 | 0 | 0 | 0 | 1.02 |
| TBC1D22B | 0 | 0 | 0 | 1.08 |
| TBCD | 0 | 0 | 0 | 1.12 |
| TBX10 | 0 | 0 | 0 | -1.12 |
| TBX18 | 0 | 0 | 0 | 3.2 |
| TC2N | 0 | 0 | 0 | 2.66 |
| TCEB3C | 0 | 0 | 0 | -1.21 |
| TCERG1 | 0 | 0 | 0 | 1.19 |
| TCF12 | 0 | 0 | 0 | 1 |
| TCF19 | 0 | 0 | 0 | 2.8 |
| TCF7 | 0 | 0 | 0 | 1 |
| TCIRG1 | 0 | 0 | 0 | 1.16 |
| TCN1 | 0 | 0 | 0 | 3.84 |
| TCP10 | 0 | 0 | 0 | -3.08 |
| TCP11L1 | 0 | 0 | 0 | 1.12 |
| TCTEX1D1 | 0 | 0 | 0 | -2.78 |
| TCTEX1D2 | 0 | 0 | 0 | 2.24 |
| TCTN2 | 0 | 0 | 0 | 2.49 |
| TDGF1 | 0 | 0 | 0 | -1.76 |
| TDP1 | 0 | 0 | 0 | 1.31 |
| TDRD12 | 0 | 0 | 0 | 1.76 |
| TDRD6 | 0 | 0 | 0 | -2.43 |
| TDRKH | 0 | 0 | 0 | 1.42 |
| TEAD4 | 0 | 0 | 0 | 2.29 |
| TEF | 0 | 0 | 0 | -1.54 |
| TENC1 | 0 | 0 | 0 | -1.23 |
| TEP1 | 0 | 0 | 0 | 1.65 |
| TESC | 0 | 0 | 0 | 2.7 |
| TET1 | 0 | 0 | 0 | -1.15 |
| TEX10 | 0 | 0 | 0 | 1.26 |
| TEX11 | 0 | 0 | 0 | 1.79 |
| TEX19 | 0 | 0 | 0 | 1.37 |
| TEX22 | 0 | 0 | 0 | 1.17 |
| TFAP2A | 0 | 0 | 0 | 3.58 |
| TFAP4 | 0 | 0 | 0 | 2.02 |
| TFCP2 | 0 | 0 | 0 | 1.06 |
| TFDP1 | 0 | 0 | 0 | 1.16 |
| TFRC | 0 | 0 | 0 | 1.85 |
| TGFB1I1 | 0 | 0 | 0 | 1.53 |
| TGFB3 | 0 | 0 | 0 | 1.4 |
| TGM1 | 0 | 0 | 0 | 1.72 |
| THBS1 | 0 | 0 | 0 | 1.73 |
| THBS2 | 0 | 0 | 0 | 3.61 |
| THNSL1 | 0 | 0 | 0 | -2.02 |
| THNSL2 | 0 | 0 | 0 | -1.54 |
| THOC4 | 0 | 0 | 0 | 1.31 |
| THOP1 | 0 | 0 | 0 | -1.46 |
| THPO | 0 | 0 | 0 | -4.45 |
| THRA | 0 | 0 | 0 | 1.18 |
| THRB | 0 | 0 | 0 | -2.24 |
| THRSP | 0 | 0 | 0 | -5.43 |
| THSD1 | 0 | 0 | 0 | 1.09 |
| THUMPD2 | 0 | 0 | 0 | 1.02 |
| THY1 | 0 | 0 | 0 | 3.91 |
| TIAM2 | 0 | 0 | 0 | 2.31 |
| TIGD1 | 0 | 0 | 0 | 1.99 |
| TIGD2 | 0 | 0 | 0 | -1.76 |
| TIGD4 | 0 | 0 | 0 | 2.01 |
| TIGIT | 0 | 0 | 0 | 1.84 |
| TIMP1 | 0 | 0 | 0 | 2.28 |
| TIMP2 | 0 | 0 | 0 | 1.42 |
| TINAG | 0 | 0 | 0 | 3.19 |
| TIPIN | 0 | 0 | 0 | 1.6 |
| TKT | 0 | 0 | 0 | 1.86 |
| TLE1 | 0 | 0 | 0 | -1.17 |
| TLR5 | 0 | 0 | 0 | 1.34 |
| TLR7 | 0 | 0 | 0 | 2.18 |
| TLR9 | 0 | 0 | 0 | 1.02 |
| TM4SF1 | 0 | 0 | 0 | 1.75 |
| TM4SF19 | 0 | 0 | 0 | 2.67 |
| TM4SF4 | 0 | 0 | 0 | -3.25 |
| TM6SF1 | 0 | 0 | 0 | 1.11 |
| TM6SF2 | 0 | 0 | 0 | -1.43 |
| TM7SF2 | 0 | 0 | 0 | -2.73 |
| TMBIM6 | 0 | 0 | 0 | -1.34 |
| TMC4 | 0 | 0 | 0 | 3.2 |
| TMC5 | 0 | 0 | 0 | 6.82 |
| TMC6 | 0 | 0 | 0 | 1.74 |
| TMCC2 | 0 | 0 | 0 | 1.52 |
| TMCO3 | 0 | 0 | 0 | 1.27 |
| TMCO7 | 0 | 0 | 0 | -1.23 |
| TMED5 | 0 | 0 | 0 | -1.26 |
| TMED6 | 0 | 0 | 0 | -2.43 |
| TMEM106C | 0 | 0 | 0 | 1.3 |
| TMEM107 | 0 | 0 | 0 | 1.47 |
| TMEM108 | 0 | 0 | 0 | 1.14 |
| TMEM117 | 0 | 0 | 0 | 1.06 |
| TMEM119 | 0 | 0 | 0 | 2.49 |
| TMEM120A | 0 | 0 | 0 | -1.28 |
| TMEM125 | 0 | 0 | 0 | 3.24 |
| TMEM140 | 0 | 0 | 0 | -1.1 |
| TMEM150A | 0 | 0 | 0 | -1.01 |
| TMEM158 | 0 | 0 | 0 | 3.66 |
| TMEM159 | 0 | 0 | 0 | 2.63 |
| TMEM163 | 0 | 0 | 0 | 3.64 |
| TMEM164 | 0 | 0 | 0 | 1.73 |
| TMEM165 | 0 | 0 | 0 | 1.88 |
| TMEM168 | 0 | 0 | 0 | 1.07 |
| TMEM170B | 0 | 0 | 0 | -2.5 |
| TMEM173 | 0 | 0 | 0 | 1.53 |
| TMEM182 | 0 | 0 | 0 | 1.13 |
| TMEM192 | 0 | 0 | 0 | -1.99 |
| TMEM194A | 0 | 0 | 0 | 1.15 |
| TMEM199 | 0 | 0 | 0 | 1.01 |
| TMEM200A | 0 | 0 | 0 | 4.68 |
| TMEM205 | 0 | 0 | 0 | -1.52 |
| TMEM216 | 0 | 0 | 0 | 1.37 |
| TMEM220 | 0 | 0 | 0 | -1.55 |
| TMEM229B | 0 | 0 | 0 | 1.48 |
| TMEM231 | 0 | 0 | 0 | 1.24 |
| TMEM233 | 0 | 0 | 0 | 1.48 |
| TMEM235 | 0 | 0 | 0 | -2.46 |
| TMEM237 | 0 | 0 | 0 | 1.61 |
| TMEM26 | 0 | 0 | 0 | -1.98 |
| TMEM27 | 0 | 0 | 0 | -3.78 |
| TMEM37 | 0 | 0 | 0 | -1.46 |
| TMEM50A | 0 | 0 | 0 | 1.32 |
| TMEM57 | 0 | 0 | 0 | -1.51 |
| TMEM65 | 0 | 0 | 0 | 1.49 |
| TMEM67 | 0 | 0 | 0 | 2.9 |
| TMEM74 | 0 | 0 | 0 | -2.22 |
| TMEM82 | 0 | 0 | 0 | -1.11 |
| TMEM8B | 0 | 0 | 0 | 1.4 |
| TMEM92 | 0 | 0 | 0 | 1.72 |
| TMEM98 | 0 | 0 | 0 | 1.54 |
| TMOD2 | 0 | 0 | 0 | 1.19 |
| TMPRSS3 | 0 | 0 | 0 | 4.07 |
| TMPRSS4 | 0 | 0 | 0 | 4.81 |
| TMPRSS9 | 0 | 0 | 0 | -1.73 |
| TMSB15B | 0 | 0 | 0 | 2.72 |
| TMSB4X | 0 | 0 | 0 | 1.37 |
| TMSB4Y | 0 | 0 | 0 | -1.7 |
| TMTC2 | 0 | 0 | 0 | 3.4 |
| TNC | 0 | 0 | 0 | 2.91 |
| TNFAIP2 | 0 | 0 | 0 | 1.23 |
| TNFAIP3 | 0 | 0 | 0 | 1.38 |
| TNFAIP8 | 0 | 0 | 0 | 1.57 |
| TNFRSF10A | 0 | 0 | 0 | 1.33 |
| TNFRSF11A | 0 | 0 | 0 | 2.37 |
| TNFRSF13C | 0 | 0 | 0 | 2.08 |
| TNFRSF17 | 0 | 0 | 0 | 2.42 |
| TNFRSF18 | 0 | 0 | 0 | 3.15 |
| TNFRSF25 | 0 | 0 | 0 | 2.17 |
| TNFRSF4 | 0 | 0 | 0 | 2.05 |
| TNFRSF6B | 0 | 0 | 0 | 3.16 |
| TNFRSF8 | 0 | 0 | 0 | 1.84 |
| TNFRSF9 | 0 | 0 | 0 | 2.14 |
| TNFSF13 | 0 | 0 | 0 | 1.29 |
| TNFSF13B | 0 | 0 | 0 | 1.39 |
| TNFSF15 | 0 | 0 | 0 | 3.51 |
| TNFSF4 | 0 | 0 | 0 | 2.47 |
| TNFSF9 | 0 | 0 | 0 | 3.05 |
| TNIK | 0 | 0 | 0 | 1.06 |
| TNIP3 | 0 | 0 | 0 | 2.23 |
| TNK2 | 0 | 0 | 0 | 1.01 |
| TNNC1 | 0 | 0 | 0 | 1.48 |
| TNNC2 | 0 | 0 | 0 | 2.58 |
| TNNI2 | 0 | 0 | 0 | 2.67 |
| TNNI3 | 0 | 0 | 0 | 2.91 |
| TNS4 | 0 | 0 | 0 | 6.66 |
| TOLLIP | 0 | 0 | 0 | -1.71 |
| TOMM34 | 0 | 0 | 0 | 1.09 |
| TOMM40 | 0 | 0 | 0 | 1.15 |
| TONSL | 0 | 0 | 0 | 2.14 |
| TOPBP1 | 0 | 0 | 0 | 1.19 |
| TOR1AIP2 | 0 | 0 | 0 | -1.2 |
| TOX | 0 | 0 | 0 | 1.13 |
| TP53AIP1 | 0 | 0 | 0 | -1.06 |
| TP53BP1 | 0 | 0 | 0 | 1.56 |
| TP53I11 | 0 | 0 | 0 | 1.13 |
| TP53INP2 | 0 | 0 | 0 | -1.81 |
| TP63 | 0 | 0 | 0 | 2.7 |
| TPBG | 0 | 0 | 0 | 3.48 |
| TPD52L2 | 0 | 0 | 0 | 1.52 |
| TPMT | 0 | 0 | 0 | -1.99 |
| TPPP | 0 | 0 | 0 | -1.75 |
| TPSAB1 | 0 | 0 | 0 | 2.87 |
| TPSD1 | 0 | 0 | 0 | 2.91 |
| TPST2 | 0 | 0 | 0 | -1.32 |
| TPTE2 | 0 | 0 | 0 | -2.65 |
| TRABD | 0 | 0 | 0 | 1.07 |
| TRAF1 | 0 | 0 | 0 | 1.75 |
| TRAF3 | 0 | 0 | 0 | 1.29 |
| TRAF5 | 0 | 0 | 0 | 2.45 |
| TRAM1L1 | 0 | 0 | 0 | 2.33 |
| TRAPPC8 | 0 | 0 | 0 | -1.1 |
| TRDMT1 | 0 | 0 | 0 | 1.02 |
| TREH | 0 | 0 | 0 | -1.81 |
| TREM1 | 0 | 0 | 0 | 1.64 |
| TREML1 | 0 | 0 | 0 | 2.32 |
| TRERF1 | 0 | 0 | 0 | 1.93 |
| TRIB1 | 0 | 0 | 0 | -1.23 |
| TRIB2 | 0 | 0 | 0 | 1.39 |
| TRIL | 0 | 0 | 0 | 1.99 |
| TRIM11 | 0 | 0 | 0 | 1.08 |
| TRIM17 | 0 | 0 | 0 | 1.53 |
| TRIM24 | 0 | 0 | 0 | -1.01 |
| TRIM28 | 0 | 0 | 0 | 1.19 |
| TRIM29 | 0 | 0 | 0 | 5.42 |
| TRIM31 | 0 | 0 | 0 | 4.25 |
| TRIM32 | 0 | 0 | 0 | 1.42 |
| TRIM37 | 0 | 0 | 0 | 1.13 |
| TRIM45 | 0 | 0 | 0 | 1.61 |
| TRIM55 | 0 | 0 | 0 | -5.56 |
| TRIM62 | 0 | 0 | 0 | 1.1 |
| TRIM7 | 0 | 0 | 0 | 4.41 |
| TRIP10 | 0 | 0 | 0 | 1.39 |
| TRMT6 | 0 | 0 | 0 | 1.29 |
| TRNP1 | 0 | 0 | 0 | 2.21 |
| TRO | 0 | 0 | 0 | 1.49 |
| TRPA1 | 0 | 0 | 0 | 1.68 |
| TRPC1 | 0 | 0 | 0 | 1.61 |
| TRPC4 | 0 | 0 | 0 | 1.77 |
| TRPC5 | 0 | 0 | 0 | -1.4 |
| TRPM2 | 0 | 0 | 0 | 3.12 |
| TRPM3 | 0 | 0 | 0 | -1.2 |
| TRPM8 | 0 | 0 | 0 | -5.1 |
| TRPS1 | 0 | 0 | 0 | 1.87 |
| TRPV1 | 0 | 0 | 0 | -2.14 |
| TRPV2 | 0 | 0 | 0 | 1.33 |
| TRPV4 | 0 | 0 | 0 | -1.19 |
| TSC22D4 | 0 | 0 | 0 | 1.08 |
| TSEN15 | 0 | 0 | 0 | 1.02 |
| TSHZ3 | 0 | 0 | 0 | 1.5 |
| TSKU | 0 | 0 | 0 | -1.52 |
| TSL | 0 | 0 | 0 | -1.84 |
| TSPAN1 | 0 | 0 | 0 | 3.28 |
| TSPAN2 | 0 | 0 | 0 | 2.92 |
| TSPAN33 | 0 | 0 | 0 | -1.2 |
| TSPAN5 | 0 | 0 | 0 | 2.87 |
| TSPAN8 | 0 | 0 | 0 | 2.98 |
| TSPAN9 | 0 | 0 | 0 | -1.39 |
| TSPO | 0 | 0 | 0 | 2.03 |
| TSTD1 | 0 | 0 | 0 | -1.04 |
| TTBK1 | 0 | 0 | 0 | -2.39 |
| TTC13 | 0 | 0 | 0 | 1.16 |
| TTC16 | 0 | 0 | 0 | -1.31 |
| TTC18 | 0 | 0 | 0 | -1.22 |
| TTC22 | 0 | 0 | 0 | 1.78 |
| TTC26 | 0 | 0 | 0 | 1.71 |
| TTC38 | 0 | 0 | 0 | -2.79 |
| TTC5 | 0 | 0 | 0 | 1.1 |
| TTC6 | 0 | 0 | 0 | -2.27 |
| TTF1 | 0 | 0 | 0 | 1.13 |
| TTF2 | 0 | 0 | 0 | 2.19 |
| TTLL1 | 0 | 0 | 0 | 1.79 |
| TTLL6 | 0 | 0 | 0 | 2.27 |
| TTPAL | 0 | 0 | 0 | -1.56 |
| TUBA1A | 0 | 0 | 0 | 1.98 |
| TUBA1B | 0 | 0 | 0 | 1.79 |
| TUBA1C | 0 | 0 | 0 | 1.81 |
| TUBA4A | 0 | 0 | 0 | 1.48 |
| TUBB1 | 0 | 0 | 0 | -1.26 |
| TUBB2A | 0 | 0 | 0 | -1.25 |
| TUBB6 | 0 | 0 | 0 | 2.42 |
| TUBG1 | 0 | 0 | 0 | 1.19 |
| TULP4 | 0 | 0 | 0 | 1.1 |
| TWIST2 | 0 | 0 | 0 | 2.23 |
| TWISTNB | 0 | 0 | 0 | 1.02 |
| TWSG1 | 0 | 0 | 0 | 1.42 |
| TXNDC3 | 0 | 0 | 0 | 1.42 |
| TXNRD2 | 0 | 0 | 0 | -1.74 |
| TYRO3 | 0 | 0 | 0 | 1.19 |
| TYRP1 | 0 | 0 | 0 | 2.95 |
| UACA | 0 | 0 | 0 | 1.37 |
| UBAP2 | 0 | 0 | 0 | 1.29 |
| UBE2A | 0 | 0 | 0 | 1.05 |
| UBE2Q2 | 0 | 0 | 0 | 1.39 |
| UBE2S | 0 | 0 | 0 | 2.62 |
| UBXN10 | 0 | 0 | 0 | -2.23 |
| UCHL1 | 0 | 0 | 0 | 4.06 |
| UCK1 | 0 | 0 | 0 | -1.03 |
| UCN2 | 0 | 0 | 0 | 3.25 |
| UGT2A2 | 0 | 0 | 0 | -3.02 |
| UGT8 | 0 | 0 | 0 | 3.72 |
| UHRF1BP1L | 0 | 0 | 0 | -1.17 |
| ULBP2 | 0 | 0 | 0 | 3.27 |
| ULBP3 | 0 | 0 | 0 | 2.68 |
| UNC13C | 0 | 0 | 0 | 1.11 |
| UNC13D | 0 | 0 | 0 | 1.19 |
| UNC5CL | 0 | 0 | 0 | -1.8 |
| UNC93A | 0 | 0 | 0 | -4.05 |
| UPK1B | 0 | 0 | 0 | 3.4 |
| UPP2 | 0 | 0 | 0 | -6.04 |
| UQCR10 | 0 | 0 | 0 | -1.14 |
| UQCRB | 0 | 0 | 0 | -1.12 |
| UQCRQ | 0 | 0 | 0 | -1.13 |
| UROD | 0 | 0 | 0 | -1.04 |
| USP1 | 0 | 0 | 0 | 1.17 |
| USP2 | 0 | 0 | 0 | -2.66 |
| USP3 | 0 | 0 | 0 | 1.05 |
| USP44 | 0 | 0 | 0 | 1.91 |
| USP46 | 0 | 0 | 0 | 1.24 |
| USP49 | 0 | 0 | 0 | 1.5 |
| USP54 | 0 | 0 | 0 | 2 |
| UTS2 | 0 | 0 | 0 | 2.4 |
| UXS1 | 0 | 0 | 0 | 1.23 |
| VANGL1 | 0 | 0 | 0 | 1.27 |
| VANGL2 | 0 | 0 | 0 | 2.92 |
| VASH1 | 0 | 0 | 0 | 1.01 |
| VAV3 | 0 | 0 | 0 | 1.04 |
| VAX2 | 0 | 0 | 0 | 1.06 |
| VEGFC | 0 | 0 | 0 | 1.6 |
| VEZF1 | 0 | 0 | 0 | 1.11 |
| VGF | 0 | 0 | 0 | 5.92 |
| VGLL4 | 0 | 0 | 0 | 1.12 |
| VILL | 0 | 0 | 0 | 3.28 |
| VIM | 0 | 0 | 0 | 1.58 |
| VIPR1 | 0 | 0 | 0 | -3.36 |
| VMA21 | 0 | 0 | 0 | 1.03 |
| VMP1 | 0 | 0 | 0 | 1.38 |
| VNN1 | 0 | 0 | 0 | -2.78 |
| VOPP1 | 0 | 0 | 0 | 1.43 |
| VPS35 | 0 | 0 | 0 | 1.09 |
| VPS37B | 0 | 0 | 0 | 1.55 |
| VPS37C | 0 | 0 | 0 | 1.25 |
| VPS72 | 0 | 0 | 0 | 1 |
| VRK1 | 0 | 0 | 0 | 1.75 |
| VSIG1 | 0 | 0 | 0 | 2.46 |
| VSIG8 | 0 | 0 | 0 | 2.81 |
| VSX1 | 0 | 0 | 0 | -2.31 |
| VWA5B1 | 0 | 0 | 0 | -1.13 |
| WARS | 0 | 0 | 0 | 1.68 |
| WASF2 | 0 | 0 | 0 | 1.05 |
| WBP2NL | 0 | 0 | 0 | 1.46 |
| WBP5 | 0 | 0 | 0 | 1.45 |
| WDR47 | 0 | 0 | 0 | 1.28 |
| WDR54 | 0 | 0 | 0 | 2.44 |
| WDR63 | 0 | 0 | 0 | 1.97 |
| WDR67 | 0 | 0 | 0 | 1.18 |
| WDR72 | 0 | 0 | 0 | -2.8 |
| WDR76 | 0 | 0 | 0 | 3.37 |
| WDR96 | 0 | 0 | 0 | 1.33 |
| WFDC10B | 0 | 0 | 0 | 2.85 |
| WFDC2 | 0 | 0 | 0 | 3.13 |
| WFDC5 | 0 | 0 | 0 | -2.43 |
| WFIKKN1 | 0 | 0 | 0 | -1.35 |
| WFIKKN2 | 0 | 0 | 0 | -1.38 |
| WIPF1 | 0 | 0 | 0 | 1.25 |
| WIPF3 | 0 | 0 | 0 | 2.45 |
| WISP1 | 0 | 0 | 0 | 3.63 |
| WLS | 0 | 0 | 0 | 1.42 |
| WNK3 | 0 | 0 | 0 | -3.77 |
| WNT10A | 0 | 0 | 0 | 2.85 |
| WNT2B | 0 | 0 | 0 | 1.77 |
| WNT3 | 0 | 0 | 0 | -1.73 |
| WNT7A | 0 | 0 | 0 | 2.03 |
| WNT7B | 0 | 0 | 0 | 2.01 |
| WRAP53 | 0 | 0 | 0 | 1.21 |
| WRAP73 | 0 | 0 | 0 | 1.21 |
| WTAP | 0 | 0 | 0 | 1.07 |
| WTIP | 0 | 0 | 0 | 1.59 |
| WWC2 | 0 | 0 | 0 | -1.33 |
| WWC3 | 0 | 0 | 0 | 1.13 |
| WWP1 | 0 | 0 | 0 | -1.14 |
| WWP2 | 0 | 0 | 0 | 1 |
| WWTR1 | 0 | 0 | 0 | 1.27 |
| XAGE3 | 0 | 0 | 0 | -3.65 |
| XKR6 | 0 | 0 | 0 | 2.69 |
| XKRX | 0 | 0 | 0 | 1.94 |
| XLOC_000011 | 0 | 0 | 0 | 1.39 |
| XLOC_000222 | 0 | 0 | 0 | -2.39 |
| XLOC_000264 | 0 | 0 | 0 | -3.54 |
| XLOC_000527 | 0 | 0 | 0 | 2.01 |
| XLOC_000643 | 0 | 0 | 0 | -1.95 |
| XLOC_000812 | 0 | 0 | 0 | 1.67 |
| XLOC_001070 | 0 | 0 | 0 | -2.05 |
| XLOC_001265 | 0 | 0 | 0 | -1.61 |
| XLOC_001532 | 0 | 0 | 0 | 1.68 |
| XLOC_001625 | 0 | 0 | 0 | 3.16 |
| XLOC_002076 | 0 | 0 | 0 | 2.26 |
| XLOC_002151 | 0 | 0 | 0 | 1.78 |
| XLOC_002643 | 0 | 0 | 0 | 1.26 |
| XLOC_002867 | 0 | 0 | 0 | 2.72 |
| XLOC_002934 | 0 | 0 | 0 | -3.23 |
| XLOC_002997 | 0 | 0 | 0 | -1.28 |
| XLOC_003406 | 0 | 0 | 0 | 1.06 |
| XLOC_003572 | 0 | 0 | 0 | -7.53 |
| XLOC_003775 | 0 | 0 | 0 | -3.15 |
| XLOC_004205 | 0 | 0 | 0 | -3.87 |
| XLOC_004323 | 0 | 0 | 0 | -2.35 |
| XLOC_004341 | 0 | 0 | 0 | -2.06 |
| XLOC_005174 | 0 | 0 | 0 | -2.28 |
| XLOC_005263 | 0 | 0 | 0 | -1.53 |
| XLOC_005315 | 0 | 0 | 0 | -5.89 |
| XLOC_005421 | 0 | 0 | 0 | -1.75 |
| XLOC_005517 | 0 | 0 | 0 | 1.06 |
| XLOC_005935 | 0 | 0 | 0 | 1.92 |
| XLOC_006178 | 0 | 0 | 0 | -1.01 |
| XLOC_006704 | 0 | 0 | 0 | -2.99 |
| XLOC_006756 | 0 | 0 | 0 | -3.86 |
| XLOC_007052 | 0 | 0 | 0 | 2.75 |
| XLOC_007085 | 0 | 0 | 0 | 4.39 |
| XLOC_007093 | 0 | 0 | 0 | -2.08 |
| XLOC_007123 | 0 | 0 | 0 | 3.24 |
| XLOC_007221 | 0 | 0 | 0 | -2.82 |
| XLOC_007290 | 0 | 0 | 0 | -2 |
| XLOC_007388 | 0 | 0 | 0 | 1.04 |
| XLOC_007458 | 0 | 0 | 0 | 1.54 |
| XLOC_007556 | 0 | 0 | 0 | 1.14 |
| XLOC_008079 | 0 | 0 | 0 | 2.15 |
| XLOC_008805 | 0 | 0 | 0 | -4.82 |
| XLOC_009181 | 0 | 0 | 0 | -3.97 |
| XLOC_009280 | 0 | 0 | 0 | -1.24 |
| XLOC_009791 | 0 | 0 | 0 | 2.76 |
| XLOC_010390 | 0 | 0 | 0 | 2.33 |
| XLOC_010552 | 0 | 0 | 0 | -1.35 |
| XLOC_010897 | 0 | 0 | 0 | -5.25 |
| XLOC_010962 | 0 | 0 | 0 | 2.53 |
| XLOC_011043 | 0 | 0 | 0 | -2.11 |
| XLOC_011072 | 0 | 0 | 0 | -1.11 |
| XLOC_011117 | 0 | 0 | 0 | 1.75 |
| XLOC_011181 | 0 | 0 | 0 | -3.2 |
| XLOC_012139 | 0 | 0 | 0 | 1.68 |
| XLOC_012170 | 0 | 0 | 0 | 1.67 |
| XLOC_012593 | 0 | 0 | 0 | -2.3 |
| XLOC_013717 | 0 | 0 | 0 | 1.64 |
| XLOC_014388 | 0 | 0 | 0 | 2.15 |
| XLOC_014399 | 0 | 0 | 0 | 4.15 |
| XLOC_014508 | 0 | 0 | 0 | 2.79 |
| XLOC_l2_000080 | 0 | 0 | 0 | -4.25 |
| XLOC_l2_000604 | 0 | 0 | 0 | -1.86 |
| XLOC_l2_000920 | 0 | 0 | 0 | -1.46 |
| XLOC_l2_001011 | 0 | 0 | 0 | 2.16 |
| XLOC_l2_001064 | 0 | 0 | 0 | 2.59 |
| XLOC_l2_001134 | 0 | 0 | 0 | 2.38 |
| XLOC_l2_001222 | 0 | 0 | 0 | -4.42 |
| XLOC_l2_001592 | 0 | 0 | 0 | -1.11 |
| XLOC_l2_001875 | 0 | 0 | 0 | -1.01 |
| XLOC_l2_002884 | 0 | 0 | 0 | 2.56 |
| XLOC_l2_003188 | 0 | 0 | 0 | 1.18 |
| XLOC_l2_003293 | 0 | 0 | 0 | 1.93 |
| XLOC_l2_003897 | 0 | 0 | 0 | 1.62 |
| XLOC_l2_004594 | 0 | 0 | 0 | 1.01 |
| XLOC_l2_004859 | 0 | 0 | 0 | -1.13 |
| XLOC_l2_006648 | 0 | 0 | 0 | 1.27 |
| XLOC_l2_007884 | 0 | 0 | 0 | 1.67 |
| XLOC_l2_008008 | 0 | 0 | 0 | 2.53 |
| XLOC_l2_008124 | 0 | 0 | 0 | 1.27 |
| XLOC_l2_008203 | 0 | 0 | 0 | 2.28 |
| XLOC_l2_008221 | 0 | 0 | 0 | 1.64 |
| XLOC_l2_008261 | 0 | 0 | 0 | 2.79 |
| XLOC_l2_009140 | 0 | 0 | 0 | -3.51 |
| XLOC_l2_009159 | 0 | 0 | 0 | -4.06 |
| XLOC_l2_009292 | 0 | 0 | 0 | -4.33 |
| XLOC_l2_009441 | 0 | 0 | 0 | 2.23 |
| XLOC_l2_011793 | 0 | 0 | 0 | 1.73 |
| XLOC_l2_011873 | 0 | 0 | 0 | -3.3 |
| XLOC_l2_012473 | 0 | 0 | 0 | 2.01 |
| XLOC_l2_013125 | 0 | 0 | 0 | -1.54 |
| XLOC_l2_013301 | 0 | 0 | 0 | 1.91 |
| XLOC_l2_013436 | 0 | 0 | 0 | -1.93 |
| XLOC_l2_013506 | 0 | 0 | 0 | 1.72 |
| XLOC_l2_013751 | 0 | 0 | 0 | 1.68 |
| XLOC_l2_014077 | 0 | 0 | 0 | -1.76 |
| XLOC_l2_014697 | 0 | 0 | 0 | -3.65 |
| XLOC_l2_014797 | 0 | 0 | 0 | 1.55 |
| XLOC_l2_014821 | 0 | 0 | 0 | 1.36 |
| XLOC_l2_015239 | 0 | 0 | 0 | -2.92 |
| XLOC_l2_015752 | 0 | 0 | 0 | 2.22 |
| XPNPEP2 | 0 | 0 | 0 | -1.83 |
| XPO5 | 0 | 0 | 0 | 1.14 |
| XPR1 | 0 | 0 | 0 | 1.48 |
| XRCC1 | 0 | 0 | 0 | 1.17 |
| XRCC3 | 0 | 0 | 0 | 2.02 |
| XRCC4 | 0 | 0 | 0 | 1.26 |
| XYLB | 0 | 0 | 0 | -2.75 |
| XYLT1 | 0 | 0 | 0 | 3.43 |
| XYLT2 | 0 | 0 | 0 | 1.04 |
| YEATS2 | 0 | 0 | 0 | 1.52 |
| YIF1B | 0 | 0 | 0 | 4.53 |
| YKT6 | 0 | 0 | 0 | 1.63 |
| YWHAB | 0 | 0 | 0 | 1.21 |
| YWHAH | 0 | 0 | 0 | 1.48 |
| YWHAQ | 0 | 0 | 0 | 1.1 |
| ZBED3 | 0 | 0 | 0 | -1.05 |
| ZBED5 | 0 | 0 | 0 | 1.3 |
| ZBP1 | 0 | 0 | 0 | 2.29 |
| ZBTB20 | 0 | 0 | 0 | -1.38 |
| ZBTB24 | 0 | 0 | 0 | 1.43 |
| ZBTB26 | 0 | 0 | 0 | 1.75 |
| ZBTB8A | 0 | 0 | 0 | 1.02 |
| ZBTB8OS | 0 | 0 | 0 | 1.01 |
| ZBTB9 | 0 | 0 | 0 | 1.1 |
| ZC3H12D | 0 | 0 | 0 | 1.61 |
| ZC3H13 | 0 | 0 | 0 | -1.67 |
| ZC3H15 | 0 | 0 | 0 | 1.14 |
| ZCCHC16 | 0 | 0 | 0 | -3.83 |
| ZCCHC18 | 0 | 0 | 0 | 1.26 |
| ZCCHC2 | 0 | 0 | 0 | -1.17 |
| ZDHHC1 | 0 | 0 | 0 | 1.05 |
| ZDHHC20 | 0 | 0 | 0 | 1.08 |
| ZDHHC7 | 0 | 0 | 0 | 1.29 |
| ZFAND2A | 0 | 0 | 0 | -1.06 |
| ZFAT | 0 | 0 | 0 | 1.26 |
| ZFHX2 | 0 | 0 | 0 | -1.84 |
| ZFP1 | 0 | 0 | 0 | -1.78 |
| ZFP112 | 0 | 0 | 0 | 1.17 |
| ZFP36L2 | 0 | 0 | 0 | 1.33 |
| ZFP57 | 0 | 0 | 0 | 1.84 |
| ZFP62 | 0 | 0 | 0 | 1.31 |
| ZFP64 | 0 | 0 | 0 | 1.35 |
| ZFP82 | 0 | 0 | 0 | 1.32 |
| ZFP90 | 0 | 0 | 0 | 1.07 |
| ZG16B | 0 | 0 | 0 | 3.18 |
| ZHX1-C8ORF76 | 0 | 0 | 0 | 1.27 |
| ZIM2 | 0 | 0 | 0 | -2.84 |
| ZMIZ2 | 0 | 0 | 0 | 1.44 |
| ZMYND12 | 0 | 0 | 0 | -1.82 |
| ZMYND19 | 0 | 0 | 0 | 1.25 |
| ZNF100 | 0 | 0 | 0 | 1.18 |
| ZNF107 | 0 | 0 | 0 | 1.64 |
| ZNF117 | 0 | 0 | 0 | 1.6 |
| ZNF14 | 0 | 0 | 0 | 1.88 |
| ZNF157 | 0 | 0 | 0 | -1.6 |
| ZNF169 | 0 | 0 | 0 | 1.1 |
| ZNF184 | 0 | 0 | 0 | 1.14 |
| ZNF185 | 0 | 0 | 0 | 2.35 |
| ZNF192P1 | 0 | 0 | 0 | 1.15 |
| ZNF202 | 0 | 0 | 0 | 1.01 |
| ZNF207 | 0 | 0 | 0 | 1.08 |
| ZNF213 | 0 | 0 | 0 | 1.24 |
| ZNF215 | 0 | 0 | 0 | 2.68 |
| ZNF233 | 0 | 0 | 0 | 2.05 |
| ZNF248 | 0 | 0 | 0 | 1.05 |
| ZNF256 | 0 | 0 | 0 | 1.37 |
| ZNF257 | 0 | 0 | 0 | 1.74 |
| ZNF267 | 0 | 0 | 0 | 1.53 |
| ZNF273 | 0 | 0 | 0 | 1.26 |
| ZNF28 | 0 | 0 | 0 | 1.82 |
| ZNF280A | 0 | 0 | 0 | 2.8 |
| ZNF280B | 0 | 0 | 0 | 2.27 |
| ZNF280C | 0 | 0 | 0 | 1.29 |
| ZNF282 | 0 | 0 | 0 | 1.14 |
| ZNF283 | 0 | 0 | 0 | 1.03 |
| ZNF284 | 0 | 0 | 0 | 1.26 |
| ZNF286A | 0 | 0 | 0 | 1.53 |
| ZNF286B | 0 | 0 | 0 | 1.2 |
| ZNF287 | 0 | 0 | 0 | 2.21 |
| ZNF292 | 0 | 0 | 0 | 1.11 |
| ZNF300 | 0 | 0 | 0 | 1.9 |
| ZNF341 | 0 | 0 | 0 | 1.01 |
| ZNF346 | 0 | 0 | 0 | 1.19 |
| ZNF35 | 0 | 0 | 0 | 1.1 |
| ZNF354A | 0 | 0 | 0 | 1.06 |
| ZNF385B | 0 | 0 | 0 | -4.07 |
| ZNF385C | 0 | 0 | 0 | 1.33 |
| ZNF391 | 0 | 0 | 0 | 2.44 |
| ZNF430 | 0 | 0 | 0 | 2.25 |
| ZNF431 | 0 | 0 | 0 | 2.23 |
| ZNF432 | 0 | 0 | 0 | 1.42 |
| ZNF439 | 0 | 0 | 0 | 1.52 |
| ZNF461 | 0 | 0 | 0 | 1.21 |
| ZNF462 | 0 | 0 | 0 | 1.38 |
| ZNF468 | 0 | 0 | 0 | 1.22 |
| ZNF474 | 0 | 0 | 0 | 1.62 |
| ZNF486 | 0 | 0 | 0 | 1.54 |
| ZNF493 | 0 | 0 | 0 | 1.22 |
| ZNF501 | 0 | 0 | 0 | 1.22 |
| ZNF506 | 0 | 0 | 0 | 1.17 |
| ZNF512 | 0 | 0 | 0 | 1.13 |
| ZNF512B | 0 | 0 | 0 | -1.12 |
| ZNF519 | 0 | 0 | 0 | 1.76 |
| ZNF521 | 0 | 0 | 0 | 1.64 |
| ZNF546 | 0 | 0 | 0 | -1.25 |
| ZNF547 | 0 | 0 | 0 | 1.01 |
| ZNF551 | 0 | 0 | 0 | 1.42 |
| ZNF554 | 0 | 0 | 0 | -1.07 |
| ZNF563 | 0 | 0 | 0 | -1.12 |
| ZNF578 | 0 | 0 | 0 | 1.15 |
| ZNF587 | 0 | 0 | 0 | 1.02 |
| ZNF589 | 0 | 0 | 0 | 1.24 |
| ZNF607 | 0 | 0 | 0 | 1.76 |
| ZNF614 | 0 | 0 | 0 | 1 |
| ZNF643 | 0 | 0 | 0 | 1.11 |
| ZNF646 | 0 | 0 | 0 | -1.07 |
| ZNF662 | 0 | 0 | 0 | -1.19 |
| ZNF668 | 0 | 0 | 0 | 1.26 |
| ZNF678 | 0 | 0 | 0 | 1.17 |
| ZNF681 | 0 | 0 | 0 | 3.12 |
| ZNF683 | 0 | 0 | 0 | 2.18 |
| ZNF684 | 0 | 0 | 0 | -1.92 |
| ZNF695 | 0 | 0 | 0 | 1.79 |
| ZNF699 | 0 | 0 | 0 | 1.64 |
| ZNF70 | 0 | 0 | 0 | 1.24 |
| ZNF701 | 0 | 0 | 0 | 2.33 |
| ZNF703 | 0 | 0 | 0 | 2.09 |
| ZNF707 | 0 | 0 | 0 | 1.19 |
| ZNF709 | 0 | 0 | 0 | 1.39 |
| ZNF711 | 0 | 0 | 0 | 1.18 |
| ZNF765 | 0 | 0 | 0 | 1.17 |
| ZNF766 | 0 | 0 | 0 | 1.06 |
| ZNF774 | 0 | 0 | 0 | 1.07 |
| ZNF775 | 0 | 0 | 0 | 1.57 |
| ZNF783 | 0 | 0 | 0 | 1.59 |
| ZNF789 | 0 | 0 | 0 | 1 |
| ZNF793 | 0 | 0 | 0 | 1.7 |
| ZNF8 | 0 | 0 | 0 | 1.14 |
| ZNF81 | 0 | 0 | 0 | 1.13 |
| ZNF813 | 0 | 0 | 0 | 1.24 |
| ZNF816 | 0 | 0 | 0 | 1.71 |
| ZNF816-ZNF321P | 0 | 0 | 0 | 2.48 |
| ZNF827 | 0 | 0 | 0 | 3.23 |
| ZNF836 | 0 | 0 | 0 | 1.01 |
| ZNF841 | 0 | 0 | 0 | 1.11 |
| ZNF850 | 0 | 0 | 0 | 1.05 |
| ZNF880 | 0 | 0 | 0 | 1.04 |
| ZNF92 | 0 | 0 | 0 | 1.14 |
| ZNF93 | 0 | 0 | 0 | 1.34 |
| ZNFX1 | 0 | 0 | 0 | 1.1 |
| ZNHIT1 | 0 | 0 | 0 | -1.09 |
| ZNHIT6 | 0 | 0 | 0 | 1.04 |
| ZNRF1 | 0 | 0 | 0 | 1.02 |
| ZP1 | 0 | 0 | 0 | 1.62 |
| ZP3 | 0 | 0 | 0 | 2.77 |
| ZP4 | 0 | 0 | 0 | -1.08 |
| ZPBP | 0 | 0 | 0 | 1.26 |
| ZRSR2 | 0 | 0 | 0 | 1.27 |
| ZSWIM1 | 0 | 0 | 0 | 1.19 |
| ZSWIM4 | 0 | 0 | 0 | 1.51 |
| ZWILCH | 0 | 0 | 0 | 1.96 |
| ZYG11A | 0 | 0 | 0 | -2.65 |
| ZYX | 0 | 0 | 0 | 1.44 |
