# Supplementary material for: Mining of RNA Methylation-Related Genes and Elucidation of Their Molecular Biology in Gallbladder Carcinoma
Source: Front Oncol. 2021 Feb 25;11:621806. doi: 10.3389/fonc.2021.621806 (PMC7947712; doi:10.3389/fonc.2021.621806)
Supplement: Supplementary file 4 [file Table_2.doc]

**Supplementary Table 2. Correlation coefficients among 17 hub genes from the CHOL data set of TCGA by Pearson statistical analysis.**

| ***Gene*** | ***AKR1C4*** | ***CFH*** | ***CFHR3*** | ***CTH*** | ***ENPP1*** | ***EPHX2*** | ***F2*** | ***FGA*** | ***GNMT*** | ***HAO1*** | ***HPX*** | ***HSD17B6*** | ***ITIH4*** | ***MAT1A*** | ***MTHFD1*** | ***NAT2*** | ***PIPOX*** |
| --- | --- | --- | --- | --- | --- | --- | --- | --- | --- | --- | --- | --- | --- | --- | --- | --- | --- |
| ***AKR1C4*** | 0 | 0.78 | 0.65 | 0.83 | 0.84 | 0.77 | 0.89 | 0.83 | 0.8 | 0.94 | 0.89 | 0.95 | 0.83 | 0.87 | 0.91 | 0.94 | 0.95 |
| ***CFH*** | 0.78 | 0 | 0.79 | 0.88 | 0.91 | 0.74 | 0.88 | 0.85 | 0.71 | 0.88 | 0.9 | 0.91 | 0.8 | 0.86 | 0.83 | 0.8 | 0.88 |
| ***CFHR3*** | 0.65 | 0.79 | 0 | 0.88 | 0.66 | 0.76 | 0.79 | 0.85 | 0.71 | 0.77 | 0.86 | 0.77 | 0.81 | 0.83 | 0.75 | 0.73 | 0.73 |
| ***CTH*** | 0.83 | 0.88 | 0.88 | 0 | 0.83 | 0.78 | 0.89 | 0.94 | 0.78 | 0.9 | 0.94 | 0.91 | 0.87 | 0.95 | 0.88 | 0.85 | 0.88 |
| ***ENPP1*** | 0.84 | 0.91 | 0.66 | 0.83 | 0 | 0.8 | 0.88 | 0.78 | 0.75 | 0.9 | 0.87 | 0.92 | 0.77 | 0.83 | 0.83 | 0.81 | 0.92 |
| ***EPHX2*** | 0.77 | 0.74 | 0.76 | 0.78 | 0.8 | 0 | 0.85 | 0.75 | 0.79 | 0.86 | 0.83 | 0.82 | 0.8 | 0.8 | 0.8 | 0.82 | 0.84 |
| ***F2*** | 0.89 | 0.88 | 0.79 | 0.89 | 0.88 | 0.85 | 0 | 0.92 | 0.9 | 0.97 | 0.97 | 0.95 | 0.96 | 0.96 | 0.91 | 0.92 | 0.96 |
| ***FGA*** | 0.83 | 0.85 | 0.85 | 0.94 | 0.78 | 0.75 | 0.92 | 0 | 0.79 | 0.87 | 0.97 | 0.89 | 0.95 | 0.95 | 0.86 | 0.87 | 0.85 |
| ***GNMT*** | 0.8 | 0.71 | 0.71 | 0.78 | 0.75 | 0.79 | 0.9 | 0.79 | 0 | 0.86 | 0.85 | 0.82 | 0.88 | 0.86 | 0.82 | 0.81 | 0.86 |
| ***HAO1*** | 0.94 | 0.88 | 0.77 | 0.9 | 0.9 | 0.86 | 0.97 | 0.87 | 0.86 | 0 | 0.95 | 0.97 | 0.89 | 0.93 | 0.93 | 0.96 | 0.99 |
| ***HPX*** | 0.89 | 0.9 | 0.86 | 0.94 | 0.87 | 0.83 | 0.97 | 0.97 | 0.85 | 0.95 | 0 | 0.96 | 0.96 | 0.96 | 0.9 | 0.93 | 0.94 |
| ***HSD17B6*** | 0.95 | 0.91 | 0.77 | 0.91 | 0.92 | 0.82 | 0.95 | 0.89 | 0.82 | 0.97 | 0.96 | 0 | 0.88 | 0.92 | 0.91 | 0.93 | 0.97 |
| ***ITIH4*** | 0.83 | 0.8 | 0.81 | 0.87 | 0.77 | 0.8 | 0.96 | 0.95 | 0.88 | 0.89 | 0.96 | 0.88 | 0 | 0.95 | 0.87 | 0.9 | 0.88 |
| ***MAT1A*** | 0.87 | 0.86 | 0.83 | 0.95 | 0.83 | 0.8 | 0.96 | 0.95 | 0.86 | 0.93 | 0.96 | 0.92 | 0.95 | 0 | 0.89 | 0.89 | 0.9 |
| ***MTHFD1*** | 0.91 | 0.83 | 0.75 | 0.88 | 0.83 | 0.8 | 0.91 | 0.86 | 0.82 | 0.93 | 0.9 | 0.91 | 0.87 | 0.89 | 0 | 0.92 | 0.93 |
| ***NAT2*** | 0.94 | 0.8 | 0.73 | 0.85 | 0.81 | 0.82 | 0.92 | 0.87 | 0.81 | 0.96 | 0.93 | 0.93 | 0.9 | 0.89 | 0.92 | 0 | 0.95 |
| ***PIPOX*** | 0.95 | 0.88 | 0.73 | 0.88 | 0.92 | 0.84 | 0.96 | 0.85 | 0.86 | 0.99 | 0.94 | 0.97 | 0.88 | 0.9 | 0.93 | 0.95 | 0 |
